# 3,3'-Linked BINOL macrocycles: optimized synthesis of crown ethers featuring one or two BINOL units

**DOI:** 10.3762/bjoc.21.134

**Published:** 2025-08-28

**Authors:** Somayyeh Kheirjou, Jan Riebe, Maike Thiele, Christoph Wölper, Jochen Niemeyer

**Affiliations:** 1 Faculty of Chemistry (Organic Chemistry) and Center for Nanointegration Duisburg-Essen (CENIDE), University of Duisburg-Essen, 45141 Essen, Germany; 2 Faculty of Chemistry (Inorganic Chemistry), University of Duisburg-Essen, 45141 Essen, Germany

**Keywords:** BINOL, chirality, crown ethers, macrocycles, supramolecular chemistry

## Abstract

Chiral macrocycles hold significant importance in various scientific fields due to their unique structural and chemical properties. By controlling their size, shape, and substituents, chiral macrocycles offer a platform for designing and synthesizing highly efficient catalysts, chemosensors, and functional materials. We have recently made strides in developing macrocyclic organocatalysts; however, their synthesis remains challenging. In this work, we aimed to discover a straightforward method for producing a diverse range of chiral macrocycles, thereby enabling further exploration in the field of interlocked and macrocyclic organocatalysts. We successfully established optimized synthetic routes for the synthesis of chiral macrocycles containing one or two stereogenic units, featuring varying ring sizes and substituents (21 examples in total).

## Introduction

Crown ethers are at the heart of supramolecular chemistry [1]. Ever since their discovery in 1960, a vast number of different crown ethers has been synthesized and their interactions with guest molecules have been studied. The pioneering works in this area by Cram, Lehn and Pedersen marked the beginning of modern supramolecular chemistry and were honoured with the Nobel Prize in Chemistry in 1987 [2–4]. Soon, it was realized that chiral crown ethers are highly promising host molecules for enantioselective molecular recognition. Different chiral backbones were used for the construction of such chiral crown ethers, especially chiral 1,2-diols such as tartaric acid [5–8], propane-1,2-diol [9–13], cyclohexane-1,2-diol [14], carbohydrates [15], 1,1'-binapthyl-2,2'-diol (BINOL) [16–23] and more [24]. Especially BINOL-based crown ethers proved to be highly useful and were applied for stereoselective molecular recognition [25–27], for catalysis [25,28–31], as stationary phases for chromatography [32–34], but also as building blocks for incorporation into larger frameworks, such as interlocked molecules [25,35].

**Figure 1:**
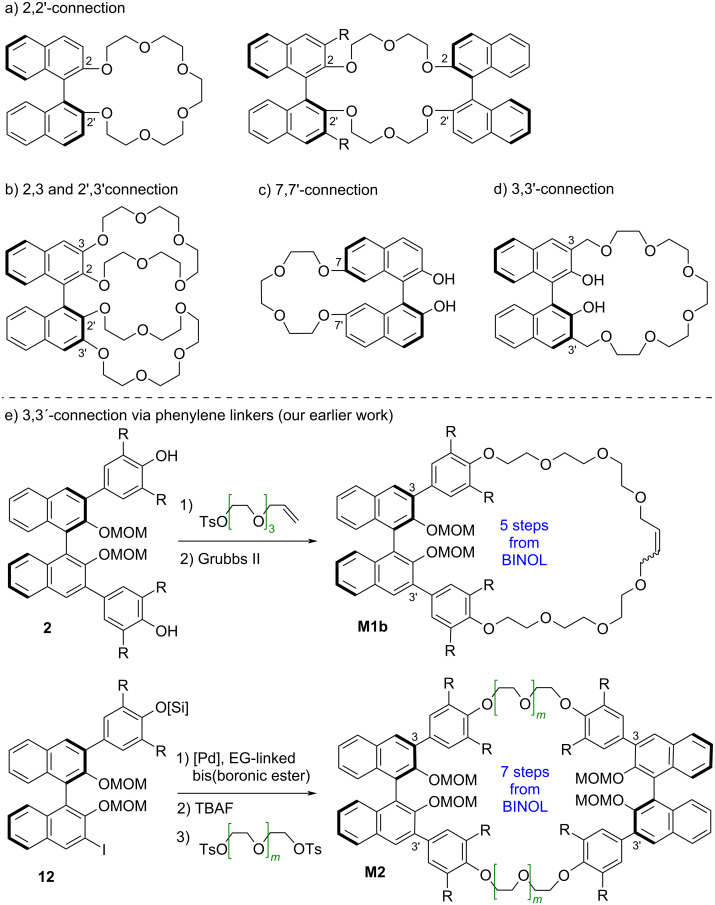
a–d) Selected structures of previously reported BINOL-based crown ether macrocycles; e) previous synthetic routes towards 3,3'-substituted BINOL crown ethers from our group.

In most BINOL-based crown ethers, the macrocycle is attached to the BINOL unit via the oxygens in the 2,2'-positions. This structural motif has been used to construct crown ethers featuring either one or two BINOL units (see Figure 1a) [16–23].

In a related approach, a single BINOL can be equipped with two crown ethers by attaching these via the 2,3 and 2',3'-positions, respectively (see Figure 1b) [36–37]. Less frequently, the 7,7'-positions (see Figure 1c) [38–40] or the 3,3'-positions (see Figure 1d) [41–43] have been used for attaching the crown ether macrocycle, although this strategy has the advantage that the 2,2'-hydroxy groups remain intact and can be used for further binding or functionalization. Our group recently became interested in synthesizing BINOL derivatives featuring macrocyclic crown ethers that are attached at the 3,3'-positions via additional phenylene spacers (see Figure 1e) [35,44–45]. Macrocycles **M1b** with a single BINOL unit were generated from the corresponding diol **2** by attachment of allylated linkers, followed by ring-closing metathesis [46–50]. For the synthesis of macrocycles **M2** with two BINOL units, we relied on the monoiodide **12**, which was first reacted in a two-fold Suzuki coupling to install the first linker, followed by silyl deprotection and introduction of the second linker via nucleophilic substitution [51]. Both procedures require multiple steps towards the desired macrocycles. The route towards the bis-BINOL macrocycles additionally requires the synthesis of the unsymmetric monoiodide **12**. In our previous work, the route starting from **12** had been designed to give access to macrocyclic and singly linked bis-BINOL derivatives from a single precursor, but this is unnecessary if only macrocyclic bis-BINOL derivatives are desired.

**Figure 2:**
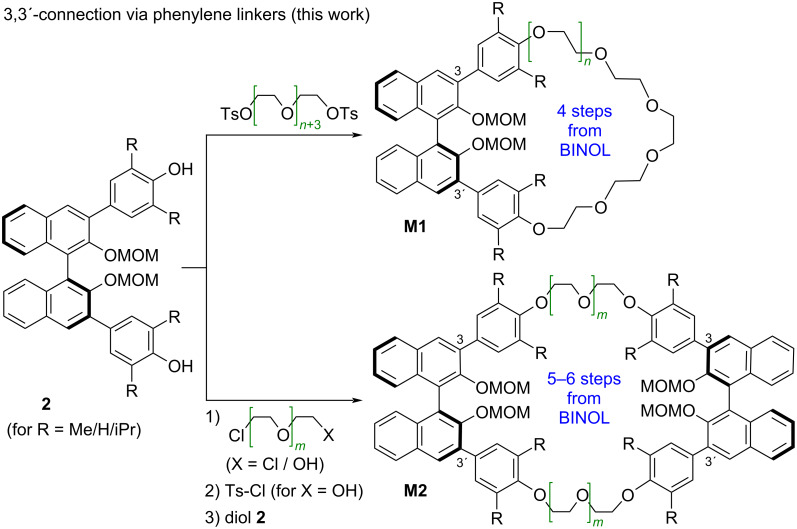
Optimized synthetic routes towards 3,3'-substituted BINOL crown ethers (this work).

For this reason, we sought to find optimized syntheses for such BINOL-based macrocycles (see Figure 2).

After investigating different synthetic routes (vide infra), we found that Williamson-type ether syntheses were the best-yielding approach towards the desired macrocycles. This route was then applied for the synthesis of macrocycles featuring one or two BINOL units, featuring differently substituted phenylene linkers and featuring ethylene glycol linkers of different lengths.

## Results and Discussion

Unless otherwise stated, all BINOL derivatives were used as the (*S*)-enantiomers and the stereochemistry will not be mentioned further.

### Synthesis of macrocycles featuring one BINOL unit

We first investigated the synthesis of crown ether-type macrocycles **M1** which feature a single BINOL unit. Our previous synthetic approaches (see Figure 1e) toward BINOL macrocycles had successfully used either Williamson-type reactions or Suzuki couplings for the synthesis of intermediates. Thus, we chose to compare the use of two-fold Suzuki coupling or two-fold Williamson reaction for the synthesis of macrocycles **M1**.

**Figure 3:**
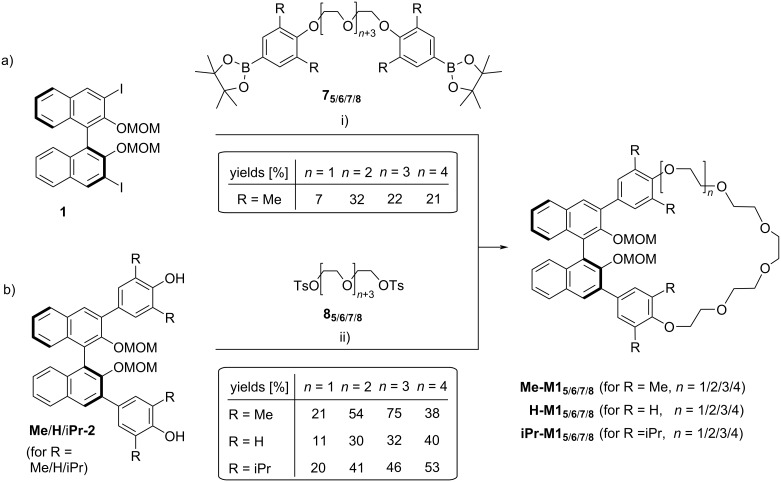
Synthetic routes towards macrocycles featuring one BINOL unit. a) Two-fold Suzuki coupling and b) two-fold Williamson synthesis. Reagents and conditions: i) bis(boronic ester) **7_5_**_/_**_6_**_/_**_7_**_/_**_8_** (1.0. equiv), Pd_2_(dba)_3_ (0.1 equiv), P(*o*-Tol)_3_ (0.2 equiv), *n*-Bu_4_N^+^OH^−^ (3.2. equiv), toluene/H_2_O 5:1, 90 °C; ii) ethylene glycol bistosylates **8_5_**_/_**_6_**_/_**_7_**_/_**_8_** (1.0 equiv), Cs_2_CO_3_ (2.0 equiv), CH_3_CN, 80 °C.

For the approach via Suzuki coupling, we employed the previously reported BINOL-diiodide **1** [52], which was reacted with bisboronic acids (see Figure 3a).

Here we chose bisboronic esters **7_5_**_/_**_6_**_/_**_7_**_/_**_8_** which feature dimethylphenyl groups that are linked via penta/hexa/hepta/octaethylene glycol chains (throughout this publication, the suffix denotes the number of ethylene glycol units in a single linker, for the structures of **7_5_**_/_**_6_**_/_**_7_**_/_**_8_**, see Figure 3a). The reactivity of **7_6_** had previously been established in the reaction with the unsymmetric monoiodide **12** (see Figure 1e), which proceeded in 59% yield [51]. However, under the same coupling conditions (Pd_2_(dba)_3_, P(*o*-Tol)_3_, *n*-Bu_4_N^+^OH^−^, toluene/H_2_O, 90 °C), the reaction of diiodide **1** with bisboronic acids **7_5_**_/_**_6_**_/_**_7_**_/_**_8_** gave only low yields of the desired macrocycles **Me-M1** (7/32/22/21% for **Me-M1_5_**_/_**_6_**_/_**_7_**_/_**_8_**). Thus, the macrocyclization via two-fold Suzuki coupling was not suitable in our hands.

Therefore, we turned our attention towards the two-fold Williamson reaction (see Figure 3b). First, we employed the tetramethyl-substituted diol **Me-2**, which gives access to the same macrocyles **Me-M1_5_**_/_**_6_**_/_**_7_**_/_**_8_** which we could only generate in low yields via two-fold Suzuki coupling (vide supra). To this end, **Me-2** was reacted with the bistosylated ethylene glycols **8_5_**_/_**_6_**_/_**_7_**_/_**_8_** in the presence of Cs_2_CO_3_ as a base (CH_3_CN, 80 °C). To our delight, we could isolate the macrocyles **Me-M1_5_**_/_**_6_**_/_**_7_**_/_**_8_** in significantly higher yields of 21/54/75/38%. Cesium carbonate was chosen as the base, because in initial experiments, we obtained consistently higher yields and fewer side-products in comparison to other bases (such as NEt_3_ or K_2_CO_3_), as reported in the literature for related macrocyclizations [53–55].

We then investigated the impact of different substituents on the phenylene linkers on the macrocycle formation. In comparison to the Me-derivative **Me-2**, both the unsubstituted diol **H-2** and the isopropyl derivative **iPr-2** gave generally lower yields for the smaller macrocycles (11/30/32% for **H-M1_5_**_/_**_6_**_/_**_7_**, 20/41/46% for **iPr-M1_5_**_/_**_6_**_/_**_7_**), while the yields for the largest macrocycles **H**/**iPr**-**M1_8_** were slightly increased (40% for **H-M1_8_**, 53% for **iPr-M1_8_**). As a general trend, we observed that the pentaethylene glycol linker seems to be too short to result in efficient macrocyclization (both in Suzuki and Williamson reactions), while the longer linkers give moderate to good yields of the desired macrocycles. In the Williamson approach, we find increasing yields in the series **H-M1_5_**_/_**_6_**_/_**_7_**_/_**_8_** and **iPr-M1_5_**_/_**_6_**_/_**_7_**_/_**_8_**, with the best yield obtained for the longest octaethylene glycol linkers in **H**/**iPr-M1_8_**. To our surprise, the Me-series not only gives generally better yields, but also shows the maximum yield for the shorter heptaethylene glycol macroycle **Me-M1_7_**. The synthesis of the octaethyleneglycol derivative **Me-M1_8_** was repeated several times but gave reproducibly lower yields than the shorter version **Me-M1_7_**.

**Figure 4:**
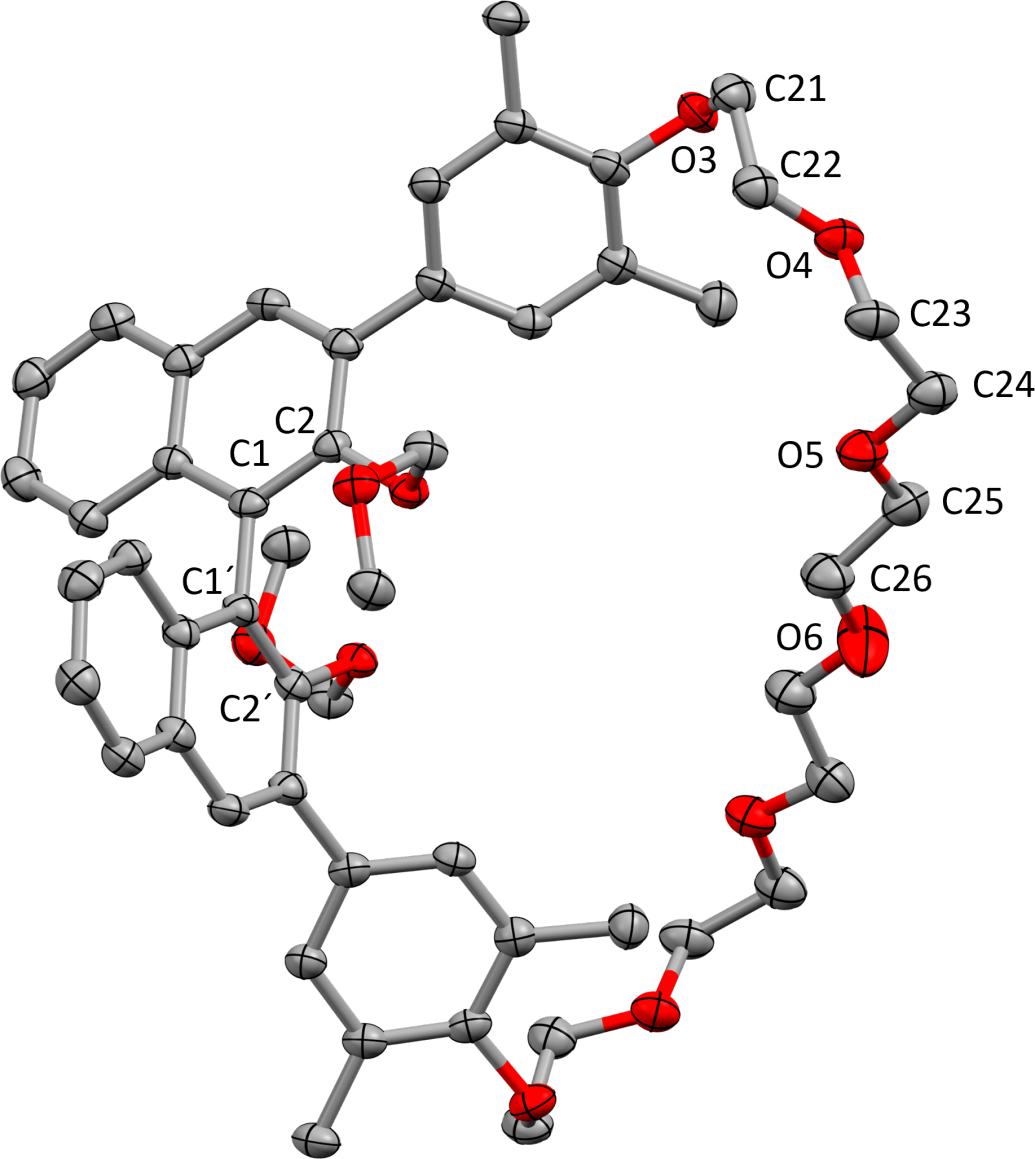
Molecular structure of macrocycle (*R*)-**Me-M1_6_** in the solid state (hydrogen atoms are omitted for clarity and thermal ellipsoids are set at the 60% probability level). The ethylene glycol chain is partially disordered, only one component is shown.

All macrocycles were fully characterized by standard analytical methods (see Supporting Information File 1). The structure of **Me-M1_6_** was additionally verified by single-crystal X-ray analysis (the enantiomeric compound (*R*)-**Me-M1_6_** resulting from a separate synthesis was crystallized, see Figure 4). Due to the macrocyclic structure, the two ethylene glycol units directly attached to each dimethylphenyl linker adapt a *gauche*-conformation (∠O3–C21–C22–O4 = 66.9(3)°, ∠O4–C23–C24–O5 = −77.3(2)°), and only the central ethylene glycol can be found in a *trans*-conformation (∠O5–C25–C26–O6 = −173.0(5)°). Probably induced by the linker, the commonly preferred perpendicular orientation of the naphthyl-units in the BINOL core is slightly distorted (∠C2–C1–C1'–C2' = −79.2(3)°).

In summary, we could obtain the desired macrocycles **M1** containing a single BINOL unit in satisfying yields (11–75% from diols **Me**/**H**/**iPr-2**). The previously published route based on ring-closing metathesis gave macrocycle **M1b** (see Figure 1e), which is a 38-membered macrocycle, in 38% yield. Our novel route gives access to 30/33/36/39-membered rings (**M1_5_**_/_**_6_**_/_**_7_**_/_**_8_**), thus complementing the previous approach. Although yields are not consistently better than with the previous route, the novel route has three key advantages: Firstly, the two-step synthesis of the allyl tosyl ethylene glycol (see Figure 1e) can be avoided and the bistosylated ethylene glycol **8** (available in one step) can be used instead. Secondly, the ring-closing metathesis was substituted for an operationally simple Williamson reaction. This results in macrocycles with regular ethylene glycol linkers and removes the internal double bond, which was previously generated as an *E*/*Z*-mixture from the ring-closing metathesis. Thirdly, this route also give access to the smaller macrocylces **M1_5_**_/_**_6_**, while similar ring sizes were difficult to obtain by ring-closing metathesis in our hands [56].

### Synthesis of macrocycles featuring two BINOL units

For the synthesis of macrocycles featuring two BINOL units, our first goal was the introduction of two hexaethylene glycol chains between the two BINOL units. These derivatives had proven to be highly efficient organocatalysts in our earlier work, based on the large conformational freedom that is given by the long, flexible linkers [51].

**Figure 5:**
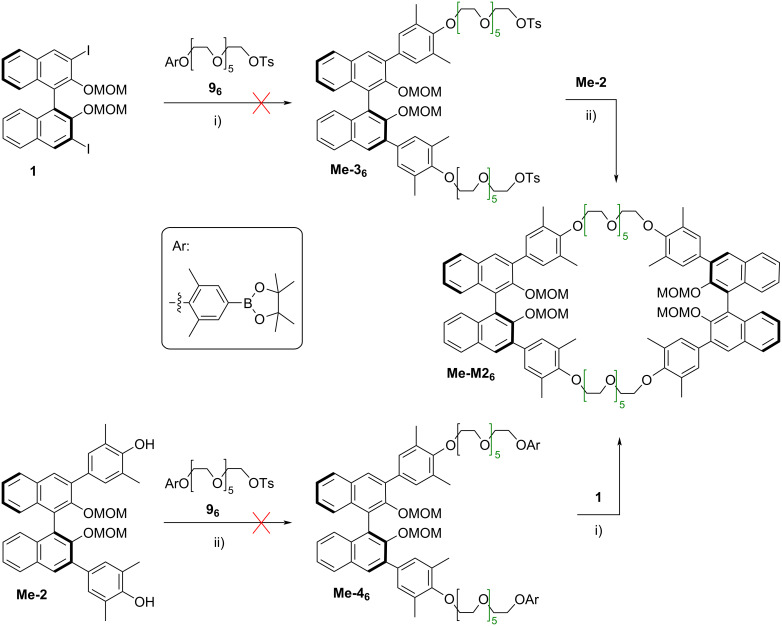
Initially attempted route towards bis-BINOL macrocycles based on precursors **Me-3_6_** or **Me-4_6_**. Conditions: i) **9_6_** (2.2 equiv), Pd_2_(dba)_3_ (0.1 equiv), P(*o*-Tol)_3_ (0.2 equiv), *n*-Bu_4_N^+^OH^−^ (3.2 equiv), toluene/H_2_O 5:1, 90 °C; ii) **9_6_** (2.2. equiv), Cs_2_CO_3_ (3.0 equiv), CH_3_CN, 80 °C.

To achieve a convergent synthesis, we first designed the hexaethylene glycol linker **9_6_**, which features a tosylate leaving group at one end and a phenylboronic ester at the other end (see Figure 5).

This would allow a two-step synthesis of the desired macrocycles by performing a sequence of two-fold Suzuki coupling, followed by two-fold Williamson synthesis or vice versa. However, attempts to realize the first step of either sequence, i.e., reaction of the precursor **9_6_** with either the BINOL diiodide **1** (in a two-fold Suzuki coupling) or with the BINOL-derived diol **Me-2** (in a two-fold Williamson reaction) gave no meaningful yields of the desired intermediates **Me-3_6_** or **Me-4_6_**, respectively.

**Figure 6:**
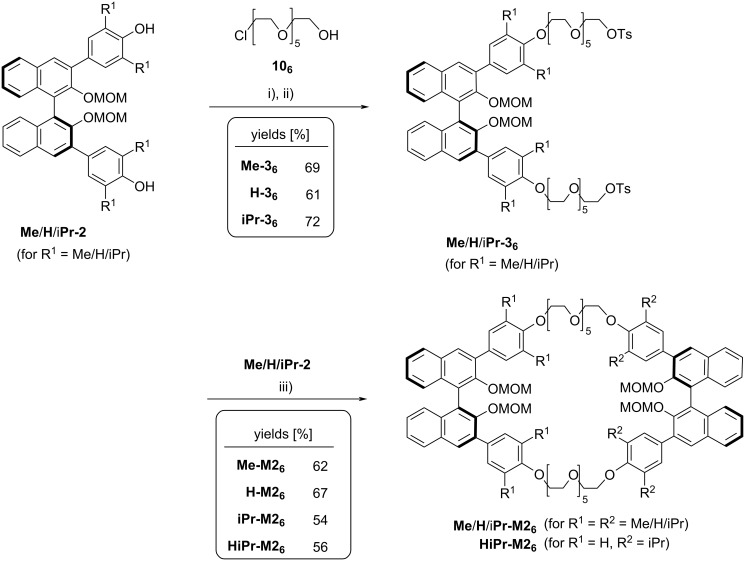
Synthetic route towards macrocycles featuring two BINOL units linked via hexaethylene glycol spacers. Conditions: i) chloroalcohol **10_6_** (2.2 equiv), Cs_2_CO_3_ (2.2 equiv), CH_3_CN, 80 °C; ii) tosyl chloride (2.5 equiv), triethylamine (4.0 equiv), DMAP (0.4 equiv), CH_2_Cl_2_, 25 °C (69/61/72% yield over two steps for **Me**/**H**/**iPr-3_6_**); iii) **Me**/**H**/**iPr-2** (1.0 equiv), Cs_2_CO_3_ (3.2 equiv), CH_3_CN, 80 °C (62/67/54/56% yield for **Me**/**H**/**iPr**/**HiPr-M2_6_**).

For this reason, we resorted to an alternative synthesis of the BINOL-based bistosylates **Me**/**H**/**iPr-3_6_**. For the long hexaethylene glycol linker, a direct reaction of the diols **Me**/**H**/**iPr-2** with a hexaethylene glycol bistosylate did not seem feasible, since this would lead to the mono-BINOL macrocycles **Me**/**H**/**iPr-M1_6_** even in the presence of a large excess of bistosylate. Thus, we first reacted diols **Me**/**H**/**iPr-2** with chloroalcohol **10_6_** in the presence of Cs_2_CO_3_ (CH_3_CN, 80 °C), which gave the desired bisglycolated products **Me**/**H**/**iPr-5** (Supporting Information File 1). Subsequent reaction with tosyl chloride in the presence of triethylamine and DMAP (CH_2_Cl_2_, 25 °C) gave the desired BINOL bistosylates **Me**/**H**/**iPr-3_6_** in good yields (69/61/72% over two steps, see Figure 6). However, only if temperature and reaction times in this step were carefully controlled, the reaction proceeded cleanly. Deviations from the optimized conditions (see Supporting Information File 1 for details) resulted in greatly diminished yields due to the formation of various by-products.

Starting from the bistosylates **Me**/**H**/**iPr-3_6_**, reaction with the diols **Me**/**H**/**iPr-2** in the presence of Cs_2_CO_3_ as base (CH_3_CN, 80 °C) proceeded cleanly to give the desired hexaethylene glycol-linked bis-BINOL macrocycles that feature a 66-membered ring structure. Here, we successfully generated the *C*_2_-symmetric macrocycles **Me**/**H**/**iPr-M2_6_** (obtained in 62/67/54% yield) and the unsymmetrically substituted, *C*_1_-symmetric derivative **HiPr-M2_6_** (56% yield from **iPr-3_6_** and **H-2**). Thus, the overall yields for the large macrocycles **Me**/**H**/**iPr**/**HiPr-M2_6_** range from 15 to 17% (6 steps from BINOL, 35–42% over 3 steps from diols **Me**/**H**/**iPr-2**). This is an improvement in comparison to the yield obtained with the previous method, based on sequential introduction of both hexaethylene glycol linkers (see Figure 1b), which gave 11% yield for compound **Me-M2_6_** over 7 steps from BINOL.

**Figure 7:**
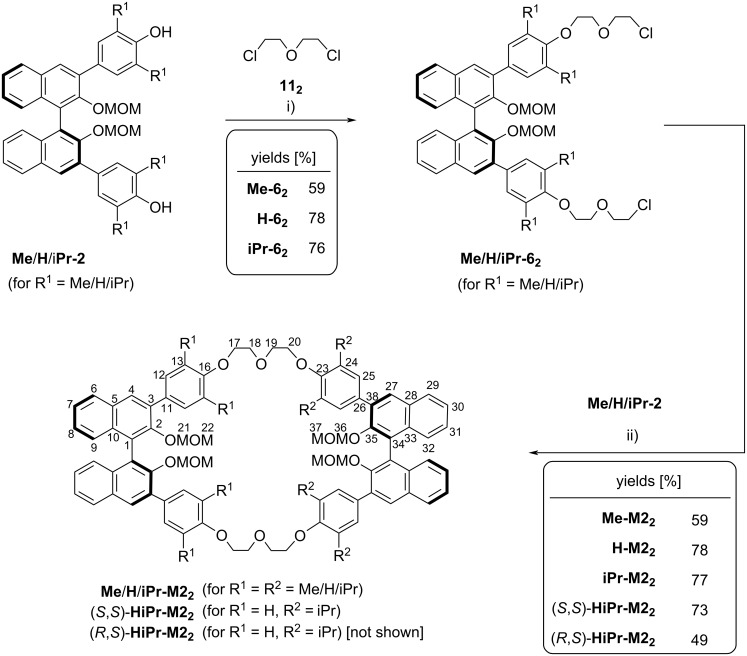
Synthetic route towards macrocycles featuring two BINOL units linked via diethylene glycol spacers. Conditions: i) dichloride **11_2_** (2.5 equiv), Cs_2_CO_3_ (2.5 equiv), CH_3_CN, 80 °C (59/78/76% yield for **Me**/**H**/**iPr-6_2_**; ii) **Me**/(*S*)-**H**/(*R*)-**H**/**iPr-2** (1.0 equiv), Cs_2_CO_3_ (3.2 equiv), CH_3_CN, 80 °C.

As a second synthetic aim, we wanted to realize the synthesis of smaller bis-BINOL macrocycles that feature two diethylene glycol linkers, thus yielding 42-membered macrocylces. In this case, it was possible to directly react diols **Me**/**H**/**iPr-2** with the corresponding dichloride **11_2_** (Cs_2_CO_3,_ CH_3_CN, 80 °C, see Figure 7), since this is too short to result in a mono-BINOL macrocycle.

The corresponding BINOL-based dichlorides **Me**/**H**/**iPr-6_2_** could be obtained in good yields (59/78/76%) and further reacted with the diols **Me**/**H**/**iPr-2** in the presence of Cs_2_CO_3_ (CH_3_CN, 80 °C). This cyclization yielded the symmetrically tetrasubstitued macrocycles **Me**/**H**/**iPr-M2_2_** in good yields of 59/78/77%. Again, we applied this protocol for the synthesis of the unsymmetric derivative **HiPr-M2_2_**, this time in two diastereomeric forms. In the unsymmetric case, the introduction of two BINOL units with opposite configuration does not furnish a *meso*-compound, so that we reacted either (*S*)- or (*R*)-**H-2** with the dichloride (*S*)-**iPr-6_2_** to give the diastereomeric macrocycles (*S*,S)-**HiPr-M2_2_** and (*R*,*S*)-**HiPr-M2_2_** in 73/49% yield, respectively.

**Figure 8:**
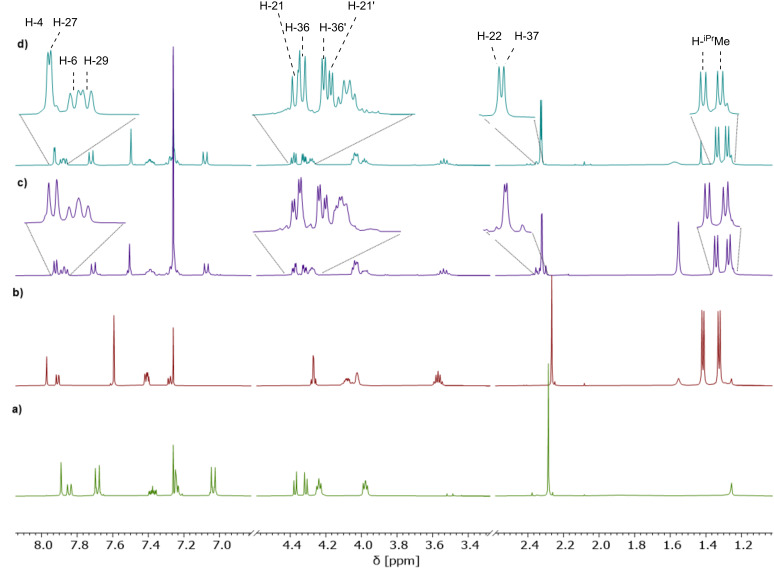
^1^H NMR spectra of a) (*S*,*S*)-**H-M2_2_**, b) (*S*,*S*)-**iPr-M2_2_**, c) (*S*,*S*)-**HiPr-M2_2_**, and d) (*R*,*S*)-**HiPr-M2_2_** (all: 400 MHz, CDCl_3_, 298 K, for labelling see Figure 7).

The ^1^H NMR spectra of the *C*_2_-symmetric derivatives (*S*,*S*)-**H**/**iPr-M2_2_** (see Figure 8a/b) differ most significantly in the splitting of the signals corresponding to the MOM-methylene protons near 4.3 ppm (H-21), which are clearly split into two doublets for (*S*,*S*)-**H-M2_2_**, but resemble more of a second order signal with a very small coupling constant in (*S*,*S*)-**iPr-M2_2_**. As expected, the two BINOL units in the symmetric macrocycles only give one set of signals, e.g., one singlet for H-4 and one doublet for H-6. When comparing these to the *C*_1_-symmetric compound (*S*,*S*)-**HiPr-M2_2_** (see Figure 8a–c), separate signals for the 4- and 6-positions of each BINOL unit can be observed (i.e., H-4/H-27 and H-6/H-29). In contrast, the signals of the MOM-methylene protons appear closer to the shift observed for (*S*,*S*)-**H-M2_2_**, however, also two differentiable sets for either BINOL unit can be observed. The MOM-CH_2_ signals of the diisopropylphenyl-substituted BINOL unit (i.e., H-21/H-21’) show a smaller splitting between the diastereomeric protons than the analogous MOM-CH_2_ protons (H-36/H-36’) of the phenyl-substituted BINOL unit, in line with the observations for the *C*_2_-symmetric compounds. The two diastereomeric macrocycles (*S*,*S*)- and (*R*,*S*)-**HiPr-M2_2_** (compare Figure 8c,d) show distinct, but small differences in the ^1^H chemical shifts. This indicates that despite the short diethylene glycol linkers, the mutual influence of both BINOL units on each other is small in the bis-BINOL macrocycle.

## Conclusion

In summary, we have developed novel approaches towards the synthesis of crown ethers that contain one or two BINOL units. The ethylene glycol units were attached to the BINOL backbone via differently substituted phenylene linkers, either featuring two methyl groups, two isopropyl groups, or no additional substituent.

First, we could show that the corresponding diols **Me**/**H**/**iPr-2** can be transformed into the corresponding mono-BINOL macrocycles via two-fold Williamson reaction with ethylene glycol bistosylates. Using penta-/hexa-/hepta-/octaethylene glycol bistosylates **8_5_**_/_**_6_**_/_**_7_**_/_**_8_**, the corresponding 30/33/36/39-membered macrocycles could be synthesized from all three diols **Me**/**H**/**iPr-2**, yielding a library of 12 different mono-BINOL macrocycles. Yields for the macrocyclization step depended strongly on ring size and substitution pattern and ranged from 11–74%.

Second, we could use **Me**/**H**/**iPr-2** as starting materials for bis-BINOL macrocycles. Attachment of ethylene glycol chains with suitable leaving groups (tosylate or chloride), followed by macrocyclization with a second equivalent of diols **Me**/**H**/**iPr-2** gave access to 4 different hexaethylene glycol-based macrocycles **M2_6_** (66-membered rings) and 5 different diethylene glycol-based macrocycles **M2_2_** (42-membered rings). Here, the yields for the macrocyclization were consistently high and ranged from 49–78%. In this fashion, we could not only generate the symmetrically tetrasubstituted macrocycles with either hexaethylene glycol linkers (**Me**/**H**/**iPr-M2_6_**) or diethylene glycol linkers (**Me**/**H**/**iPr-M2_2_**), but also the unsymmetrically substituted macrocycles **HiPr-M2_6_** and **HiPr-M2_2_**. Furthermore, the latter compound was generated in both diastereomeric forms, namely (*S*,*S*)- and (*R*,*S*)-**HiPr-M2_2_**.

We believe that these systems are highly promising candidates for further application in enantioselective chemosensing or organocatalysis, e.g., after transformation into the corresponding BINOL phosphoric acids. However, at this point, the application of BINOL-based crown ethers with 3,3'-appended ethylene glycol chains remains underdeveloped, partially due to the lack of simple and high-yielding synthetic routes. Thus, we believe that our newly developed optimized synthetic strategy will enable further applications of these BINOL-based macrocycles.

## Supporting Information

Synthetic procedures and NMR spectra for all new compounds, as well as the crystal structure data for **Me-M1_6_**. CCDC-2427523 contains the supplementary crystallographic data for this paper. This data can be obtained free of charge from The Cambridge Crystallographic Data Centre via https://www.ccdc.cam.ac.uk/data_request/cif.

File 1 Experimental procedures and characterization data of new compounds.

File 2 Crystallographic Information File (CIF) for the solid-state structure of (*R*)-**Me-M1_6_**.

## Data Availability

All data that supports the findings of this study is available in the published article and/or the supporting information of this article.
