# Supplementary material for: 3,3'-Linked BINOL macrocycles: optimized synthesis of crown ethers featuring one or two BINOL units
Source: Beilstein J Org Chem. 2025 Aug 28;21:1719–29. doi: 10.3762/bjoc.21.134 (PMC12415923; doi:10.3762/bjoc.21.134)
Supplement: File 1 — Experimental procedures and characterization data of new compounds. [file Beilstein_J_Org_Chem-21-1719-s001.pdf]

BEILSTEIN JOURNAL OF ORGANIC CHEMISTRY

Supporting Information

for

3,3'-Linked BINOL macrocycles: optimized synthesis of crown ethers featuring one or two BINOL units

Somayyeh Kheirjou, Jan Riebe, Maike Thiele, Christoph Wölper and Jochen Niemeyer

Beilstein J. Org. Chem. **2025,** *21,* 1719–1729. doi:10.3762/bjoc.21.134

Experimental procedures and characterization data of new compounds

License and Terms: This is a supporting information file under the terms of the Creative Commons Attribution License (https://creativecommons.org/licenses/by/4.0). Please note that the reuse, redistribution and reproduction in particular requires that the author(s) and source are credited and that individual graphics may be subject to special legal provisions.
The license is subject to the *Beilstein Journal of Organic Chemistry* terms and conditions: (https://www.beilstein-journals.org/bjoc/terms)

Contents

1. General information ... S2
1.1. Analytical methods ... S2
1.2. Materials and methods ... S2
1.2.1. Solvents ... S2
1.2.2. Chemicals ... S2
1.2.3. Materials ... S3
2. Synthetic procedures ... S3
2.1. Overview ... S3
2.2. Precursors ... S4
2.2.1. Synthesis of compounds **Me/H/iPr-3**$_6$ ... S4
2.2.2. Synthesis of compound **Me/H/**iPr-6$_2$ ... S14
2.2.3. Synthesis of compounds 7$_{5,6,7,8}$ ... S21
2.2.4. Synthesis of compound **Me/H/iPr-9**$_6$ ... S28
2.3. General procedure for the synthesis of macrocycles ... S35
Synthetic routes towards macrocycles featuring one BINOL unit (**Me/H/iPr-M1**$_{5/6/7/8}$) ... S35
2.3.1. Two-fold Suzuki coupling **(General procedure A)** ... S35
2.3.2. Two-fold Williamson synthesis **(General procedure B)** ... S35
Synthetic routes towards macrocycles featuring two BINOL units ... S35
2.3.3. Macrocycles **M2**$_6$ **(General procedure C)** ... S35
2.3.4. Macrocycles **M2**$_2$ **(General procedure D)** ... S35
2.4. Mono-BINOL macrocycles ... S36
2.5. Bis-BINOL macrocycles ... S62
3. Crystal structure of **Me-M1**$_6$ ... S83
4. References ... S88

1. General information

1.1. Analytical methods

NMR spectra were recorded on a Bruker Avance NEO 400 spectrometer (^{1}H: 400 MHz, ^{13}C: 101 MHz, ^{19}F: 376 MHz, ^{31}P: 162 MHz) or DRX 600 spectrometer (^{1}H: 600 MHz, ^{13}C: 151 MHz, ^{31}P: 243 MHz). All NMR experiments were performed at room temperature. The residual proton signals of the deuterated solvents were used to reference the ^{1}H NMR spectra. The chemical shifts of the residual proton signals of the solvents in the ^{1}H NMR are: $CDCl_3$: δ = 7.26 ppm, d_6-DMSO: δ = 2.50 ppm. The solvent signals were used to reference the ^{13}C NMR spectra. The chemical shifts of the signals of the solvents in the ^{13}C NMR are: $CDCl_3$ δ = 77.16 ppm, d_6-DMSO δ = 39.52 ppm. The apparent coupling constants are given in hertz. The description of the fine structure means: s = singlet, bs = broad singlet, d = doublet, t = triplet, m = multiplet, hept = heptet.

Low-resolution ESI mass spectra were recorded on a Bruker Amazon SL spectrometer. High-resolution ESI mass spectra were recorded on a Bruker Maxis 4G spectrometer.

All IR spectra were measured on a Jasco FT/IR-430 spectrometer. The data was analysed using the supplementary software.

1.2. Materials and methods

All reactions that needed exclusion of residual air or humidity were performed under an argon inert gas atmosphere using common Schlenk techniques. All commercially purchased chemicals were not purified before use.

1.2.1. Solvents

Solvents for synthetic procedures were analytically pure, solvents for aqueous extraction processes or flash column chromatographies were of technical grade. Technical grade ethyl acetate and cyclohexane were always distilled before being used for work-ups or columns. Anhydrous tetrahydrofuran (THF) and dichloromethane (DCM) were obtained via an MBRAUN SPS-800 solvent purification system. Acetonitrile and toluene/water were degassed by bubbling with argon for 15 minutes before use. Glacial acetic acid was purchased from VWR without further purification. 1,4-dioxane, acetonitrile, ethanol, toluene, ethyl acetate, cyclohexane, acetone, dichloromethane were purchased from VWR.

1.2.2. Chemicals

MOM-Cl, *p*-toluenesulfonyl chloride, $Pd(PPh_3)_4$, $Pd(dppf)Cl_2$, tris(dibenzylidene acetone)dipalladium(0), 2,6-diisopropylphenol, 2,6-dimethylphenol, tri(*o*-tolyl)phosphine and tetrabutylammonium hydroxide 30-hydrate were purchased from Sigma-Aldrich. 4-Hydroxy-phenylboronic acid and iodine were purchased from TCI. Bis(pinacolato)diboron and cesium carbonate were purchased from Carbolution Chemicals. Pentaethylene glycol was purchased from Alfa Aesar, hexaethylene glycol and heptaethylene glycol were purchased from BLDpharm. Octaethylene glycol and 4-(dimethylamino)pyridine were purchased from Fluorochem. Bis(chloroethyl)ether was purchased from Thermo Scientific. (*R*)-1,1'-Binaphthyl-2,2'-diol (>99.9% ee) was purchased from RCA Separations and used without further purification. *n*-Butyllithium (2.7 M in toluene) was purchased from Thermo Scientific. Ammonium chloride, sodium chloride, sodium bicarbonate, triethyl amine and sodium sulfate were purchased from VWR.

1.2.3. Materials

For thin-layer chromatography (TLC) analysis throughout this work, Polygram® SIL G/UV254 TLC plates (silica gel 0.2 mm, 40 × 80 mm) were used. Visualization of the spots was carried under a 254 nm UV light source and, if necessary, stained by potassium permanganate and heated with a heat gun. The products were purified by flash column chromatography on silica gel 60M (40–63 µm) which was purchased from MACHEREY-NAGEL GmbH & Co. KG.

2. Synthetic procedures

2.1. Overview

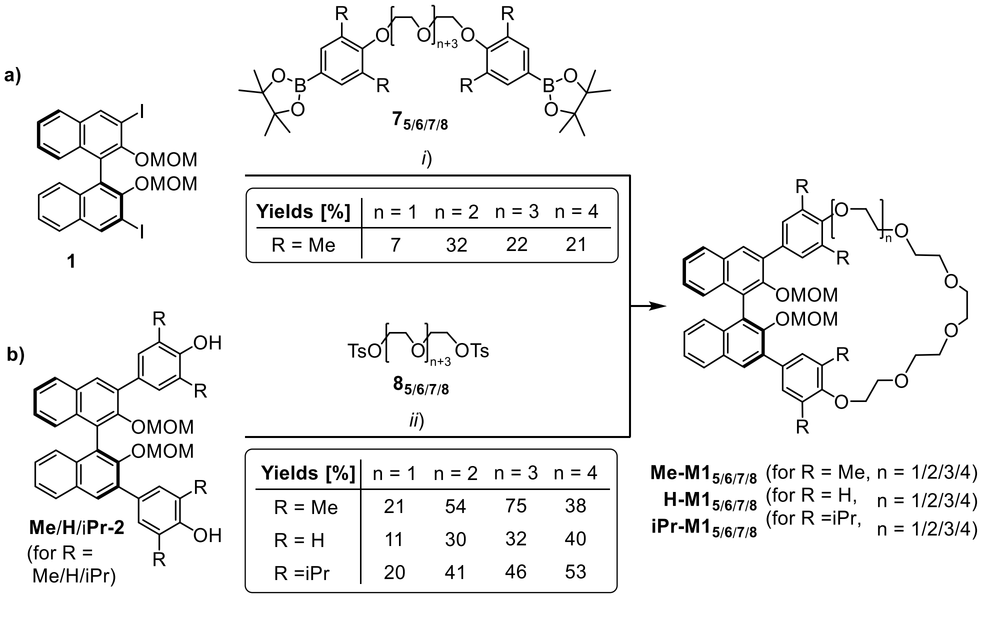

Yields [%]	n = 1	n = 2	n = 3	n = 4
R = Me	7	32	22	21

Yields [%]	n = 1	n = 2	n = 3	n = 4
R = Me	21	54	75	38
R = H	11	30	32	40
R =iPr	20	41	46	53

Figure S1: Synthetic routes towards macrocycles featuring one BINOL unit. a) Two-fold Suzuki coupling; b) Two-fold Williamson synthesis. Reagents and conditions: *i*) bis(boronic ester) $\mathbf{7}_{5/6/7/8}$ (1.0. equiv), $Pd_2(dba)_3$ (0.1 equiv), P(*o*-Tol)$_3$ (0.2 equiv), *n*-$Bu_4N^+OH^-$ (3.2. equiv), toluene/H_20 5:1, 90 °C, ii) ethylene glycol bistosylates $\mathbf{8}_{5/6/7/8}$ (1 equiv), Cs_2CO_3 (2.0 equiv), CH_3CN, 80 °C.

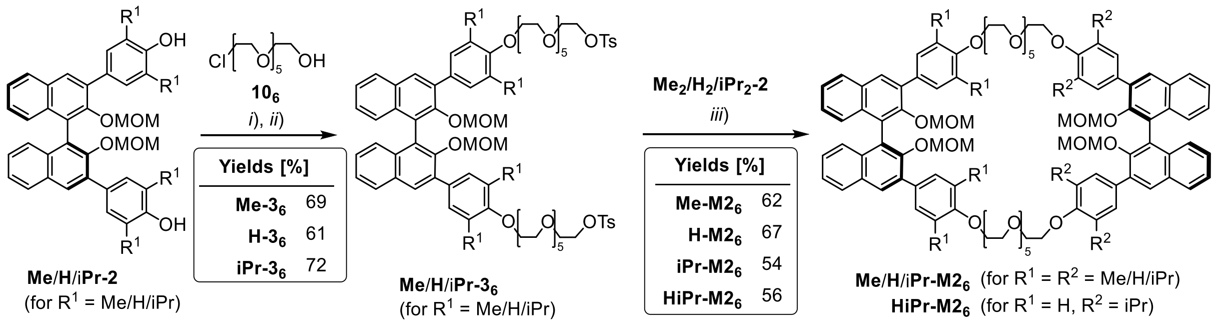

Figure S2: Synthetic route towards macrocycles featuring two BINOL-units linked via hexaethylene glycol spacers. i) chloroalcohol **10**$_6$ (2.2 equiv), Cs_2CO_3 (3.2 equiv), CH_3CN, 85 °C, ii) tosyl chloride (2.3 equiv), triethylamine (4.0 equiv), 4-(dimethylamino)pyridine (0.4 equiv), CH_2Cl_2, 25 °C (69%/61%/72% yield over two steps for **Me/H/iPr-3**$_6$), iii) **Me/H/iPr-2** (1.0 equiv), Cs_2CO_3 (3.2 equiv), CH_3CN, 85 °C (62%/67%/54%/56% yield for **Me/H/iPr/HiPr-M2**$_6$).

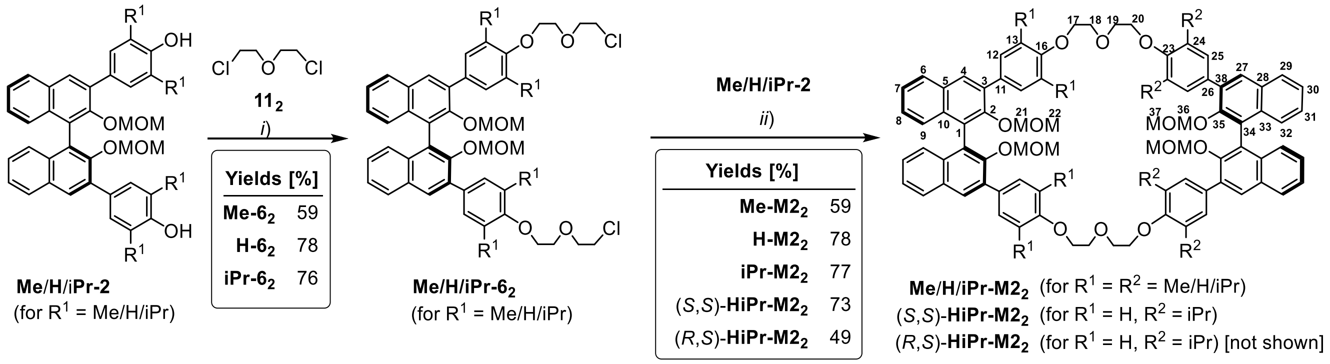

Figure S3: Synthetic route towards macrocycles featuring two BINOL-units linked via diethylene glycol spacers. *i*) dichloride **11**$_2$ (2.5 equiv), Cs_2CO_3 (2.5 equiv), CH_3CN, 80 °C (59%/78%/76% yield for **Me/H/iPr-6**$_2$), *ii*) **Me/**(*S*)-**H/**(*R*)-**H/-2** (1 equiv), Cs_2CO_3 (3.2 equiv), CH_3CN, 80 °C.

2.2. Precursors

Chloroalcohol **10**$_6$ and oligo-ethylene glycol bistosylates **8**$_{5,6,7,8}$ were prepared according to literature procedures.[1, 3]

2.2.1. Synthesis of compounds **Me/H/iPr-3**$_6$

Compound **Me/H/iPr-2** (1 equiv) and cesium carbonate (2.2 equiv), were dissolved in degassed acetonitrile and stirred for 15 minutes. After the addition of chloroalcohol **10**$_6$ (2.2 equiv) , the reaction mixture was stirred overnight under reflux. After cooling to room temperature, cesium carbonate was removed by filtration and all volatiles were removed in *vacuo* yielding compound **Me/H/iPr-5**$_6$**.** The crude product was used in the next step without further purification[2].

Compound **Me/H/iPr-5**$_6$ (1 equiv), triethylamine (4.1 equiv) and 4-(dimethylamino)pyridine (0.4 equiv) were mixed in CH_2Cl_2 and cooled to 0 °C using an ice bath. 4-Toluenesulfonyl chloride (2.5 equiv) was added at once to the reaction mixture with vigorous stirring. After keeping at 0–5 °C for 5 hours, the ice bath was removed[3]. The reaction mixture was stirred at room temperature overnight. The solution was washed with 0.1 N HCl (twice (20 mL/0.03 mmol of **Me/H/iPr-5**$_6$)), and brine (twice (20 mL/0.03 mmol of **Me/H/iPr-5**$_6$)). The organic layer was dried by Na_2SO_4, filtered and concentrated by evaporation. The crude product was purified by column chromatography.

Compound **Me-3$_6$**

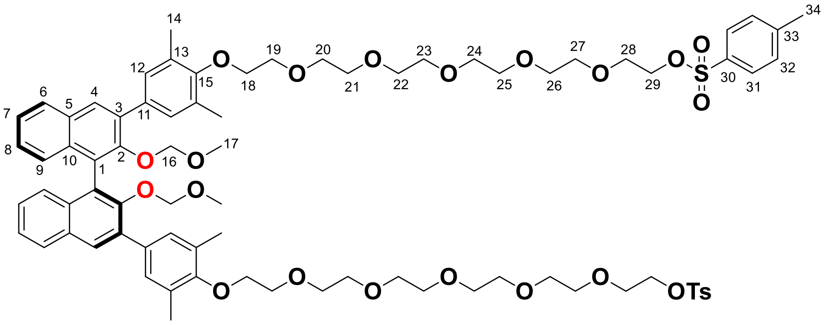

As described above, compound **Me-5$_6$** was synthesized using **Me-2** (22.7 mg, 0.0369 mmol, 1 equiv), cesium carbonate (26.5 mg, 0.0813 mmol, 2.2 equiv) and chloroalcohol **10$_6$** (26.2 mg, 0.0811 mmol, 2.2 equiv) in degassed acetonitrile (2 mL) as a colorless liquid and used without purification in the next step. Compound **Me-5$_6$** (23.0 mg, 0.0201 mmol, 1 equiv), *p*-toluenesulfonyl chloride (8.98 mg, 0.0471 mmol, 2.5 equiv), triethylamine (11.0 µL, 7.99 mg, 0.0789 mmol, 4.1 equiv), 4-(dimethylamino)pyridine (0.94 mg, 0.0077 mmol, 0.4 equiv) were reacted in dichloromethane (340 µL). After purification by column chromatography (cyclohexane/ethyl acetate 1:2) the product was obtained as a colorless wax (19.1 mg, 0.0132 mmol, 68.5%).

$C_{78}H_{98}O_{22}S_2$: 1451.74 g/mol.

^{1}H-NMR (400 MHz, [D$_6$]-dimethyl sulfoxide, 298 K) δ [in ppm] = 8.04 (s, 2H, H-4), 8.01 (d, *J* = 8.0 Hz, 2H, H-6), 7.77 (d, *J* = 8.4 Hz, 4H, H-31), 7.46 (d, *J* = 8.3 Hz, 4H, H-32), 7.47-7.43 (m, 2H, H-7), 7.34 (s, 4H, H-12), 7.31 (dd, *J* = 8.3 Hz, *J* = 1.4 Hz, 2H, H-8), 7.06 (d, *J* = 8.2 Hz, 2H, H-9), 4.38 (d, *J* = 5.4 Hz, 2H, H-$16_{1/2}$), 4.28 (d, *J* = 5.4 Hz, 2H, H-$16_{1/2}$), 4.09 (t, *J* = 4.3 Hz, 4H, H-29), 3.94-3.91 (m, 4H, H-18), 3.75-3.73 (m, 4H, H-19), 3.63-3.60 (m, 4H, H-28), 3.58-3.47 (m, 24H, H-27, H-26, H-25, H-22, H-21, H-20), 3.43 (br s, 8H, H-23, H-24), 2.40 (s, 6H, H-34), 2.32 (s, 6H, H-17), 2.30 (s, 12H, H-14).

^{13}C-NMR (101 MHz, [D$_6$]-dimethyl sulfoxide, 298 K) δ [in ppm] = 154.99 (C-15), 150.49 (C-2), 144.86 (C-33), 134.71 (C-3), 133.70 (C-11), 132.80 (C-10), 132.40 (C-30), 130.65 (C-13), 130.44 (C-5), 130.38 (C-1), 130.19 (C-32), 130.11 (C-4), 129.52 (C-12), 128.03 (C-6), 127.61 (C-31), 126.34 (C-8), 125.57 (C-9), 125.13 (C-7), 97.56 (C-16), 71.44 (C-18), 69.86 (C-19), 67.86 (C-28), 69.97, 69.96, 69.85, 69.80, 69.79, 69.77,69.69, 69.64 (C-29, 28, 27, 26, 25, 24, 23, 22, 21, 20)*, 55.25 (C-17), 21.06 (C-34), 16.06 (C-14).

*Partially overlapping signals.

^{1}H,^{1}H-COSY (400 MHz / 400 MHz, [D$_6$]-dimethyl sulfoxide, 298 K) δ [in ppm] = 8.01/7.47-7.43 (H-6/H-7), 7.77/7.46 (H-31/H-32), 7.46/7.77 (H-32/H-31), 7.47-7.43/7.77, 7.31 (H-7/H-6, H-8), 7.34/2.30 (H-12/H-14), 7.31/7.47-7.43, 7.06 (H-8/H-7, 9), 7.06/7.31 (H-9/H-8), 4.38/4.28 (H-$16_{1/2}$/ H-$16_{1/2}$), 4.28/4.38 (H-$16_{1/2}$/ H-$16_{1/2}$), 4.09/3.63-3.60 (H-29/H-28), 3.94-3.91/3.75-3.73 (H-18/H-19), 3.75-3.73/3.94-3.91 (H-19/H-18), 3.63-3.60 /4.09 (H-28/H-29), 2.30/7.34 (H-14/H-12).

^{1}H,^{13}C-GHSQC (400 MHz / 101 MHz, [D$_6$]-dimethyl sulfoxide, 298 K) δ (^{1}H) / δ (^{13}C) [in ppm] = 8.04/130.11 (H-4/C-4), 8.01/128.03 (H-6/C-6), 7.77/127.61 (H-31/C-31), 7.46/130.19 (H-32/C-32), 7.47-7.43/125.13 (H-7/C-7), 7.34/129.52 (H-12/C-12), 7.31/126.34 (H-8/C-8), 7.06/125.57 (H-9/C-9), 4.38/97.56 (H-$16_{1/2}$/C-16), 4.28/97.56 (H-$16_{1/2}$/C-16), 3.94-3.91/71.44 (H-18/C-18), 3.75-3.73/69.86 (H-19/ C-19), 3.63-3.60/67.86 (H-28/C-28), 2.40/21.06 (H-34/C-34), 2.32/55.25 (H-17/C-17), 2.30/16.06 (H-14/C-14).

^{1}H,^{13}C-GHMBC (400 MHz / 101 MHz, [D$_6$]-dimethyl sulfoxide, 298 K) δ (^{1}H) / δ (^{13}C) [in ppm] = 8.04/150.49, 133.70, 132.80, 128.03 (H-4/C-2, 11, 10, 6), 8.01/132.80, 130.11, 126.34 (H-6/ C-10, 4, 8), 7.77/144.86 (H-31/C-33), 7.46/132.40, 21.06 (H-32/ C-30, 34), 7.47-7.43/130.44, 125.57 (H-7/ C-5, 9),

7.34/154.99, 134.71, 16.06 (H-12/C-15, 3, 14), 7.31/132.80, 128.03 (H-8/ C-10, 6), 7.06/130.44, 130.38, 125.13 (H-9/C-5, 1, 7), 4.38/150.49, 55.25 (H-$16_{1/2}$/ C-2, 17), 4.28/150.49, 55.25 (H-$16_{1/2}$/ C-2, 17), 2.40/144.86, 130.19 (H-34/ C-33, 32), 2.32/97.56 (H-17/ C-16), 2.30/154.99, 129.52 (H-14/C-15, 12).

MS (ESI-pos, MeOH): *m/z* = 748.2889 ($[M+2Na]^{2+}$, calcd. 748.2888 $[C_{78}H_{98}O_{22}S_2Na_2]^{2+}$).

IR (ATR-FT): $\tilde{\nu}$ (cm^{-1}) = 702, 749, 764, 897, 1099, 1263, 1275, 1457, 2885, 2921, 3005, 3054.

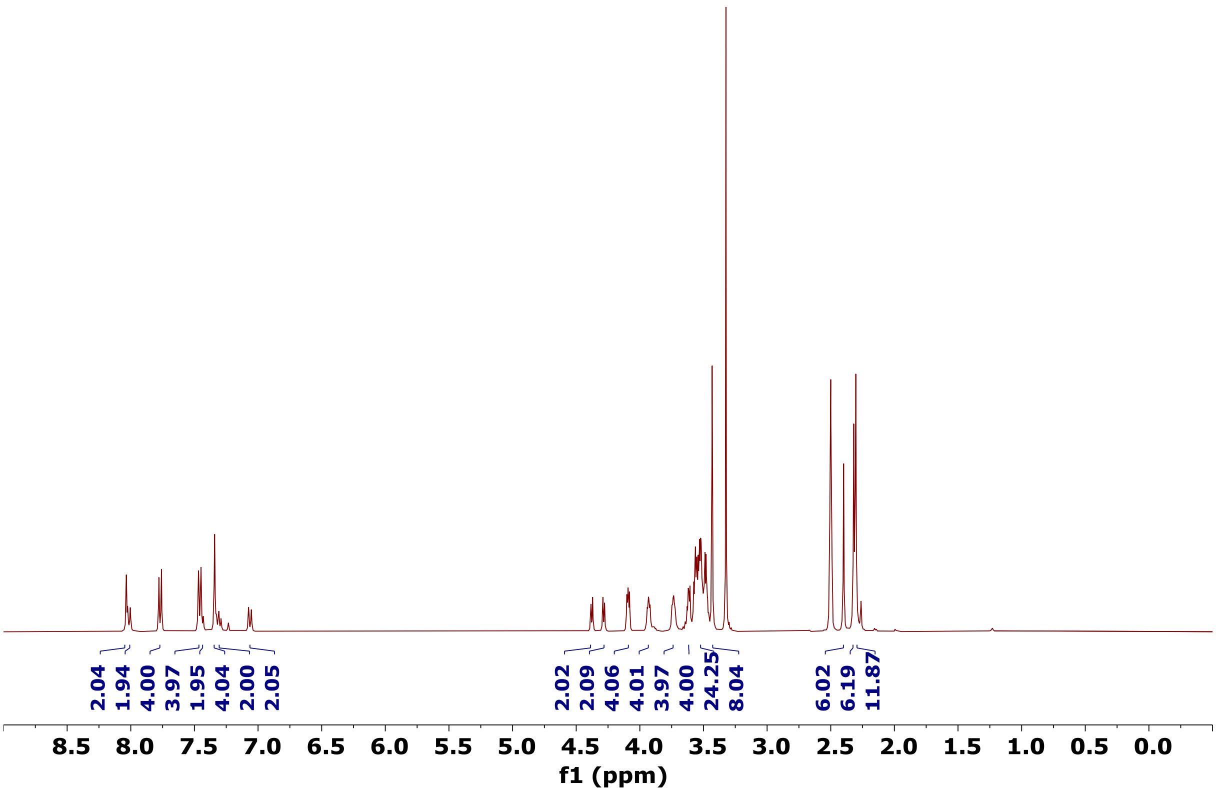

Figure S4: ^{1}H NMR spectrum of (*S*)-**Me-3$_6$** (DMSO-*d$_6$*, 298 K, 400 MHz).

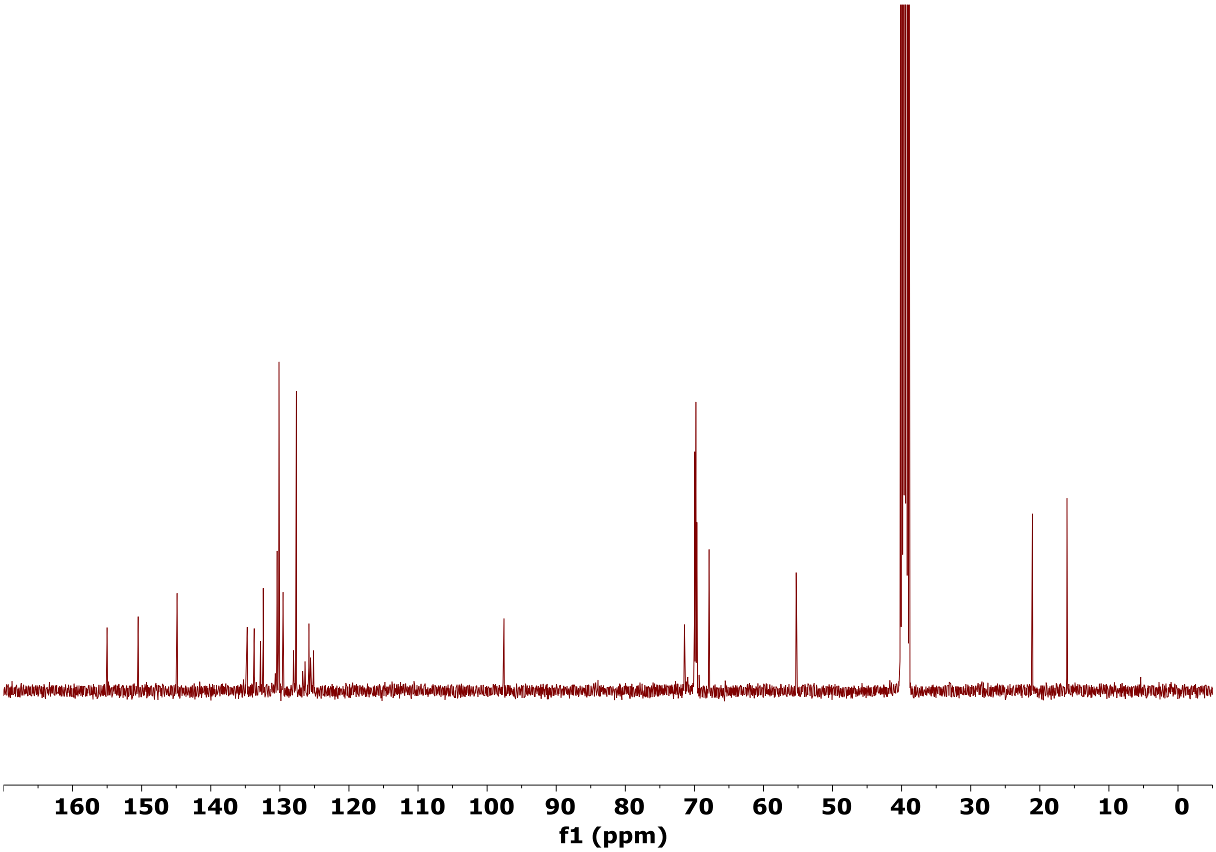

Figure S5: ^{13}C NMR spectrum of (*S*)-**Me-3$_6$** (DMSO-*d$_6$*, 298 K, 400 MHz).

Compound **H-3$_6$**

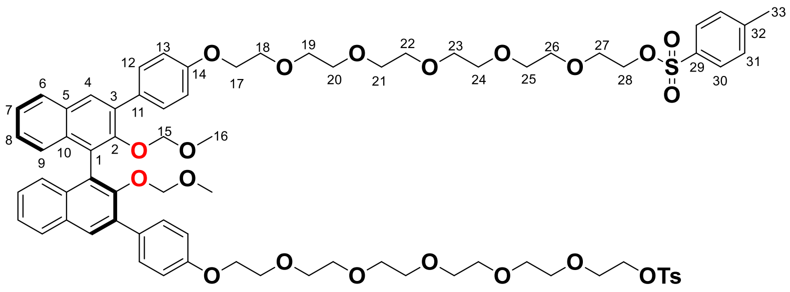

As described above (section 2.2.1), compound **H-5$_6$** was synthesized using **H-2** (55.8 mg, 0.0999 mmol, 1 equiv), cesium carbonate (72.0 mg, 0.220 mmol, 2.2 equiv) and chloroalcohol **10$_6$** (72.0 mg, 0.223 mmol, 2.2 equiv) in degassed acetonitrile (5.5 mL) as a colorless liquid and used without purification in the next step.

Compound **H-5$_6$** (25.1 mg, 0.0231 mmol, 1 equiv), *p*-toluenesulfonyl chloride (9.97 mg, 0.0523 mmol, 2.3 equiv), triethylamine (13.0 µL, 9.44 mg, 0.0933 mmol, 4.0 equiv), 4-(dimethylamino)pyridine (1.11 mg, 0.0091 mmol, 0.4 equiv) were reacted in dichloromethane (340 µL). The crude product was purified by column chromatography (cyclohexane/ethyl acetate 1:2) and afforded the product as a clear wax (20.2 mg, 0.0144 mmol, 60.9% yield).

$C_{74}H_{90}O_{22}S_2$: 1395.63 g/mol.

^{1}H-NMR (600 MHz, [D$_6$]-dimethyl sulfoxide, 298 K) δ [in ppm] = 8.04 (s, 2H, H-4), 8.02 (d, *J* = 8.2 Hz, 2H, H-6), 7.77 (d, *J* = 8.3 Hz, 4H, H-30), 7.63 (d, *J* = 8.8 Hz, 4H, H-12), 7.45 (d, *J* = 8.3 Hz, 4H, H-31), 7.46-7.43 (m, 2H, H-7), 7.31 (dd, *J* = 1.3 Hz, *J* = 8.3 Hz, 2H, H-8), 7.11-7.08 (m, 2H, H-9), 7.07 (d, *J* = 7.03 Hz, 4H, H-13), 4.35 (d, *J* = 5.5 Hz, 2H, H-15$_{1/2}$), 4.26 (d, *J* = 5.5 Hz, 2H, H-15$_{1/2}$), 4.18-4.15 (m, 4H, H-17), 4.10-4.08 (m, 4H, H-28), 3.78-3.76 (m, 4H, H-18), 3.61-3.59 (m, 4H, H-27), 3.58-3.47 (m, 24H, H-19, 20, 21, 24, 25, 26), 3.43 (bs, 8H, H-22, 23), 2.40 (s, 6H, H-33), 2.28 (S, 6H, H-16).

^{13}C-NMR (151 MHz, [D$_6$]-dimethyl sulfoxide, 298 K) δ [in ppm] = 157.94 (C-14), 150.56 (C-2), 144.87 (C-32), 134.53 (C-3), 132.68 (C-10), 132.39 (C-29), 130.62 (C-11),130.54 (C-5), 130.38 (C-12), 130.12 (C-31), 129.98 (C-4), 128.03 (C-6), 127.61 (C-30), 126.28 (C-8), 125.87 (C-1), 125.62 (C-9), 125.13 (C-7), 114.43 (C-13), 97.50 (C-15), 70.95 (C-27), 69.97 (C-28), 68.96 (C-18), 69.68, 69.63 (C-22, 23), 70.21, 69.81, 69.77, 69.75, 69.30, 67.86 (C-19, 20, 21, 24, 25, 26), 67.17 (C-17), 66.82 (C-17), 55.24 (C-16), 21.07 (C-33).

^{1}H,^{1}H-COSY (600 MHz / 600 MHz, [D$_6$]-dimethyl sulfoxide, 298 K) δ [in ppm] = 8.02/7.46-7.43 (H-6/H-7), 7.77/7.45 (H-30/H-31), 7.63/7.07 (H-12/H-13), 7.45/7.77 (H-31/H-30), 7.46-7.43/8.02, 7.31 (H-7/H-6, 8), 7.31/7.46-7.43, 7.11-7.08 (H-8/H-7, 9), 7.11-7.08/7.31 (H-9/H-8), 7.07/7.63 (H-13/H-12), 4.35/4.26 (H-15$_{1/2}$/ H-15$_{1/2}$), 4.26/4.35 (H-15$_{1/2}$/H-15$_{1/2}$), 4.18-4.15/3.78-3.76 (H-17/H-18), 4.10-4.08/3.61-3.59 (H-28/H-27), 3.78-3.76/4.18-4.15 (H-18/H-17), 3.61-3.59/4.10-4.08 (H-27/H-28), 3.58-3.47/3.43 (H-24+21/H-22+23), 3.43/3.58-3.47 (H-22+23/H-24+21).

^{1}H,^{13}C-GHSQC (600 MHz / 151 MHz, [D$_6$]-dimethyl sulfoxide, 298 K) δ (^{1}H) / δ (^{13}C) [in ppm] = 8.04/129.98 (H-4/C-4), 8.02/128.03 (H-6/C-6), 7.77/127.61 (H-30/C-30), 7.63/130.38 (H-12/C-12), 7.45/130.12 (H-31/C-31), 7.46-7.43/125.13 (H-7/C-7), 7.31/126.28 (H-8/C-8), 7.11-7.07/125.62 (H-9/C-9), 7.08/114.43 (H-13/C-13), 4.35/97.50 (H-15$_{1/2}$/C-15), 4.26/97.50 (H-15$_{1/2}$/C-15), 4.18-4.15/67.17 (H-17/C-17), 4.10-4.08/69.97 (H-28/C-28), 3.78-3.76/68.96 (H-18/C-18), 3.61-3.59/70.95 (H-27/C-27), 3.58-3.47/70.21, 69.81, 69.77, 69.75, 69.30, 67.86 (H-19, 20, 21, 24, 25, 26/ C-19, 20, 21, 24, 25, 26), 3.43/69.68, 69.63 (H-22+23/C-22+23), 2.40/21.07 (H-33/C-33), 2.28/55.24 (H-16/C-16).

^{1}H,^{13}C-GHMBC (600 MHz / 151 MHz, [D$_6$]-dimethyl sulfoxide, 298 K) δ (^{1}H) / δ (^{13}C) [in ppm] = 8.04/150.56, 132.68, 130.62, 128.03 (H-4/C-2, 10, 11, 6), 8.02/132.68, 129.98, 126.28 (H-6/C-10, 4, 8), 7.77/144.89 (H-30/C-32), 7.63/157.94, 134.53 (H-12/C-14, 3), 7.45/132.39, 21.07 (H-31/C-29, 33), 7.46-7.43/130.54, 125.62 (H-7/C-5, 9), 7.31/132.68, 128.03 (H-8/C-10, 6), 7.11-7.07/130.54, 125.87, 125.13 (H-9/C-5, 1, 7), 7.08/130.62 (H-13/C-11), 4.35/150.56, 55.24 (H-15$_{1/2}$/C-2, 16), 4.26/150.56, 55.24 (H-15$_{1/2}$/C-2, 16), 2.40/144.87, 130.12 (H-33/C-32, 31), 2.28/97.50 (H-16/C-15),

MS (ESI-pos, MeOH): *m/z* = 1417.5311 ([M+Na]$^+$, calcd. 1417.5257 [$C_{74}H_{90}O_{22}S_2Na$]$^+$).

IR (ATR-FT): $\tilde{\nu}$ (cm^{-1}) = 702, 749, 764, 879, 1098, 1262, 1275, 1457, 2871, 2999, 3005, 3054.

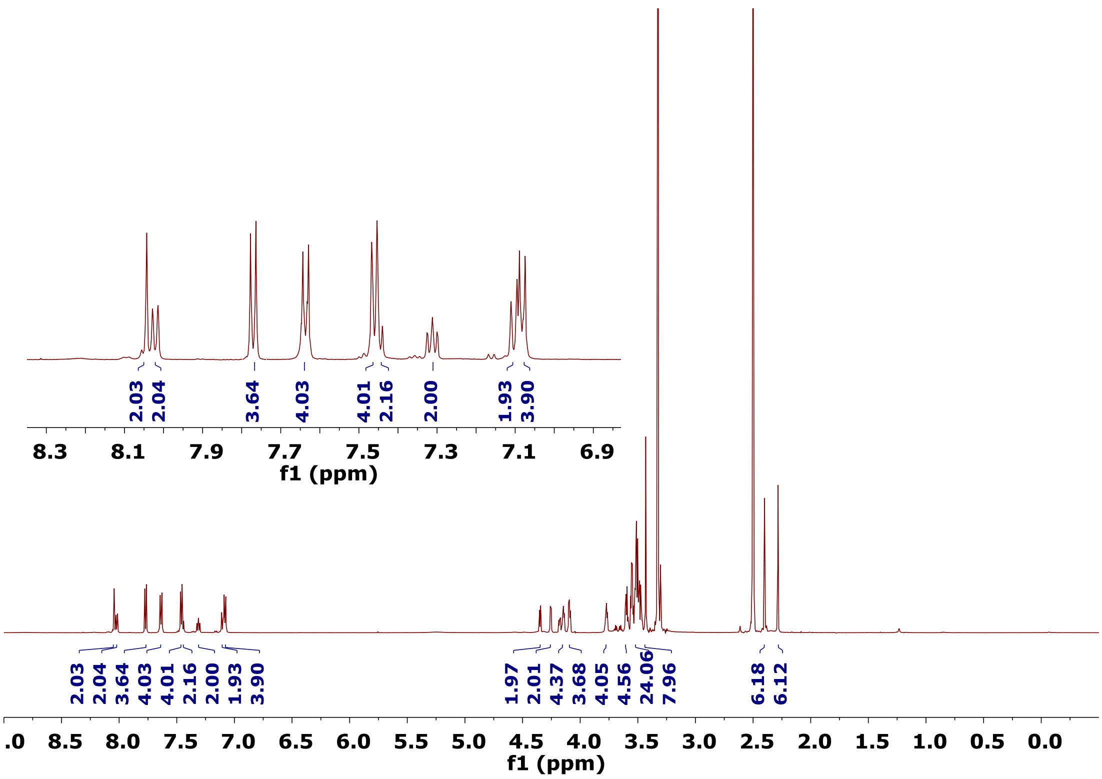

Figure S6: ^{1}H NMR spectrum of (*S*)-**H-3$_6$** (DMSO-*d$_6$*, 298 K, 400 MHz).

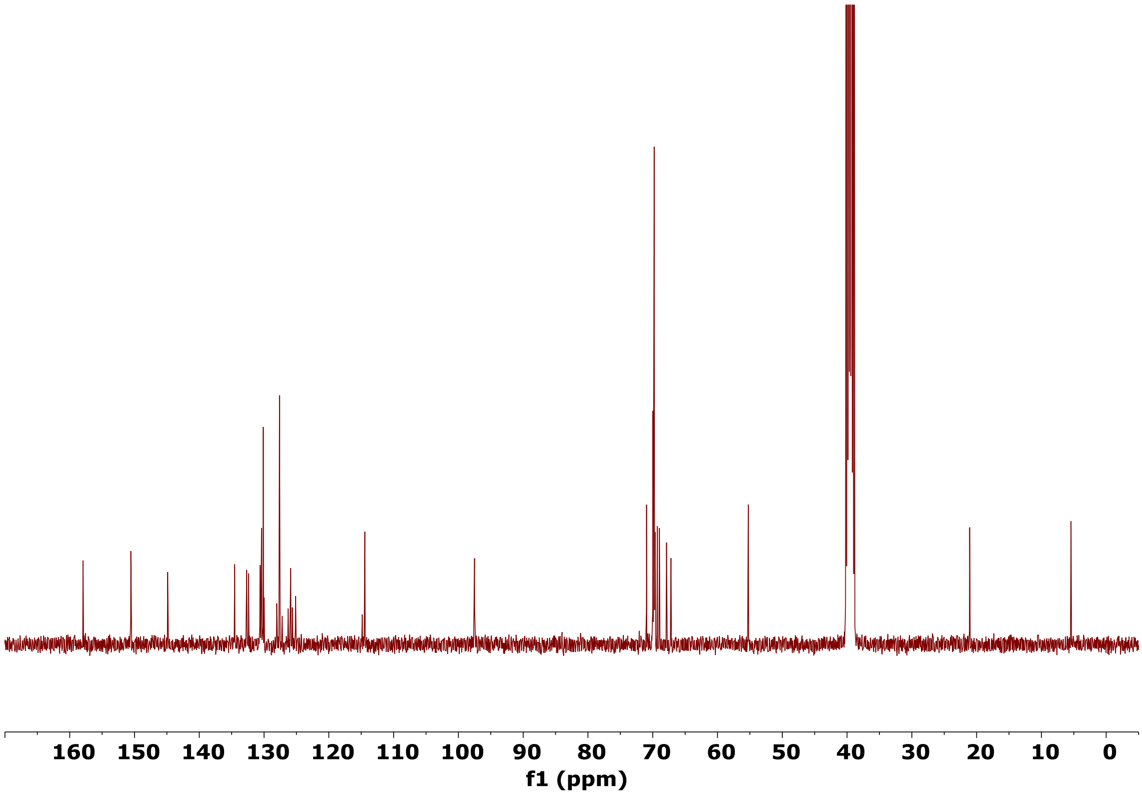

Figure S7: ^{13}C NMR spectrum of (*S*)-**H-3$_6$** (DMSO-*d$_6$*, 298 K, 400 MHz).

Compound **iPr-3$_6$**

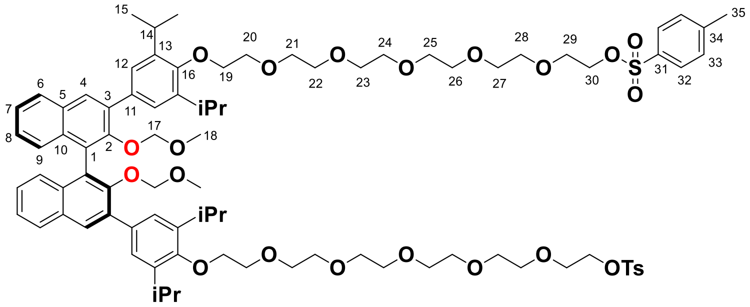

As described above (section 2.2.1), compound **iPr-5$_6$** was synthesized using **iPr-2** (71.8 mg, 0.0987 mmol, 1 equiv), cesium carbonate (72.2 mg, 0.221 mmol, 2.2 equiv) and chloroalcohol **10$_6$** (73.0 mg, 0.225 mmol, 2.2 equiv) in degassed acetonitrile (5.5 mL) as a light yellow liquid and was used without purification in the next step. *p*-Toluenesolfonyl chloride (140 mg, 0.0734 mmol, 2.5 equiv), triethylamine (16.7 µL, 12.1 mg, 0.120 mmol, 4.1 equiv), compound **iPr-5$_6$** (36.1 mg, 0.0288 mmol, 1 equiv), and 4-(dimethylamino)pyridine (1.47 mg, 0.0120 mmol, 0.4 equiv) were reacted in dichloromethane (0.5 mL). Column chromatography (MeOH/DCM 2:98) was used to purify the crude product, yielding a clear oil (32.2 mg, 0.0206 mmol, 72.4%).

$C_{86}H_{114}O_{22}S_2$: 1562.72 g/mol.

^{1}H-NMR (400 MHz, [D$_6$]-dimethyl sulfoxide, 298 K) δ [in ppm] = 8.07 (s, 2H, H-4), 8.05 (d, *J* = 7.9 Hz, 2H, H-6), 7.77 (d, *J* = 8.3 Hz, 4H, H-32), 7.48-7.45 (m, 6H, H-33, 7), 7.43 (s, 4H, H-12), 7.33 (dd, *J* = 8.4 Hz, *J* = 1.7 Hz, 2H, H-8), 7.11 (d, *J* = 8.4 Hz, 2H, H-9), 4.40 (d, *J* = 5.3 Hz, 2H, H-17$_{1/2}$), 4.27 (d, *J* = 5.3 Hz, 2H, H-17$_{1/2}$), 4.10 (t, *J* = 4.4 Hz, 4H, H-30), 3.89-3.87 (m, 4H, H-19), 3.77-3.75 (m, 4H, H-20), 3.65-3.62 (m, 4H, H-29), 3.59-3.47 (m, 24H, H-21, H-22, H-23, H-26, H-27, H-28), 3.44 (bs, 8H, H-24, H-25), 3.43-3.38 (m, 4H, H-14), 2.40 (s, 6H, H-35), 2.36 (s, 6H, H-18), 1.23 (d, *J* = 6.8 Hz, 12H, H-15$_{1/2}$), 1.20 (d, *J* = 6.8 Hz, 12H, H-15$_{1/2}$).

^{13}C-NMR (101 MHz, [D$_6$]-dimethyl sulfoxide, 298 K) δ [in ppm] = 152.14 (C-16), 150.57 (C-2), 144.84 (C-34), 141.25 (C-13), 135.29 (C-3), 134.45 (C-11), 132.72 (C-10), 132.40 (C-31), 130.47 (C-5), 130.10 (C-33), 129.96 (C-4), 128.15 (C-6), 127.61 (C-32), 126.38 (C-8), 125.77 (C-1), 125.47 (C-9), 125.13 (C-7), 125.00 (C-12), 97.62 (C-17), 73.98 (C-19), 70.13 (C-30), 69.95 (C-29), 69.93, 69.91, 69.84, 69.78, 67.87 (C-21, 22, 23, 26, 27, 28)*, 69.74 (C-20), 69.69 (C-24 or C-25), 69.65 (C-24 or C-25), 55.35 (C-18), 25.73 (C-14), 23.97 (C-15$_{1/2}$), 23.91 (C-15$_{1/2}$), 21.06 (C-35).

* Partially overlapping signals.

^{1}H,^{1}H-COSY (400 MHz / 400 MHz, [D$_6$]-dimethyl sulfoxide, 298 K) δ [in ppm] = 8.05/7.48-7.45 (H-6/H-7), 7.77/7.48-7.45 (H-32/H-33), 7.48-7.45/7.77 (H-33/H-32), 7.48-7.45 /8.05, 7.33 (H-7/H-6, 8), 7.33/7.11 (H-8/H-9), 7.11/7.33 (H-9/H-8), 4.40/4.27 (H-17$_{1/2}$/ H-17$_{1/2}$), 4.27/4.40 (H-17$_{1/2}$/ H-17$_{1/2}$), 4.10/3.59-3.47 (H-30/H-29), 3.89-3.87/3.77-3.75 (H-19/H-20), 3.77-3.75/3.89-3.87 (H-20/H-19), 3.59-3.47/4.10 (H-29/H-30), 3.43-3.38/1.23, 1.20 (H-14/H-15$_{1/2}$), 1.23/3.43-3.38 (H-15$_{1/2}$/H-14), 1.20/3.43-3.38 (H-15$_{1/2}$/H-14).

^{1}H,^{13}C-GHSQC (400 MHz / 101 MHz, [D$_6$]-dimethyl sulfoxide, 298 K) δ (^{1}H) / δ (^{13}C) [in ppm] = 8.07/129.96 (H-4/C-4), 8.05/128.15 (H-6/C-6), 7.77/127.61 (H-32/C-32), 7.47/130.10 (H-33/C-33), 7.48-7.45/125.13 (H-7/C-7), 7.43/125.00 (H-12/C-12), 7.33/126.38 (H-8/C-8), 7.11/125.47 (H-9/C-9), 4.40/97.62 (H-17$_{1/2}$/C-17), 4.27/97.62 (H-17$_{1/2}$/C-17), 4.10/70.13 (H-30/C-30), 3.89-3.87/73.98 (H-19/C-19), 3.77-3.75/69.74 (H-20/C-20), 3.65-3.62/69.95 (H-28/C-28), 3.59-3.47/69.93, 69.91, 69.84, 69.78, 67.87 (H-21, 22, 23, 26, 27, 28/C-21, 22, 23, 26, 27, 28), 3.44/69.69, 69.65 (H-24, 25/C-24, 25),

3.3-3.38/25.73 (H-14/C-14), 2.40/21.06 (H-35/C-35), 2.36/55.35 (H-18/C-18), 1.23/23.97 (H-$15_{1/2}$/C-$15_{1/2}$), 1.20/23.91 (H-$15_{1/2}$/C-$15_{1/2}$).

^{1}H,^{13}C-GHMBC (400 MHz / 101 MHz, [D$_6$]-dimethyl sulfoxide, 298 K) δ (^{1}H) / δ (^{13}C) [in ppm] = 8.07/150.57, 134.45, 132.72, 128.15 (H-4/C-2, 11, 10, 6), 8.05/132.72, 126.38 (H-6/C-10, 8), 7.77/144.84 (H-32/C-34), 7.48-7.45/132.40, 130.47, 125.47, 21.06 (H-33, 7/C-31, 5, 9, 35), 7.43/152.14, 135.29, 25.73 (H-12/C-16, 3, 14), 7.33/132.72, 128.15 (H-8/C-10, 6), 7.11/130.47, 125.77, 125.13 (H-9/C-5, 1, 7), 4.40/150.57, 55.35 (H-$17_{1/2}$/C-2, 18), 4.27/150.57, 55.35 (H-$17_{1/2}$/C-2, 18), 2.40/144.84, 130.10 (H-35/C-34, 33), 2.36/97.62 (H-18/C-17), 1.23/141.25, 25.73 (H-$15_{1/2}$/C-13, 14), 1.20/141.25, 25.73 (H-$15_{1/2}$/C-13, 14).

MS (ESI-pos, MeOH): *m/z* = 1497.6617 ([M+Na-(MOM)$_2$+H$_2$]$^+$, calcd. 1497.6611 [$C_{82}H_{106}O_{20}S_2Na$]$^+$).

IR (ATR-FT): $\tilde{\nu}$ (cm^{-1}) = 702, 749, 764, 890, 1139, 1262, 1275, 1456, 2989, 3009, 3053.

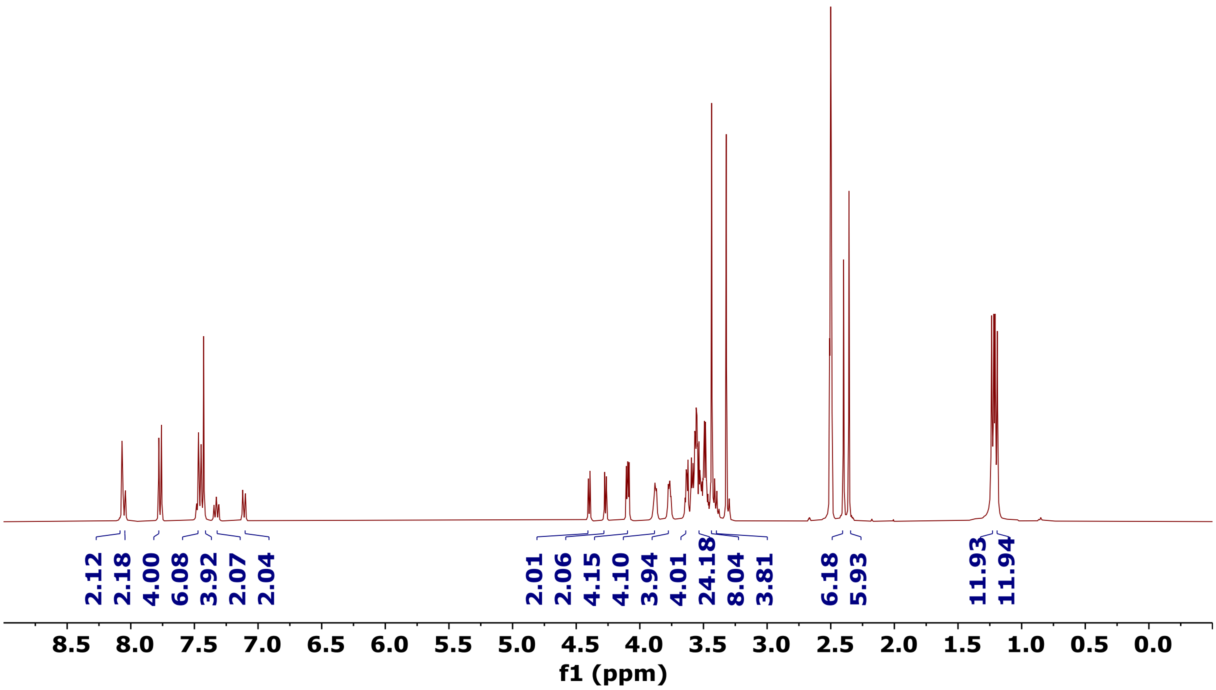

Figure S8: ^{1}H NMR spectrum of (*S*)-**iPr-3$_6$** (DMSO-d_6, 298 K, 400 MHz).

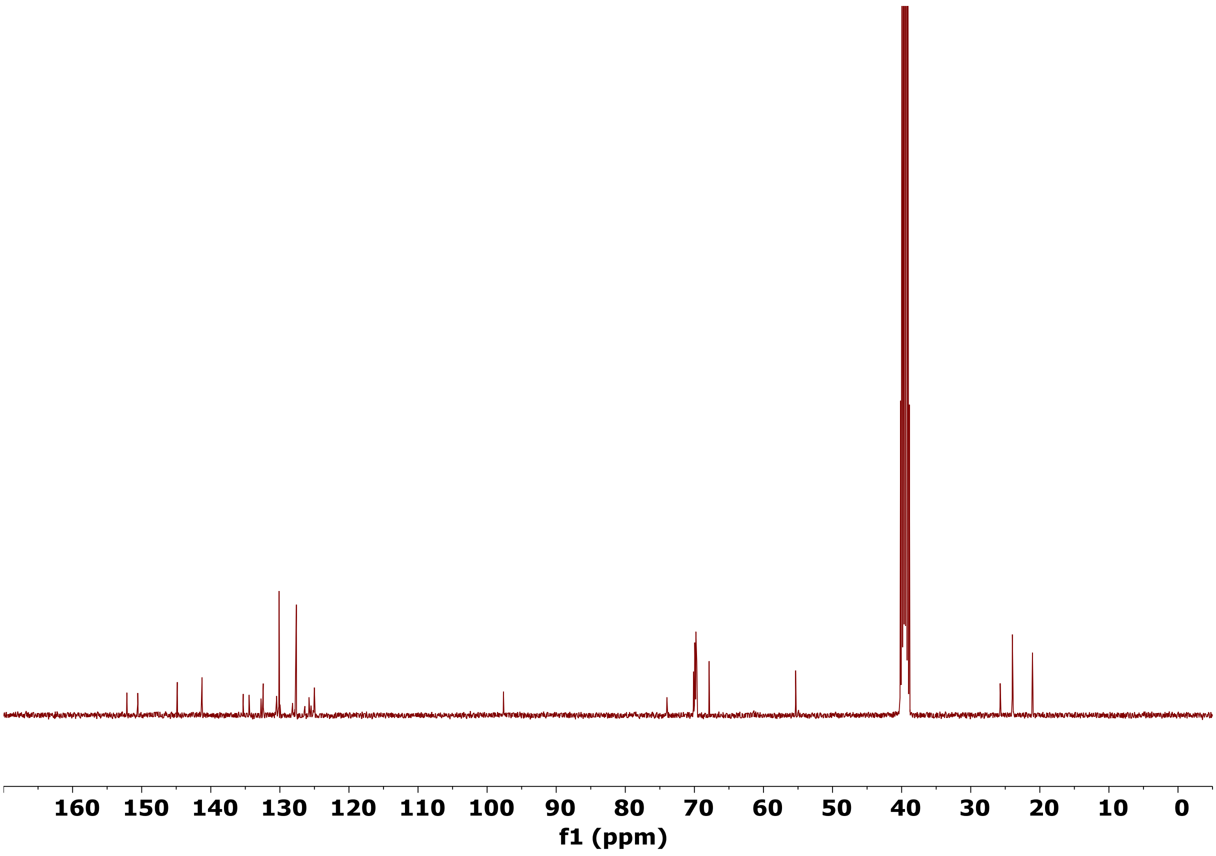

Figure S9: ^{13}C NMR spectrum of (*S*)-**iPr-3$_6$** (DMSO-d_6, 298 K, 400 MHz).

2.2.2. Synthesis of compound **Me/H/iPr-6$_2$**

Compound **Me/H/iPr-2** (1 equiv) and cesium carbonate (2.2 equiv), were dissolved in acetonitrile and stirred for 15 minutes. After the dropwise addition of dichloride **11$_2$** (2.5 equiv), the reaction mixture was stirred at 85 °C for 5 hours. Then 2 more equiv of the dichloride **11$_2$** were added and the reaction mixture was stirred under reflux for 18 hours. After cooling to room temperature, cesium carbonate was removed by filtration and all volatiles were removed in *vacuo*. The crude product was purified by column chromatography.

Compound **Me-6$_2$**

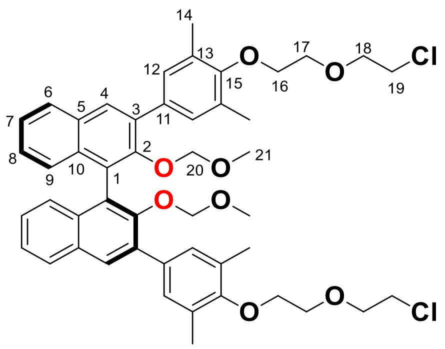

Compound **Me-6$_2$** was prepared as described above using **Me-2** (91.1 mg, 0.148 mmol, 1 equiv), dichloride **11$_2$** (44.0 µL, 53.7 mg, 0.375 mmol, 2.5 equiv) and cesium carbonate (124 mg, 0.380 mmol, 2.5 equiv) in acetonitrile (3 mL). After purification by column chromatography (cyclohexane/ethyl acetate 5:1), the desired product was obtained as a colorless wax (71.9 mg, 0.087 mmol, 58.7% yield).

$C_{48}H_{52}O_8Cl_2$: 827.84 g/mol.

^{1}H-NMR (400 MHz, [D$_1$]-chloroform, 298 K) δ [in ppm] = 7.77 (s, 2H, H-4), 7.73 (d, *J* = 8.1 Hz, 2H, H-6), 7.28-7.24 (m, 6H, H-7, 12), 7.13-7.10 (m, 4H, H-9, 8), 4.31 (d, *J* = 5.8 Hz, 2H, H-20$_{1/2}$), 4.27 (d, *J* = 5.8 Hz, 2H, H-20$_{1/2}$), 3.90-3.87 (m, 4H, H-16), 3.77-3.73 (m, 8H, H-17, 18), 3.57 (t, 5.92 Hz, 4H, H-19), 2.25-2.24 (bs, 18H, H-21, 14).

^{13}C-NMR (101 MHz, [D$_1$]-chloroform, 298 K) δ [in ppm] = 155.26 (C-15), 151.53 (C-2), 135.35 (C-3), 134.83 (C-10), 133.67 (C-11), 130.94 (C-13), 130.86 (C-5), 130.54 (C-4), 130.20 (C-12), 127.87 (C-6), 126.54 (C-8 or C-9), 126.27 (C-8 or C-9), 125.23 (C-7), 98.60 (C-20), 71.75 (C-16), 71.68 (C-18), 70.80 (C-17), 55.96 (C-21), 42.94 (C-19), 16.52 (C-14).

^{1}H,^{1}H-COSY (400 MHz / 400 MHz, [D$_1$]-chloroform, 298 K) δ [in ppm] = 7.73/7.28-7.24 (H-6/H-7), 7.28-7.24/7.73, 7.13-7.10 (H-7/H-6, 8), 7.28-7.24/2.25-2.24 (H-12/ 14), 7.13-7.10/7.28-7.24 (H-8, 9/H-7), 4.31/4.27 (H-20$_{1/2}$/ H-20$_{1/2}$), 4.27/4.31 (H-20$_{1/2}$/ H-20$_{1/2}$), 3.90-3.87/3.77-3.73 (H-16/H-17, 18), 3.77-3.73/3.90-3.87, 3.57 (H-17, 18/H-16, 19), 3.57/3.77-3.73 (H-19/H-17, 18), 2.25-2.24/7.28-7.24 (H-14/H-12).

^{1}H,^{13}C-GHSQC (400 MHz / 101 MHz, [D$_1$]-chloroform, 298 K) δ (^{1}H) / δ (^{13}C) [in ppm] = 7.77/130.54 (H-4/C-4), 7.73/127.87 (H-6/C-6), 7.28-7.24/125.23 (H-7/C-7), 7.28-7.24/130.20 (H-12/C-12), 7.13-7.10/126.54, 126.27 (H-8, 9/C-8, 9), 4.31/98.60 (H-20$_{1/2}$/C-20), 4.27/98.60 (H-20$_{1/2}$/C-20), 3.90-3.87/71.75 (H-16/C-16), 3.77-3.73/71.68, 70.80 (H-17, 18/C-18, 17), 3.57/42.94 (H-19/C-19), 2.25-2.24/55.96 (H-21/C-21), 2.25-2.24/16.52 (H-14/C-14).

^{1}H,^{13}C-GHMBC (400 MHz / 101 MHz, [D$_1$]-chloroform, 298 K) δ (^{1}H) / δ (^{13}C) [in ppm] = 7.77/151.53, 134.83, 133.67, 127.87 (H-4/C-2, 10, 11, 6), 7.73/134.83, 130.54, 126.54, 126.27 (H-6/C-10, 4, 8, 9), 7.28-7.24/155.26, 135.35, 130.94, 130.86, 126.54, 126.27 (H-7, 12/C-15, 3, 13, 5, 9, 8), 7.13-7.10 /134.83, 130.86, 127.87, 125.23 (H-8, 9/C-10, 5, 6, 7), 4.31/151.53, 55.96 (H-20$_{1/2}$/C-2, 21), 4.27/151.53, 55.96 (H-20$_{1/2}$/C-2, 21), 2.25-2.24/98.60 (H-21/C-20), 2.25-2.24/155.26, 130.20 (H-14/C-15, 12).

MS (ESI-pos, MeOH): *m/z* = 849.2907 ([M+Na]$^+$, calcd. 849.2931 [$C_{48}H_{52}O_8Cl_2Na$]$^+$).

IR (ATR-FT): $\tilde{\nu}$ (cm^{-1}) = 703, 749, 764, 970, 1157, 1200, 1262, 1275, 1457, 2869, 2927, 2998, 3005, 3053.

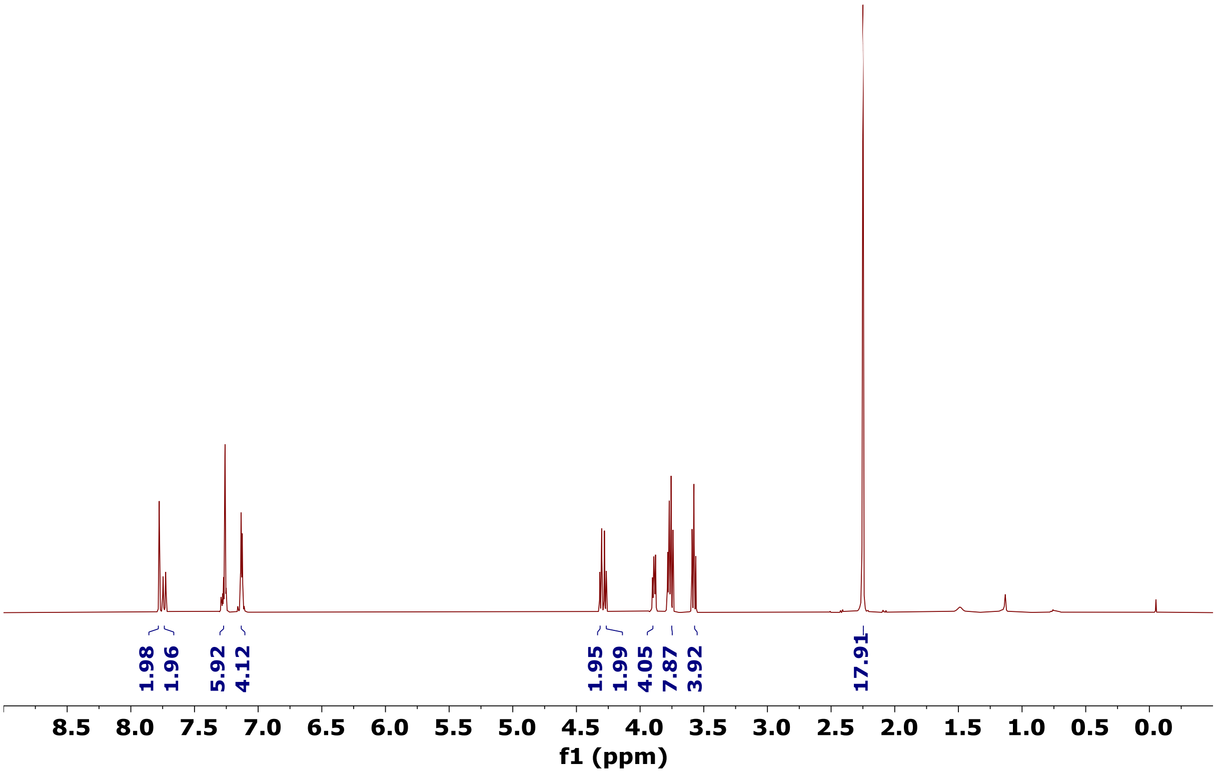

Figure S10: ^{1}H NMR spectrum of (*S*)-**Me-6$_2$** ($CDCl_3$, 298 K, 400 MHz).

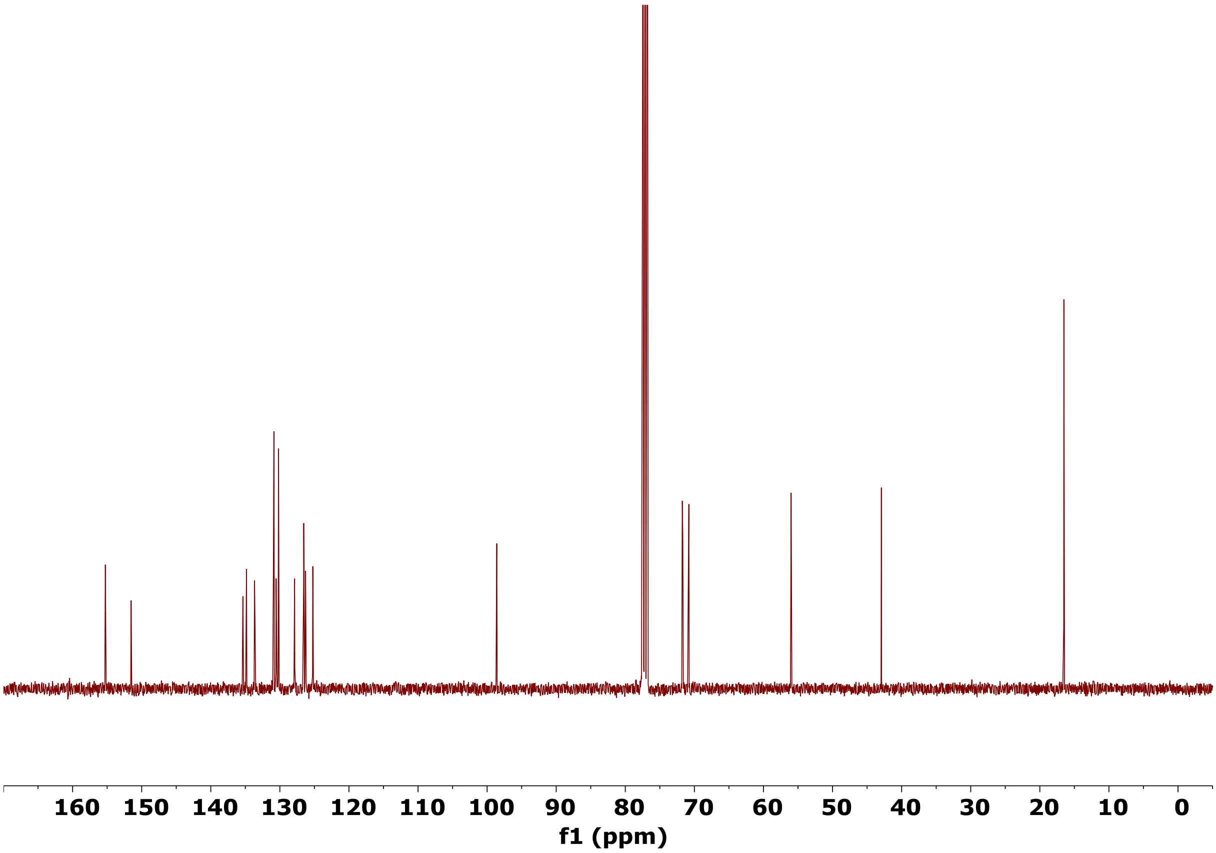

Figure S11: ^{13}C NMR spectrum of (S)-**Me-6$_2$** (DMSO-d_6, 298 K, 400 MHz).

Compound **H-6$_2$**

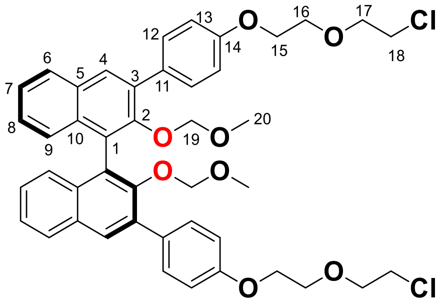

Compound **H-6$_2$** was prepared as described above using **H-2** (92.9 mg, 0.166 mmol, 1 equiv), dichloride **11$_2$** (50.0 µL, 61.0 mg, 0.427 mmol, 2.5 equiv) and Cs_2CO_3 (140 mg, 0.429 mmol, 2.5 equiv) in acetonitrile (3.5 mL). After purification by column chromatography (cyclohexane/ethyl acetate 3:1), the desired product was obtained as a colorless wax (99.9 mg, 0.129 mmol, 78.0% yield).

$C_{44}H_{44}O_8Cl_2$: 771.73 g/mol.

^{1}H-NMR (400 MHz, [D$_1$]-chloroform, 298 K) δ [in ppm] = 8.14 (s, 2H, H-4), 8.09 (d, *J* = 8.1 Hz, 2H, H-6), 7.92 (d, *J* = 8.7 Hz, 4H, H-12), 7.64-7.60 (m, 2H, H-7), 7.51-7.46 (m, 4H, H-8, 9), 7.25 (d, *J* = 8.8 Hz, 4H, H-13), 4.63 (d, *J* = 5.7 Hz, 2H, H-19$_{1/2}$), 4.58 (d, *J* = 5.7 Hz, 2H, H-19$_{1/2}$), 4.44 (t, 4.7 Hz, 4H, H-15), 4.16 (t, 4.8 Hz, 4H, H-16), 4.09 (t, 5.8 Hz, 4H, H-17), 3.91 (t, 6.0 Hz, 4H, H-18), 2.58 (s, 6H, H-20).

^{13}C-NMR (400 MHz, [D$_1$]-chloroform, 298 K) δ [in ppm] = 158.24 (C-14), 151.44 (C-2), 135.06 (C-3), 133.56 (C-10), 131.84 (C-11), 131.02 (C-5), 130.85 (C-12), 130.32 (C-4), 127.86 (C-6), 126.67 (C-8), 126.55 (C-9), 126.22 (C-1), 125.24 (C-7), 114.63 (C-13), 98.47 (C-19), 71.72 (C-17), 69.96 (C-16), 67.61 (C-15), 56.01 (C-20), 42.85 (C-18).

^{1}H,^{1}H-COSY (400 MHz / 400 MHz, [D$_1$]-chloroform, 298 K) δ [in ppm] = 8.08/7.64-7.60 (H-6/H-7), 7.92/7.25 (H-12/H-13), 7.64-7.60/7.46-7.51 (H-7/H-8, 9), 7.51-7.46 /7.64-7.60 (H-8, 9/H-7), 7.25/7.92 (H-13/H-12), 4.63/4.58 (H-19$_{1/2}$/ H-19$_{1/2}$), 4.58/4.63 (H-19$_{1/2}$/H-19$_{1/2}$), 4.44/4.16 (H-15/H-16), 4.16/4.44 (H-16/H-15), 4.09/3.91 (H-17/H-18), 3.91/4.09 (H-18/H-17).

^{1}H,^{13}C-GHSQC (400 MHz / 101 MHz, [D$_1$]-chloroform, 298 K) δ (^{1}H) / δ (^{13}C) [in ppm] = 8.14/130.32 (H-4/C-4), 8.09/127.86 (H-6/C-6), 7.92/130.85 (H-12/C-12), 7.64-7.60/125.24 (H-7/C-7), 7.51-7.46/126.67, 126.55 (H-8, 9/C-8, 9), 7.25/114.63 (H-13/C-13), 4.63/98.47 (H-19$_{1/2}$/C-19), 4.58/98.47 (H-19$_{1/2}$/C-19), 4.44/67.61 (H-15/C-15), 4.16/69.96 (H-16/C-16), 4.09/71.72 (H-17/C-17), 3.91/42.85 (H-18/C-18), 2.58/56.01 (H-20/C-20).

^{1}H,^{13}C-GHMBC (400 MHz / 101 MHz, [D$_1$]-chloroform, 298 K) δ (^{1}H) / δ (^{13}C) [in ppm] = 8.14/151.44, 133.56, 131.84, 127.86 (H-4/C-2, 10, 11, 6), 8.09/133.56, 130.32, 126.67 (H-6/C-10, 4, 8), 7.92/158.24, 135.06, 114.63 (H-12/C-14, 3, 13), 7.64-7.60/131.02, 126.55 (H-7/C-5, 9), 7.51-7.46 /133.56, 131.02, 127.86, 126.22, 125.24 (H-8, 9/C-10, 5, 6, 1, 7), 7.25/158.24, 131.84 (H-13/C-14, 11), 4.63/151.44, 56.01 (H-19$_{1/2}$/C-2, 20), 4.58/151.44, 56.01 (H-19$_{1/2}$/C-2, 20), 4.09/42.85 (H-17/C-18), 3.91/71.72 (H-18/C-17), 2.58/98.47 (H-20/C-19).

MS (ESI-pos, MeOH): *m/z* = 793.2311 ([M+Na]$^+$, calcd. 793.2305 [$C_{44}H_{44}O_8Cl_2Na$]$^+$).

IR (ATR-FT): $\tilde{\nu}$ (cm^{-1}) = 702, 750, 764, 898, 1097, 1140, 1189, 1263, 1275, 1361, 1512, 2877, 2987, 3004, 3052.

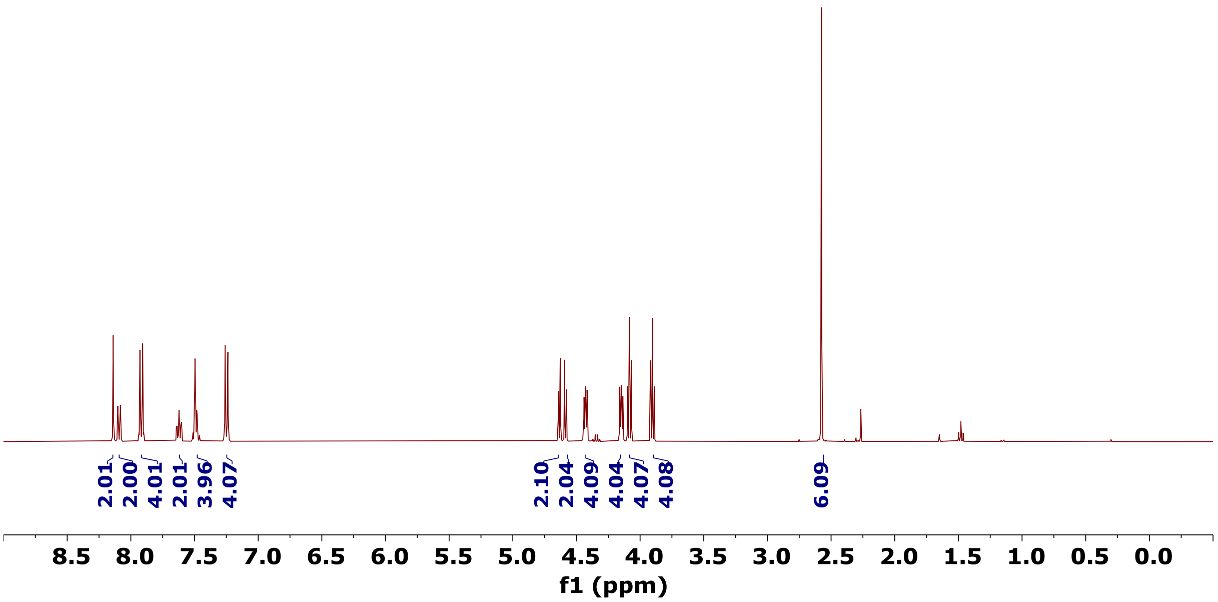

Figure S12: ^{1}H NMR spectrum of (*S*)-**H-6₂** (CDCl₃, 298 K, 400 MHz).

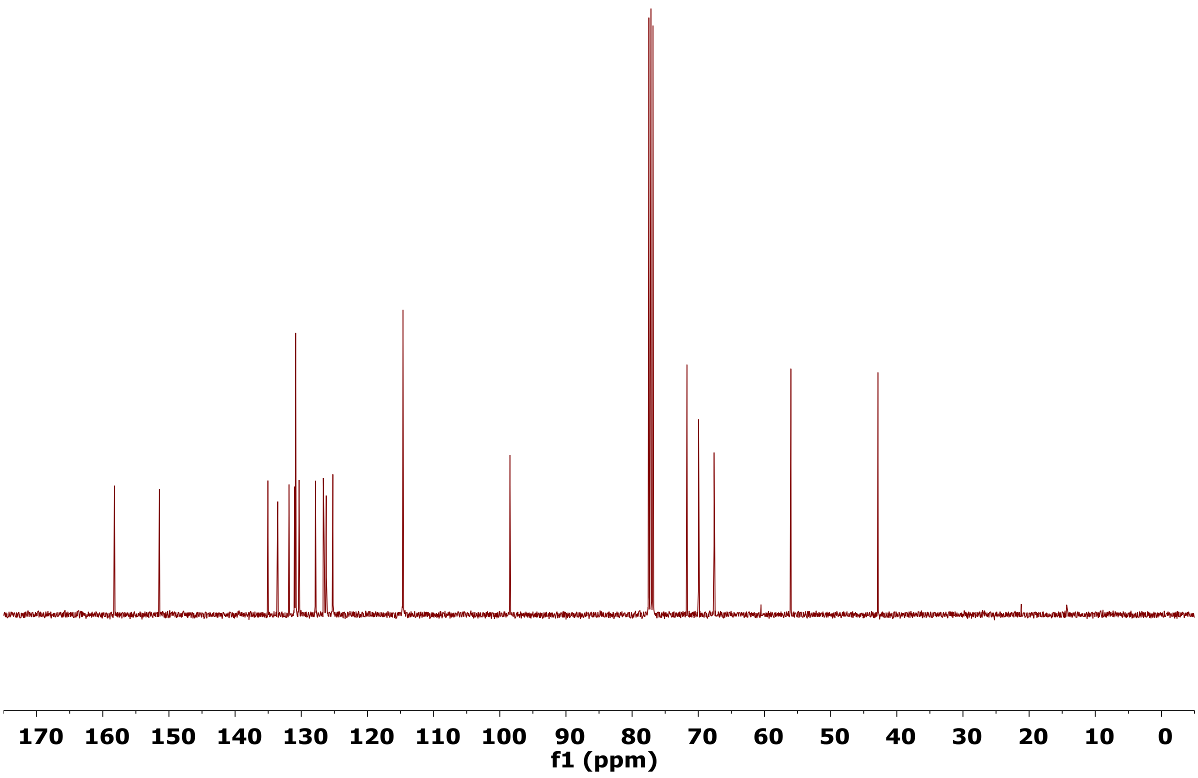

Figure S13: ^{13}C NMR spectrum of (*S*)-**H-6₂** (CDCl₃, 298 K, 400 MHz).

Compound **iPr-6$_2$**

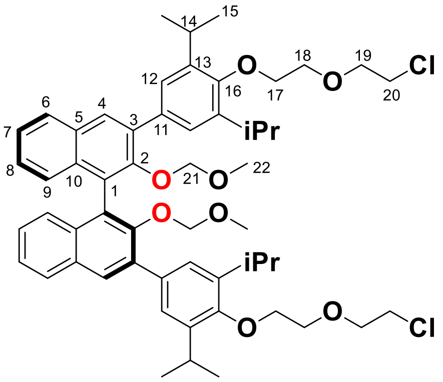

Compound **iPr-6$_2$** was prepared according to the general procedure described in section 2.2.2. **iPr-2** (70.7 mg, 0.0972 mmol, 1 equiv), dichloride **11$_2$** (29.0 µL, 35.4 mg, 0.247 mmol, 2.5 equiv) and cesium carbonate (81.8 mg, 252 µmol, 2.5 equiv) dissolved in acetonitrile (2 mL). After purification by column chromatography (cyclohexane/ethyl acetate 9:1), the desired product was obtained as a white solid (69.6 mg, 0.0741 mmol, 76.2% yield).

$C_{56}H_{68}O_8Cl_2$: 940.05 g/mol.

^{1}H-NMR (400 MHz, [D$_1$]-chloroform, 298 K) δ [in ppm] = 7.94 (s, 2H, H-4), 7.90 (d, *J* = 8.1 Hz, 2H, H-6), 7.48 (s, 4H, H-12), 7.41 (dd, *J* = 1.7 Hz, *J* = 6.5 Hz, 2H, H-7), 7.32 (d, *J* = 8.5 Hz, 2H, H-9), 7.29-7.26 (m, 2H, H-8), 4.43 (d, *J* = 5.7 Hz, 2H, H-21$_{1/2}$), 4.40 (d, *J* = 5.7 Hz, 2H, H-21$_{1/2}$), 4.01-3.94 (m, 4H, H-17), 3.93-3.91 (m, 4H, H-18), 3.90 (t, *J* = 5.9 Hz, 4H, H-19), 3.72 (t, *J* = 5.8 Hz, 4H, H-20), 3.46 (hept., *J* = 6.9 Hz, 4H, H-14), 2.40 (s, 6H, H-22), 1.31 (d, *J* = 6.9 Hz, 12H, H-15$_{1/2}$), 1.28 (d, *J* = 6.9 Hz, 12H, H-15$_{1/2}$).

^{13}C-NMR (101 MHz, [D$_1$]-chloroform, 298 K) δ [in ppm] = 152.35 (C-16), 151.52 (C-2), 141.67 (C-13), 135.73 (C-3), 135.32 (C-11), 133.43 (C-10), 130.83 (C-5), 130.06 (C-4), 127.78 (C-6), 126.46 (C-8), 126.43 (C-1), 126.02 (C-9), 125.49 (C-12), 125.00 (C-7), 98.32 (C-21), 74.01 (C-18), 71.65 (C-17), 70.63 (C-19), 42.84 (C-20), 55.82 (C-22), 26.42 (C-14), 24.20 (C-15$_{1/2}$), 24.15 (C-15$_{1/2}$).

^{1}H,^{1}H-COSY (400 MHz / 400 MHz, [D$_1$]-chloroform, 298 K) δ [in ppm] = 7.90/7.41 (H-6/H-7), 7.41/7.90, 7.29-7.26 (H-7/H-6, 8), 7.32/7.29-7.26 (H-9/H-8), 7.29-7.21/7.41, 7.32 (H-8/H-7, 9), 4.01-3.94/3.93-3.91 (H-17/H-18), 3.93-3.91/4.01-3.94 (H-18/H-17), 3.90/3.72 (H-19/H-20), 3.72/3.90 (H-20/H-19), 3.46/1.31, 1.28 (H-14/H-15$_{1/2}$), 1.31/3.40 (H-15$_{1/2}$/H-14), 1.28/3.40 (H-15$_{1/2}$/H-14).

^{1}H,^{13}C-GHSQC (400 MHz / 101 MHz, [D$_1$]-chloroform, 298 K) δ (^{1}H) / δ (^{13}C) [in ppm] = 7.94/130.06 (H-4/C-4), 7.90/127.78 (H-6/C-6), 7.48/125.49 (H-12/C-12), 7.41/125.00 (H-7/C-7), 7.32/126.02 (H-9/C-9), 7.29-7.26/126.46 (H-8/C-8), 4.43/98.32 (H-21$_{1/2}$/C-21), 4.40/98.32 (H-21$_{1/2}$/C-21), 4.01-3.94/71.65 (H-17/C-17), 3.93-3.91/74.01 (H-18), 3.90/70.63 (H-19/C-19), 3.72/42.84 (H-20/C-20), 3.46/26.42 (H-14/C-14), 2.40/55.82 (H-22/C-22), 1.31+1.28/24.20+24.15 (H-15$_{1/2}$/C-15$_{1/2}$).

^{1}H,^{13}C-HMBC (400 MHz / 101 MHz, [D$_1$]-chloroform, 298 K) δ (^{1}H) / δ (^{13}C) [in ppm] = 7.94/151.52, 135.32, 133.43, 127.78 (H-4/C-2, 11, 10, 6), 7.90/133.43, 130.06, 126.46 (H-6/C-10, 4, 8), 7.48/152.35, 135.73, 26.42 (H-12/C-16, 3, 14), 7.41/130.83, 126.02 (H-7/C-5, 9), 7.32/130.83, 126.43, 125.00 (H-9/C-5, 1, 7), 7.29-7.26/133.43, 127.78 (H-8/C-10, 6), 4.43/55.82 (H-21$_{1/2}$/C-22), 4.40/55.82 (H-21$_{1/2}$/C-22), 3.46/152.35, 141.67, 125.49, 24.20, 24.15 (H-14/C-16, 13, 12, 15), 2.40/98.32 (H-22/C-21), 1.31/141.67 (H-15$_{1/2}$/C-13), 1.28/141.67 (H-15$_{1/2}$/C-13).

MS (ESI-pos, MeOH): *m/z* = 961.4169 ([M+Na]$^+$, calcd. 961.4183 [$C_{56}H_{68}O_8Cl_2Na$]$^+$).

IR (ATR-FT): $\tilde{\nu}$ (cm^{-1}) = 702, 749, 764, 897, 1142, 1201, 1263, 1275, 1368, 1471, 2870, 2988, 3005, 3053.

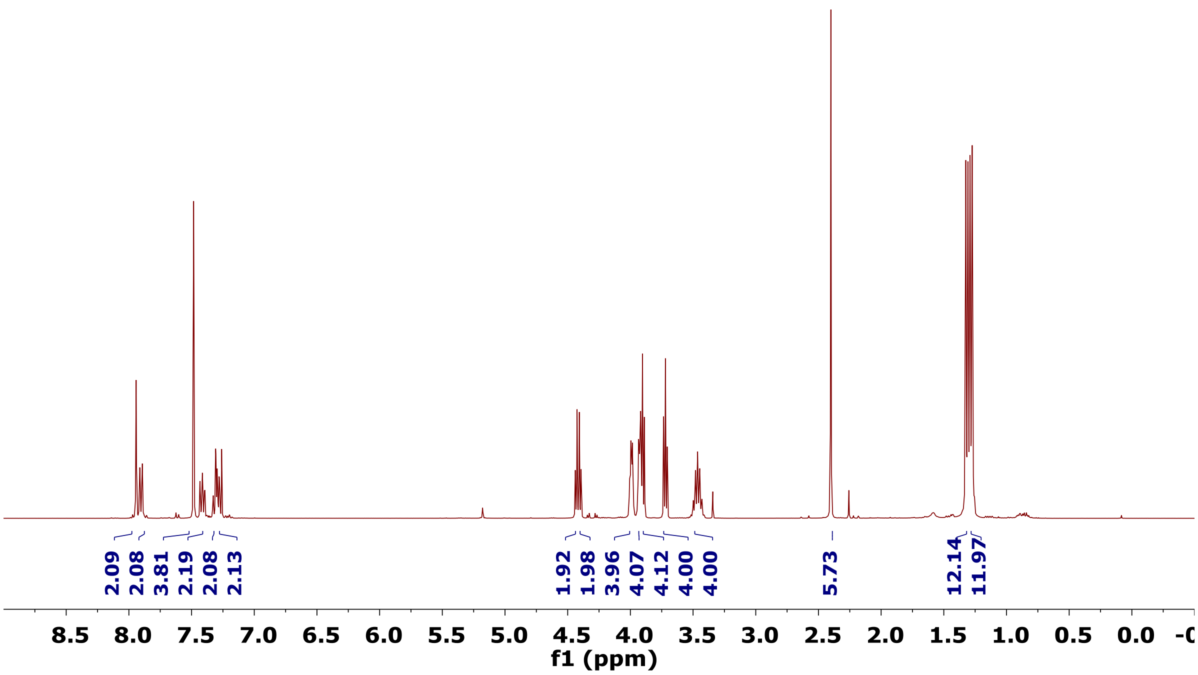

Figure S14: ^{1}H NMR spectrum of (*S*)-**iPr-6$_2$** ($CDCl_3$, 298 K, 400 MHz).

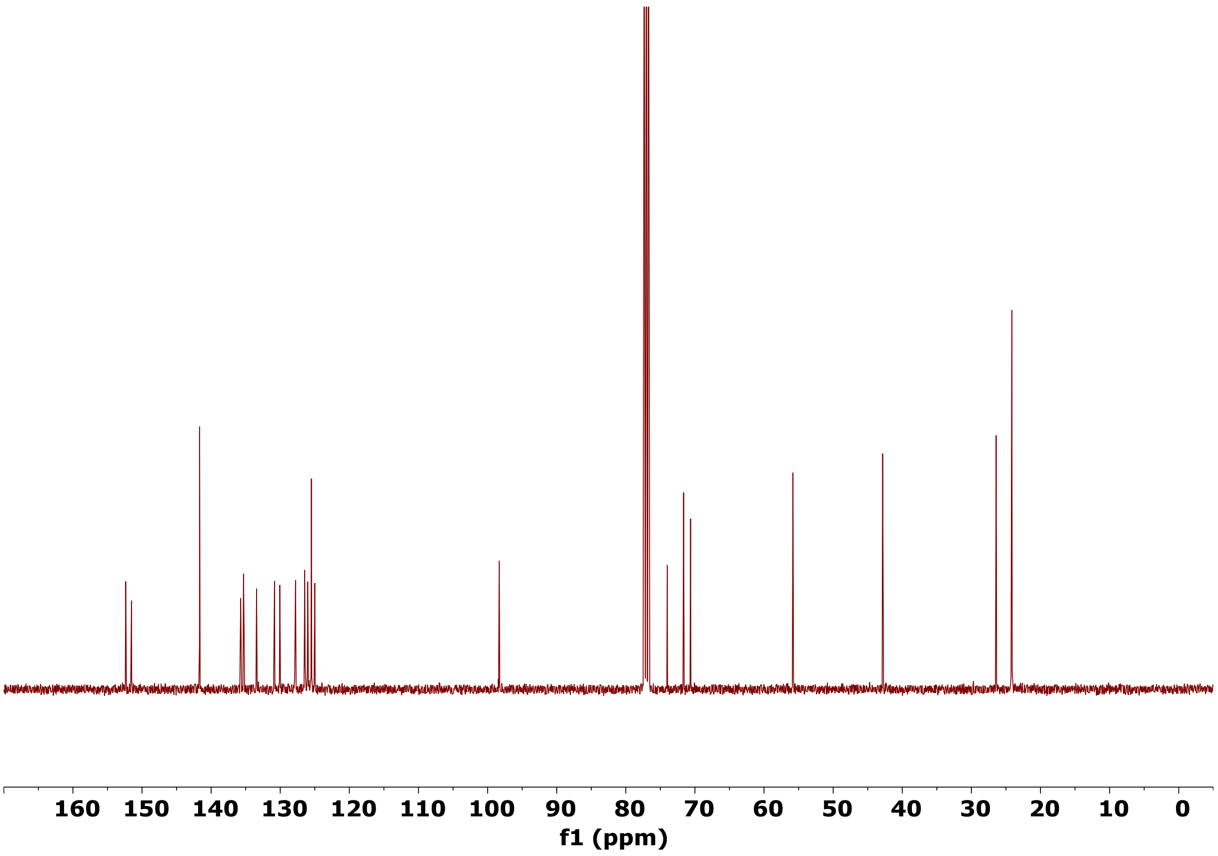

Figure S15: ^{13}C NMR spectrum of (*S*)-**iPr-6$_2$** ($CDCl_3$, 298 K, 400 MHz).

2.2.3. Synthesis of compounds $7_{5,6,7,8}$

The synthesis of compounds $\mathbf{7_{5,6,7,8}}$ was performed according to the procedures reported in the literature for $\mathbf{7_6}$[2] The corresponding 2,6-(**Me**/**H**/**iPr**)$_2$-4-(4,4,5,5-tetramethyl-1,3,2-dioxaborolan-2-yl)phenol (2 equiv) and cesium carbonate (3 equiv) were suspended in degassed acetonitrile and stirred for 15 minutes under argon. After the addition of the appropriate oligo-ethylene glycol bistosylate **8** (1 equiv) the reaction mixture was stirred at 85 °C for five hours. After cooling to room temperature, cesium carbonate was removed by filtration and all volatiles were removed in vacuo. The crude product was purified by column chromatography.

Compound 7_5

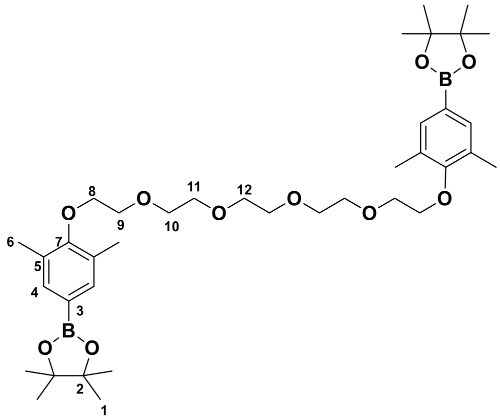

Compound $\mathbf{7_5}$ was prepared as described in section 2.2.3 using 2,6-dimethyl-4-(4,4,5,5-tetramethyl-1,3,2-dioxaborolan-2-yl)phenol (323 mg, 1.30 mmol, 2.0 equiv), cesium carbonate (640 mg, 1.96 mmol, 3 equiv) and $\mathbf{8_5}$ (362 mg, 662 µmol, 1 equiv) in degassed acetonitrile (2.5 mL). After purification by column chromatography (cyclohexane/ethyl acetate 3:1) the desired product was obtained as a yellow oil (268 mg, 384 µmol, 58.0% yield).

$\mathbf{C_{38}H_{60}O_{10}B_2}$: 698.51 g/mol.

^{1}H-NMR (400 MHz, [D$_1$]-chloroform, 298 K) δ [in ppm] = 7.47 (s, 4H, H-4), 3.94 (t, *J* = 4.3 Hz, 4H, H-8), 3.82 (t, *J* = 4.3 Hz, 4H, H-9), 3.73-3.68 (m, 12H, H-10, 11, 12), 2.28 (s, 12H, H-6), 1.33 (s, 24H, H-1).

^{13}C-NMR (400 MHz, [D$_1$]-chloroform, 298 K) δ [in ppm] = 158.63 (C-7), 135.59 (C-4), 131.30 (C-3), 130.35 (C-5), 83.64 (C-2), 71.30 (C-8), 70.85, 70.68, 70.66 (C-10, 11, 12), 70.50 (C-9), 24.83 (C-1), 16.06 (C-6).

^{1}H,^{1}H-COSY (400 MHz / 400 MHz, [D$_1$]-chloroform, 298 K) δ [in ppm] = 7.47/2.28 (H-4/H-6), 3.94/3.82 (H-8/H-9), 3.82/3.94 (H-9/H-8), 2.28/7.47 (H-6/H-4).

^{1}H,^{13}C-GHSQC (400 MHz / 101 MHz, [D$_1$]-chloroform, 298 K) δ (^{1}H) / δ (^{13}C) [in ppm] = 7.47/135.59 (H-4/C-4), 3.94/71.30 (H-8/C-8), 3.82/70.50 (H-9/C-9), 3.73-3.68/70.85, 70.68, 70.66 (H-10, 11, 12/C-10, 11, 12), 2.28/16.06 (H-6/C-6), 1.33/24.83 (H-1/C-1).

^{1}H,^{13}C-GHMBC (400 MHz / 101 MHz, [D$_1$]-chloroform, 298 K) δ (^{1}H) / δ (^{13}C) [in ppm] = 7.47/158.63, 16.06 (H-4/C-7, 6), 2.28/ 158.63, 135.59, 130.35 (H-6/C-7, 4, 5), 1.33/83.64 (H-1/C-2).

MS (ESI-pos, MeOH): *m/z* = 721.4324 ($[M+Na]^+$, calcd. 721.4287 for $[C_{38}H_{60}O_{10}B_2Na]^+$), *m/z* = 716.4756 ($[M+NH_4]^+$, calcd. 716.4724 for $[C_{38}H_{60}O_{10}B_2NH_4]^+$).

IR (ATR-FT): $\tilde{\nu}$ (cm^{-1}) = 702, 731, 749, 764, 1142, 1201, 1264, 1275, 1367, 1988, 3004, 3053.

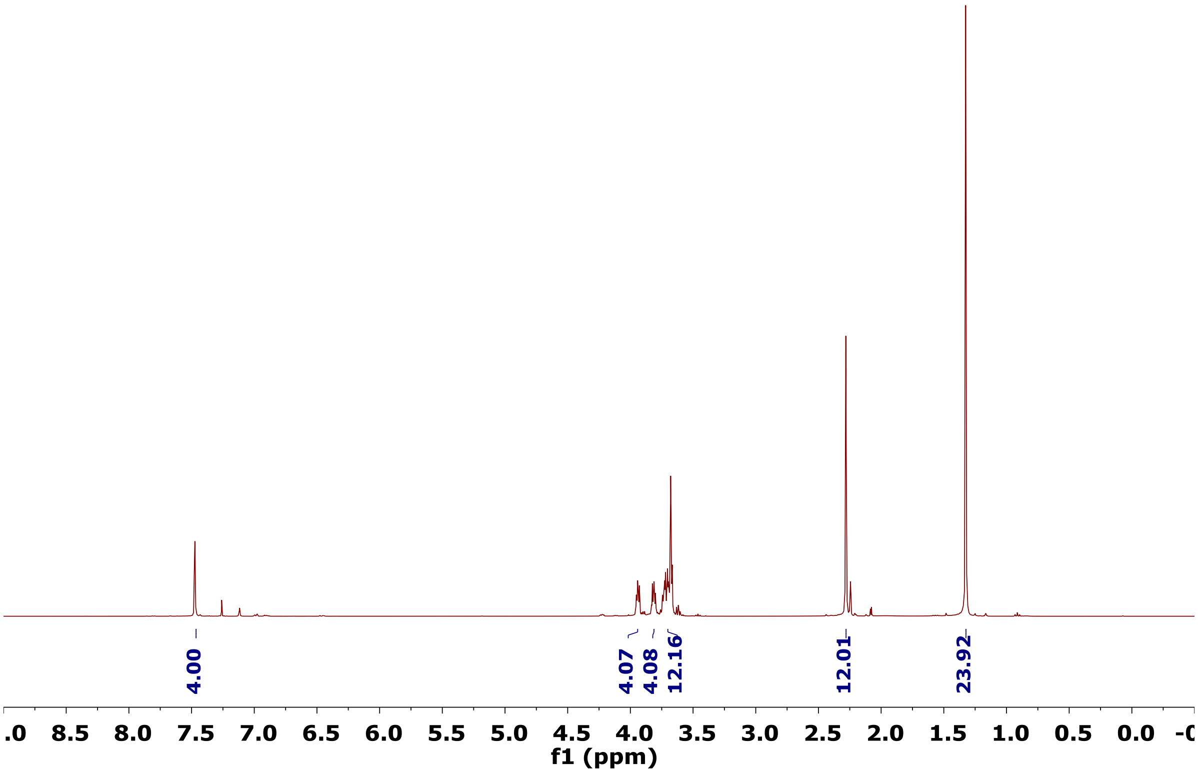

Figure 16: ^{1}H NMR spectrum of (*S*)-**7**$_5$ ($CDCl_3$, 298 K, 400 MHz).

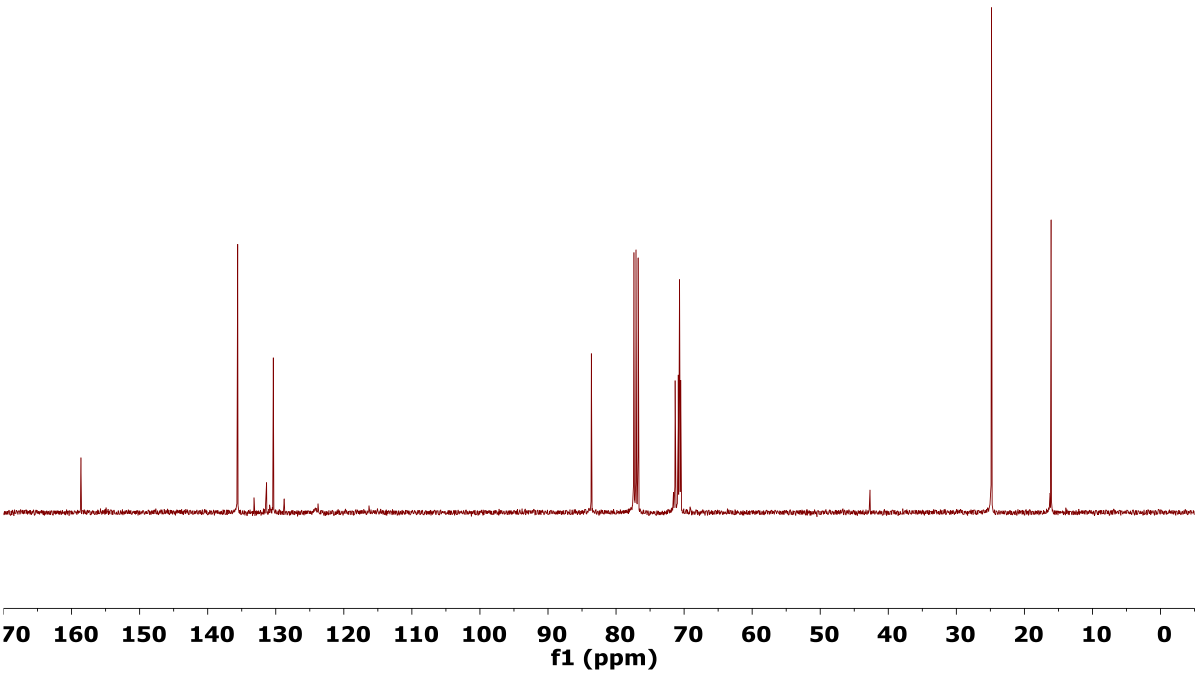

Figure S17: ^{13}C NMR spectrum of (*S*)-**7**$_5$ ($CDCl_3$, 298 K, 400 MHz).

Compound 7_6

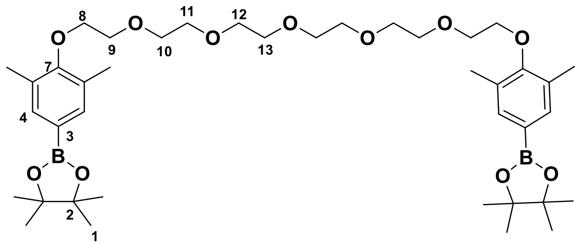

Compound $\mathbf{7_6}$ was prepared as described in section 2.2.3 using 2,6-dimethyl-4-(4,4,5,5-tetramethyl-1,3,2-dioxaborolan-2-yl)phenol (478 mg, 1.93 mmol, 2.0 equiv), cesium carbonate (1.88 g, 5.78 mmol, 3 equiv) and $\mathbf{8_6}$ (568 mg, 0.962 mmol, 1 equiv) dissolved in degassed acetonitrile (5 mL). After purification by column chromatography (cyclohexane/ethyl acetate 2:1) the product was obtained as a colorless oil (555 mg, 749 µmol, 78.0% yield). Spectroscopic data matches the literature.[2]

$C_{41}H_{66}O_{10}B_2$: 740.59 g/mol.

^{1}H-NMR (400 MHz, [D_1]-chloroform, 298 K) δ [in ppm] = 7.47 (s, 4H, H-4), 3.95-3.93 (m, 4H, H-8), 3.82-3.80 (m, 4H, H-9), 3.74-3.61 (m, 16H, H-10, 11, 12, 13), 2.28 (s, 12H, H-6), 1.32 (s, 24H, H-1).

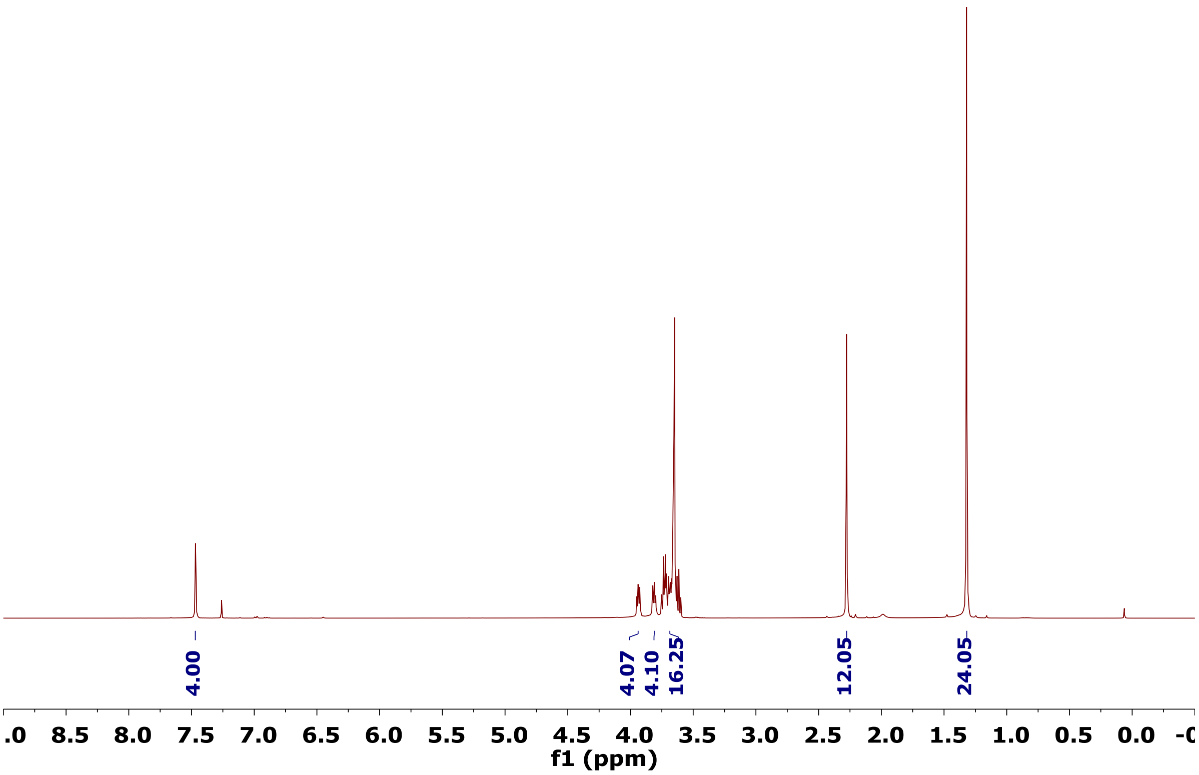

Figure S18: ^{1}H NMR spectrum of (*S*)-$\mathbf{7_6}$ ($CDCl_3$, 298 K, 400 MHz).

Compound 7_7

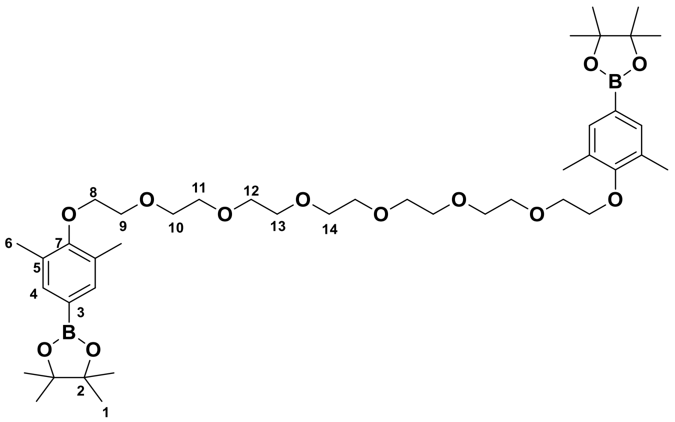

Compound $\mathbf{7_7}$ was prepared as described in section 2.2.3 using 2,6-dimethyl-4-(4,4,5,5-tetramethyl-1,3,2-dioxaborolan-2-yl)phenol (671 mg, 2.70 mmol, 2.0 equiv), cesium carbonate (1.32 g, 4.06 mmol, 3 equiv) and $\mathbf{8_7}$ (859 mg, 1.35 mmol, 1 equiv) dissolved in degassed acetonitrile (5 mL). After purification by column chromatography (cyclohexane/ethyl acetate 1:1) the product was obtained as a yellowish oil (786 mg, 999 µmol, 74.0% yield).

$C_{42}H_{68}O_{12}B_2$: 786.61 g/mol.

^{1}H-NMR (400 MHz, [D$_1$]-chloroform, 298 K) δ [in ppm] = 7.48 (s, 4H, H-4), 3.95 (t, *J* = 4.4 Hz, 4H, H-8), 3.81 (t, *J* = 4.4 Hz, 4H, H-9), 3.74-3.72 (m, 4H, H-10), 3.69-3.67 (m, 4H, H-11), 3.66-3.64 (m, 12H, H-12, 13, 14), 2.28 (s, 12H, H-6), 1.33 (s, 24H, H-1).

^{13}C-NMR (101 MHz, [D$_1$]-chloroform, 298 K) δ [in ppm] = 158.80 (C-7), 135.75 (C-4), 130.53 (C-5), 128.54 (C-3), 83.80 (C-2), 71.47 (C-8), 71.01 (C-10), 70.82, 70.74, 70.75 (C-11, 12, 13, 14), 70.66 (C-9), 24.99 (C-1), 16.22 (C-6).

^{1}H,^{1}H-COSY (400 MHz / 400 MHz, [D$_1$]-chloroform, 298 K) δ [in ppm] = 7.48/2.28 (H-4/H-6), 3.95/3.81 (H-8/H-9), 3.81/3.95 (H-9/H-8), 3.74-3.72/3.69-3.67 (H-10/H-11), 3.69-3.67/3.74-3.72 (H-11/H-10), 2.28/7.48 (H-6/H-4).

^{1}H,^{13}C-GHSQC (400 MHz / 101 MHz, [D$_1$]-chloroform, 298 K) δ (^{1}H) / δ (^{13}C) [in ppm] = 7.48/135.75 (H-4/C-4), 3.95/71.47 (H-8/C-8), 3.81/70.66 (H-9/C-9), 3.74-3.72/71.01 (H-10/C-10), 3.69-3.67/70.82 (H-11/C-11), 3.66-3.64/70.74, 70.75 (H-12, 13, 14/C-12, 13, 14), 2.28/16.22 (H-6/C-6), 1.33/24.99 (H-1/C-1).

^{1}H,^{13}C-GHMBC (400 MHz / 101 MHz, [D$_1$]-chloroform, 298 K) δ (^{1}H) / δ (^{13}C) [in ppm] = 7.48/158.80, 16.22 (H-4/C-7, 6), 2.28/ 158.80, 135.75, 130.53 (H-6/C-7, 4, 5), 1.33/83.80 (H-1/C-2).

MS (ESI-pos, MeOH): *m/z* = 809.4795 ([M+Na]$^+$, calcd. 809.4802 for [$C_{42}H_{68}O_{12}B_2$Na]$^+$).

IR (ATR-FT): $\tilde{\nu}$ (cm^{-1}) = 703, 731, 749, 764, 1142, 1263, 1276, 1368, 1988, 3005, 3054.

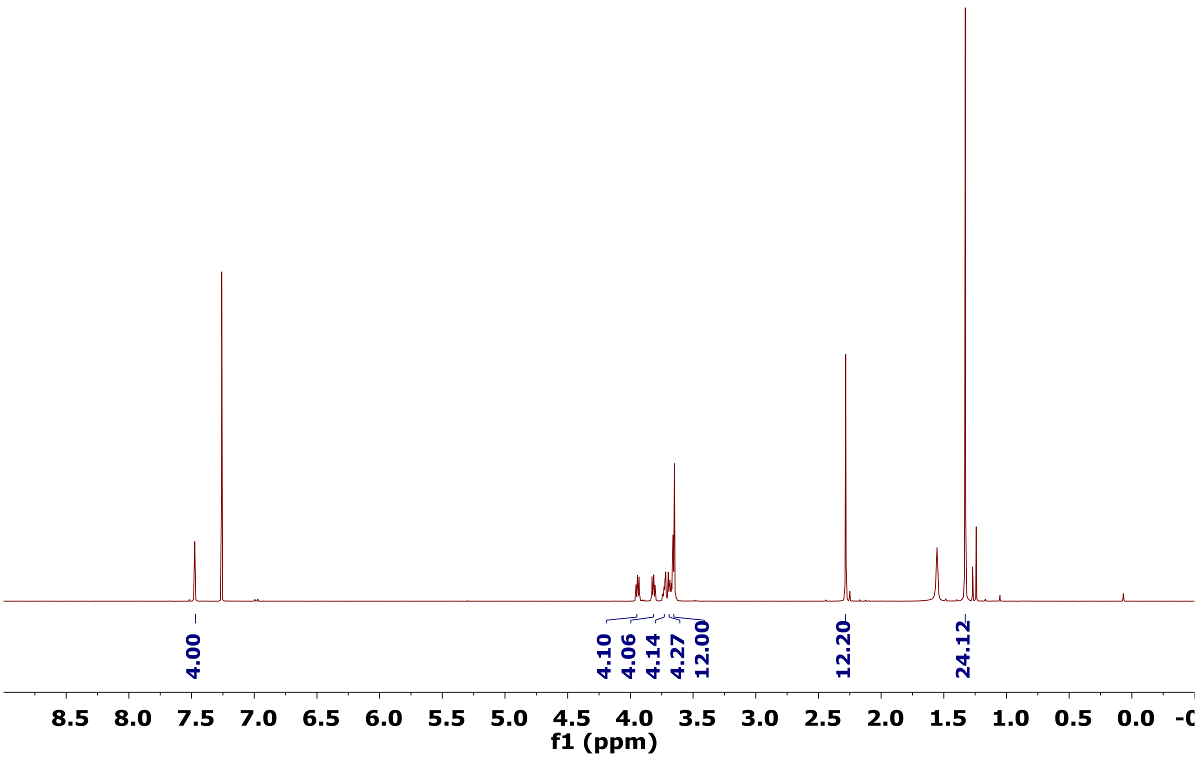

Figure S19: ^{1}H NMR spectrum of (*S*)-$\mathbf{7_7}$ ($CDCl_3$, 298 K, 400 MHz).

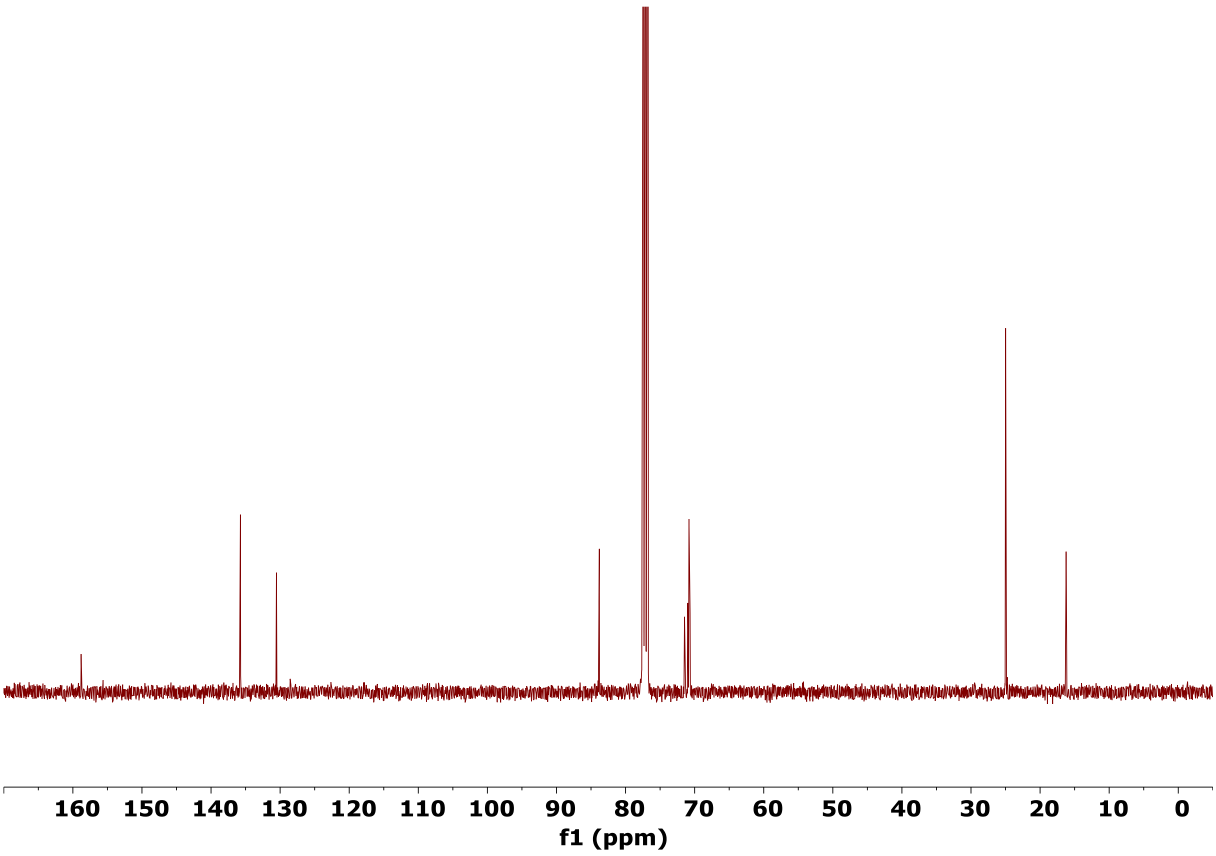

Figure S20: ^{13}C NMR spectrum of (*S*)-$\mathbf{7_7}$ ($CDCl_3$, 298 K, 400 MHz).

Compound 7_8

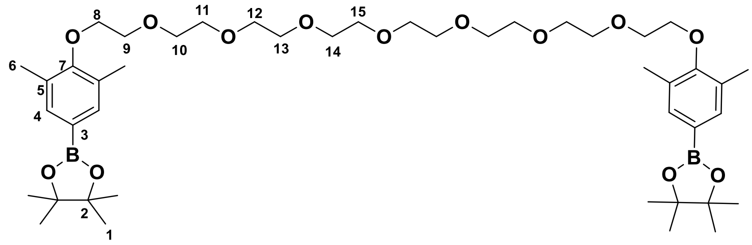

Compound $\mathbf{7_8}$ was prepared as described in section 2.2.3 using 2,6-dimethyl-4-(4,4,5,5-tetramethyl-1,3,2-dioxaborolan-2-yl)phenol (497 mg, 2.00 mmol, 2.0 equiv), cesium carbonate (988 mg, 3.03 mmol, 3 equiv) and $\mathbf{8_8}$ (690 mg, 1.02 mmol, 1 equiv) dissolved in degassed acetonitrile (4 mL). After purification by column chromatography (cyclohexane/ethyl acetate 2:3) the product was obtained as a light brown oil (570 mg, 686 µmol, 67.5% yield).

$C_{44}H_{72}O_{13}B_2$: 830.67 g/mol.

^{1}H-NMR (400 MHz, [D_1]-chloroform, 298 K) δ [in ppm] = 7.26 (s, 4H, H-4), 3.73 (t, *J* = 4.2 Hz, 4H, H-8), 3.59 (t, *J* = 4.2 Hz, 4H, H-9), 3.52-3.43 (m, 24H, H-10, 11, 12, 13, 14, 15), 2.07 (s, 12H, H-6), 1.11 (s, 24H, H-1).

^{13}C-NMR (400 MHz, [D_1]-chloroform, 298 K) δ [in ppm] = 158.71 (C-7), 135.68 (C-4), 130.44 (C-5), 128.84 (C-3), 83.71 (C-2), 71.40 (C-8), 71.44, 70.93, 70.74, 70.67, 70.65 (C-10, 11, 12, 13, 14, 15)*, 70.58 (C-9), 24.92 (C-1), 16.15 (C-6).

* Partially overlapping signals.

^{1}H,^{1}H-COSY (400 MHz / 400 MHz, [D_1]-chloroform, 298 K) δ [in ppm] = 7.26/2.07 (H-4/H-6), 2.07/7.26 (H-6/H-4).

^{1}H,^{13}C-GHSQC (400 MHz / 101 MHz, [D_1]-chloroform, 298 K) δ (^{1}H) / δ (^{13}C) [in ppm] = 7.26/135.68 (H-4/C-4), 3.73/71.40 (H-8/C-8), 3.59/70.58 (H-9/C-9), 3.52-3.43/71.44, 70.93, 70.74, 70.67, 70.65 (H-10, 11, 12, 13, 14, 15/C-10, 11, 12, 13, 14, 15), 2.07/16.15 (H-6/C-6), 1.11/24.92 (H-1/C-1).**^{1}H,^{13}C-GHMBC (400 MHz / 101 MHz, [D_1]-chloroform, 298 K) δ (^{1}H) / δ (^{13}C) [in ppm]** = 7.26/158.71, 16.15 (H-4/C-7, 6), 2.07/ 158.71, 135.68, 130.44 (H-6/C-7, 4, 5), 1.11/83.71 (H-1/C-2).

MS (ESI-pos, MeOH): *m/z* = 853.5063 ([M+Na]$^+$, calcd. 853.5066 for [$C_{44}H_{72}O_{13}B_2Na$]$^+$), *m/z* = 831.5239 ([M+H]$^+$, calcd. 831.5246 for [$C_{44}H_{72}O_{13}B_2H$]$^+$).

IR (ATR-FT): $\tilde{\nu}$ (cm^{-1}) = 702, 732, 768, 1265, 1275, 1367, 3005, 3054.

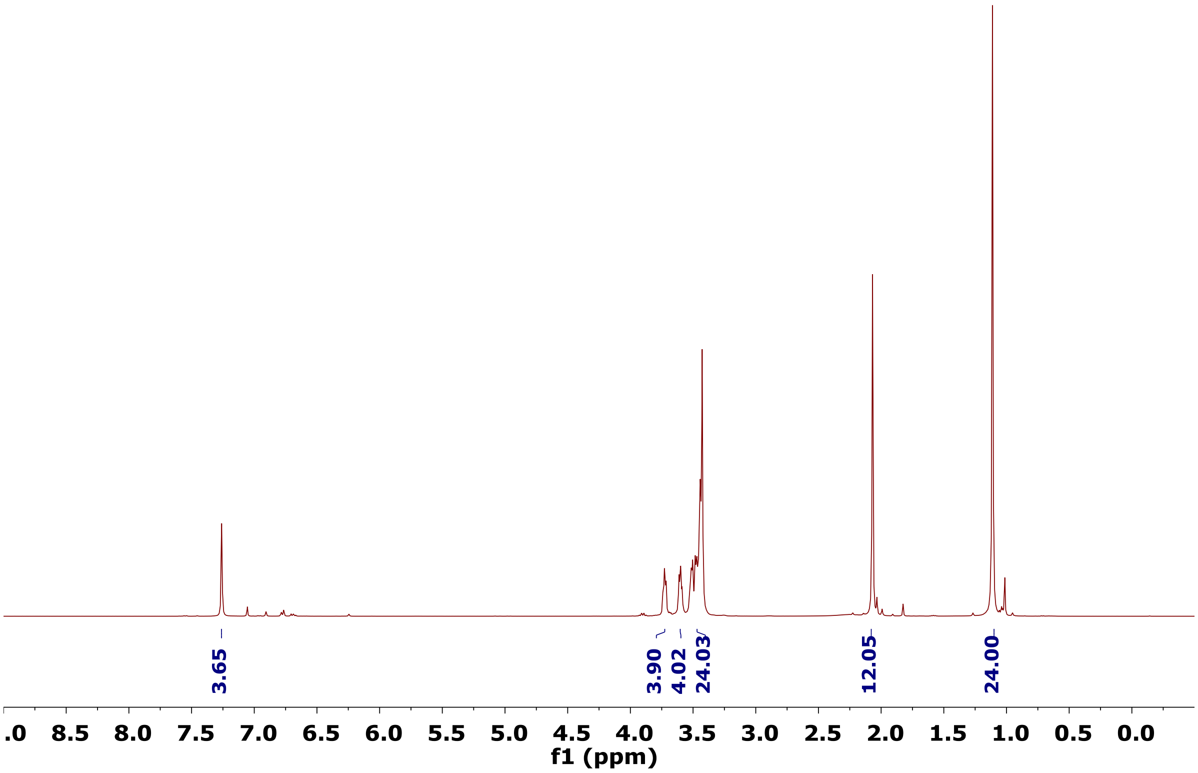

Figure S21: ^{1}H NMR spectrum of (*S*)-**7**$_{8}$ ($CDCl_3$, 298 K, 400 MHz).

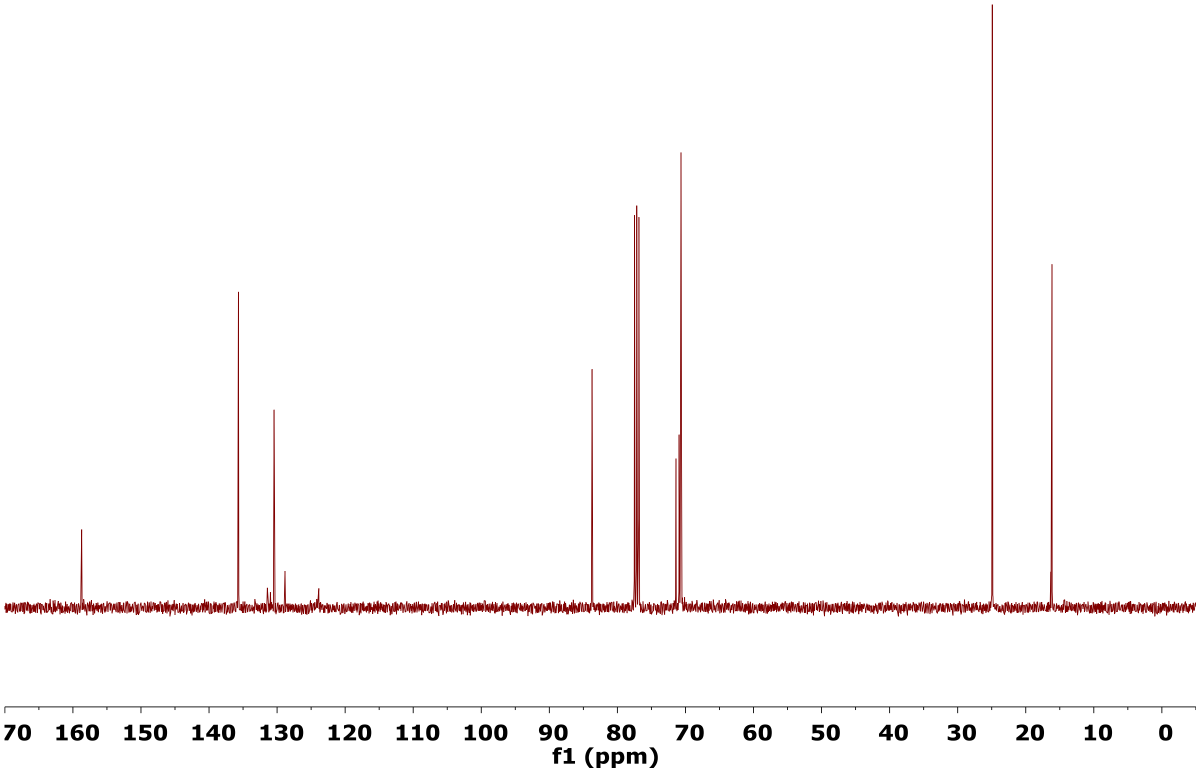

Figure S22: ^{13}C NMR spectrum of (*S*)-**7**$_{8}$ ($CDCl_3$, 298 K, 400 MHz).

2.2.4. Synthesis of compound **Me/H/iPr-9$_6$**

2,4-(**Me/H/iPr**)$_2$-4-(4,4,5,5-tetramethyl-1,3,2-dioxaborolan-2-yl)phenol (1 equiv) and cesium carbonate (2.1 equiv) were suspended in degassed acetonitrile and stirred for 15 minutes under argon. After the addition of the appropriate oligo-ethylene glycol bistosylate **8** (1 equiv) the reaction mixture was stirred at 85 °C for four hours. After cooling to room temperature, cesium carbonate was removed by filtration and all volatiles were removed in *vacuo*. The crude product was purified by column chromatography.

Compound **Me-9$_6$**

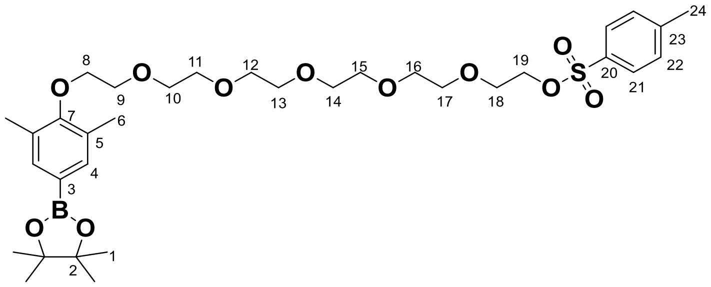

Compound **Me-9$_6$** was prepared as described in section 2.2.4 using 2,6-dimethyl-4-(4,4,5,5-tetramethyl-1,3,2-dioxaborolan-2-yl)phenol (690 mg, 2.78 mmol, 1 equiv), cesium carbonate (1.87 g, 5.74 mmol, 2.1 equiv) and **8$_6$** (1.64 g, 2.78 mmol, 1 equiv) dissolved in degassed acetonitrile (190 mL). After purification by column chromatography (cyclohexane/ethyl acetate 1:1) the product was obtained oil as a light brown oil (1.00 g, 1.50 mmol, 53.9% yield).

$C_{33}H_{51}O_{11}BS$: 666.63 g/mol.

^{1}H-NMR (400 MHz, [D$_1$]-chloroform, 298 K) δ [in ppm] = 7.79 (d, *J* = 8.3 Hz, 2H, H-21), 7.47 (s, 2H, H-4), 7.33 (d, *J* = 8.3 Hz, 2H, H-22), 4.15 (t, *J* = 4.6 Hz, 2H, H-8), 3.93 (t, *J* = 5.6 Hz, 2H, H-10), 3.81 (t, *J* = 5.5 Hz, 2H, H-11), 3.72-3.61 (m, 14H, H-9, 12, 15, 16, 17, 18, 19), 3.57 (bs, 4H, H-13, 14), 2.44 (s, 3H, H-24), 2.28 (s, 6H, H-6), 1.33 (s, 12H, H-1).

^{13}C-NMR (101 MHz, [D$_1$]-chloroform, 298 K) δ [in ppm] = 158.78 (C-7), 144.91 (C-23), 135.73 (C-4), 133.15 (C-20), 130.51 (C-5), 129.95 (C-22), 128.90 (C-3), 128.13 (C-21), 83.79 (C-2), 71.00, 70.88, 70.81, 70.75, 70.65 (C-9, 12, 15, 16, 17, 18, 19)*, 71.46 (C-10), 70.70 (C-11), 69.37 (C-8), 68.81 (C-13, 14), 24.98 (C-1), 21.78 (C-24), 16.20 (C-6).

* Partially overlapping signals.

^{1}H,^{1}H-COSY (400 MHz / 400 MHz, [D$_1$]-chloroform, 298 K) δ [in ppm] = 7.79/7.33 (H-21/H-22), 7.47/2.28 (H-4/H-6), 7.33/7.79 (H-22/H-21), 4.15/3.72-3.61 (H-8/H-9), 3.93/3.81 (H-10/H-11), 3.81/3.93 (H-11/H-10) 3.72-3.61/4.15 (H-9/H-8), 2.28/7.47 (H-6/H-4).

^{1}H,^{13}C-GHSQC (400 MHz / 101 MHz, [D$_1$]-chloroform, 298 K) δ (^{1}H) / δ (^{13}C) [in ppm] = 7.79/128.13 (H-21/C-21), 7.47/135.73 (H-4/C-4), 7.33/129.95 (H-22/C-22), 4.15/69.37 (H-8/C-8), 3.93/71.46 (H-10/C-10), 3.81/70.70 (H-11/C-11), 3.72-3.61/71.00, 70.88, 70.81, 70.75, 70.65 (H-9, 12, 15, 16, 17, 18, 19/C-9, 12, 15, 16, 17, 18, 19), 3.57/83.79 (H-13, 14/C-13, 14), 2.44/21.78 (H-24/C-24), 2.28/16.20 (H-6/C-6), 1.33/24.98 (H-1/C-1).

^{1}H,^{13}C-GHMBC (400 MHz / 101 MHz, [D$_1$]-chloroform, 298 K) δ (^{1}H) / δ (^{13}C) [in ppm] = 7.79/144.91 (H-21/C-23), 7.47/158.78, 16.20 (H-4/C-7, 6), 7.33/133.15, 21.78 (H-22/C-20, 24), 2.44/144.91, 129.95 (H-24/C-23, 22), 2.28/158.78, 135.73, 130.51 (H-6/C-7, 4, 5), 1.33/83.79 (H-1/C-2).

MS (ESI-pos, MeOH): *m/z* = 689.3137 ([M+Na]$^+$, calcd. 689.3143 [$C_{33}H_{51}O_{11}BSNa$]$^+$), *m/z* = 684.3585 ([M+NH$_4$]$^+$, calcd. 684.3589 [$C_{33}H_{51}O_{11}BSNH_4$]$^+$).

IR (ATR-FT): $\tilde{\nu}$ (cm^{-1}) = 555, 702, 731, 748, 764, 1098, 1143, 1177, 1264, 1275, 1368, 2809, 2988, 3005, 3053.

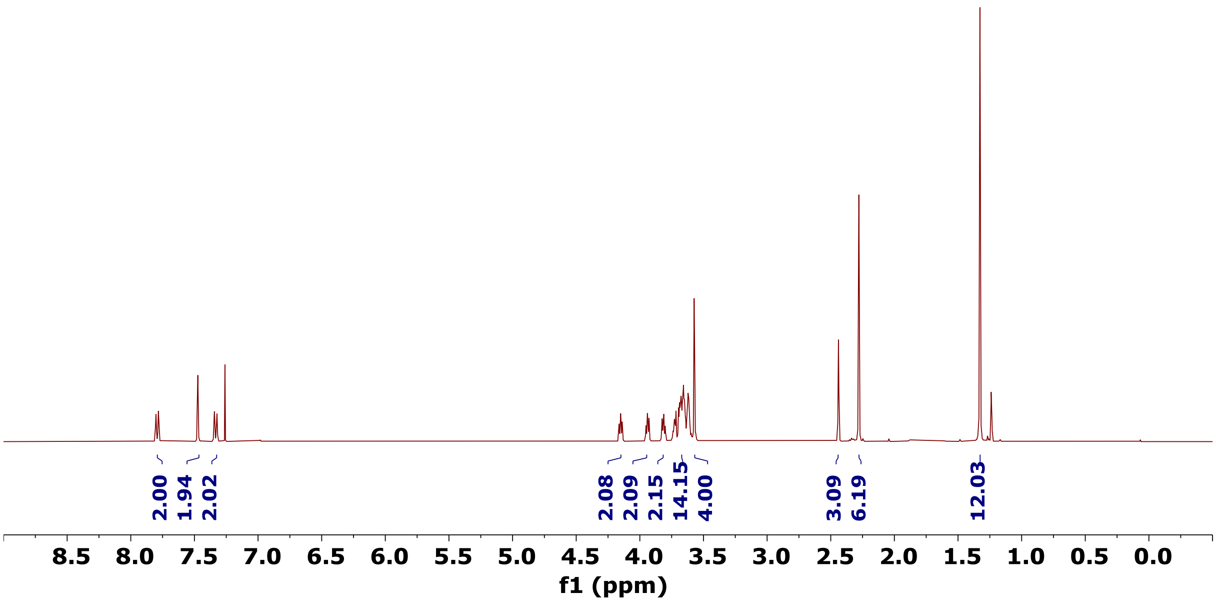

Figure S23: ^{1}H NMR spectrum of **Me-9₆** (CDCl₃, 298 K, 400 MHz).

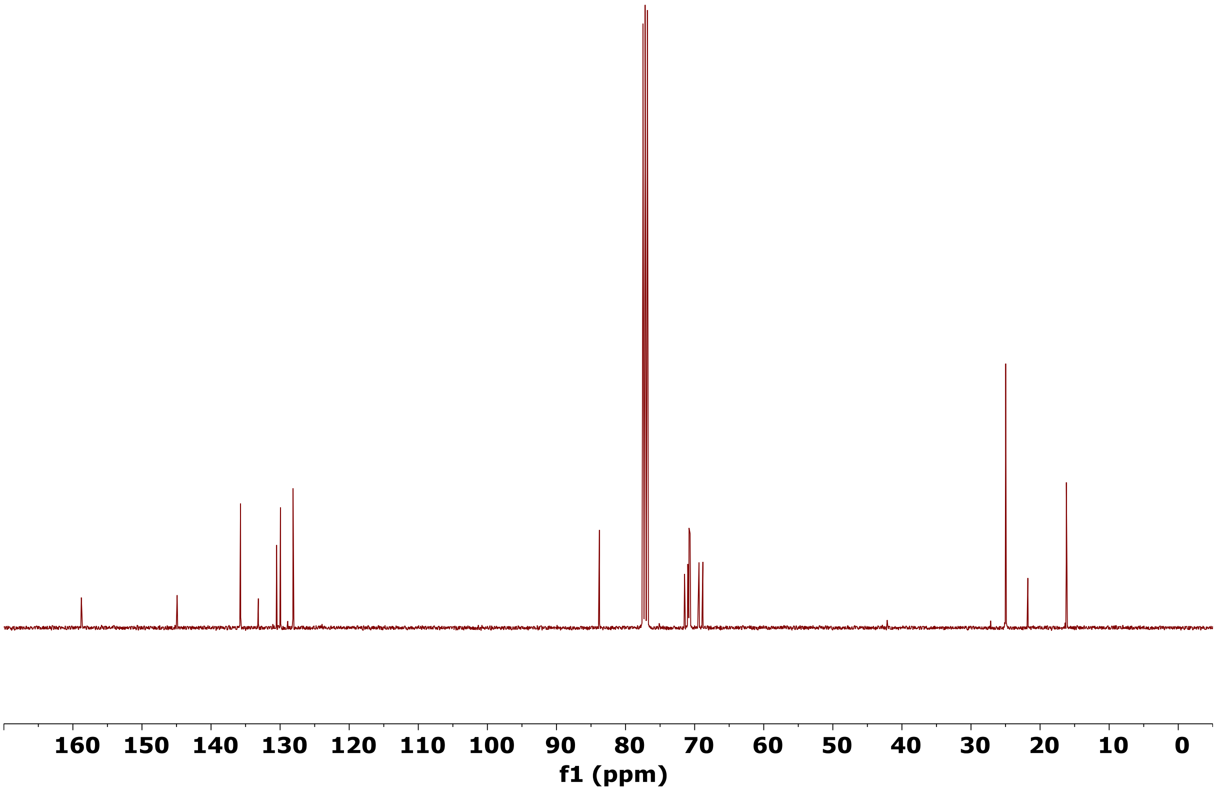

Figure S24: ^{13}C NMR spectrum of **Me-9₆** (CDCl₃, 298 K, 400 MHz).

Compound **H-9$_6$**

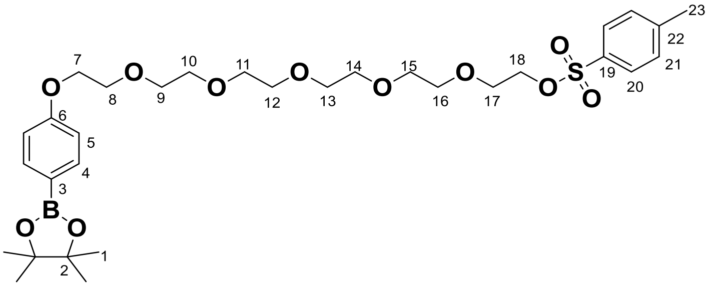

Compound **H-9$_6$** was prepared as described in section 2.2.4 using 4-(4,4,5,5-tetramethyl-1,3,2-dioxaborolan-2-yl)phenol (221 mg, 1.00 mmol, 1.0 equiv), cesium carbonate (740 mg, 2.27 mmol, 2.3 equiv) and **8$_6$** (730 mg, 1.24 mmol, 1.2 equiv) dissolved in degassed acetonitrile (85 mL). After purification by column chromatography (cyclohexane/ethyl acetate 1:1) the product was obtained as a clear oil (370 mg, 579 µmol, 57.9% yield).

$C_{31}H_{47}O_{11}BS$: 638.58 g/mol.

^{1}H-NMR (400 MHz, [D$_1$]-chloroform, 298 K) δ [in ppm] = 7.79 (d, *J* = 8.3 Hz, 2H, H-20), 7.72 (d, *J* = 8.6 Hz, 2H, H-4), 7.33 (d, *J* = 8.4 Hz, 2H, H-21), 6.89 (d, *J* = 8.6 Hz, 2H, H-5), 4.16-4.12 (m, 4H, H-7, 18), 3.86-3.84 (m, 2H, H-8), 3.73-3.69 (m, 2H, H-17), 3.68-3.57 (m, 16H, H-9, 10, 11, 12, 13, 14, 15, 16), 2.43 (s, 3H, H-23), 1.32 (s, 12H, H-1).

^{13}C-NMR (400 MHz, [D$_1$]-chloroform, 298 K) δ [in ppm] = 161.33 (C-6), 144.77 (C-22), 136.45 (C-4), 132.99 (C-19), 129.81 (C-21), 127.97 (C-20), 120.82 (C-3), 113.92 (C-5), 83.53 (C-2), 70.83, 70.72, 70.59, 70.55, 70.54, 70.49, 68.66 (H-9, 10, 11, 12, 13, 14, 15, 16, 17)*, 69.65 (C-8), 69.23 (C-7 or 18), 67.17 (C-7 or 18), 24.85 (C-1), 21.63 (C-23).

* Partially overlapping signals.

^{1}H,^{1}H-COSY (400 MHz / 400 MHz, [D$_1$]-chloroform, 298 K) δ [in ppm] = 7.79/7.33 (H-20/H-21), 7.72/6.89, 1.32 (H-4/H-5, 1), 7.33/7.79 (H-21/H-20), 6.89/7.72 (H-5/H-4), 4.16-4.12/3.86-3.84 (H-7, 18/H-8, 17), 3.86-3.84/4.16-4.12 (H-8, 17/H-7, 18).

^{1}H,^{13}C-GHSQC (400 MHz / 101 MHz, [D$_1$]-chloroform, 298 K) δ (^{1}H) / δ (^{13}C) [in ppm] = 7.79/127.97 (H-20/C-20), 7.72/136.45 (H-4/C-4), 7.33/129.81 (H-21/C-21), 6.89/113.92 (H-5/C-5), 4.16-4.12/69.23, 67.17 (H-7, 18/C-7, 18), 3.86-3.84/69.65 (H-8/C-8), 3.72-3.57/70.83, 70.72, 70.59, 70.55, 70.54, 70.49, 68.66 (H-9, 10, 11, 12, 13, 14, 15, 16, 17/C-9, 10, 11, 12, 13, 14, 15, 16, 17), 2.43/21.63 (H-23/C-23), 1.32/24.85 (H-1/C-1).

^{1}H,^{13}C-GHMBC (400 MHz / 101 MHz, [D$_1$]-chloroform, 298 K) δ (^{1}H) / δ (^{13}C) [in ppm] = 7.79/144.77 (H-20/C-22), 7.72/161.33 (H-4/C-6), 7.33/132.99, 21.63 (H-21/C-19, 23), 6.89/161.33, 120.82 (H-5/C-6, 3), 2.43/144.77, 129.81 (H-23/C-22, 21), 1.32/83.53 (H-1/C-2).

MS (ESI-pos, MeOH): *m/z* = 661.2833 ([M+Na]$^+$, calcd. 661.2830 for [$C_{31}H_{47}O_{11}BSNa$]$^+$).

IR (ATR-FT): $\tilde{\nu}$ (cm^{-1}) = 554, 663, 702, 732, 748, 764, 852, 1019, 1097, 1177, 1189, 1264, 1275, 1362, 2986, 3004, 3053.

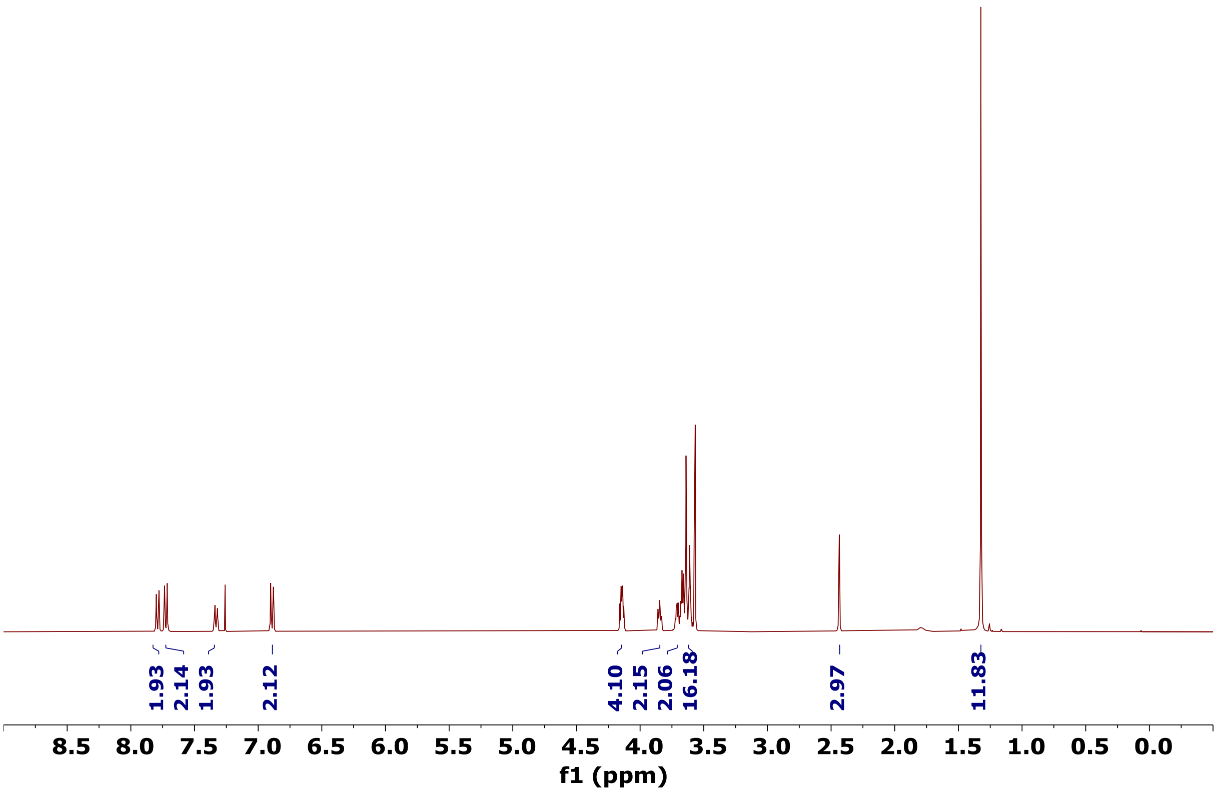

Figure S25: ^{1}H NMR spectrum of **H-9$_6$** ($CDCl_3$, 298 K, 400 MHz).

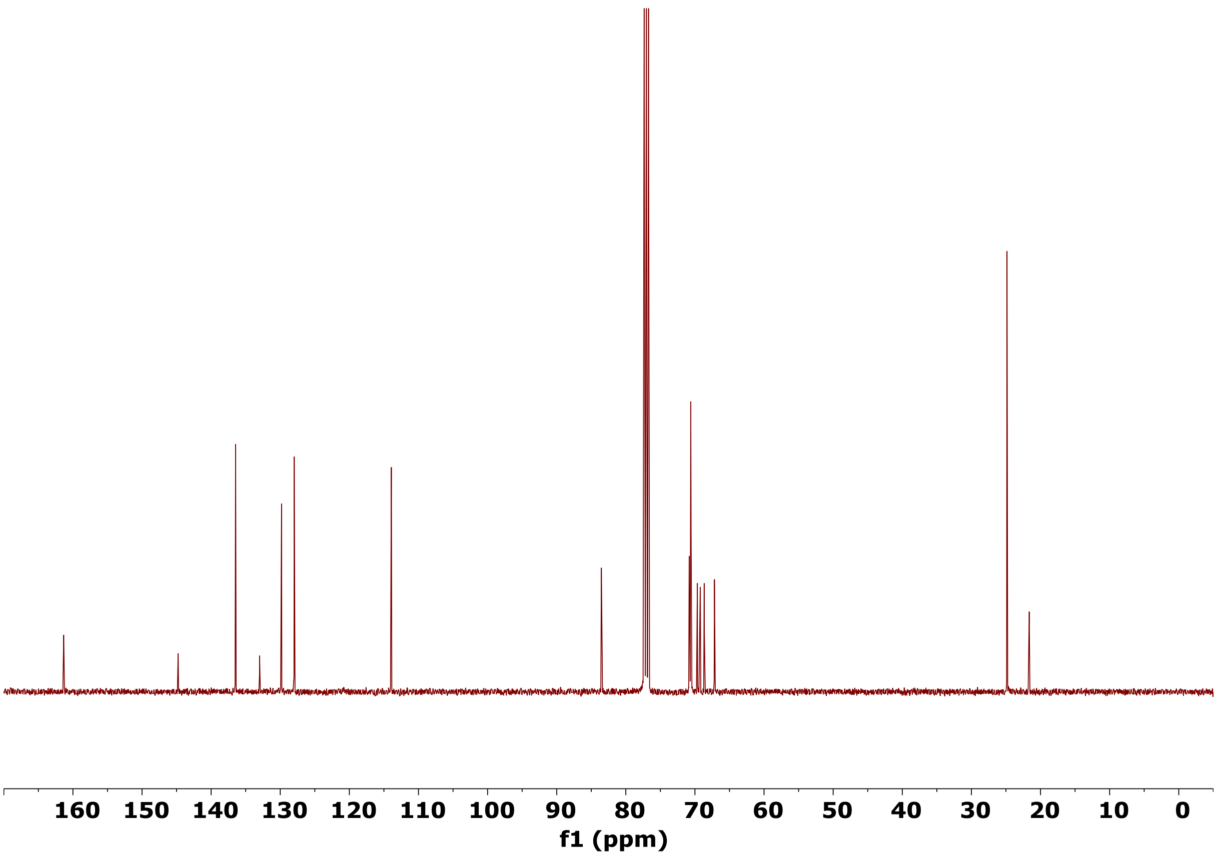

Figure S26: ^{13}C NMR spectrum of **H-9$_6$** ($CDCl_3$, 298 K, 400 MHz).

Compound **iPr-9**$_6$

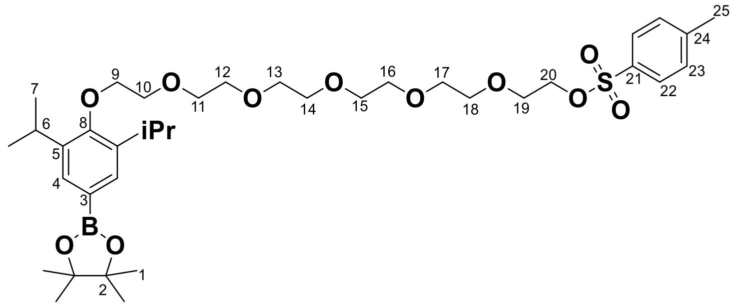

Compound iPr-**9**$_6$ was prepared as described in section 2.2.4 using 2,6-diisopropyl-4-(4,4,5,5-tetramethyl-1,3,2-dioxaborolan-2-yl)phenol (200 mg, 0.657 mmol, 1.0 equiv), cesium carbonate (429 mg, 1.32 mmol, 2.0 equiv) and **8**$_6$ (389 mg, 0.659 mmol, 1 equiv) dissolved in degassed acetonitrile (45 mL). After purification by column chromatography (cyclohexane/ethyl acetate 1:1) the product was obtained as a colorless oil (267 mg, 369 µmol, 56.2% yield).

$C_{37}H_{59}O_{11}BS$: 722.74 g/mol.

^{1}H-NMR (400 MHz, [D$_1$]-chloroform, 298 K) δ [in ppm] = 7.79 (d, *J* = 8.4 Hz, 2H, H-22), 7.56 (s, 2H, H-4), 7.33 (d, *J* = 8.4 Hz, 2H, H-23), 4.15 (t, 2H, H-9), 3.92-3.89 (m, 2H, H-10), 3.86-3.83 (m, 2H, H-19), 3.76-3.74 (m, 2H, H-20), 3.71-3.60 (m, 12H, H-11, 12, 13, 16, 17, 18), 3.76-3.74 (m, 2H, H-12), 3.58 (bs, 4H, H-14, 15), 3.37 (hept., *J* = 7.0 Hz, H-6), 2.44 (s, 3H, H-25), 1.33 (s, 12H, H-1), 1.24 (d, *J* = 7.0 Hz, 12H, H-7).

^{13}C-NMR (101 MHz, [D$_1$]-chloroform, 298 K) δ [in ppm] = 155.93 (C-8), 144.77 (C-24), 141.10 (C-5), 133.00 (C-21), 130.96 (C-4), 129.81 (C-23), 127.98 (C-22), 83.55 (C-2), 73.77 (C-10), 70.51 (C-19), 70.97 (C-20), 70.74, 70.69, 70.61, 70.55, 70.48, 68.67 (C-11, 12, 13, 16, 17, 18), 70.70, 70.69 (C-14, 15), 69.22 (C-9), 26.23 (C-6), 24.86 (C-1), 24.07 (C-7), 21.63 (C-25).

C-3 could not be detected.

^{1}H,^{1}H-COSY (400 MHz / 400 MHz, [D$_1$]-chloroform, 298 K) δ [in ppm] = 7.79/7.33 (H-22/H-23), 7.33/7.79 (H-23/H-22), 4.15/3.92-3.89 (H-9/H-10), 3.92-3.89/4.15 (H-10/H-9), 3.86-3.83/3.76-3.74 (H-20/H-19), 3.76-3.74/3.86-3.83 (H-19/H-20), 3.37/1.24 (H-6/H-7), 1.24/3.37 (H-7/H-6).

^{1}H,^{13}C-GHSQC (400 MHz / 101 MHz, [D$_1$]-chloroform, 298 K) δ (^{1}H) / δ (^{13}C) [in ppm] = 7.79/127.98 (H-22/C-22), 7.56/130.96 (H-4/C-4), 7.33/129.81 (H-23/C-23), 4.15/69.22 (H-9/C-9), 3.92-3.89/73.77 (H-10/C-10), 3.86-3.83/70.51 (H-19/C-19), 3.76-3.74/70.97 (H-20/C-20), 3.71-3.60/70.74, 70.69, 70.61, 70.55, 70.48, 68.67 (H-11, 12, 13, 16, 17, 18/C-11, 12, 13, 16, 17, 18), 3.58/70.70, 70.69 (H-14, 15/C-14, 15), 3.37/26.23 (H-6/C-6), 2.44/21.63 (H-25/C-25), 1.33/24.86 (H-1/C-1), 1.24/24.07 (H-7/C-7).

^{1}H,^{13}C-GHMBC (400 MHz / 101 MHz, [D$_1$]-chloroform, 298 K) δ (^{1}H) / δ (^{13}C) [in ppm] = 7.79/144.77 (H-22/C-24), 7.56/155.93, 26.23 (H-4/C-8, 6), 7.33/133.00, 21.63 (H-23/C-21, 25), 3.37/155.93, 141.10, 130.96, 24.07 (H-6/C-8, 5, 4, 7), 2.44/144.77, 129.81 (H-25/C-24, 23), 1.33/83.55 (H-1/C-2), 1.24/141.10 (H-7/C-5).

MS (ESI-pos, MeOH): *m/z* = 745.3779 ([M+Na]$^+$, calcd. 745.3770 [$C_{37}H_{59}O_{11}BSNa$]$^+$), *m/z* = 740.4224 ([M+NH$_4$]$^+$, calcd. 740.4216 [$C_{37}H_{59}O_{11}BSNH_4$]$^+$).

IR (ATR-FT): $\tilde{\nu}$ (cm^{-1}) = 557, 663, 702, 732, 748, 764, 1097, 1142, 1177, 1189, 1200, 1263, 1275, 1363, 2986, 3004, 3052.

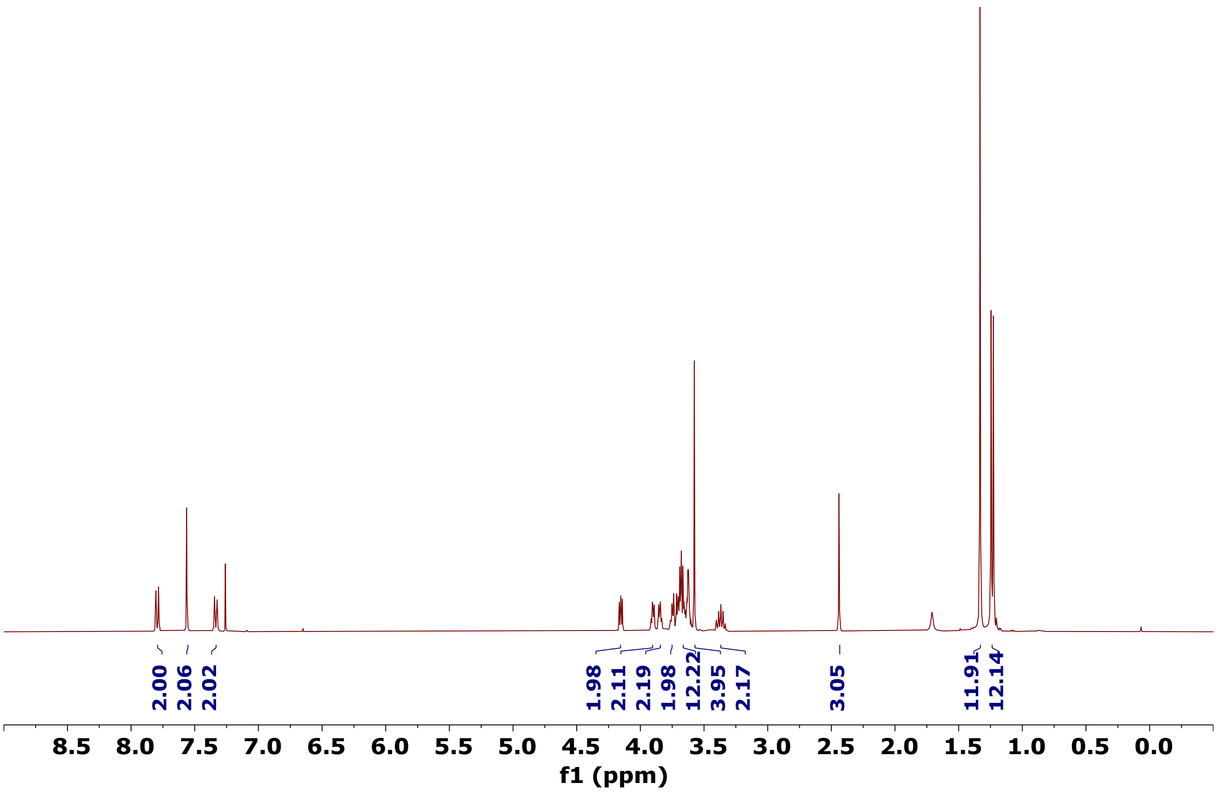

Figure S27: ^{1}H NMR spectrum of **iPr-9$_6$** ($CDCl_3$, 298 K, 400 MHz).

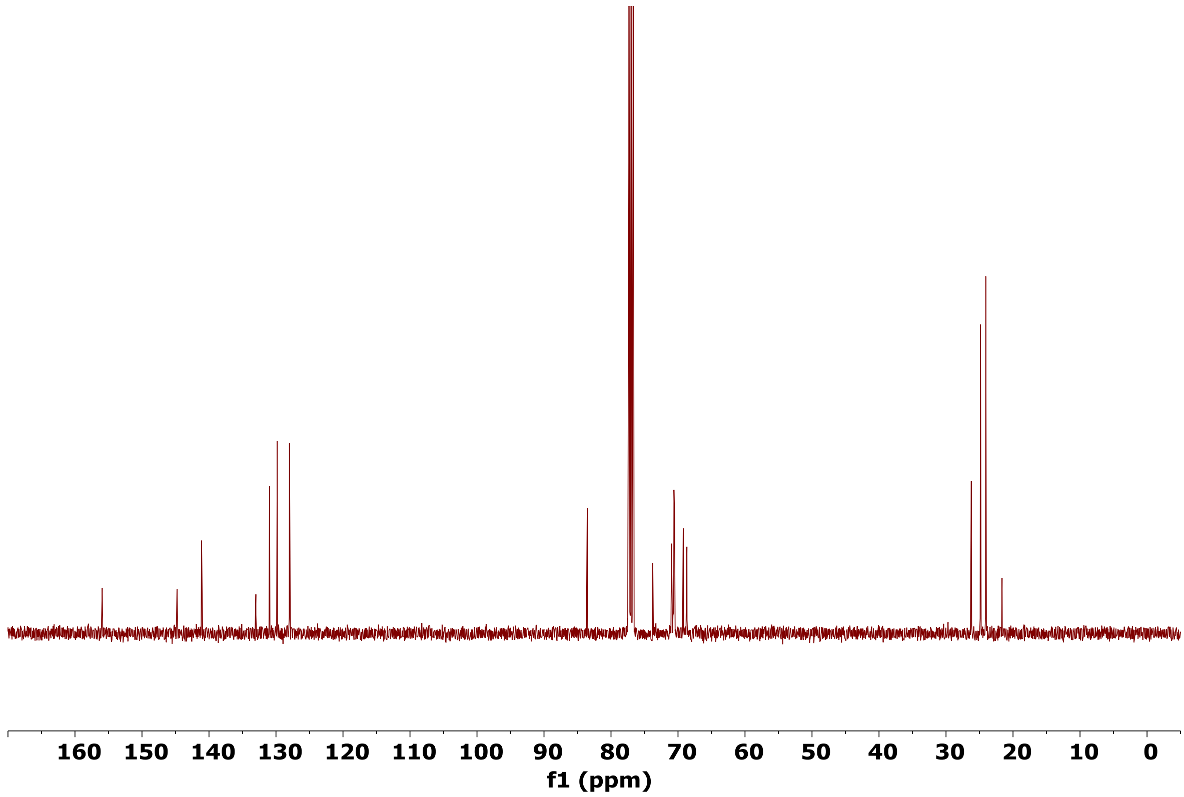

Figure S28: ^{13}C NMR spectrum of **iPr-9$_6$** ($CDCl_3$, 298 K, 400 MHz).

2.3. General procedure for the synthesis of macrocycles

Synthetic routes towards macrocycles featuring one BINOL unit (Me/H/iPr-M1$_{5/6/7/8}$)

2.3.1. Two-fold Suzuki coupling (General procedure A)

Compound (*S*)-**1** (1 equiv), the related boronic ester (1 equiv), tetrabutylammonium hydroxide 30 hydrate (3.2 equiv), and tri(*o*-tolyl)phosphine (0.2 equiv) were dissolved in a degassed solution of toluene and water 5:1 (v/v). Tris(dibenzylideneacetone)dipalladium(0) (0.1 equiv) was added and the reaction mixture was refluxed for 12 hours. After cooling to room temperature, the reaction mixture was diluted with water (100 mL/mmol of (*S*)-**1**) and ethyl acetate (100 mL/mmol of (*S*)-**1**). The aqueous phase was extracted two times with ethyl acetate (100 mL/mmol of(*S*)-**1**) and the combined organic layer was washed with a saturated solution of sodium chloride (100 mL/mmol of (*S*)-**1**) once. The organic layer was dried over sodium sulfate and concentrated in *vacuo*. The crude product was purified by column chromatography.

2.3.2. Two-fold Williamson synthesis (General procedure B)

Compound **Me/H/iPr-2** (1 equiv) and cesium carbonate (2 equiv), were dissolved in degassed acetonitrile and stirred for 15 minutes. After the addition of corresponding bistosylate **8**$_{5/6/7/8}$ (1 equiv) the reaction mixture was stirred at 85 °C overnight. After cooling to room temperature, ethyl acetate (10 mL/0.5 mmol of **Me/H/iPr-2**) was added. Cesium carbonate was removed by filtration and all volatiles were removed in *vacuo.* The crude product was purified by column chromatography.

Synthetic routes towards macrocycles featuring two BINOL units

2.3.3. Macrocycles M2$_6$ (General procedure C)

Compound **Me/H/iPr-2** (1 equiv) and cesium carbonate (3.2 equiv), were dissolved in degassed acetonitrile and stirred for 15 minutes. After the addition of corresponding bistosylate **Me/H/iPr-3**$_6$ (1 equiv) the reaction mixture was stirred at 85 °C for six hours. After cooling to room temperature, cesium carbonate was removed by filtration and all volatiles were removed in *vacuo.* The crude product was purified by column chromatography.

2.3.4. Macrocycles M2$_2$ (General procedure D)

Compound **Me/H/iPr-2** (1 equiv) and cesium carbonate (3.2 equiv), were dissolved in degassed acetonitrile and stirred for 15 minutes. After the addition of corresponding dichloride **Me/H/iPr-6**$_2$ (1 equiv) the reaction mixture was stirred at 85 °C for six hours. If incomplete conversion of **Me/H/iPr-2** was observed by TLC, additional cesium carbonate (1.2 equiv) was added and the mixture was stirred for additional twelve hours under reflux. After cooling to room temperature, cesium carbonate was removed by filtration and all volatiles were removed in *vacuo.* The crude product was purified by column chromatography.

2.4. Mono-BINOL macrocycles

Compound **Me-M1$_5$**

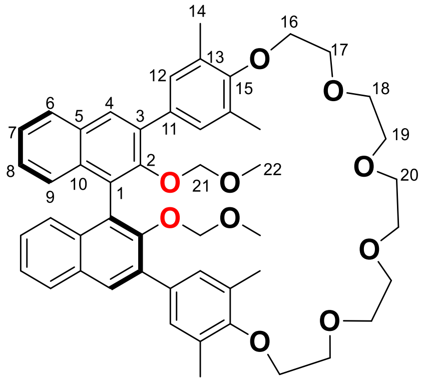

As described above (General procedure **B**), compound **Me-M1$_5$** was synthesized using **Me-2** (35.0 mg, 0.0569 mmol, 1.0 equiv), cesium carbonate (45.9 mg, 0.141 mmol, 2.5 equiv) and **8$_5$** (31.2 mg, 0.0571 mmol, 1.0 equiv) dissolved in degassed acetonitrile (57 mL). After purification by column chromatography (cyclohexane/ethyl acetate 3.2) the product was obtained as a colorless oil (10.2 mg, 0.0124 mmol, 21.1% yield).

$C_{50}H_{56}O_{10}$: 816.99 g/mol

^{1}H-NMR (400 MHz, [D$_1$]-chloroform, 298 K) δ [in ppm] = 7.92 (s, 2H, H-4), 7.88 (d, *J* = 8.2 Hz, 2H, H-6), 7.44 (d, *J* = 7.9 Hz, 2H, H-9), 7.41 (dd, *J* = 1.3, *J* = 6.8 Hz, 2H, H-7), 7.30 (dd, *J* = 1.4, *J* = 6.9 Hz, 2H, H-8), 7.29 (s, 4H, H-12), 4.33 (d, *J* = 5.8 Hz, 2H, H-21$_{1/2}$), 4.20 (d, *J* = 5.8 Hz, 2H, H-21$_{1/2}$), 4.25-4.21 (m, 2H, H-16$_{1/2}$), 4.08-4.03 (m, 2H, H-16$_{1/2}$), 3.77-3.70 (m, 2H, H-17$_{1/2}$), 3.64-3.60 (m, 2H, H-17$_{1/2}$), 3.59-3.46 (m, 12H, H-18, 19, 20), 2.46 (s, 6H, H-22), 2.37 (s, 12H, H-14).

^{13}C-NMR (101 MHz, [D$_1$]-chloroform, 298 K) δ [in ppm] = 155.79 (C-15), 152.54 (C-2), 135.66 (C-3), 133.90 (C-11), 133.18 (C-10), 130.75 (C-5), 130.42 (C-13), 129.83 (C-12), 129.45 (C-4), 127.99 (C-6), 126.24 (C-1), 126.15 (C-9), 125.76 (C-8), 124.88 (C-7), 98.71 (C-21), 72.11 (C-16), 71.17 (C-17), 70.47, 70.26, 70.18 (C-18, 19, 20), 55.95 (C-22), 16.91 (C-14).

^{1}H,^{1}H-COSY (400 MHz / 400 MHz, [D$_1$]-chloroform, 298 K) δ [in ppm] = 7.88/7.41 (H-6/H-7), 7.44/7.30 (H-9/H-8), 7.41/7.88, 7.30 (H-7/H-6, 8), 7.30/7.44, 7.41 (H-8/H-9, 7), 7.29/2.37 (H-12/H-14), 4.33/4.20 (H-21$_{1/2}$/H-21$_{1/2}$), 4.25-4.21/4.08-4.03, 3.64-3.60 (H-16$_{1/2}$/H-16$_{1/2}$, 17$_{1/2}$), 4.08-4.03/4.25-4.21, 3.77-3.70 (H-16$_{1/2}$/H-16$_{1/2}$, 17$_{1/2}$), 3.77-3.70/4.08-4.03, 3.64-3.60 (H-17$_{1/2}$/H-16$_{1/2}$, 17$_{1/2}$), 3.64-3.60/4.25-4.21, 3.77-3.70 (H-17$_{1/2}$/H-16$_{1/2}$, 17$_{1/2}$), 4.20/4.33 (H-21$_{1/2}$/H-21$_{1/2}$).

^{1}H,^{13}C-GHSQC (400 MHz / 101 MHz, [D$_1$]-chloroform, 298 K) δ (^{1}H) / δ (^{13}C) [in ppm] = 7.92/129.45 (H-4/C-4), 7.88/127.99 (H-6/C-6), 7.44/126.15 (H-9/C-9), 7.41/124.88 (H-7/C-7), 7.30/125.76 (H-8/C-8), 7.29/129.83 (H-12/C-12), 4.33/98.71 (H-21$_{1/2}$/C-21), 4.20/98.71 (H-21$_{1/2}$/C-21), 4.25-4.21/72.11 (H-16$_{1/2}$/ C-16,), 4.08-4.03/72.11 (H-16$_{1/2}$/ C-16), 3.77-3.70/71.17 (H-17$_{1/2}$/C-17), 3.64-3.60/71.17 (H-17$_{1/2}$/ C-17), 3.59-3.46/70.47, 70.26, 70.18 (H-18, 19, 20/C-18, 19, 20), 2.46/55.95 (H-22, C-22), 2.37/16.91 (H-14/C-14).

^{1}H,^{13}C-GHMBC (400 MHz / 101 MHz, [D$_1$]-chloroform, 298 K) δ (^{1}H) / δ (^{13}C) [in ppm] = 7.92/152.54, 133.90, 133.18, 127.99 (H-4/C-2, 11, 10, 6), 7.88/133.18, 129.45, 125.76 (H-6/C-10, 4, 8), 7.44/130.75, 126.24, 124.88 (H-9/C-5, 1, 7), 7.41/130.75, 126.15 (H-7/C-5, 9), 7.30/133.18, 127.99 (H-8/C-10, 6),

7.29/155.79, 135.66, 16.91 (H-12/C-15, 3, 14), 4.33/55.95 (H-$21_{1/2}$/C-22), 4.20/55.95 (H-$21_{1/2}$/C-22), 2.46/98.71 (H-22/C-21), 2.37/155.79, 130.42, 129.83 (H-14/C-15, 13, 12).

MS (ESI-pos, MeOH): *m/z* = 839.3759 ($[M+Na]^+$, calcd. 839.3766 for $[C_{50}H_{56}O_{10}Na]^+$), *m/z* = 834.4204 ($[M+NH_4]^+$, calcd. 834.4212 for $[C_{50}H_{56}O_{10}NH_4]^+$).

IR (ATR-FT): $\tilde{\nu}$ (cm^{-1}) = 702, 732, 748, 764, 881, 993, 1147, 1264, 1275, 2988, 3002, 3054.

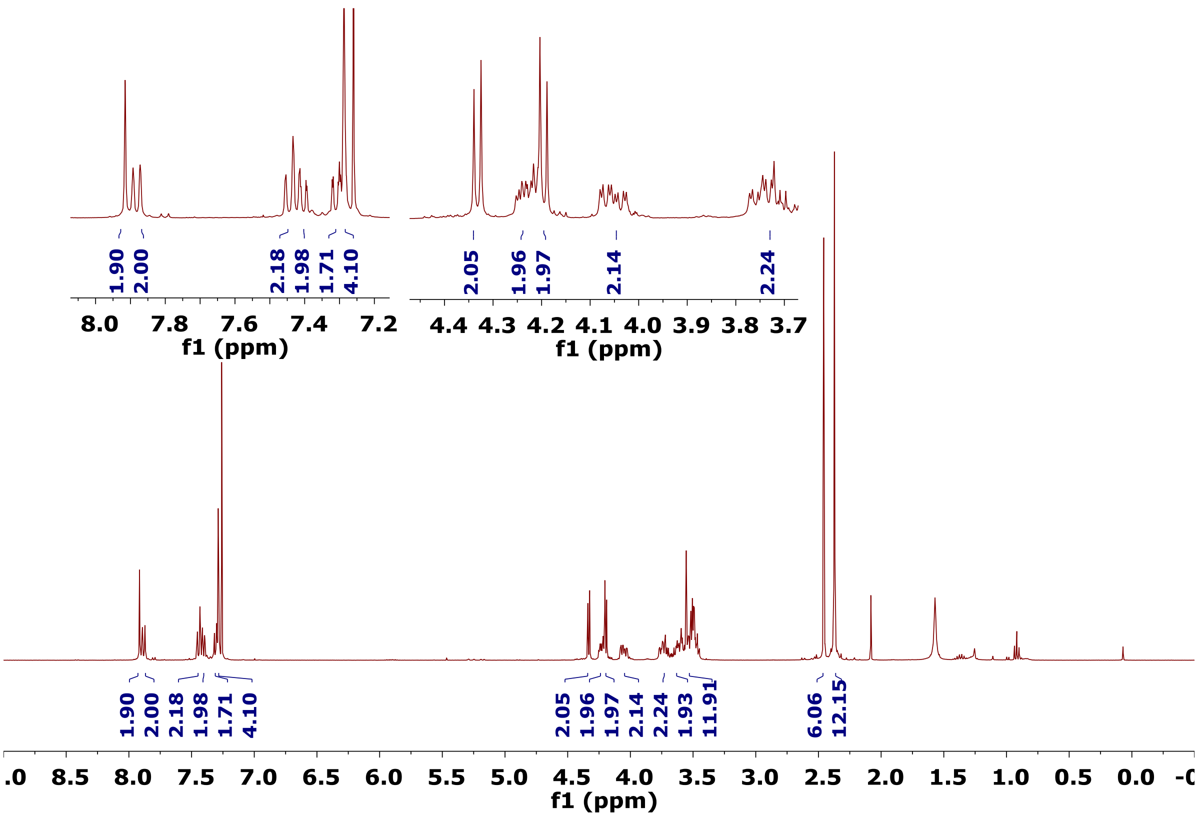

Figure S29: ^{1}H NMR spectrum of (*S*)- **Me-M1**$_5$ ($CDCl_3$, 298 K, 400 MHz).

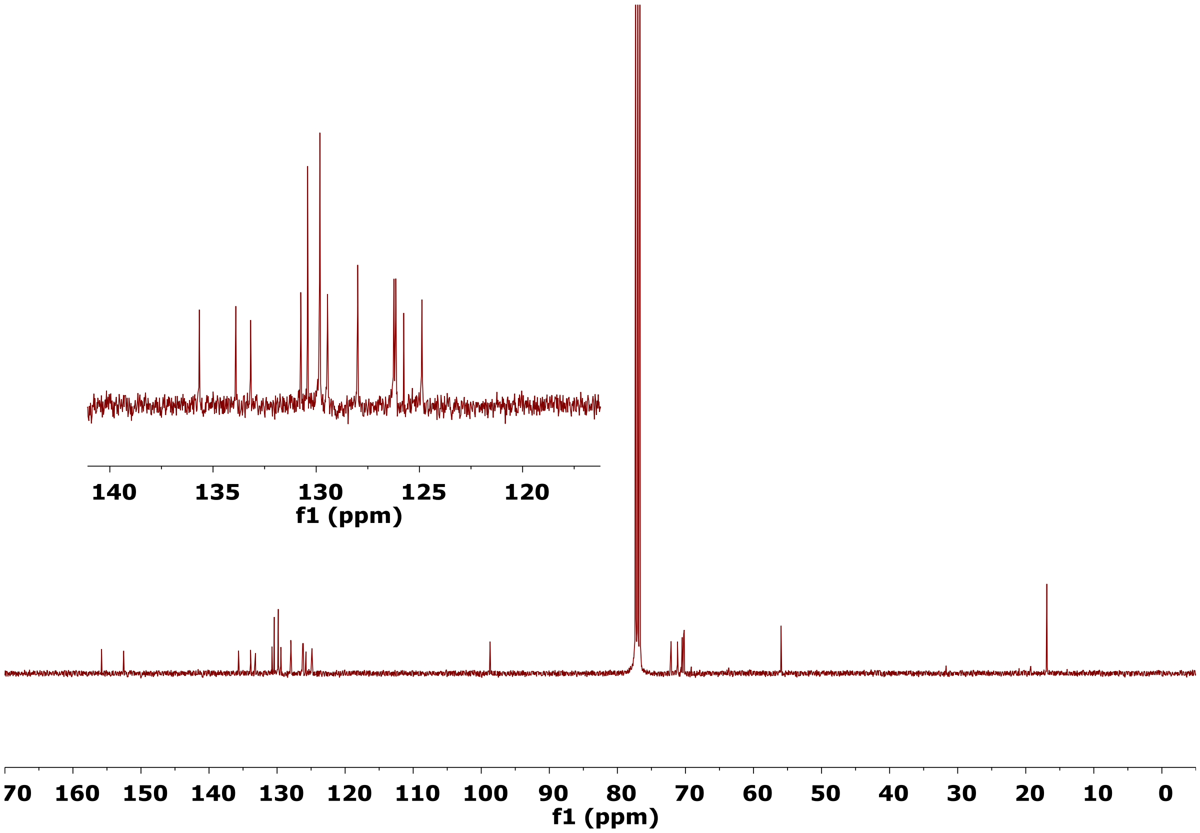

Figure S30: ^{13}C NMR spectrum of (*S*)- **Me-M1**$_5$ ($CDCl_3$, 298 K, 400 MHz).

Compound **Me-M1$_6$**

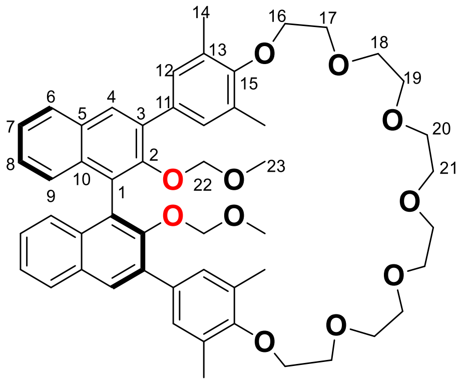

As described above (General procedure **B**), compound **Me-M1$_6$** was synthesized using **Me-2** (24.9 mg, 0.0405 mmol, 1.0 equiv), cesium carbonate (34.2mg, 104 µmol, 2.5 equiv) and **8$_6$** (23.9 mg, 0.0405 mmol, 1.0 equiv) dissolved in degassed acetonitrile (40 mL). After purification by column chromatography (methanol/dichloromethane 3:97) the product was obtained as white solid (19.1 mg, 0.0222 mmol, 53.7% yield).

$C_{52}H_{60}O_{11}$: 861.04 g/mol.

^{1}H-NMR (400 MHz, [D1]-chloroform, 298 K) δ [in ppm] = 7.78 (s, 2H, H-4), 7.73 (d, *J* = 8.2 Hz, 2H, H-6), 7.28-7.24 (m, 2H, H-7, 12), 7.18 (m, 2H, H-9), 7.13-7.10 (m, 2H, H-8), 4.23 (d, *J* = 6.0 Hz, 2H, H-22$_{1/2}$), 4.16 (d, *J* = 6.0 Hz, 2H, H-22$_{1/2}$), 3.98-3.85 (m, 4H, H-16), 3.65-3.61 (m, 4H, H-17), 3.57-3.50 (m, 16H, H-18, 19, 20, 21), 2.27 (s, 12H, H-14), 2.19 (s, 6H, H-23).

^{13}C-NMR (101 MHz, [D1]-chloroform, 298 K) δ [in ppm] = 155.14 (C-15), 151.83 (C-2), 135.18 (C-3), 134.23 (C-11), 133.20 (C-10), 130.68 (C-5), 129.84 (C-7), 129.81 (C-4), 127.70 (C-6), 126.26 (C-9), 126.13 (C-1), 125.93 (C-8), 124.79 (C-12), 98.53 (C-22), 71.82 (C-16), 70.77, 70.64, 70.41, 70.33 (C-18, 19, 20, 21), 70.27 (C-17), 55.68 (C-23), 16.46 (C-14).

^{1}H,^{1}H-COSY (400 MHz / 400 MHz, [D1]-chloroform, 298 K) δ [in ppm] = 7.73/7.28-7.24 (H-6/H-7, 12), 7.28-7.24/7.73, 7.13-7.10, 2.27 (H-7, 12/H-6, 8, 14), 7.18/7.13-7.10 (H-9/H-8), 4.23/4.16 (H-22$_{1/2}$/ H-22$_{1/2}$), 4.16/4.23 (H-22$_{1/2}$/ H-22$_{1/2}$), 3.98-3.85/3.65-3.61 (H-16/H-17), 3.65-3.61/3.98-3.85 (H-17/H-16), 2.27/7.28-7.24 (H-14/H-7, 12).

^{1}H,^{13}C-GHSQC (400 MHz / 101 MHz, [D1]-chloroform, 298 K) δ (^{1}H) / δ (^{13}C) [in ppm] = 7.78/129.81 (H-4/C-4), 7.73/127.70 (H-6/C-6), 7.28-7.24/129.84 (H-7/C-7), 7.28-7.24/ 124.79 (H-12/C-12), 7.18/126.26 (H-9/C-9), 7.13-7.10/125.93 (H-8/C-8), 4.23/98.53 (H-22$_{1/2}$/C-22), 4.16/98.53 (H-22$_{1/2}$/C-22), 3.98-3.85/71.82 (H-16/C-16), 3.65-3.61/70.27 (H-17/C-17), 3.57-3.50/70.77, 70.64, 70.41, 70.33 (H-18, 19, 20, 21/C-18, 19, 20, 21), 2.27/16.46 (H-14/C-14), 2.19/55.68 (H-23/C-23).

^{1}H,^{13}C-GHMBC (400 MHz / 101 MHz, [D1]-chloroform, 298 K) δ (^{1}H) / δ (^{13}C) [in ppm] = 7.78/151.83, 134.23, 133.20 , 127.70 (H-4/ C-2, 11, 10, 6), 7.73/133.20, 129.81, 125.93 (H-6/C-10, 4, 8), 7.28-7.24/155.14, 135.18, 130.68, 126.26, 16.46 (H-7, 12/C-15, 3, 5, 9, 14), 7.18/130.68, 129.84, 126.13 (H-9/C-5, 7, 1), 7.13-7.10/133.20, 127.70 (H-8/C-10, 6), 4.23/55.68 (H-22$_{1/2}$/C-23), 4.16/55.68 (H-22$_{1/2}$/C-23), 2.27/155.14, 124.79 (H-14/C-15, 12), 2.19/98.53 (H-23/C-22).

MS (ESI-pos, MeOH): *m/z* = 883.4028 ([M+Na]$^+$, calcd. 883.4028 for [$C_{52}H_{60}O_{11}Na$]$^+$); *m/z* = 878.4471 ([M+NH$_4$]$^+$, calcd. 878.4474 for [$C_{52}H_{60}O_{11}NH_4$]$^+$).

IR (ATR-FT): $\tilde{\nu}$ (cm^{-1}) = 750, 764, 1147, 1260, 1267, 1275, 2921, 2996, 3006.

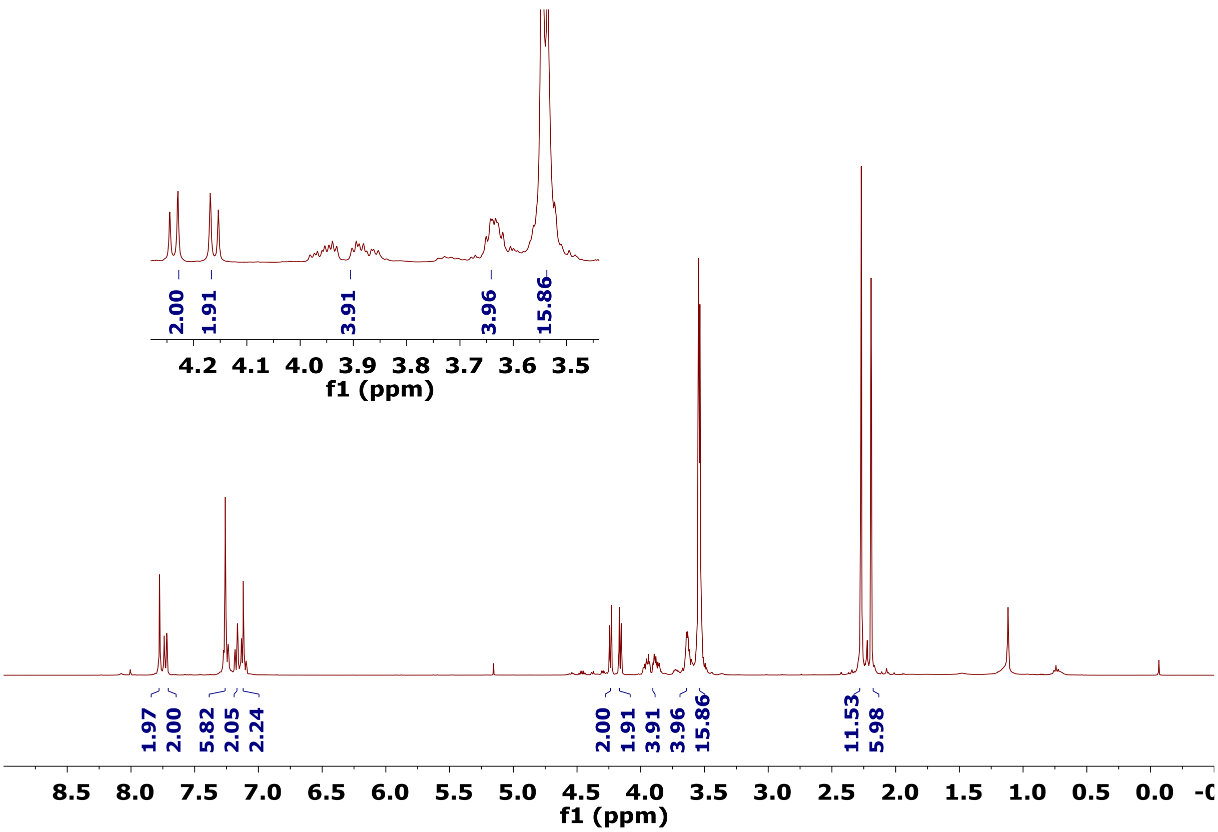

Figure S31: ^{1}H NMR spectrum of (*S*)- **Me-M1$_6$** ($CDCl_3$, 298 K, 400 MHz).

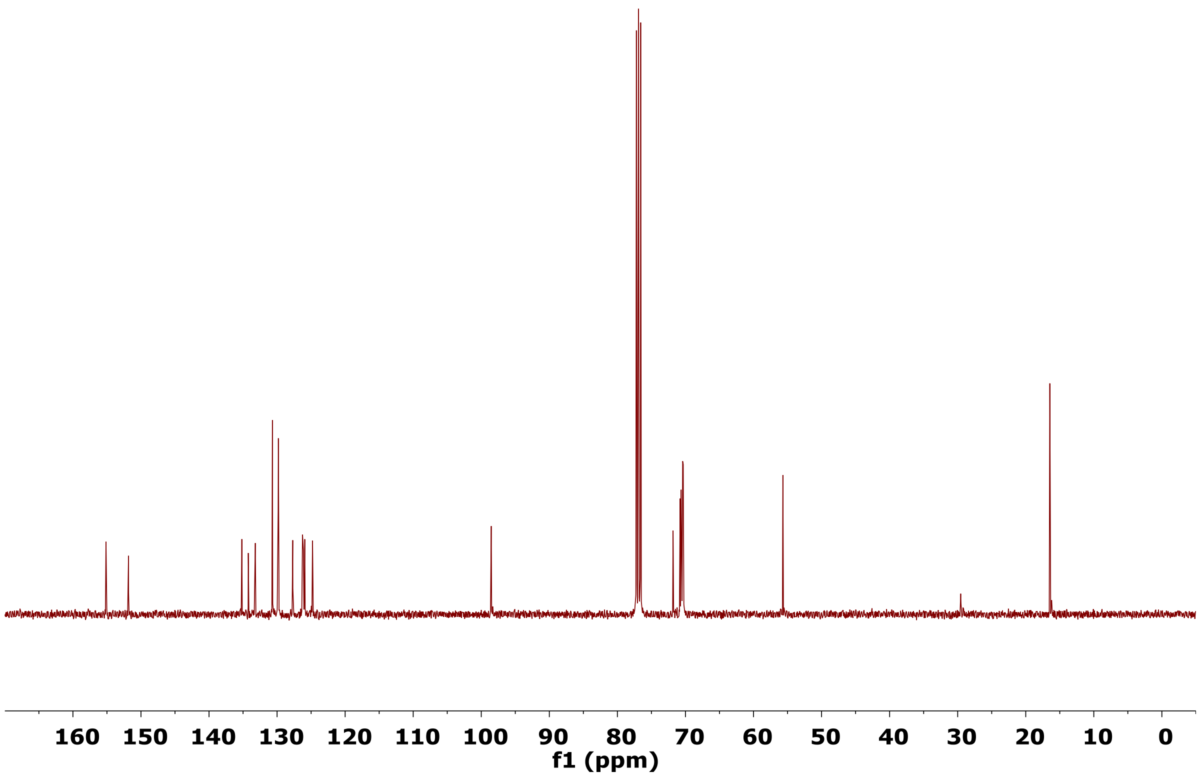

Figure S32: ^{13}C NMR spectrum of (*S*)- **Me-M1$_6$** ($CDCl_3$, 298 K, 400 MHz).

Compound **Me-M1$_7$**

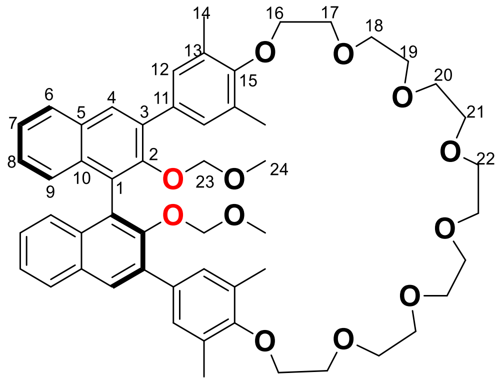

As described above (General procedure **B**), compound compound **Me-M1$_7$** was synthesized using **Me-2** (42.1 mg, 0.0684 mmol, 1.0 equiv), cesium carbonate (56.1 mg, 172 µmol, 2.5 equiv) and **8$_7$** (45.0 mg, 0.0709 mmol, 1.0 equiv) mixed in degassed acetonitrile (68 mL). After purification by column chromatography (methanol/dichloromethane 3:97) the product was obtained as a pale-yellow wax (46.2 mg, 0.0510 mmol, 75.0% yield).

$C_{54}H_{64}O_{12}$: 905.09 g/mol.

^{1}H-NMR (400 MHz, [D$_1$]-chloroform, 298 K) δ [in ppm] = 7.78 (s, 2H, H-4), 7.73 (d, *J* = 8.2 Hz, 2H, H-6), 7.28-7.24 (m, 2H, H-7, 12), 7.18 (d, *J* = 8.5 Hz, 2H, H-9), 7.13-7.09 (m, 2H, H-8), 4.23 (d, *J* = 6.0 Hz, 2H, H-23$_{1/2}$), 4.16 (d, *J* = 6.0 Hz, 2H, H-23$_{1/2}$), 3.95-3.88 (m, 4H, H-16), 3.65-3.59 (m, 4H, H-17), 3.56-3.54 (m, 20H, H-18, 19, 20, 21, 22), 2.27 (s, 12H, H-14), 2.19 (s, 6H, H-24).

^{13}C-NMR (101 MHz, [D$_1$]-chloroform, 298 K) δ [in ppm] = 155.42 (C-15), 152.11 (C-2), 135.46 (C-3), 134.51 (C-11), 133.48 (C-10), 130.96 (C-5), 130.13 (C-4), 130.09 (C-6), 127.98 (C-12), 127.13 (C-8), 126.55 (C-9), 126.41 (C-7), 126.21 (C-1), 125.07 (C-13), 98.81 (C-23), 72.10 (C-16), 71.05, 70.91, 70.69, 70.67, 70.60 (C-18, 19, 20, 21, 22), 70.55 (C-17), 55.96 (C-24), 16.74 (C-14).

^{1}H,^{1}H-COSY (400 MHz / 400 MHz, [D$_1$]-chloroform, 298 K) δ [in ppm] = 7.73/7.28-7.24 (H-6/H-7), 7.28-7.24/2.27 (H-12/H-14), 7.28-7.24/7.73, 7.13-7.09 (H-7/H-6, 8), 7.18/7.13-7.09 (H-9/H-8), 4.23/4.16 (H-23$_{1/2}$/H-23$_{1/2}$), 4.16/4.23 (H-23$_{1/2}$/H-23$_{1/2}$), 3.95-3.88/3.65-3.59 (H-16/H-17), 3.65-3.59/3.95-3.88 (H-17/H-16), 2.27/7.28-7.24 (H-14/ 12).

^{1}H,^{13}C-GHSQC (400 MHz / 101 MHz, [D$_1$]-chloroform, 298 K) δ (^{1}H) / δ (^{13}C) [in ppm] = 7.78/130.13 (H-4/C-4), 7.73/130.09 (H-6/C-6), 7.28-7.24/126.41 (H-7/C-7), 7.28-7.24/127.98 (H-12/C-12), 7.18/126.55 (H-9/C-9), 7.13-7.09/127.13 (H-8/C-8), 4.23/98.81 (H-23$_{1/2}$/C-23), 4.16/98.81 (H-23$_{1/2}$/C-23), 3.95-3.88/72.10 (H-16/C-16), 3.65-3.59/70.55 (H-17/C-17), 3.56-3.54/71.05, 70.91, 70.69, 70.67, 70.60 (H-18, 19, 20, 21, 22/C-18, 19, 20, 21, 22), 2.27/16.74 (H-14/C-14), 2.19/55.96 (H-24/C-24).

^{1}H,^{13}C-GHMBC (400 MHz / 101 MHz, [D$_1$]-chloroform, 298 K) δ (^{1}H) / δ (^{13}C) [in ppm] = 7.78/152.11, 134.51, 133.48, 130.09 (H-4/ C-2, 11, 10, 6), 7.73/133.48, 130.13, 127.13 (H-6/C-10, 4, 8), 7.28-7.24/155.42, 135.46, 130.96, 130.13, 126.55, 16.74 (H-7, 12/C-15, 3, 5, 4, 9, 14), 7.18/130.96, 126.41, 126.21 (H-9/C-5, 7, 1), 7.13-7.09/133.48, 130.09 (H-8/C-10, 6), 4.23/55.96 (H-23$_{1/2}$/C-24), 4.16/55.96 (H-23$_{1/2}$/C-24), 2.27/155.42, 127.98 (H-14/C-15, 12), 2.19/98.81 (H-24/C-23).

MS (ESI-pos, MeOH): *m/z* = 927.4287 ([M+Na]$^+$, calcd. 927.4290 for [$C_{54}H_{64}O_{12}$Na]$^+$).

IR (ATR-FT): $\tilde{\nu}$ (cm^{-1}) = 702, 731, 764, 896, 923, 1090, 1148, 1204, 1264, 1274, 2872, 2987, 3004.

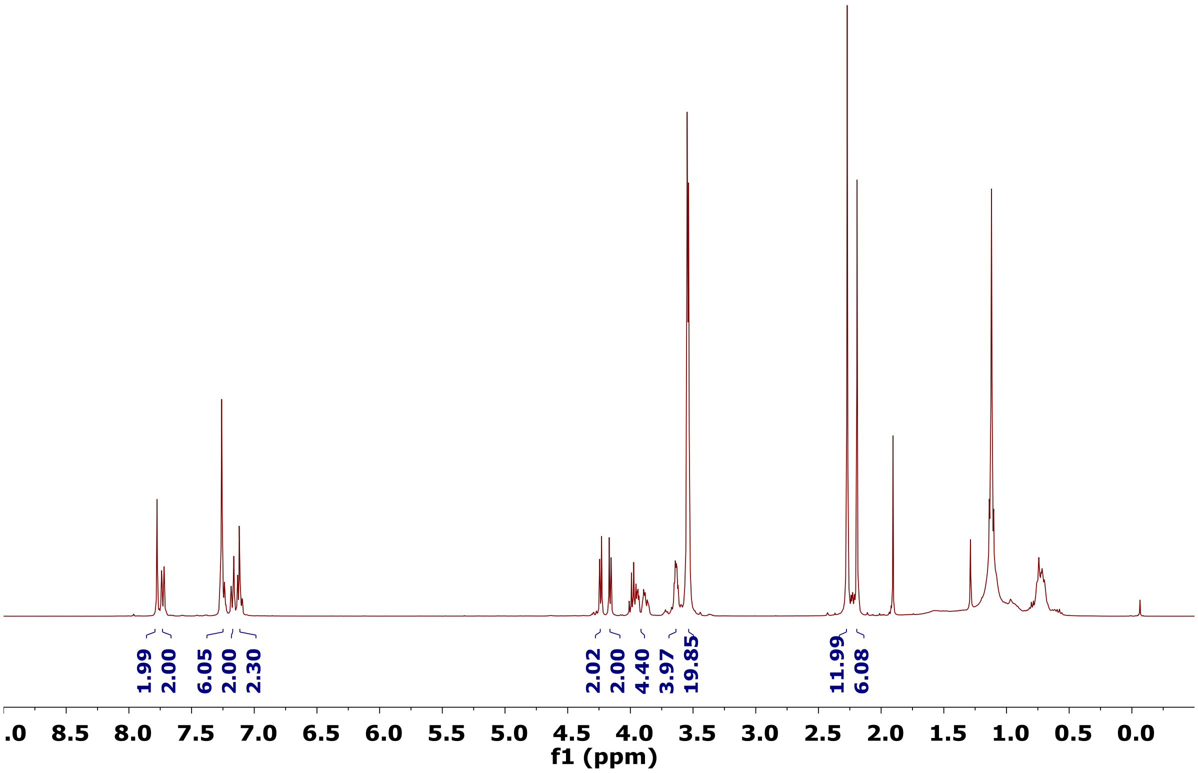

Figure S33: ^{1}H NMR spectrum of (*S*)- **Me-M1**$_7$ ($CDCl_3$, 298 K, 400 MHz).

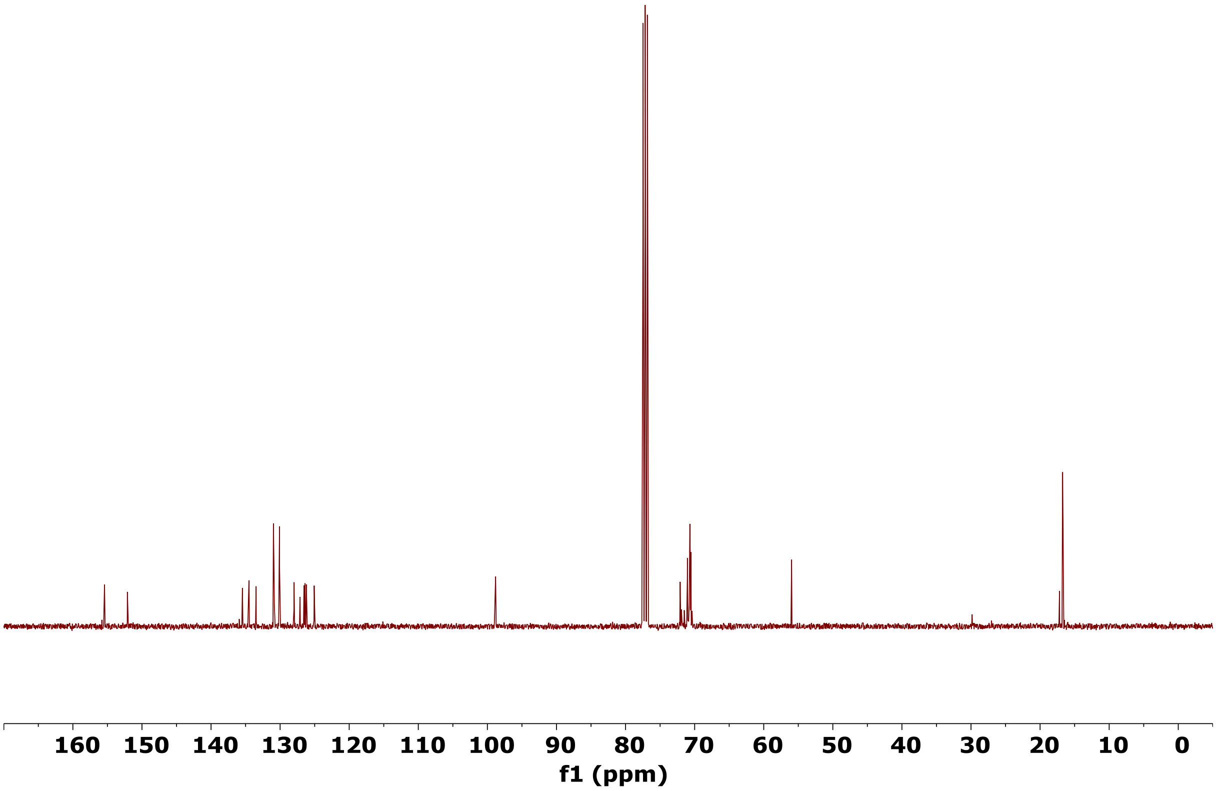

Figure S34: ^{13}C NMR spectrum of (*S*)- **Me-M1**$_7$ ($CDCl_3$, 298 K, 400 MHz).

Compound **Me-M1$_8$**

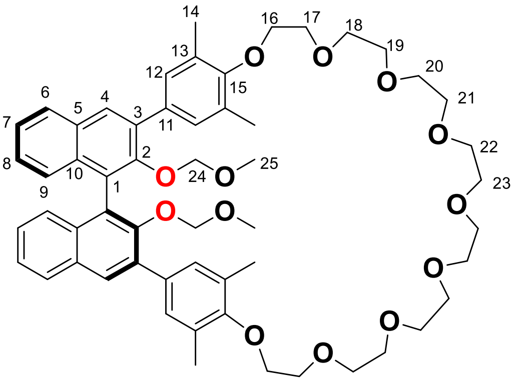

As described above (General procedure **B**), compound compound **Me-M1$_8$** was synthesized using **Me-2** (42.1 mg, 0.0684 mmol, 1.0 equiv), cesium carbonate (56.1 mg, 172 µmol, 2.5 equiv) and **8$_8$** (47.0 mg, 0.0692 mmol, 1.0 equiv) in degassed acetonitrile (70 mL). After purification by column chromatography (methanol/dichloromethane 3:97) the product was obtained as a colorless oil (24.5 mg, 0.0258 mmol, 38.2% yield).

$C_{56}H_{68}O_{13}$:949.15 g/mol.

^{1}H-NMR (400 MHz, [D$_1$]-chloroform, 298 K) δ [in ppm] = 7.76 (s, 2H, H-4), 7.72 (d, *J* = 8.2 Hz, 2H, H-6), 7.26 (s, 4H, H-12), 7.24 (dd, *J* =6.0 Hz, *J* = 2.2 Hz, 2H, H-7), 7.13-7.09 (m, 4H, H-8+9), 4.24 (d, *J* = 6.0 Hz, 2H, H-24$_{1/2}$), 4.17 (d, *J* = 6.0 Hz, 2H, H-24$_{1/2}$), 3.90-3.86 (m, 4H, H-16), 3.66 (t, 4.4 Hz, 4H, H-17), 3.57-3.52 (m, 24H, H-18, 19, 20, 21, 22, 23), 2.26 (s, 12H, H-14), 2.18 (s, 6H, H-25).

^{13}C-NMR (101 MHz, [D$_1$]-chloroform, 298 K) δ [in ppm] = 155.32 (C-15), 151.92 (C-2), 135.44 (C-3), 134.60 (C-11), 133.59 (C-10), 130.96 (C-5), 130.94 (C-1), 130.28 (C-4), 130.13 (C-12), 127.92 (C-6), 126.57, 126.47(C-8, 9), 125.20 (C-1), 125.10 (C-7), 98.74 (C-24), 71.95 (C-16), 71.23, 70.92, 70.83, 70.64 (C-18, 19, 20, 21, 22, 23)*, 70.67 (C-17), 55.95 (C-25), 16.61 (C-14).

* Partially overlapping signals.

^{1}H,^{1}H-COSY (400 MHz / 400 MHz, [D$_1$]-chloroform, 298 K) δ [in ppm] = 7.72/7.24 (H-6/H-7), 7.26/2.26 (H-12/H-14), 7.24/7.72, 7.13-7.09 (H-7/H-6, 8, 9), 7.13-7.09/7.24 (H-8, 9/H-7), 4.24/4.17 (H-24$_{1/2}$/H-24$_{1/2}$), 4.17/4.24 (H-24$_{1/2}$/H-24$_{1/2}$), 3.90-3.86/3.66 (H-16/H-17), 3.66/3.90-3.86 (H-17/H-16).

^{1}H,^{13}C-GHSQC (400 MHz / 101 MHz, [D$_1$]-chloroform, 298 K) δ (^{1}H) / δ (^{13}C) [in ppm] = 7.76/130.28 (H-4/C-4), 7.72/127.92 (H-6/C-6), 7.26/130.13 (H-12/C-12), 7.24/125.10 (H-7/C-7), 7.13-7.09/126.57, 126.47 (H-8, 9/C-8, 9), 4.24/98.74 (H-24$_{1/2}$/C-24), 4.17/98.74 (H-24$_{1/2}$/C-24), 3.90-3.86/71.95 (H-16/C-16), 3.66/70.67 (H-17/C-17), 3.57-3.52/71.23, 70.92, 70.83, 70.64 (H-18, 19, 20, 21, 22, 23/C-18, 19, 20, 21, 22, 23), 2.26/16.61 (H-14/C-14), 2.18/55.95 (H-25/C-25).

^{1}H,^{13}C-GHMBC (400 MHz / 101 MHz, [D$_1$]-chloroform, 298 K) δ (^{1}H) / δ (^{13}C) [in ppm] = 7.76/151.92, 134.60, 133.59, 127.92 (H-4/C-2, 11, 10, 6), 7.72/133.59, 130.28, 126.57 (H-6/C-10, 4, 8), 7.26/155.32, 135.44, 130.28, 16.61 (H-12/C-15, 3, 4, 14), 7.24/130.96, 130.28, 126.57, 126.47 (H-7/C-5, 4, 8, 9), 7.13-7.09/133.59, 127.92, 125.20 (H-8, 9/C-10, 6, 1), 4.24/55.95 (H-24$_{1/2}$/C-25), 4.17/55.95 (H-24$_{1/2}$/C-25), 2.26/155.32, 130.13 (H-14/C-15, 12), 2.18/98.74 (H-25/C-24).

MS (ESI-pos, MeOH): *m/z* = 971.4542 ([M+Na]$^+$, calcd. 971.4552 for [$C_{56}H_{68}O_{13}Na$]$^+$).

IR (ATR-FT): $\tilde{\nu}$ (cm^{-1}) = 702, 731, 764, 888, 1149, 1264, 2883, 2945, 2952, 3005.

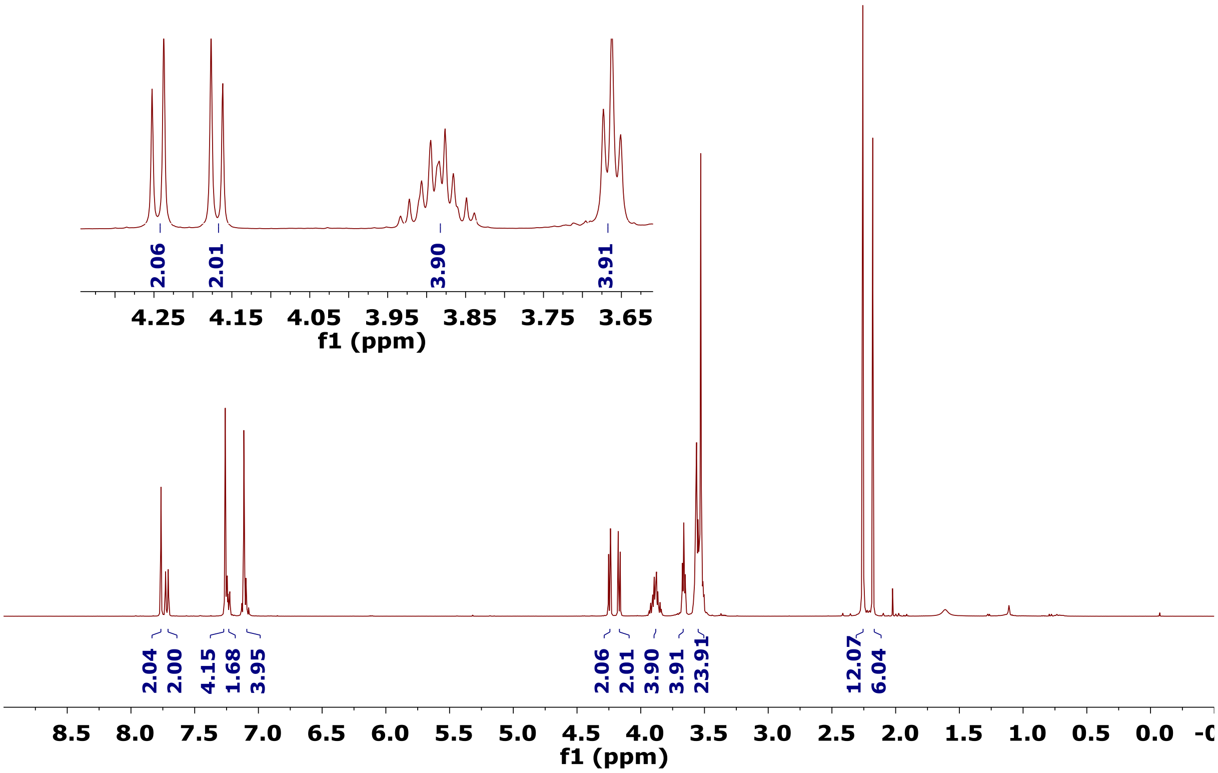

Figure S35: ^{1}H NMR spectrum of (*S*)- **Me-M1$_{8}$** ($CDCl_3$, 298 K, 400 MHz).

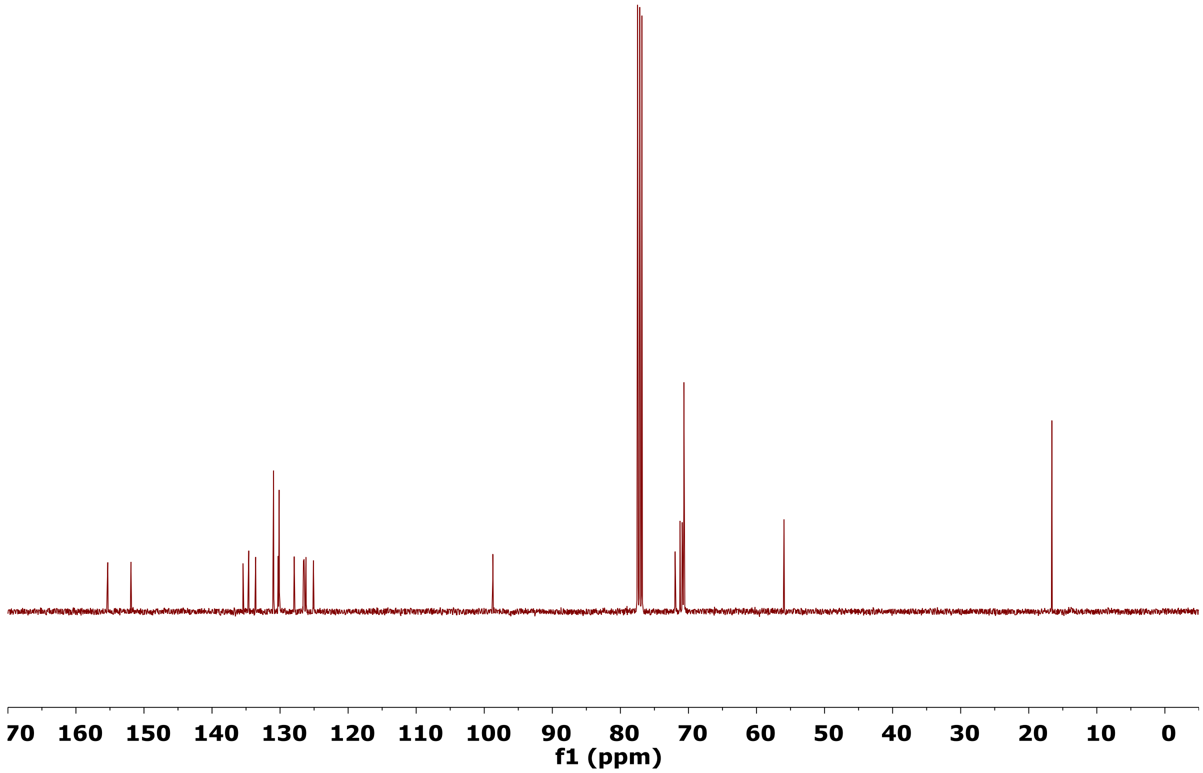

Figure S36: ^{13}C NMR spectrum of (*S*)- **Me-M1$_{8}$** ($CDCl_3$, 298 K, 400 MHz).

Compound **H-M1₅**

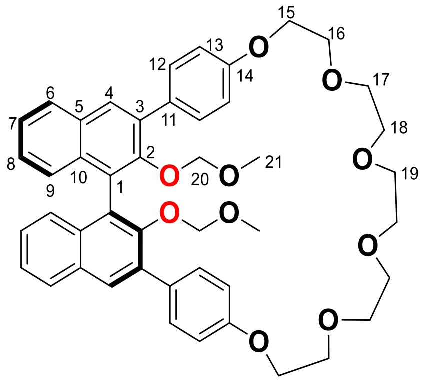

As described above (General procedure **B**), compound **H-M1$_5$** was synthesized using compound **H-2** (39.1 mg, 0.0699 mmol, 1.0 equiv), cesium carbonate (57.0 mg, 175 µmol, 2.5 equiv) and **8$_5$** (38.2 mg, 0.0699 mmol, 1.0 equiv) in degassed acetonitrile (70 mL). After purification by column chromatography (methanol/dichloromethane 1:50) the product was obtained as a clear wax (5.9 mg, 0.0078 mmol, 11% yield).

$C_{46}H_{48}O_{10}$: 760.88 g/mol.

^{1}H-NMR (400 MHz, [D$_1$]-chloroform, 298 K) δ [in ppm] = 7.92 (s, 2H, H-4), 7.89 (d, *J* = 8.7 Hz, 2H, H-6), 7.63 (d, *J* = 8.8 Hz, 4H, H-12), 7.43-7.39 (m, 4H, H-8, 9), 7.30-7.26 (m, 2H, H-7), 7.22 (d, *J* = 8.8 Hz, 4H, H-13), 4.46-4.40 (m, 2H, H-15$_{1/2}$), 4.33-4.27 (m, 4H, H-15$_{1/2}$, 20$_{1/2}$), 4.21 (d, *J* = 5.9 Hz, 2H, H-20$_{1/2}$), 3.79-3.56 (m, 16H, H-16, 17, 18, 19), 2.32 (s, 6H, H-21).

^{13}C-NMR (101 MHz, [D$_1$]-chloroform, 298 K) δ [in ppm] = 158.22 (C-14), 151.38 (C-2), 134.96 (C-3), 133.41 (C-10), 131.52 (C-11), 130.87 (C-5), 130.66 (C-12), 130.11 (C-4), 127.71 (C-6), 126.53 (C-9), 126.43 (C-8), 126.03 (C-1), 125.05 (C-7), 114.46 (C-13), 98.33 (C-20), 70.89 70.67 (C-17, 18, 19)*, 69.76 (C-16), 67.47 (C-15), 55.84 (C-21).

* Partially overlapping signals.

^{1}H,^{1}H-COSY (400 MHz / 400 MHz, [D$_1$]-chloroform, 298 K) δ [in ppm] = 7.89/7.30-7.26 (H-6/H-7), 7.63/7.22 (H-12/H-13), 7.43-7.39/7.30-7.26 (H-8/H-7), 7.30-7.26/7.89, 7.43-7.39 (H-7/H-6, 8), 7.22/7.63 (H-13/H-12), 4.46-4.40/4.33-4.27(H-15$_{1/2}$/H-15$_{1/2}$), 4.33-4.27/4.46-4.40 (H-15$_{1/2}$/H-15$_{1/2}$), 4.33-4.27/4.21 (H-20$_{1/2}$/H-20$_{1/2}$), 4.21/4.33-4.27 (H-20$_{1/2}$/H-20$_{1/2}$).

^{1}H,^{13}C-GHSQC (400 MHz / 101 MHz, [D$_1$]-chloroform, 298 K) δ (^{1}H) / δ (^{13}C) [in ppm] = 7.92/130.11 (H-4/C-4), 7.89/127.71 (H-6/C-6), 7.63/130.66 (H-12/C-12), 7.43-7.39/126.53, 126.43 (H-8, 9/C-8, 9), 7.30-7.26/125.05 (H-7/C-7), 7.22/114.46 (H-13/C-13), 4.46-4.40/67.47 (H-15/C-15), 4.33-4.27/67.47 (H-15$_{1/2}$/C-15), 4.33-4.27/98.33 (H-20$_{1/2}$/C-20), 4.21/98.33 (H-20$_{1/2}$/C-20), 3.79-3.56/69.76 (H-16/C-16), 3.79-3.56/70.89, 70.67 (H-17,18,19/C-17, 18, 19)*, 2.32/55.97 (H-21/C-21).

* Partially overlapping signals.

^{1}H,^{13}C-GHMBC (400 MHz / 101 MHz, [D$_1$]-chloroform, 298 K) δ (^{1}H) / δ (^{13}C) [in ppm] = 7.92/151.38, 133.41, 131.52, 127.71 (H-4/C-2, 10, 11, 6), 7.89/133.41, 130.11, 126.43 (H-6/C-10, 4, 8), 7.63/158.22, 134.96, 114.46 (H-12/C-14, 3, 13), 7.43-7.39/130.87, 126.03, 125.05 (H-8, 9/C-5, 1, 7), 7.30-7.26/130.87, 126.53 (H-7/C-5, 9), 7.22/158.22, 131.52 (H-13/C-14, 11), 4.33-4.27/152.38, 55.84 (H-20$_{1/2}$/C-2, 21), 4.21/151.38, 55.84 (H-20$_{1/2}$/C-2, 21), 2.32/98.33 (H-21/C-20).

MS (ESI-pos, MeOH**)**: *m/z* = 783.3130 ([M+Na]$^+$, calcd. 783.3140 for [$C_{46}H_{48}O_{10}$Na]$^+$).

IR (ATR-FT): $\tilde{\nu}$ (cm^{-1}) = 702, 732, 749, 772, 970, 1131, 1150, 1263, 1275, 1511, 2887, 2989, 3004, 3053.

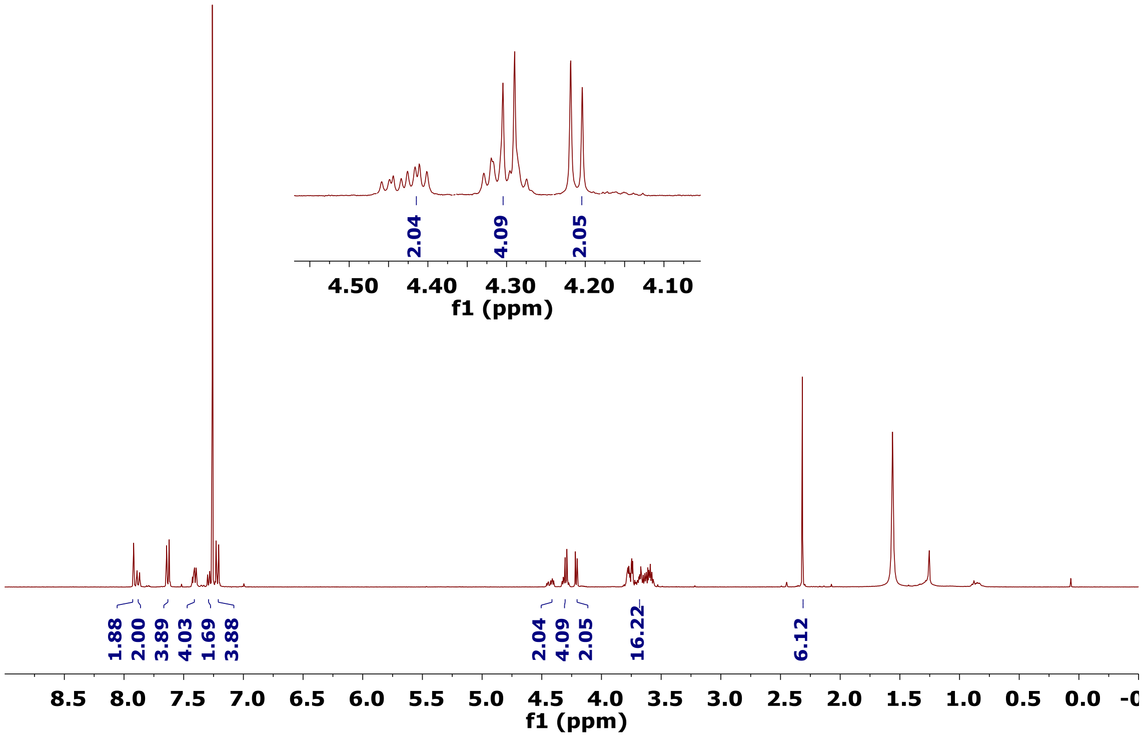

Figure S37: ^{1}H NMR spectrum of (*S*)-**H-M1$_5$** ($CDCl_3$, 298 K, 400 MHz).

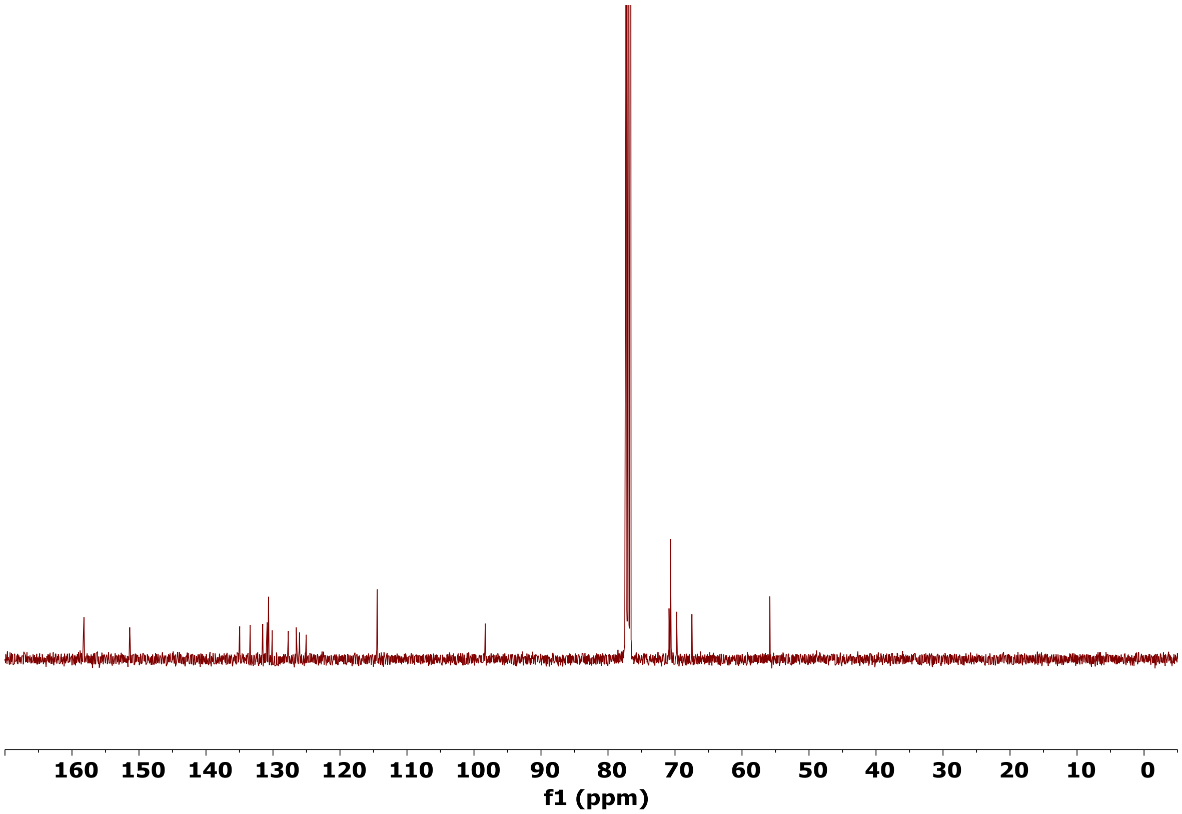

Figure S38: ^{13}C NMR spectrum of (*S*)-**H-M1$_8$** ($CDCl_3$, 298 K, 400 MHz).

Compound **H-M1$_6$**

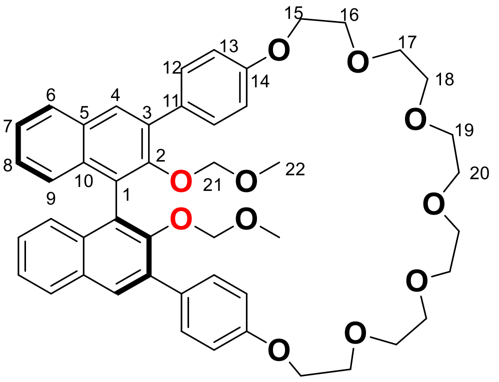

As described above (General procedure **B**), compound **H-M1$_6$** was synthesized using compound **H-2** (51.0 mg, 0.0913 mmol, 1.0 equiv), cesium carbonate (81.2 mg, 249 µmol, 2.5 equiv) and **8$_6$** (53.9 mg, 0.0912 mmol, 1.0 equiv) dissolved in degassed acetonitrile (91 mL). After purification by column chromatography (methanol/dichloromethane 1:25) the product was obtained as a colorless wax (22.1 mg, 0.0274 mmol, 29.7% yield).

$C_{48}H_{52}O_{11}$: 804.93 g/mol.

^{1}H-NMR (400 MHz, [D$_1$]-chloroform, 298 K) δ [in ppm] = 8.02 (s, 2H, H-4), 7.97 (d, *J* = 8.1 Hz, 2H, H-6), 7.77 (d, *J* = 8.7 Hz, 4H, H-12), 7.50 (dd, *J* = 1.3 Hz, *J* = 6.7 Hz, 2H, H-7), 7.44 (d, *J* = 8.4 Hz, 2H, H-9), 7.36 (dd, *J* = 1.3 Hz, *J* = 6.7 Hz, 2H, H-8), 7.25 (d, *J* = 8.7 Hz, 4H, H-13), 4.45-4.40 (m, 6H, H-21$_{1/2}$, 15), 4.34 (d, *J* = 5.8 Hz, 2H, H-21$_{1/2}$), 3.89 (t, *J* = 4.8 Hz, 4H, H-16), 3.78-3.71 (m, 16H, H-17, 18, 19, 20), 2.41 (s, 6H, H-22).

^{13}C-NMR (101 MHz, [D$_1$]-chloroform, 298 K) δ [in ppm] = 158.65 (C-14), 152.03 (C-2), 135.26 (C-3), 133.38 (C-10), 131.85 (C-11), 131.04 (C-5), 130.72 (C-12), 129.92 (C-4), 128.02 (C-6), 126.53 (C-9), 126.47 (C-8), 126.24 (C-1), 125.13 (C-7), 116.07 (C-13), 98.73 (C-21), 71.08 (C-16), 70.81, 70.74, 70.69, 70.36 (C-17, 18, 19, 20), 68.76 (C-15), 55.97 (C-22).

^{1}H,^{1}H-COSY (400 MHz / 400 MHz, [D$_1$]-chloroform, 298 K) δ [in ppm] = 7.97/7.50 (H-6/H-7), 7.77/7.25 (H-12/H-13), 7.50/7.97, 7.36 (H-7/H-6, 8), 7.44/7.36 (H-9/H-8), 7.36/7.50, 7.44 (H-8/H-7, 9), 7.25/7.77 (H-13/H-12), 4.45-4.40/4.34, 3.89 (H-21$_{1/2}$, 15/H-21$_{1/2}$, 16), 4.34/4.45-4.40 (H-21$_{1/2}$/H-21$_{1/2}$), 3.89/4.45-4.40 (H-16/ H-15).

^{1}H,^{13}C-GHSQC (400 MHz / 101 MHz, [D$_1$]-chloroform, 298 K) δ (^{1}H) / δ (^{13}C) [in ppm] = 8.02/129.92 (H-4/C-4), 7.97/128.02 (H-6/C-6), 7.77/130.72 (H-12/C-12), 7.50/125.13 (H-7/C-7), 7.44/126.53 (H-9/C-9), 7.38-7.34/126.47 (H-8/C-8), 7.25/116.07 (H-13/C-13), 4.45-4.40/98.73, 68.76 (H-21$_{1/2}$, 15/C-21$_{1/2}$, 15), 4.34/98.73 (H-21$_{1/2}$/C-21), 3.89/71.08 (H-16/C-16), 3.78-3.71/70.81, 70.74, 70.69, 70.36 (H-17, 18, 19, 20/C-17, 18, 19, 20), 2.41/55.97 (H-22/C-22).

^{1}H,^{13}C-GHMBC (400 MHz / 101 MHz, [D$_1$]-chloroform, 298 K) δ (^{1}H) / δ (^{13}C) [in ppm] = 8.02/152.03, 133.38, 131.85, 128.02 (H-4/C-2, 10, 11, 6), 7.97/133.38, 129.92, 126.47 (H-6/C-10, 4, 8), 7.77/158.65, 135.26, 116.07 (H-12/C-14, 3, 13), 7.50/131.04, 126.53 (H-7/C-5, 9), 7.44/131.04, 126.24, 125.13 (H-9/C-5, 1, 7), 7.36/133.38, 128.02 (H-8/C-10, 6), 7.25/158.65, 131.85 (H-13/C-14, 11), 4.45-4.40/152.03, 55.97 (H-21$_{1/2}$/C-2, 22), 4.34/152.03, 55.97 (H-21$_{1/2}$/C-2, 22), 2.41/98.73 (H-22/C-21).

MS (ESI-pos, MeOH**)**: *m/z* = 827.3399 ([M+Na]$^+$, calcd. 827.3402 for [$C_{48}H_{52}O_{11}Na$]$^+$).

IR (ATR-FT): $\tilde{\nu}$ (cm^{-1}) = 702, 731, 764, 971, 1131, 1148, 1264, 1274, 1512, 2884, 2987, 2997, 3004.

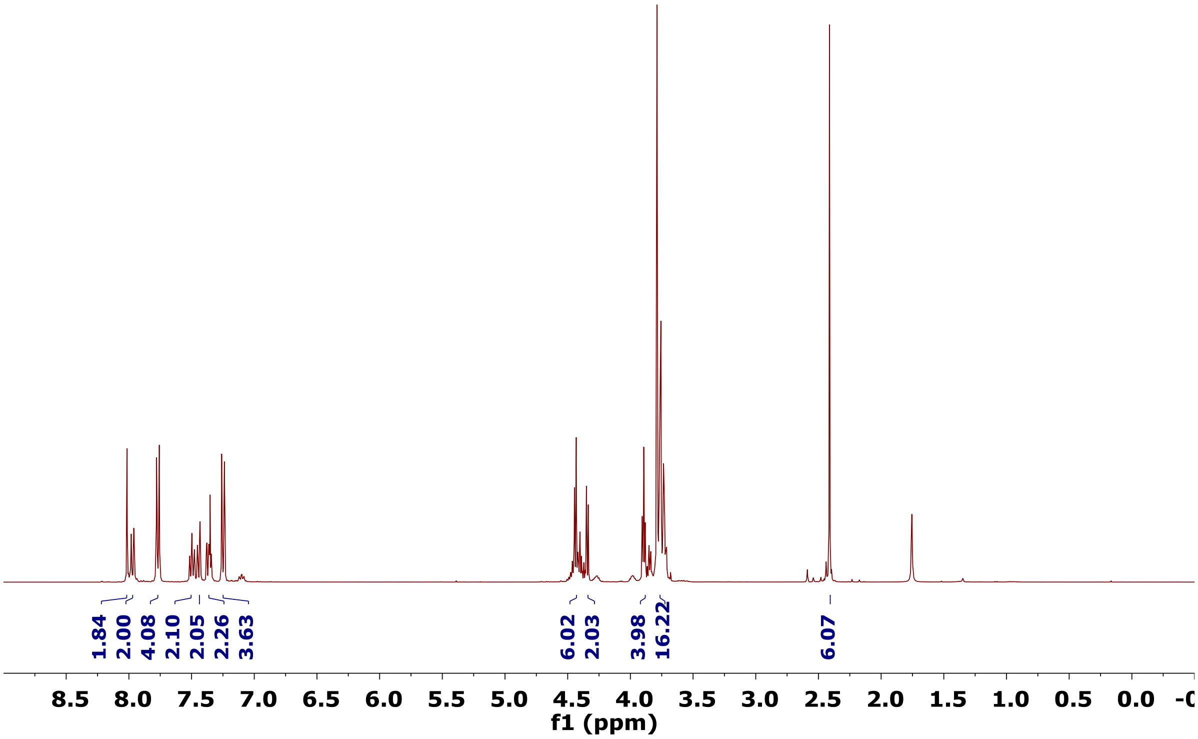

Figure S39: ^{1}H NMR spectrum of (*S*)-**H-M1$_6$** ($CDCl_3$, 298 K, 400 MHz).

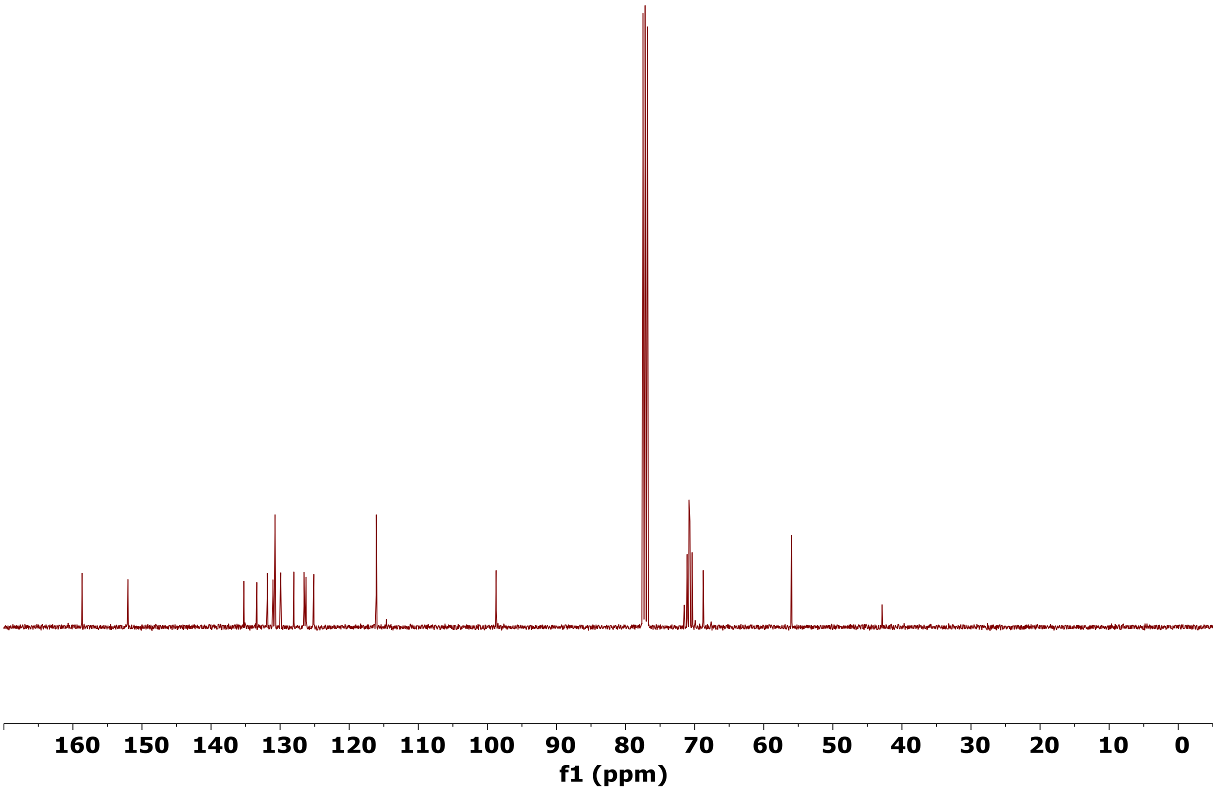

Figure S40: ^{13}C NMR spectrum of (*S*)-**H-M1$_6$** ($CDCl_3$, 298 K, 400 MHz).

Compound **H-M1$_7$**

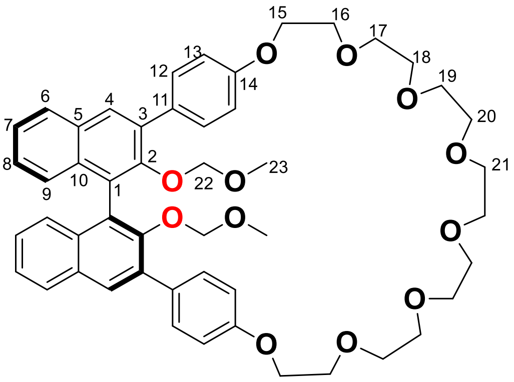

As described above (General procedure **B**), compound **H-M1$_7$** was synthesized using compound **H-2** (47.2 mg, 0.0844 mmol, 1.0 equiv), cesium carbonate (68.9 mg, 212 µmol, 2.5 equiv) and **8$_7$** (51.0 mg, 0.0804 mmol, 1.0 equiv) dissolved in degassed acetonitrile (85 mL). After purification by column chromatography (methanol/dichloromethane 1:25) the product was obtained as a pale brown wax (22.8 mg, 0.0268 mmol, 32.1% yield).

$C_{50}H_{56}O_{12}$: 848.99 g/mol.

^{1}H-NMR (400 MHz, [D$_1$]-chloroform, 298 K) δ [in ppm] = 7.92 (s, 2H, H-4), 7.87 (d, *J* = 8.3 Hz, 2H, H-6), 7.69 (d, *J* = 8.7 Hz, 4H, H-12), 7.40 (dd, *J* = 1.4 Hz, *J* = 8.1 Hz, 2H, H-7), 7.32 (d, *J* = 8.6 Hz, 2H, H-9), 7.28-7.24 (m, 2H, H-8), 7.09 (d, *J* = 8.7 Hz, 4H, H-13), 4.36 (d, *J* = 5.9 Hz, 2H, H-22$_{1/2}$), 4.29-4.26 (m, 6H, H-22$_{1/2}$, 15), 3.83 (t, *J* = 5.0 Hz, 4H, H-16), 3.70-3.64 (m, 20H, H-17, 18, 19, 20, 21), 2.32 (s, 6H, H-23).

^{13}C-NMR (101 MHz, [D$_1$]-chloroform, 298 K) δ [in ppm] = 158.52 (C-14), 151.96 (C-2), 135.22 (C-3), 133.45 (C-10), 131.84 (C-11), 131.05 (C-5), 130.80 (C-12), 130.01 (C-4), 128.00 (C-6), 126.57 (C-1), 126.54 (C-9), 126.25 (C-8), 125.15 (C-7), 115.57 (C-13), 98.71 (C-22), 71.51, 71.23, 70.82, 70.76, 70.62 (C-17, 18, 19, 20, 21), 69.98 (C-16), 68.35 (C-15), 56.00 (C-23).

^{1}H,^{1}H-COSY (400 MHz / 400 MHz, [D$_1$]-chloroform, 298 K) δ [in ppm] = 7.87/7.40 (H-6/H-7), 7.69/7.09 (H-12/H-13), 7.40/7.87, 7.28-7.24 (H-7/H-6, 8), 7.28-7.24/7.40, 7.32 (H-8/H-7, 9), 7.32/7.28-7.24 (H-9/H-8), 7.09/7.69 (H-13/H-12), 4.36/4.29-4.26 (H-22$_{1/2}$/H-22$_{1/2}$, 15), 4.29-4.26/4.36, 3.83 (H-22$_{1/2}$, 15/H-22$_{1/2}$, 16), 3.83/4.29-4.26 (H-16/ H-22$_{1/2}$, 15).

^{1}H,^{13}C-GHSQC (400 MHz / 101 MHz, [D$_1$]-chloroform, 298 K) δ (^{1}H) / δ (^{13}C) [in ppm] = 7.92/130.01 (H-4/C-4), 7.87/128.00 (H-6/C-6), 7.69/130.80 (H-12/C-12), 7.40/125.15 (H-7/C-7), 7.32/126.54 (H-9/C-9), 7.28-7.24/126.25 (H-8/C-8), 7.09/115.57 (H-13/C-13), 4.36/98.71 (H-22$_{1/2}$/C-22), 4.29-4.26/98.71(H-22$_{1/2}$/C-22)4.29-4.26/68.35 (H-15/C-15), 3.83/69.98 (H-16/C-16), 3.70-3.64/71.51, 71.23, 70.82, 70.76, 70.62 (H-17, 18, 19, 20, 21/C-17, 18, 19, 20, 21), 2.32/56.00 (H-23/C-23).

^{1}H,^{13}C-GHMBC (400 MHz / 101 MHz, [D$_1$]-chloroform, 298 K) δ (^{1}H) / δ (^{13}C) [in ppm] = 7.92/151.96, 133.45, 131.84, 128.00 (H-4/C-2, 10, 11, 6), 7.87/133.45, 130.01, 126.25 (H-6/C-10, 4, 8), 7.69/158.52, 135.22, 115.57 (H-12/C-14, 3, 13), 7.40/131.05, 126.54 (H-7/C-5, 9), 7.32/131.05, 126.57, 125.15 (H-9/C-5, 1, 7), 7.28-7.24/133.45, 128.00 (H-8/C-10, 6), 7.09/158.52, 131.84 (H-13/C-14, 11), 4.36/151.96, 56.00 (H-22$_{1/2}$/C-2, 23), 4.29-4.26/151.96, 56.00 (H-22$_{1/2}$/C-2, 23), 2.32/98.71 (H-23/C-22).

MS (ESI-pos, MeOH): *m/z* = 871.3661 ([M+Na]$^+$, calcd. 871.3664 for [$C_{50}H_{56}O_{12}Na$]$^+$).

IR (ATR-FT): $\tilde{\nu}$ (cm^{-1}) = 701, 751, 764, 883, 914, 1027, 1098, 1261, 1267, 1275, 1510, 1607, 2708, 2870, 2988, 3005, 3053.

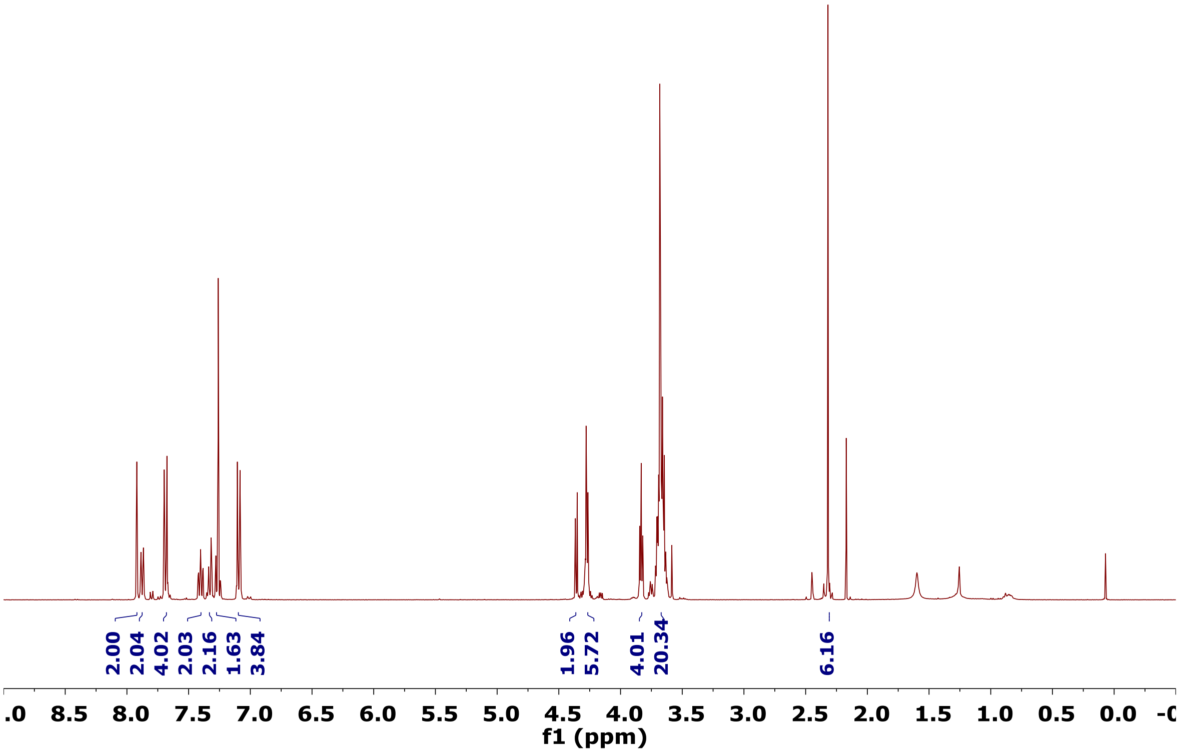

Figure S41: ^{1}H NMR spectrum of (*S*)-**H-M1$_7$** ($CDCl_3$, 298 K, 400 MHz).

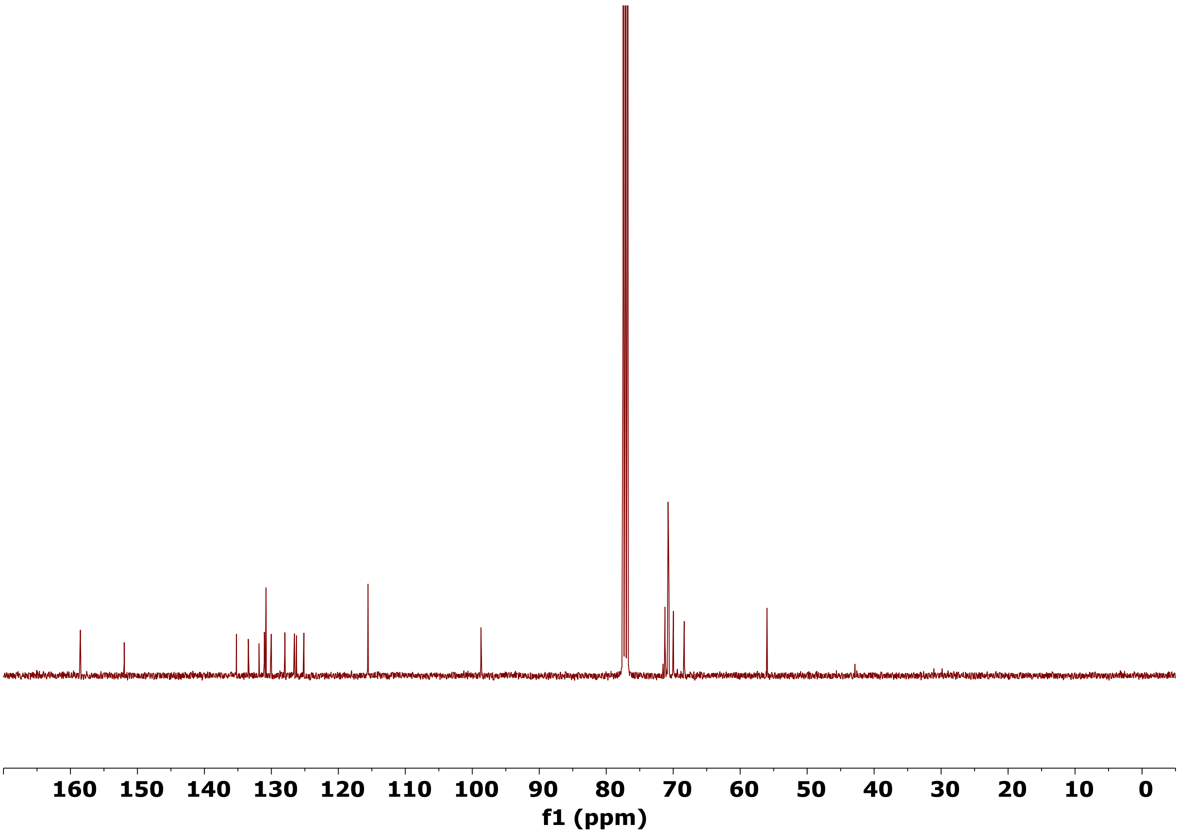

Figure S42: ^{13}C NMR spectrum of (*S*)-**H-M1$_7$** ($CDCl_3$, 298 K, 400 MHz).

Compound **H-M1$_8$**

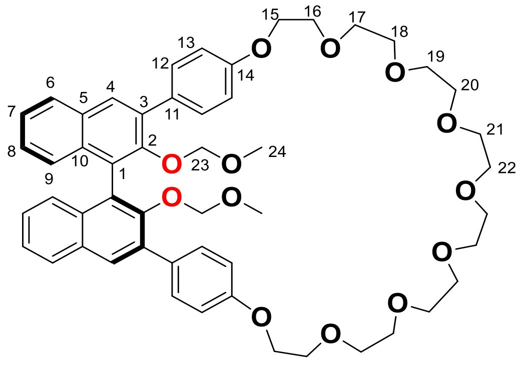

As described above (General procedure **B**), compound **H-M1$_8$** was synthesized using compound **H-2** (40.1 mg, 0.0718 mmol, 1.0 equiv), cesium carbonate (46.1 mg, 141 µmol, 2.5 equiv) and **8$_8$** (49.0 mg, 0.0722 mmol, 1.0 equiv) in degassed acetonitrile (75 mL). After purification by column chromatography (methanol/dichloromethane 1:25) the product was obtained as a yellow oil (25.7 mg, 0.0288 mmol, 40.3% yield).

$C_{52}H_{60}O_{13}$: 893.04 g/mol.

^{1}H-NMR (400 MHz, [D$_1$]-chloroform, 298 K) δ [in ppm] = 8.10 (s, 2H, H-4), 8.06 (d, *J* = 8.2 Hz, 2H, H-6), 7.88 (d, *J* = 8.8 Hz, 4H, H-12), 7.59 (dd, *J* = 1.4 Hz, *J* = 6.6 Hz, 2H, H-7), 7.50 (d, *J* = 8.7 Hz, 2H, H-9), 7.45 (dd, *J* = 1.3 Hz, *J* = 6.6 Hz, 2H, H-8), 7.25 (d, *J* = 8.7 Hz, 4H, H-13), 4.55 (d, *J* = 5.9 Hz, 2H, H-$23_{1/2}$), 4.48 (d, *J* = 5.9 Hz, 2H, H-$23_{1/2}$), 4.43 (t, *J* = 5.0 Hz, 4H, H-15), 4.05 (t, *J* = 5.0 Hz, 4H, H-16), 3.91-3.83 (m, 24H, H-17, 18, 19, 20, 21, 22), 2.51 (s, 6H, H-24).

^{13}C-NMR (101 MHz, [D$_1$]-chloroform, 298 K) δ [in ppm] = 158.43 (C-14), 151.85 (C-2), 135.17 (C-3), 133.47 (C-10), 131.77 (C-11), 131.03 (C-5), 130.80 (C-12), 130.05 (C-4), 127.95 (C-6), 126.59 (C-1), 126.57 (C-9), 126.22 (C-8), 125.15 (C-7), 115.17 (C-13), 98.64 (C-23), 70.79, 70.75, 70.73, 70.70, 70.64, 70.61 (C-17, 18, 19, 20, 21, 22), 71.19 (C-16), 69.83 (C-15), 55.98 (C-24).

^{1}H,^{1}H-COSY (400 MHz / 400 MHz, [D$_1$]-chloroform, 298 K) δ [in ppm] = 8.06/7.59 (H-6/H-7), 7.88/7.25 (H-12/H-13), 7.59/8.06, 7.45 (H-7/H-6, 8),7.50/7.45 (H-9/H-8), 7.45/7.59, 7.50 (H-8/H-7, 9), 7.25/7.88 (H-13/H-12), 4.55/4.48 (H-$23_{1/2}$/H-$23_{1/2}$), 4.48/4.55 (H-$23_{1/2}$/H-$23_{1/2}$), 4.43/4.05 (H-15/H-16), 4.05/4.43 (H-16/H-15).

^{1}H,^{13}C-GHSQC (400 MHz / 101 MHz, [D$_1$]-chloroform, 298 K) δ (^{1}H) / δ (^{13}C) [in ppm] = 8.10/130.05 (H-4/C-4), 8.06/127.95 (H-6/C-6), 7.88/130.80 (H-12/C-12), 7.59/125.15 (H-7/C-7), 7.50/126.57 (H-9/C-9), 7.45/126.22 (H-8/C-8), 7.25/115.17 (H-13/C-13), 4.55/98.64 (H-$23_{1/2}$/C-23), 4.48/98.64 (H-$23_{1/2}$/C-23), 4.43/69.83 (H-15/C-15), 4.05/71.19 (H-16/C-16), 3.91-3.83/70.79, 70.75, 70.73, 70.70, 70.64, 70.61 (H-17, 18, 19, 20, 21, 22/C-17, 18, 19, 20, 21, 22), 2.32/55.85 (H-24/C-24).

^{1}H,^{13}C-GHMBC (400 MHz / 101 MHz, [D$_1$]-chloroform, 298 K) δ (^{1}H) / δ (^{13}C) [in ppm] = 8.10/151.85, 133.47, 131.77, 127.95 (H-4/C-2, 10, 11, 6), 8.06/133.47, 130.05, 126.22 (H-6/C-10, 4, 8), 7.88/158.43, 135.17, 115.17 (H-12/C-14, 3, 13), 7.59/131.03, 126.57 (H-7/C-5, 9), 7.50/131.03, 126.59, 125.15 (H-9/C-5, 1, 7), 7.45/133.47, 127.95 (H-8/C-10, 6), 7.25/158.43, 131.77 (H-13/C-14, 11), 4.55/151.85, 55.98 (H-$23_{1/2}$/C-2, 24), 4.48/151.85, 55.98 (H-$23_{1/2}$/C-2, 24), 2.51/98.64(H-24/C-23).

MS (ESI-pos, MeOH): *m/z* = 915.3916 ([M+Na]$^+$, calcd. 915.3926 for [$C_{52}H_{60}O_{13}Na$]$^+$).

IR (ATR-FT): $\tilde{\nu}$ (cm^{-1}) = 725, 750, 764, 890, 909, 1150, 1261, 1267, 1275, 2870, 2989, 3005.

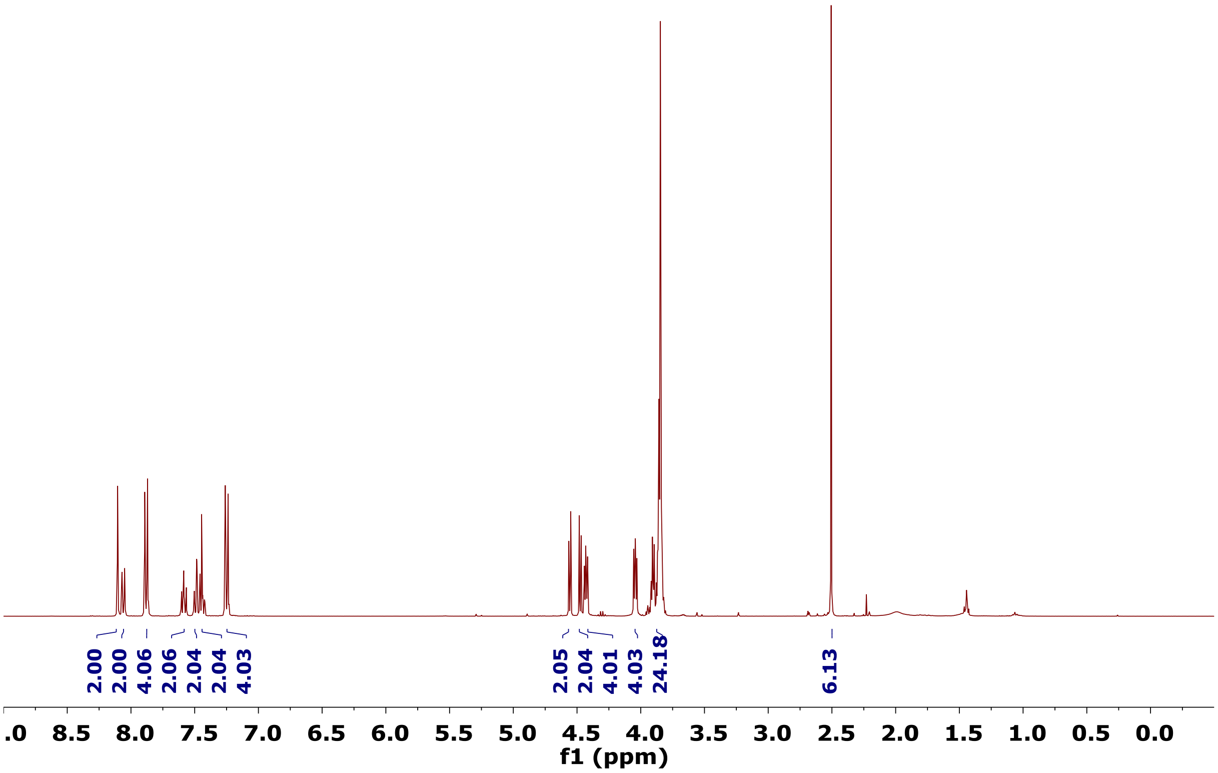

Figure S43: ^{1}H NMR spectrum of (*S*)-**H-M1$_8$** ($CDCl_3$, 298 K, 400 MHz).

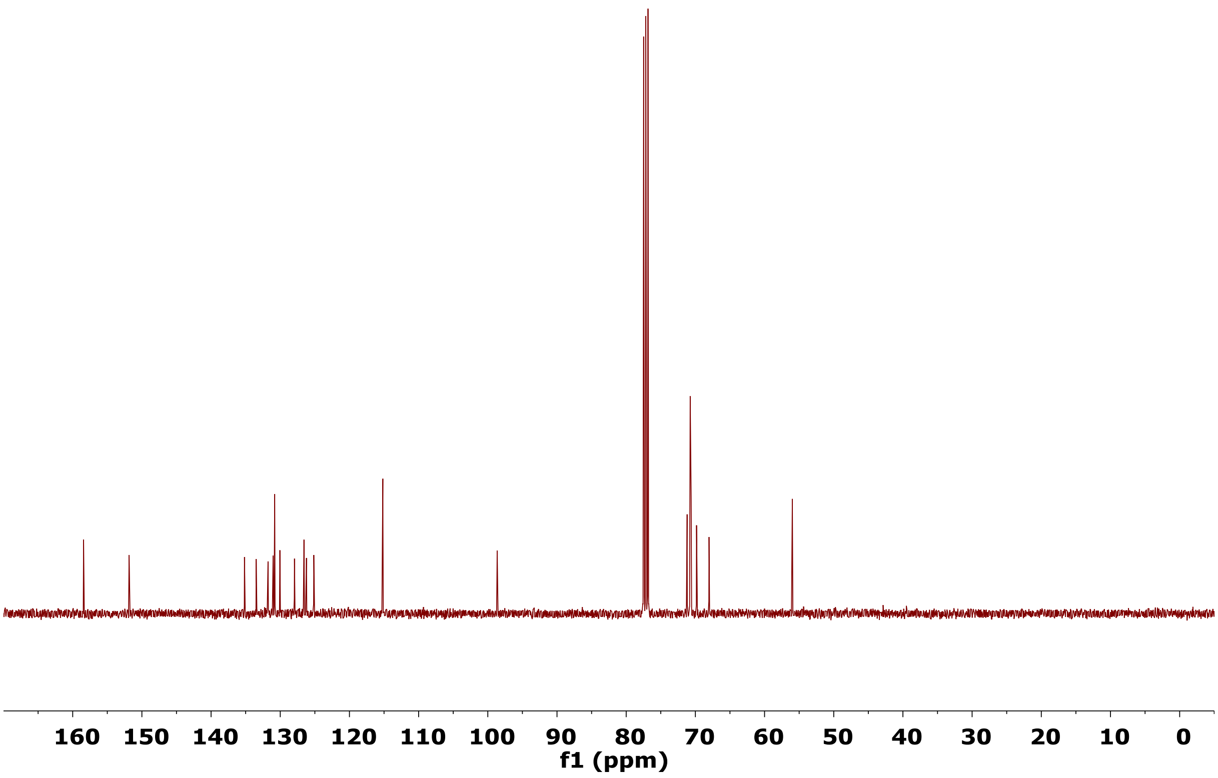

Figure S44: ^{13}C NMR spectrum of (*S*)-**H-M1$_8$** ($CDCl_3$, 298 K, 400 MHz).

Compound **iPr-M1$_5$**

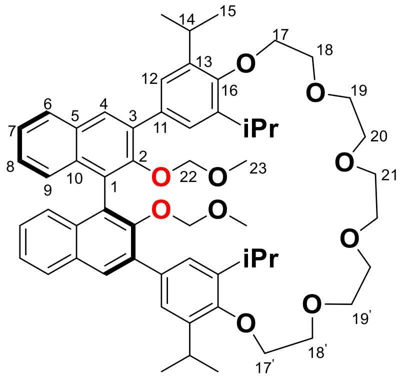

As described above (General procedure **B**), compound **iPr-M1$_5$** was synthesized usingcompound **iPr-2** (41.2 mg, 0.0566 mmol, 1.0 equiv), cesium carbonate (46.1 mg, 0.141 mmol, 2.5 equiv) and **8$_5$** (32.9 mg, 0.0602 mmol, 1.0 equiv) in degassed acetonitrile (65 mL). After purification by column chromatography (cyclohexane/ethyl acetate 5:1) the product was obtained as white wax (10.6 mg, 0.0114 mmol, 19.6% yield).

$C_{58}H_{72}O_{10}$: 929.20 g/mol.

^{1}H-NMR (400 MHz, [D$_1$]-chloroform, 298 K) δ [in ppm] = 7.94 (s, 2H, H-4), 7.90 (d, *J* = 8.1 Hz, 2H, H-6), 7.48 (d, *J* = 7.8 Hz, 2H, H-9), 7.43-7.39 (m, 6H, H-8, 12), 7.29 (dd, *J* =7.0 Hz, *J* = 1.2 Hz, 2H, H-7), 4.25 (d, *J* = 6.0 Hz, 2H, H-22$_{1/2}$), 4.21 (d, *J* = 6.0 Hz, 2H, H-22$_{1/2}$), 4.20-4.16 (m, 2H, H-17$_{1/2}$), 3.95-3.90 (m, 2H, 17$_{1/2}$), 3.78-3.74 (m, 2H, H-18$_{1/2}$), 3.69-3.52 (m, 18H, H-18$_{1/2}$, 19, 20, 21, 14), 2.35 (s, 6H, H-23), 1.31(d, *J* = 6.9 Hz, 12H, H-15$_{1/2}$), 1.23 (d, *J* = 6.9 Hz, 12H, H-15$_{1/2}$).

^{13}C-NMR (101 MHz, [D$_1$]-chloroform, 298 K) δ [in ppm] = 152.44 (C-16), 151.58 (C-2), 141.63 (C-13), 135.74 (C-3), 135.20 (C-11), 133.39 (C-10), 130.80 (C-5), 129.97 (C-4), 127.77 (C-6), 126.42 (C-1), 126.40 (C-9), 126.00 (C-8), 125.46 (C-12), 124.95 (C-7), 98.31 (C-22), 74.00 (C-17), 71.09, 70.83, 70.76, (C-19, 20, 21), 70.56 (C-18), 55.78 (C-23), 26.38 (C-14), 24.19 (C-15$_{1/2}$), 24.14 (C-15$_{1/2}$).

^{1}H,^{1}H-COSY (400 MHz / 400 MHz, [D$_1$]-chloroform, 298 K) δ [in ppm] = 7.90/7.29 (H-6/H-7), 7.29/7.90, 7.48/7.43-7.39 (H-9/H-8), 7.43-7.39/7.48, 7.29 (H-8/H-9, 7), 7.29/7.90, ,7.43-7.39 (H-7/H-6, 8), 4.25/4.21 (H-22$_{1/2}$/H-22$_{1/2}$), 4.21/4.25 (H-22$_{1/2}$/H-22$_{1/2}$), 4.20-4.16/3.95-3.90, 3.78-3.74 (H-17$_{1/2}$/ H-17$_{1/2}$, H-18$_{1/2}$), 3.95-3.90/4.20-4.16, 3.78-3.74 (H-17$_{1/2}$/H-17$_{1/2}$, H-18$_{1/2}$), 3.78-3.74/4.20-4.16, 3.95-3.90 (H-18$_{1/2}$/ H-17$_{1/2}$), 3.69-3.52/1,31, 1.23 (H-14/H-15$_{1/2}$), 1.31/3.69-3.52 (H-15$_{1/2}$/H-14), 1.23/3.69-3.52 (H-15$_{1/2}$/H-14).

^{1}H,^{13}C-GHSQC (400 MHz / 101 MHz, [D$_1$]-chloroform, 298 K) δ (^{1}H) / δ (^{13}C) [in ppm] = 7.94/129.97 (H-4/C-4), 7.90/127.77 (H-6/C-6), 7.48/126.40 (H-9/C-9), 7.43-7.39/125.46 (H-12/C-12), 7.43-7.39/126.00 (H-8/C-8), 7.29/124.95 (H-7/C-7), 4.25/98.31 (H-22$_{1/2}$/C-22), 4.21/98.31 (H-22$_{1/2}$/C-22), 4.20-4.16/74.00 (H-17$_{1/2}$/C-17), 3.95-3.90/74.00 (H-17$_{1/2}$/C-17), 3.78-3.74/70.56 (H-18$_{1/2}$/C-18), 3.69-3.52/71.09, 70.83, 70.76 (H-19, 20, 21/C-19, 20, 21), 3.69-3.52/26.38 (H-14/C-14), 2.35/55.78 (H-23/C-23), 1.31/24.19 (H-15$_{1/2}$/C-15), 1.23/24.14 (H-15$_{1/2}$/C-15).

^{1}H,^{13}C-GHMBC (400 MHz / 101 MHz, [D_1]-chloroform, 298 K) δ (^{1}H) / δ (^{13}C) [in ppm] = 7.94/151.58, 135.20, 133.39, 127.77 (H-4/C-2, 11, 10, 6), 7.90/133.39, 129.97, 126.00 (H-6/C-10, 4, 8), 7.48/130.80, 124.95, 126.42 (H-9/C-5, 7, 1), 7.43-7.39/152.44, 135.74, 26.38 (H-12/C-16, 3, 14), 7.43-7.39/133.39, 127.77 (H-8/C-10, 6), 47.29/130.80, 126.40 (H-7/C-5, 9), .25/55.78 (H-$22_{1/2}$/C-23), 4.21/55.78 (H-$22_{1/2}$/C-23), 3.69-3.52/152.44, 141.63, 125.46, 24.19, 24.14 (H-14/C-16, 13, 12, 15), 2.35/98.31 (H-23/C-22), 1.31/141.63, 26.38 (H-$15_{1/2}$/C-13, 14), 1.23/141.63, 26.38 (H-$15_{1/2}$/C-13, 14).

MS (ESI-pos, MeOH): *m/z* = 951.5016 ($[M+Na]^+$, calcd. 951.5018 for $[C_{58}H_{72}O_{10}Na]^+$).

IR (ATR-FT): $\tilde{\nu}$ (cm^{-1}) = 750, 764, 1191, 1261, 1267, 1275, 2870, 2922, 2960, 2986, 3005.

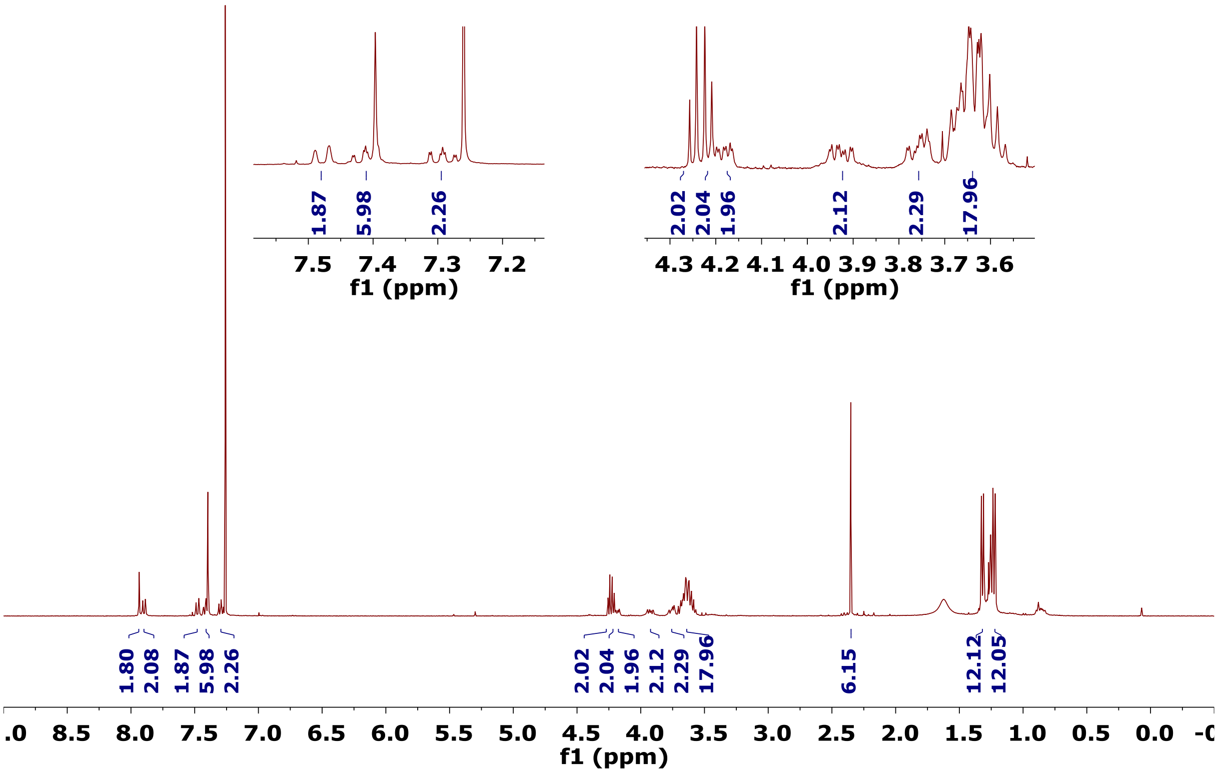

Figure S45: ^{1}H NMR spectrum of (*S*)- **iPr-M1$_5$** ($CDCl_3$, 298 K, 400 MHz).

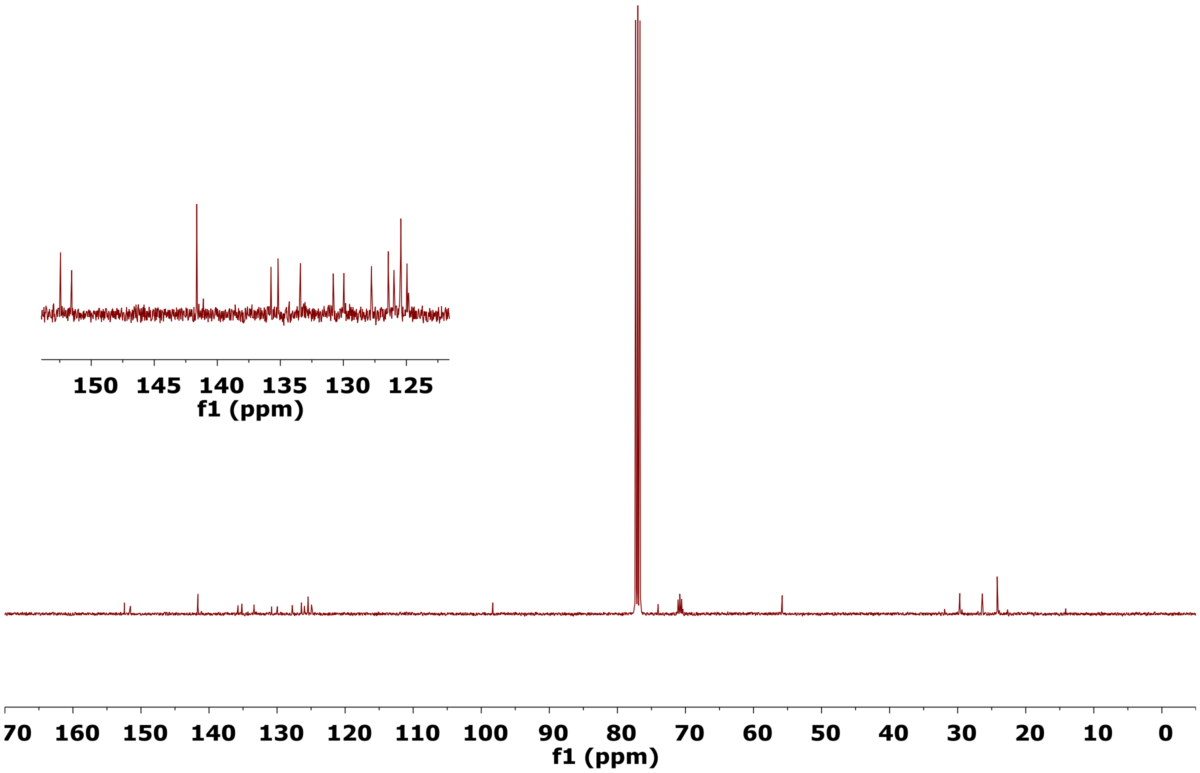

Figure S46: ^{13}C NMR spectrum of (*S*)- **iPr-M1$_5$** ($CDCl_3$, 298 K, 400 MHz).

Compound **iPr-M1$_6$**

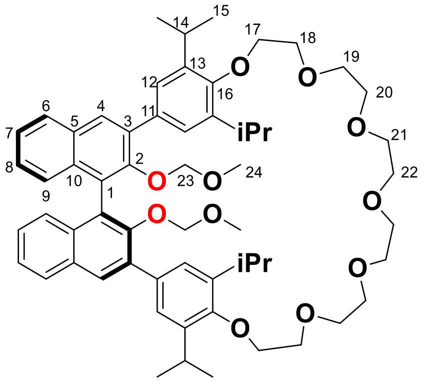

As described above (General procedure **B**), compound **iPr-M1$_6$** was synthesized using compound **iPr-2** (58.2 mg, 0.0800 mmol, 1.0 equiv), cesium carbonate (56.1mg, 172 µmol, 2.5 equiv) and **8$_6$** (49.9 mg, 0.0845 mmol, 1.0 equiv) in degassed acetonitrile (80 mL). After purification by column chromatography (cyclohexane/ethyl acetate 3:1) the product was obtained as a yellow wax (32.6 mg, 0.0334 mmol, 41.2% yield).

$C_{60}H_{76}O_{11}$: 973.76 g/mol.

^{1}H-NMR (400 MHz, [D1]-chloroform, 298 K) δ [in ppm] = 7.96 (s, 2H, H-4), 7.91 (d, *J* = 8.2 Hz, 2H, H-6), 7.56 (s, 4H, H-12), 7.47 (d, *J* = 8.4 Hz, 2H, H-9), 7.41 (dd, *J* = 6.9 Hz, *J* = 1.2 Hz, 2H, H-7), 7.28 (dd, *J* = 7.0 Hz, *J* = 1.2 Hz, 2H, H-8), 4.23 (d, *J* = 6.3 Hz, 2H, H-$23_{1/2}$), 4.21 (d, *J* = 6.3 Hz, 2H, H-$23_{1/2}$), 4.09-3.95 (m, 4H, H-17), 3.83-3.81 (m, 4H, H-18), 3.75-3.69 (m, 16H, H-19, 20, 21, 22), 3.64 (hept., *J* = 6.8 Hz, 4H, H-14), 2.24 (s, 6H, H-24), 1.37 (d, *J* = 6.9 Hz, 12H, H-$15_{1/2}$), 1.27 (d, *J* = 6.9 Hz, 12H, H-$15_{1/2}$).

^{13}C-NMR (101 MHz, [D1]-chloroform, 298 K) δ [in ppm] = 152.55 (C-16), 152.28 (C-2), 141.85 (C-13), 135.81 (C-3), 135.01 (C-11), 132.85 (C-10), 130.96 (C-5), 129.24 (C-4), 128.05 (C-6), 126.32 (C-1), 126.27 (C-9), 126.04 (C-8), 125.42 (C-12), 124.83 (C-7), 98.71 (C-23), 74.49 (C-17), 71.24, 70.93, 70.69, 70.38, 70.25 (C-18, 19, 20, 21, 22), 55.60 (C-24), 25.96 (C-14), 24.66 (C-$15_{1/2}$), 24.14 (C-$15_{1/2}$).

^{1}H,^{1}H-COSY (400 MHz / 400 MHz, [D1]-chloroform, 298 K) δ [in ppm] =7.91/7.41 (H-6/H-7), 7.47/7.28 (H-9/H-8), 7.41/7.91, 7.28 (H-7/H-6, 8), 7.28/7.47, 7.41 (H-8/H-9, 7), 4.23/4.21 (H-$23_{1/2}$/H-$23_{1/2}$), 4.21/4.23 (H-$23_{1/2}$/H-$23_{1/2}$), 4.09-3.95/3.83-3.81 (H-17/H-18), 3.83-3.81/4.09-3.95 (H-18/H-17), 3.64/1.37, 1.27 (H-14/H-$15_{1/2}$), 1.37/3.64 (H-$15_{1/2}$/H-14), 1.27/3.64 (H-$15_{1/2}$/H-14).

^{1}H,^{13}C-GHSQC (400 MHz / 101 MHz, [D1]-chloroform, 298 K) δ (1H) / δ (13C) [in ppm] = 7.96/129.24 (H-4/C-4), 7.91/128.05 (H-6/C-6), 7.56/125.42 (H-12/C-12), 7.47/126.27 (H-9/C-9), 7.41/124.83 (H-7/C-7), 7.28/126.04 (H-8/C-8), 4.23/98.71 (H-$23_{1/2}$/C-23), 4.21/98.71 (H-$23_{1/2}$/C-23), 4.09-3.95/74.49 (H-17/C-17), 3.83-3.81/70.69 (H-18/C-18), 3.75-3.69/71.24, 70.93, 70.38, 70.25 (H-19, 20, 21, 22/C- 19, 20, 21, 22), 3.64/25.96 (H-14/C-14), 2.24/55.60 (H-24/C-24), 1.37/24.66 (H-$15_{1/2}$/ C-$15_{1/2}$), 1.27/24.14 (H-$15_{1/2}$/C-$15_{1/2}$).

^{1}H,^{13}C-GHMBC (400 MHz / 101 MHz, [D1]-chloroform, 298 K) δ (1H) / δ (13C) [in ppm] = 7.96/152.28, 135.01, 132.85, 128.05 (H-4/C-2, 11, 10, 6), 7.91/132.85, 129.24, 126.04 (H-6/C-10, 4, 8), 7.56/152.55, 135.81, 25.96 (H-12/C-16, 3, 14), 7.47/130.96, 124.83, 126.32 (H-9/C-5, 7, 1), 7.41/130.96, 126.27 (H-7/C-5, 9), 7.28/132.85, 128.05 (H-8/C-10, 6), 4.23/55.60 (H-$23_{1/2}$/C-24), 4.21/55.60 (H-$23_{1/2}$/C-24), 3.64/152.55, 141.85, 125.42, 24.66, 24.14 (H-14/C-16, 13, 12, C-$15_{1/2}$), 2.24/98.71 (H-24/C-23), 1.37/141.85, 25.96 (H-$15_{1/2}$/C-13, 14), 1.27/141.85, 25.96 (H-$15_{1/2}$/C-13, 14).

MS (ESI-pos, MeOH): *m/z* = 995.5287 ($[M+Na]^+$, calcd. 995.5280 for $[C_{60}H_{76}O_{11}Na]^+$).

IR (ATR-FT): $\tilde{\nu}$ (cm^{-1}) = 750, 763, 980, 1073, 1177, 1194, 1267, 1456, 1770, 2884, 2992, 3053.

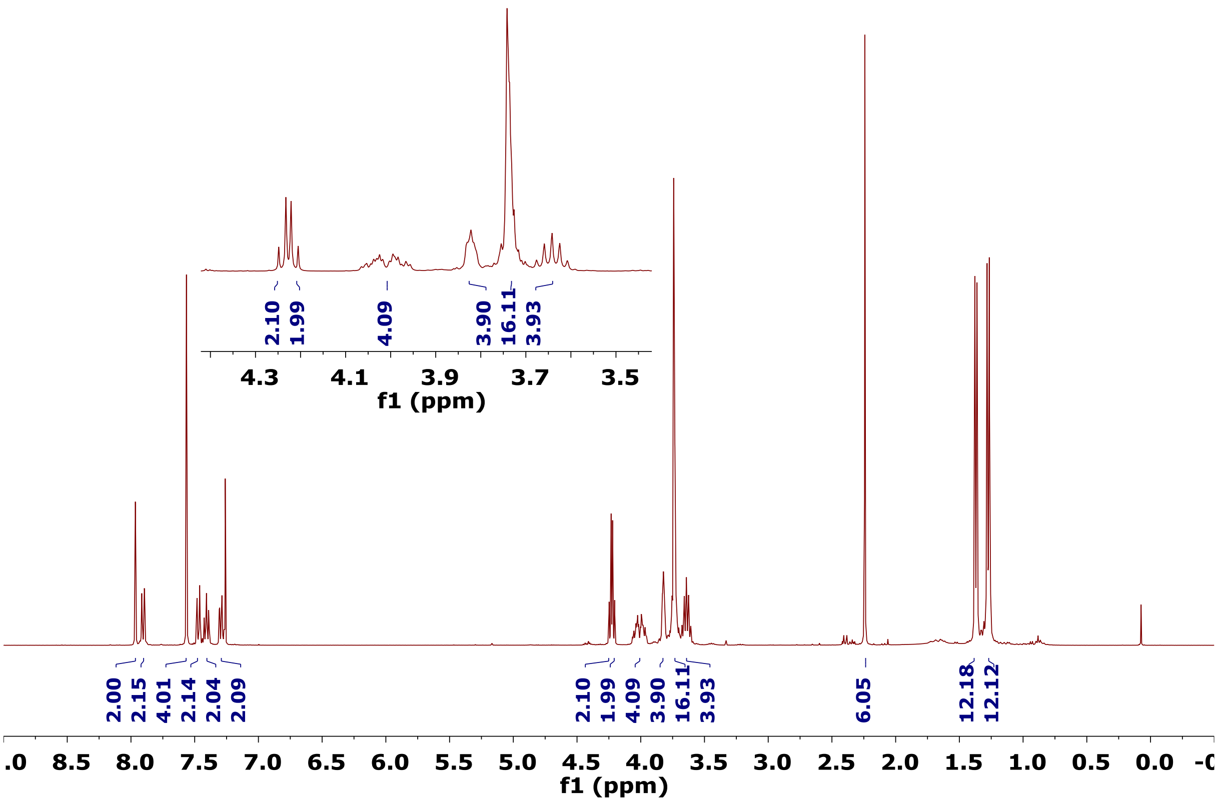

Figure S47: ^{1}H NMR spectrum of (*S*)- **iPr-M1$_6$** ($CDCl_3$, 298 K, 400 MHz).

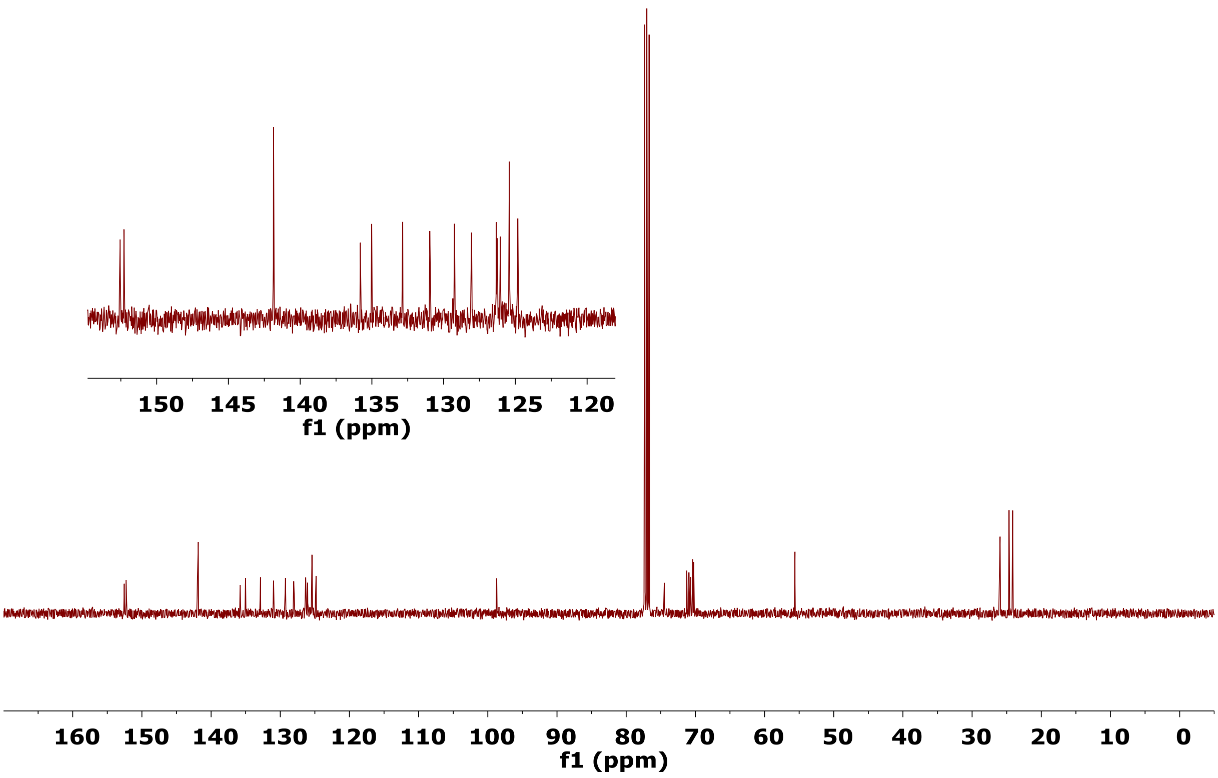

Figure S48: ^{13}C NMR spectrum of (*S*)- **iPr-M1$_6$** ($CDCl_3$, 298 K, 400 MHz).

Compound **iPr-M1$_7$**

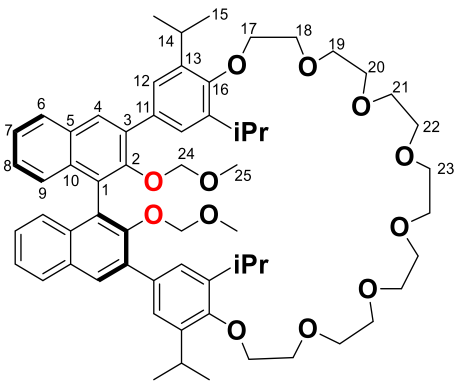

As described above (General procedure **B**), compound **iPr-M1$_7$** was synthesized using compound **iPr-2** (48.0 mg, 0.0660 mmol, 1.0 equiv), cesium carbonate (48.1 mg, 147 µmol, 2.2 equiv) and **8$_7$** (42.9 mg, 0.0676 mmol, 1.0 equiv) in degassed acetonitrile (66 mL). After purification by column chromatography (cyclohexane/ethyl acetate 3:2) the product was obtained as a white solid (30.9 mg, 0.0304 mmol, 45.5% yield).

$C_{62}H_{80}O_{12}$: 1017.31 g/mol.

^{1}H-NMR (400 MHz, [D$_1$]-chloroform, 298 K) δ [in ppm] = 7.96 (s, 2H, H-4), 7.90 (d, *J* = 8.2 Hz, 2H, H-6), 7.56 (s, 4H, H-12), 7.43 (d, *J* = 8.4 Hz, 2H, H-9), 7.41 (dd, *J* = 6.9 Hz, *J* = 1.2 Hz, 2H, H-7), 7.28 (dd, *J* = 7.0 Hz, *J* = 1.2 Hz, 2H, H-8), 4.23 (bs, 4H, H-24), 4.00-3.95 (m, 4H, H-17), 3.88-3.81 (m, 4H, H-18), 3.78-3.69 (m, 20H, H-19, 20, 21, 22, 23), 3.55 (hept., *J* = 6.8 Hz, 4H, H-14), 2.21 (s, 6H, H-25), 1.35 (d, *J* = 6.8, 12H, H-15$_{1/2}$), 1.27 (d, *J* = 6.8, 12H, H-15$_{1/2}$).

^{13}C-NMR (101 MHz, [D$_1$]-chloroform, 298 K) δ [in ppm] = 152.43 (C-16), 152.33 (C-2), 141.89 (C-13), 135.90 (C-3), 135.18 (C-11), 133.14 (C-10), 131.09 (C-5), 129.53 (C-4), 128.15 (C-6), 126.51 (C-1), 126.45 (C-9), 126.20 (C-8), 125.61 (C-12), 125.01 (C-7), 98.75 (C-24), 74.49 (C-17), 71.59, 71.14, 70.91, 70.75, 70.58 (C-19, 20, 21, 22, 23), 70.78 (C-18), 55.75 (C-25), 26.26 (C-14), 24.50 (C-15$_{1/2}$), 24.31(C-15$_{1/2}$).

^{1}H,^{1}H-COSY (400 MHz / 400 MHz, [D$_1$]-chloroform, 298 K) δ [in ppm] =7.90/7.41 (H-6/H-7), 7.43/7.28 (H-9/H-8), 7.41/7.90, 7.28 (H-7/H-6, H-8), 7.28/7.43, 7.41 (H-8/H-9, 7), 4.00-3.95/3.88-3.81 (H-17/H-18), 3.88-3.81/4.00-3.95 (H-18/H-17), 3.55/1.35, 1.27 (H-14/H-15$_{1/2}$), 1.35/3.55 (H-15$_{1/2}$/H-14), 1.27/3.55 (H-15$_{1/2}$/H-14).

^{1}H,^{13}C-GHSQC (400 MHz / 101 MHz, [D$_1$]-chloroform, 298 K) δ (1H) / δ (^{13}C) [in ppm] = 7.96/129.53 (H-4/C-4), 7.90/128.15 (H-6/C-6), 7.56/125.61 (H-12/C-12), 7.43/126.45 (H-9/C-9), 7.41/125.01 (H-7/C-7), 7.28/126.20 (H-8/C-8), 4.25/98.75 (H-24$_{1/2}$/C-24), 4.22/98.75 (H-24$_{1/2}$/C-24), 4.00-3.95/74.49 (H-17/C-17), 3.88-3.81/70.78 (H-18/C-18), 3.78-3.69/71.59, 71.14, 70.91, 70.75, 70.58 (H-19, 20, 21, 22, 23/C-19, 20, 21, 22, 23), 3.55/26.26 (H-14/C-14), 2.21/55.75 (H-25/C-25), 1.35/24.50 (H-15$_{1/2}$/ C-15$_{1/2}$), 1.27/24.31 (H-15$_{1/2}$/C-15$_{1/2}$).

^{1}H,^{13}C-GHMBC (400 MHz / 101 MHz, [D$_1$]-chloroform, 298 K) δ (^{1}H) / δ (^{13}C) [in ppm] = 7.96/152.33, 135.18, 133.14, 128.15 (H-4/C-2, 11, 10, 6), 7.90/133.14, 129.53, 126.20 (H-6/C-10, 4, 8), 7.56/152.43, 135.90, 26.26 (H-12/C-16, 3, 14), 7.43/131.09, 126.51, 125.01 (H-9/C-5, 1, 7), 7.41/131.09, 126.45 (H-7/C-5, 9), 7.28/133.14, 128.15 (H-8/C-10, 6), 4.23/152.33, 55.75 (H-24C-2, 25) 3.55/152.43, 141.89, 125.61, 24.50, 24.31 (H-14/C-16, 13, 12, C-15$_{1/2}$), 2.21/98.75 (H-25/C-24), 1.35/141.89, 26.26 (H-15$_{1/2}$/C-13, 14), 1.27/141.89, 26.26 (H-15$_{1/2}$/C-13, 14).

MS (ESI-pos, MeOH): *m/z* = 1039.5535 ([M+Na]$^+$, calcd. 1039.5542 for [$C_{62}H_{80}O_{12}Na$]$^+$).

IR (ATR-FT): $\tilde{\nu}$ (cm^{-1}) = 750, 763, 882, 1073, 1096, 1148, 1195, 1262, 1457, 1867, 2959, 3052.

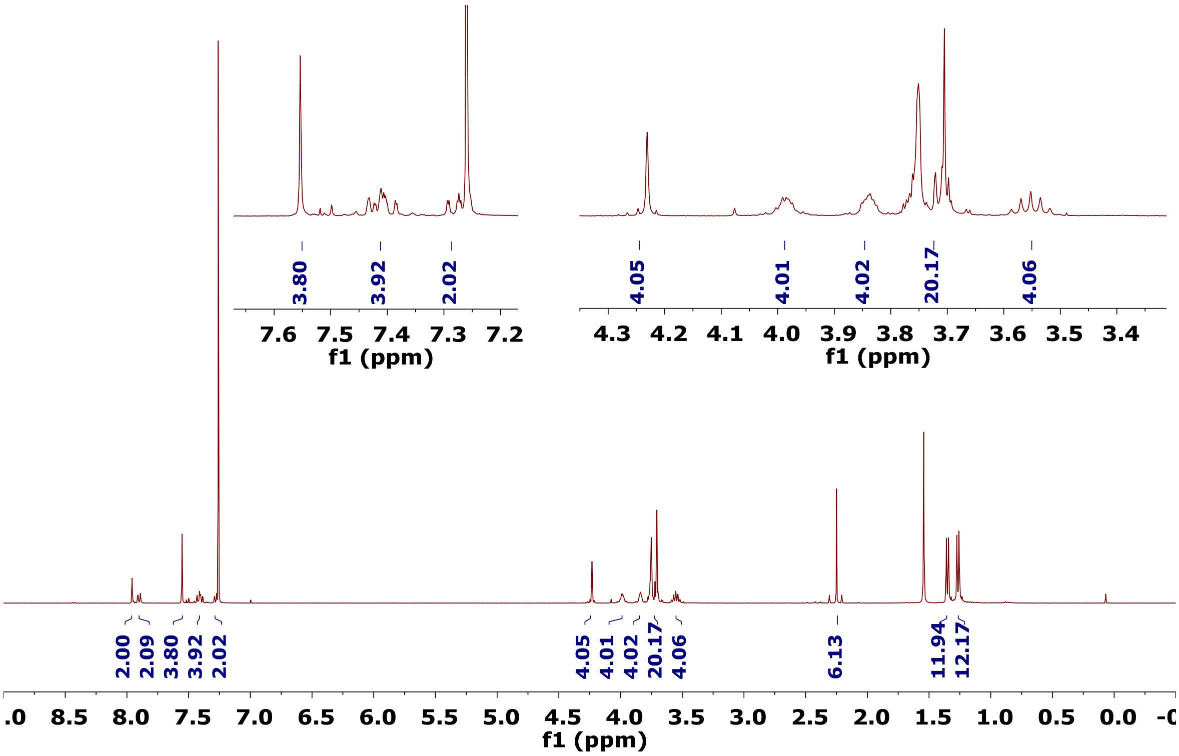

Figure S49: ^{1}H NMR spectrum of (*S*)- **iPr-M1$_7$** ($CDCl_3$, 298 K, 400 MHz).

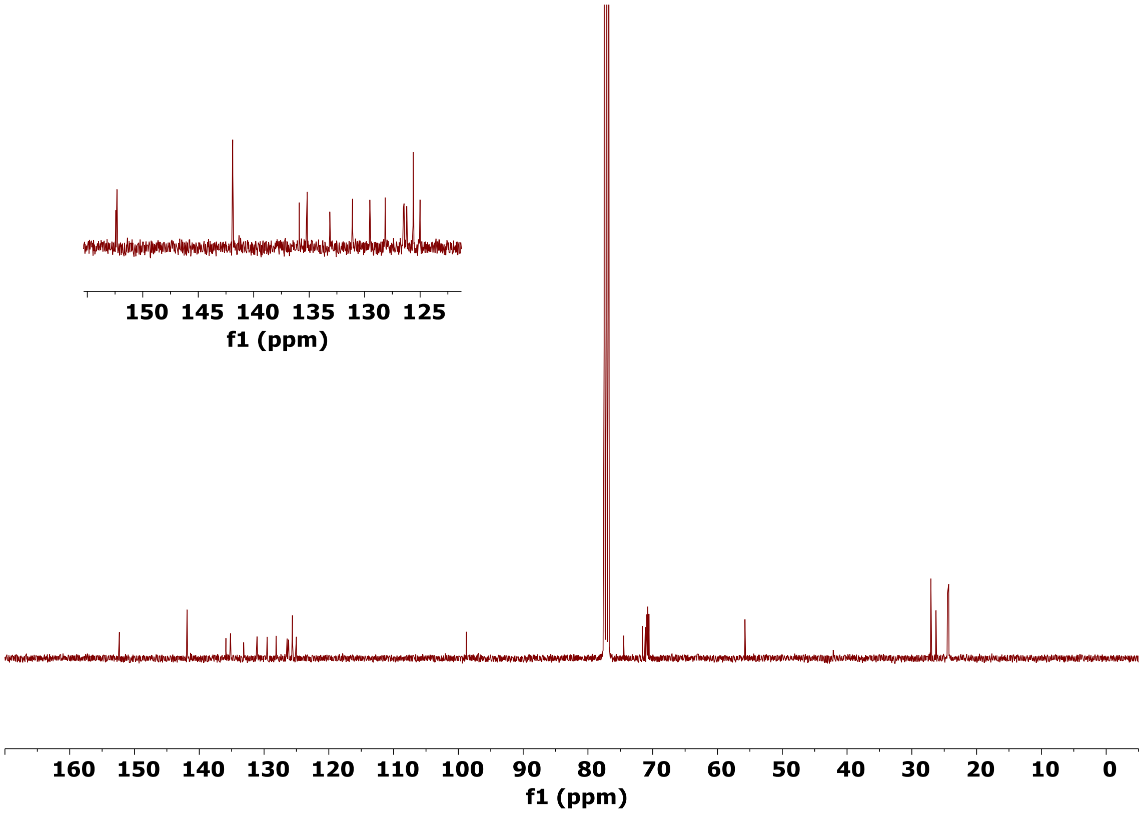

Figure S50: ^{13}C NMR spectrum of (*S*)- **iPr-M1$_7$** ($CDCl_3$, 298 K, 400 MHz).

Compound **iPr-M1$_8$**

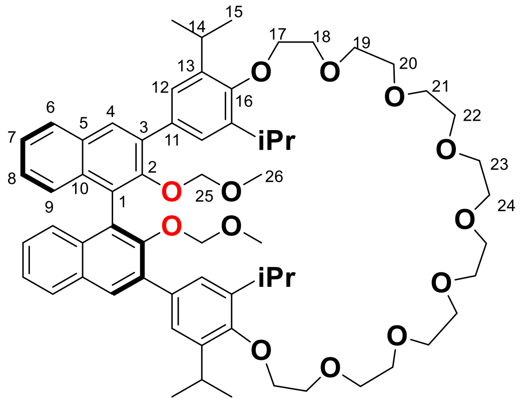

As described above (General procedure **B**), compound **iPr-M1$_8$** was synthesized using compound **iPr-2** (97.9 mg, 0.135 mmol, 1.0 equiv), cesium carbonate (114 mg, 0.350 mmol, 2.5 equiv) and **8$_8$** (94.1 mg, 138 µmol, 1.2 equiv) in degassed acetonitrile (140 mL). After purification by column chromatography (cyclohexane/ethyl acetate 2:1) the product was obtained as a clear wax (75.9 mg, 0.0715 mmol, 53.3% yield).

$C_{64}H_{84}O_{13}$: 1061.36 g/mol.

^{1}H-NMR (400 MHz, [D$_1$]-chloroform, 298 K) δ [in ppm] = 7.85 (s, 2H, H-4), 7.79 (d, *J* = 8.1 Hz, 2H, H-6), 7.43 (s, 4H, H-12), 7.30-7.27 (m, 2H, H-7), 7.26 (d, *J* = 8.2 Hz, 2H, H-9), 7.17-7.14 (m, 2H, H-8), 4.19 (d, *J* = 6.0 Hz, 2H, H-25$_{1/2}$), 4.15 (d, *J* = 6.0 Hz, 2H, H-25$_{1/2}$), 3.90-3.86 (m, 4H, H-17), 3.76-3.73 (m, 4H, H-18), 3.65-3.62 (m, 12h, H-19, 20, 21), 3.59-3.55 (m, 12h, H-22, 23, 24), 3.41 (hept., *J* = 6.9 Hz, 4H, H-14), 2.19 (s, 6H, H-26), 1.23 (d, *J* = 6.8, 12H, H-15$_{1/2}$), 1.16 (d, *J* = 6.8, 12H, H-15$_{1/2}$).

^{13}C-NMR (101 MHz, [D$_1$]-chloroform, 298 K) δ [in ppm] = 152.40 (C-16), 152.18 (C-2), 141.80 (C-13), 135.87 (C-3), 135.18 (C-11), 133.27 (C-10), 131.01 (C-5), 129.66 (C-4), 128.06 (C-6), 126.49 (C-9), 126.46 (C-1), 126.15 (C-8), 125.60 (C-12), 125.00 (C-7), 98.60 (C-25), 74.34 (C-17), 71.46, 71.00, 70.92, 70.76, 70.74, 70.70, 70.65 (C-18, 19, 20, 21, 22, 23), 55.76 (C-26), 26.34 (C-14), 24.37 (C-15$_{1/2}$), 24.29 (C-15$_{1/2}$).

^{1}H,^{1}H-COSY (400 MHz / 400 MHz, [D1]-chloroform, 298 K) δ [in ppm] = 7.79/7.30-7.27 (H-6/H-7), 7.30-7.27/7.79, 7.17-7.14 (H-7/H-6, 8), 7.17-7.14/7.30-7.27 (H-8/H-7), 4.19/4.15 (H-25$_{1/2}$/H-25$_{1/2}$), 3.41/1.23, 1.16 (H-14/ H-15$_{1/2}$).

^{1}H,^{13}C-GHSQC (400 MHz / 101 MHz, [D$_1$]-chloroform, 298 K) δ (^{1}H) / δ (^{13}C) [in ppm] = 7.85/129.66 (H-4/C-4), 7.79/128.06 (H-6/C-6), 7.43/125.60 (H-12/C-12), 7.30-7.27/125.00 (H-7/C-7), 7.26/126.49 (H-9/C-9), 7.17-7.14/126.15 (H-8/C-8), 4.19/98.59 (H-25$_{1/2}$/C-25), 4.15/98.59 (H-25$_{1/2}$/C-25), 3.90-3.86/74.34 (H-17/C-17), 3.76-3.55/71.46, 71.00, 70.92, 70.76, 70.74, 70.70, 70.65 (H-18, 19, 20, 21, 22, 23/C-18, 19, 20, 21, 22, 23), 3.41/26.34 (H-14/C-14), 2.19/55.76 (H-26/C-26), 1.23/24.37 (H-15$_{1/2}$/C-15$_{1/2}$), 1.16/24.29 (H-15$_{1/2}$/C-15$_{1/2}$).

^{1}H,^{13}C-GHMBC (400 MHz / 101 MHz, [D$_1$]-chloroform, 298 K) δ (^{1}H) / δ (^{13}C) [in ppm] = 7.85/152.18, 135.18, 133.27, 128.06 (H-4/C-2, 11, 10, 6), 7.79/133.27, 129.66, 126.15 (H-6/C-10, 4, 8), 7.43/152.40, 141.80, 135.87, 26.34 (H-12/C-16, 13, 3, 14), 7.30-7.27/131.01, 126.49 (H-7/C-5, 9), 7.26/131.01, 126.46, 125.00 (H-9/C-5, 1, 7), 7.17-7.14/133.27, 128.06 (H-8/C-10, 6), 4.19/152.18, 55.76 (H-25$_{1/2}$/C-2, 26), 4.15/152.18, 55.76 (H-25$_{1/2}$/C-2, 26), 3.41/152.40, 141.80, 125.60, 24.37, 24.29 (H-14/C-16, 13, 12, 15$_{1/2}$), 2.19/98.59 (H-26/C-25), 1.23/141.80 (H-15$_{1/2}$/C-13), 1.16/141.80 (H-15$_{1/2}$/ C-13).

MS (ESI-pos, MeOH): *m/z* = 1083.5808 ([M+Na]$^+$, calcd. 1083.5804 for [$C_{64}H_{84}O_{13}Na$]$^+$).

IR (ATR-FT): $\tilde{\nu}$ (cm^{-1}) = 701, 733, 750, 756, 940, 1096, 1147, 1195, 1468, 2868, 2992, 3051.

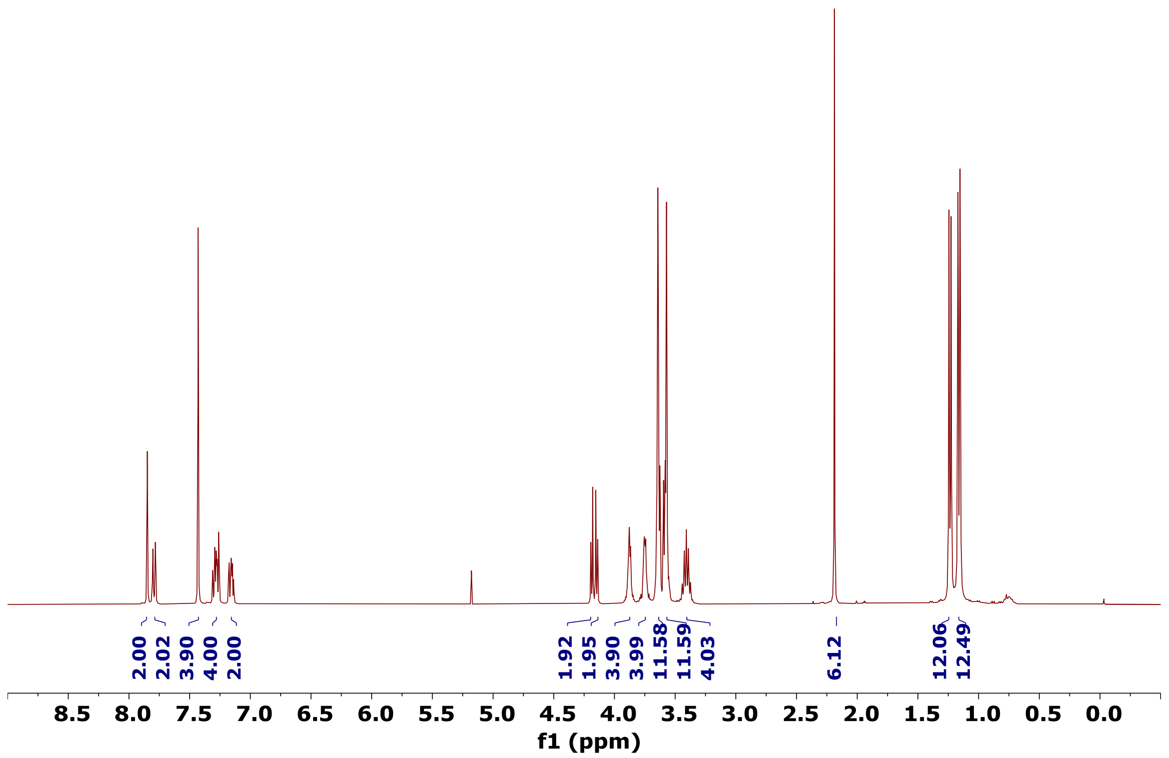

Figure S51: ^{1}H NMR spectrum of (*S*)- **iPr-M1$_8$** ($CDCl_3$, 298 K, 400 MHz).

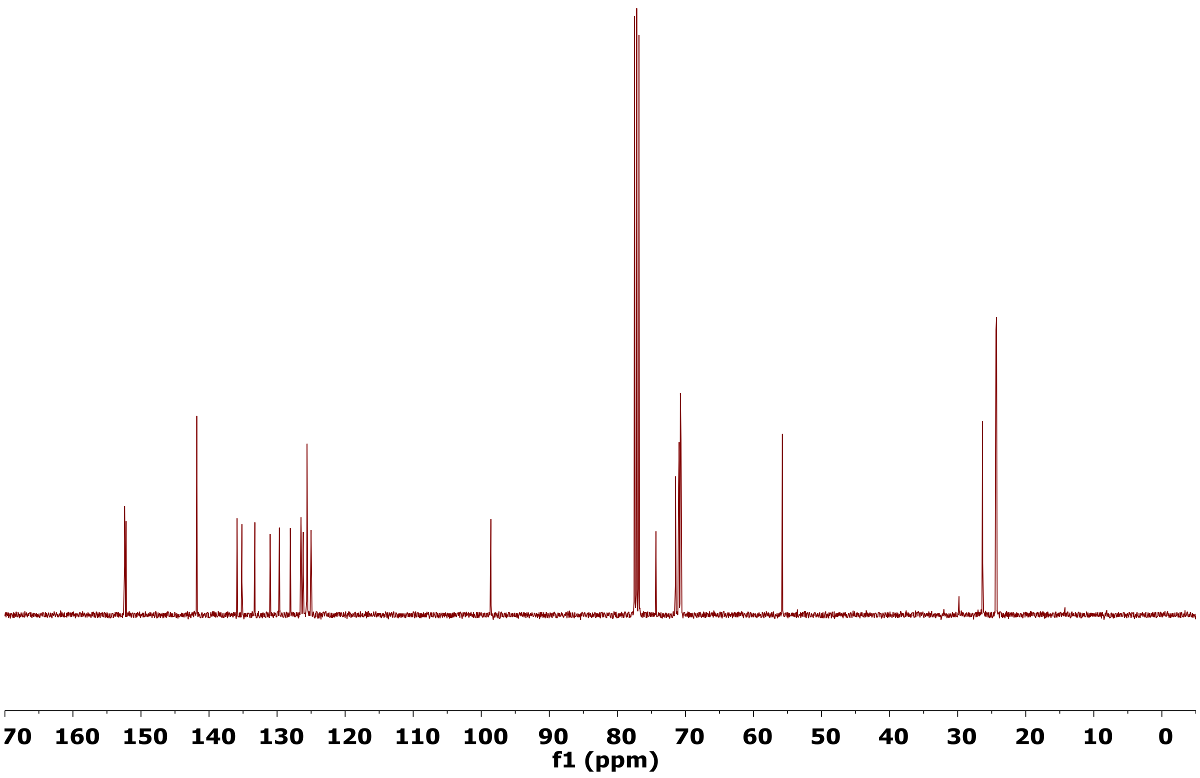

Figure S52: ^{13}C NMR spectrum of (*S* - **iPr-M1$_8$** ($CDCl_3$, 298 K, 400 MHz).

2.5. Bis-BINOL macrocycles

Compound **Me-M2$_6$**

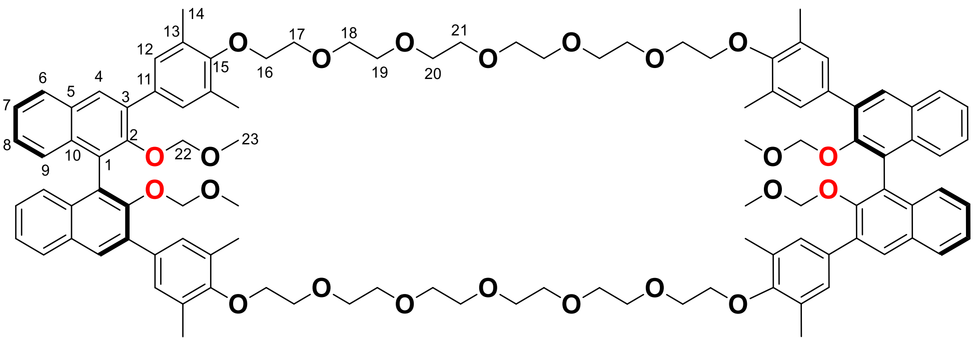

As described above (General procedure **C**), compound **Me-M2$_6$** was synthesized using compound **Me-2** (8.8 mg, 0.0144 mmol, 1.0 equiv), cesium carbonate (15.0 mg, 0.0461 mmol, 3.2 equiv) and **Me-3$_6$** (20.9 mg, 0.0144 mmol, 1.0 equiv) dissolved in degassed acetonitrile (1 mL). After purification by column chromatography (cyclohexane/ethyl aetate 1:2) the product was obtained as a white solid (15.3 mg, 0.0089 mmol, 61.8 %yield).

$C_{104}H_{120}O_{22}$: 1722.08 g/mol.

^{1}H-NMR (400 MHz, [D$_6$]-dimethyl sulfoxide, 298 K) δ [in ppm] = 8.00 (s, 4H, H-4), 7.98 (d, *J* = 8.5 Hz, 4H, H-6), 7.42 (t, *J* = 7.2 Hz, 4H, H-7), 7.30 (s, 8H, H-12), 7.28 (t, *J* = 7.6 Hz, 4 H, H-8), 7.04 (d, *J* = 8.6 Hz, 4H, H-9), 4.32 (d, *J* = 5.5 Hz, 4H, H-22$_{1/2}$), 4.23 (d, *J* = 5.5 Hz, 4H, H-22$_{1/2}$), 3.90 – 3.88 (m, 8H, H-16), 3.71 – 3.69 (m, 8H, H-17), 3.60 – 3.58 (m, 8H, H-18), 3.56 – 3.54 (m, 8H, H-19), 3.54 – 3.48 (m, 16H, H20+21), 2.25 (s, 36 H, H-14+23).

The spectroscopic data is in agreement with the literature.[2]

Compound **H-M2$_6$**

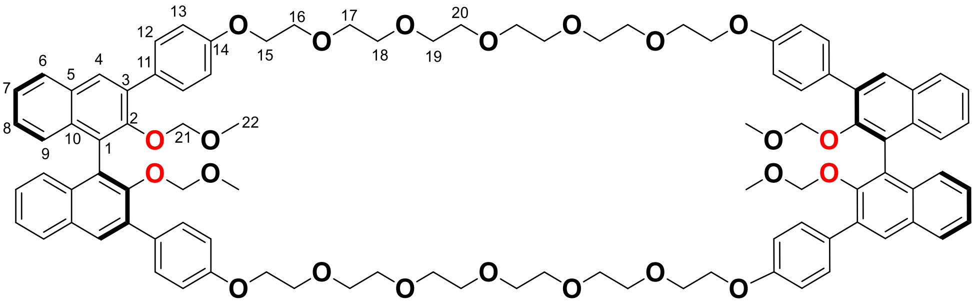

As described above (General procedure **C**), compound **H-M2$_6$** was synthesized using compound **H-2** (20.0 mg, 0.0358 mmol, 1.0 equiv), cesium carbonate (41.9 mg, 129 µmol, 3.2 equiv) and **H-3$_6$** (56.3 mg, 0.0403 mmol, 1.0 equiv) in degassed acetonitrile (50 mL). After purification by column chromatography (methanol/dichloromethane 1.50) the product was obtained as pale-yellow oil (38.9 mg, 0.0242 mmol, 66.7% yield).

$C_{96}H_{104}O_{22}$: 1609.87 g/mol.

^{1}H-NMR (400 MHz, [D$_6$]-dimethyl sulfoxide, 298 K) δ [in ppm] = 8.02 (s, 4H, H-4), 7.99 (d, *J* = 8.2 Hz, 4H, H-6), 7.60 (d, *J* = 8.8 Hz, 8H, H-12), 7.43 (t, *J* = 7.2 Hz, 4H, H-7), 7.29 (t, *J* = 7.3 Hz, 4H, H-8), 7.08 (d, *J* = 8.0 Hz, 4H, H-9), 7.04 (d, *J* = 8.8 Hz, 8H, H-13), 4.30 (d, *J* = 5.4 Hz, 4H, H-$21_{1/2}$), 4.21 (d, *J* = 5.4 Hz, 4H, H-$21_{1/2}$), 4.11 (t, *J* = 4.3 Hz, 8H, H-15), 3.74 (t, *J* = 4.3 Hz, 8H, H-16), 3.59-3.51 (m, 32H, H-17, 18, 19, 20), 2.23 (s, 12H, H-22).

^{13}C-NMR (101 MHz, [D$_6$]-dimethyl sulfoxide, 298 K) δ [in ppm] = 157.92 (C-14), 150.60 (C-2), 134.49 (C-3), 132.65 (C-10), 130.58 (C-11), 130.51 (C-5), 130.34 (C-12), 129.92 (C-4), 128.00 (C-6), 126.24 (C-8), 125.86 (C-1), 125.62 (C-9), 125.10 (C-7), 114.39 (C-13), 97.49 (C-21), 69.98, 69.85, 69.83, 69.81(C-17, 18, 19, 20), 68.94 (C-16), 67.17 (C-15), 55.17 (C-22),

^{1}H,^{1}H-COSY (400 MHz / 400 MHz, [D$_6$]-dimethyl sulfoxide, 298 K) δ [in ppm] = 7.99/7.43 (H-6/H-7), 7.60/7.04 (H-12/H-13), 7.43/7.99, 7.29 (H-7/H-6, 8), 7.29/7.43, 7.08 (H-8/H-7, 9), 7.08/7.29 (H-9/H-8), 7.04/7.60 (H-13/H-12), 4.30/4.21 (H-$21_{1/2}$/ H-$21_{1/2}$), 4.21/4.30 (H-$21_{1/2}$/ H-$21_{1/2}$), 4.11/3.74 (H-15/H-16), 3.74/4.11 (H-16/H-15).

^{1}H,^{13}C-GHSQC (400 MHz / 101 MHz, [D$_6$]-dimethyl sulfoxide, 298 K) δ (^{1}H) / δ (^{13}C) [in ppm] = 8.02/129.92 (H-4/C-4), 7.99/128.00 (H-6/C-6), 7.60/130.34 (H-12/C-12), 7.43/125.10 (H-7/C-7), 7.29/126.24 (H-8/C-8), 7.08/125.62 (H-9/C-9), 7.04/114.39 (H-13/C-13), 4.30/97.49 (H-$21_{1/2}$/C-21), 4.21/97.49 (H-$21_{1/2}$/C-21), 4.11/67.17 (H-15/C-15), 3.74/68.94 (H-16/C-16), 2.23/55.17 (H-22/C-22).

^{1}H,^{13}C-GHMBC (400 MHz / 101 MHz, [D$_6$]-dimethyl sulfoxide, 298 K) δ (^{1}H) / δ (^{13}C) [in ppm] = 8.02/150.60, 132.65, 130.58, 128.00 (H-4/C-2, 10, 11, 6), 7.99/132.65, 129.92, 126.24 (H-6/C-10, 4, 8), 7.60/157.92, 134.49 (H-12/C-14, 3), 7.43/130.51, 125.62 (H-7/C-5, 9), 7.29/132.65, 128.00 (H-8/C-10, 6), 7.08/130.51, 125.86, 125.10 (H-9/C-5, 1, 7), 7.04/157.92, 130.58 (H-13/C-14, 11), 4.30/150.60, 55.17 (H-$21_{1/2}$/ C-2, 22), 4.21/150.60, 55.17 (H-$21_{1/2}$/ C-2, 22), 2.23/97.49 (H-22/C-21).

MS (ESI-pos, MeOH): *m/z* = 1632.6984 ([M+Na]$^+$, calcd. 1632.6945 [$C_{96}H_{104}O_{22}Na$]$^+$).

IR (ATR-FT): $\tilde{\nu}$ (cm^{-1}) = 702, 734, 749, 764, 898, 1079, 1097, 1177, 1263, 1275, 1361, 1512, 1606, 2827, 2998, 3004, 3054.

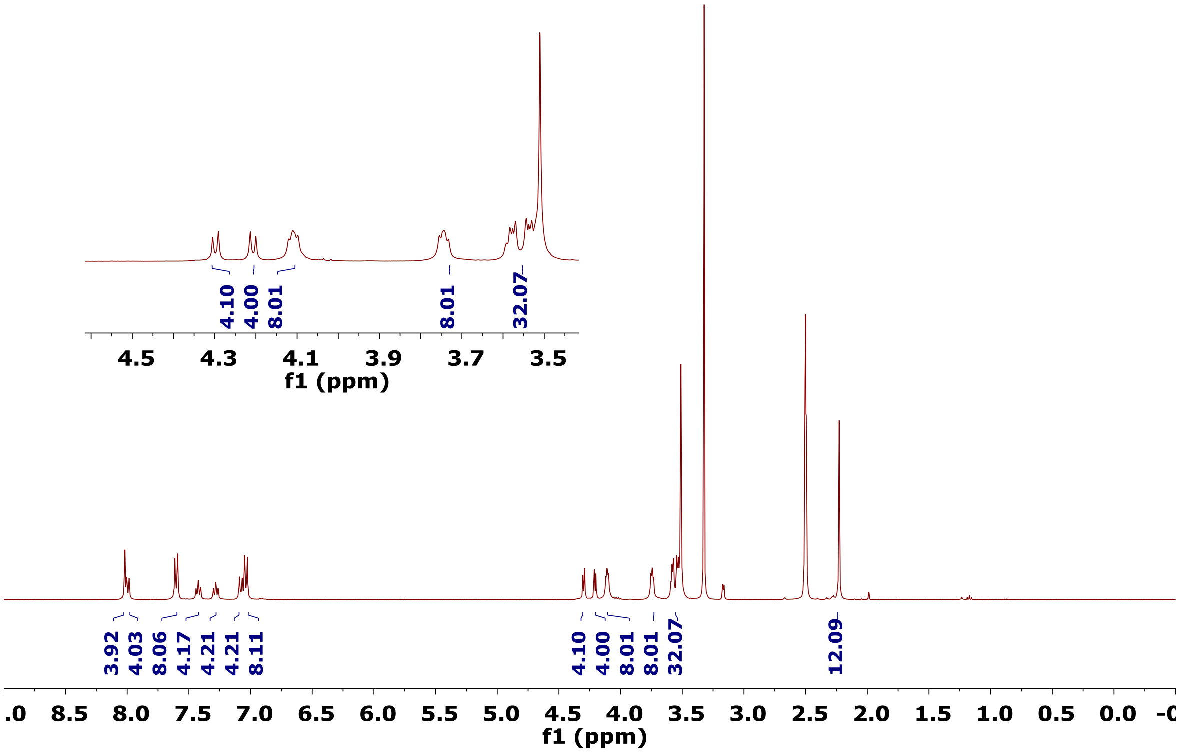

Figure S53: ^{1}H NMR spectrum of (*S,S*)-**H-M2$_6$** (DMSO-*d$_6$*, 298 K, 400 MHz).

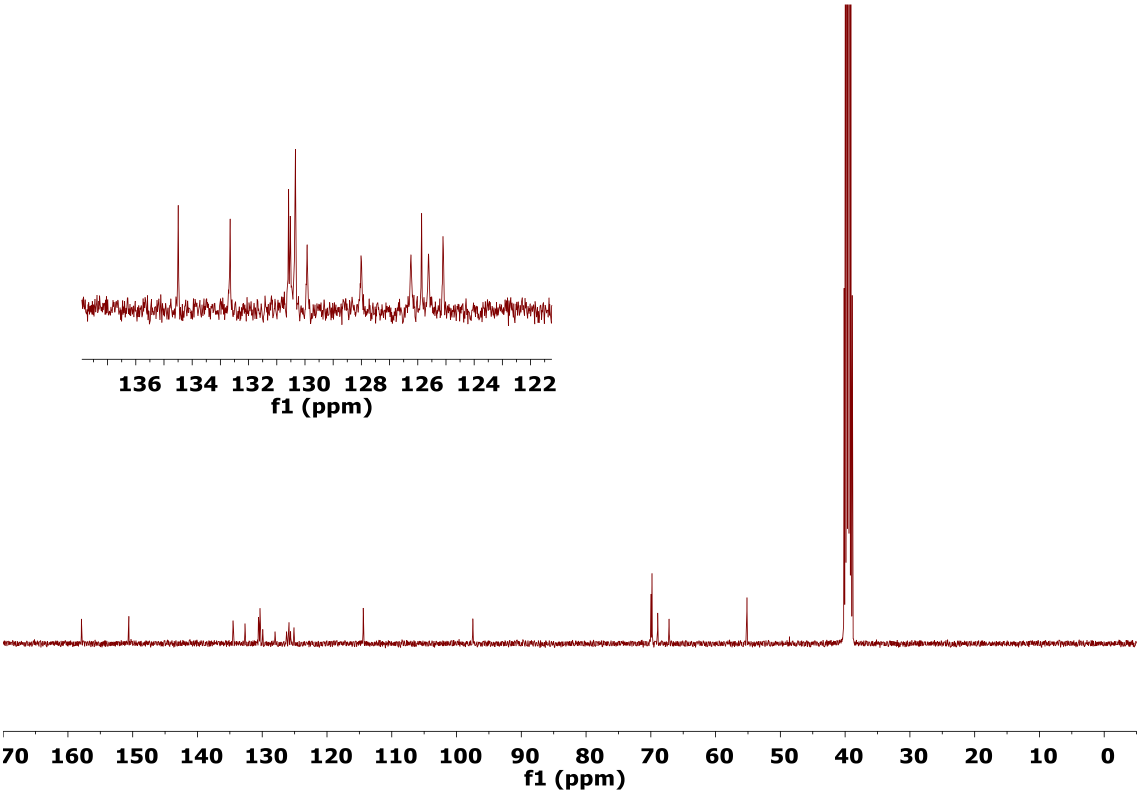

Figure S54: ^{13}C NMR spectrum of (*S,S*)-**H-M2$_6$** (DMSO-*d$_6$*, 298 K, 400 MHz).

Compound **iPr-M2$_6$**

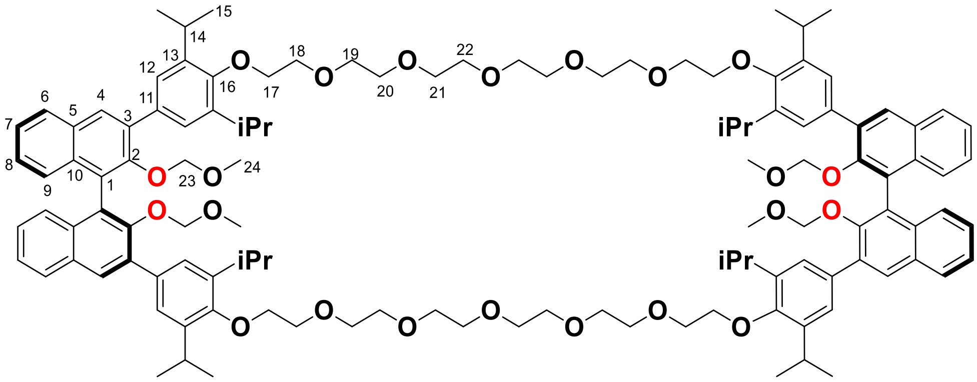

As described above (General procedure **C**), compound **iPr-M2$_6$** was synthesized using compound **iPr-2** (10.2 mg, 0.0140 mmol, 1.0 equiv), cesium carbonate (10.5 mg, 0.0322 mmol, 3.2 equiv) and **iPr-3$_6$** (22.1 mg, 0.0141 mmol, 1.0 equiv) dissolved in degassed acetonitrile (20 mL). After purification by column chromatography (methanol/dichloromethane 1:50) the product was obtained as a yellow wax (14.7 mg, 0.00755 mmol, 53.9% yield).

$C_{120}H_{152}O_{22}$: 1946.51 g/mol.

^{1}H-NMR (400 MHz, [D$_6$]-dimethyl sulfoxide, 298 K) δ [in ppm] = 8.03 (s, 4H, H-4), 8.01 (d, *J* = 8.1 Hz, 4H, H-6), 7.42 (dd, *J* = 1.2 Hz, *J* = 6.8 Hz, 4H, H-7), 7.39 (s, 8H, H-12), 7.29 (dd, *J* = 1.2 Hz, *J* = 7.0 Hz, 4H, H-8), 7.08 (d, *J* = 8.6 Hz, 4H, H-9), 4.33 (d, *J* = 5.3 Hz, 4H, H-23$_{1/2}$), 4.24 (d, *J* = 5.3 Hz, 4H, H-23$_{1/2}$), 3.85 (t, *J* = 3.6 Hz, 8H, H-17), 3.74 (t, *J* = 3.6 Hz, 8H, H-18), 3.62-3.57 (m, 32H, H-19, 20, 21, 22), 3.38 (hept., *J* = 6.8 Hz, 8H, H-14), 2.28 (s, 12 H, H-24), 1.19 (d, *J* = 6.8 Hz, 24H, H-15$_{1/2}$), 1.16 (d, *J* = 6.8 Hz, 24H, H-15$_{1/2}$).

^{13}C-NMR (101 MHz, [D$_6$]-dimethyl sulfoxide, 298 K) δ [in ppm] = 152.12 (C-16), 150.57 (C-2), 141.21 (C-13), 135.23 (C-3), 134.39 (C-11), 132.69 (C-10), 130.44 (C-5), 129.87 (C-4), 128.10 (C-1), 126.32 (C-6), 125.75 (C-8), 125.45 (C-9), 125.08 (C-7), 124.94 (C-12). 97.57 (C-23), 73.96 (C-17), 70.19, 70.00, 69.98, 69.92 (C-19, 20, 21, 22), 69.73 (C-18), 55.23 (C-24), 25.69 (C-14), 23.90 (C-15$_{1/2}$), 23.84 (C-15$_{1/2}$).

^{1}H,^{1}H-COSY (400 MHz / 400 MHz, [D$_6$]-dimethyl sulfoxide, 298 K) δ [in ppm] = 8.01/7.42 (H-6/H-7), 7.42/8.01, 7.29 (H-7/H-6, 8), 7.29/7.42, 7.08 (H-8/H-7, 9), 7.08/7.29 (H-9/H-8), 4.33/4.24 (H-23$_{1/2}$/H-23$_{1/2}$), 4.24/4.33 (H-23$_{1/2}$/H-23$_{1/2}$), 3.85/3.74 (H-18/H-17), 3.74/3.85 (H-17/H-18), 3.38/1.19, 1.16 (H-14/H-15$_{1/2}$), 1,19/3.38 (H-15$_{1/2}$/H-14), 1,16/3.38 (H-15$_{1/2}$/H-14).

^{1}H,^{13}C-GHSQC (400 MHz / 101 MHz, [D$_6$]-dimethyl sulfoxide, 298 K) δ (^{1}H) / δ (^{13}C) [in ppm] = 8.03/129.87 (H-4/C-4), 8.01/126.32 (H-6/C-6), 7.42/125.08 (H-7/C-7), 7.39/124.94 (H-12/C-12), 7.29/125.75 (H-8/C-8), 7.08/125.45 (H-9/C-9), 4.33/97.57 (H-23$_{1/2}$/C-23), 4.24/97.57 (H-23$_{1/2}$/C-23), 3.85/73.96 (H-17/C-17), 3.74/69.73 (H-18/C-18), 3.62-3.57/70.19, 70.00, 69.98, 69.92 (H-19, 20, 21, 22/C-19, 20, 21, 22), 3.38/25.69 (H-14/C-14), 2.28/55.23 (H-24/C-24), 1.19/23.90, 23.84 (H-15$_{1/2}$/C-15$_{1/2}$), 1.16/23.90, 23.84 (H-15$_{1/2}$/C-15$_{1/2}$).

^{1}H,^{13}C-GHMBC (400 MHz / 101 MHz, [D$_6$]-dimethyl sulfoxide, 298 K) δ (^{1}H) / δ (^{13}C) [in ppm] = 8.03/150.57, 134.39, 132.69, 126.32 (H-4/C-2, 11, 10, 6), 8.01/132.69, 129.87, 125.75 (H-6/C-10, 4, 8), 7.42/130.44, 125.45 (H-7/C-5, 9), 7.39/152.12, 135.23, 25.69 (H-12/C-16, 3, 14), 7.29/132.69, 126.32 (H-8/C-10, 6), 7.08/130.44, 128.10, 125.08 (H-9/C-5, 1, 7), 4.33/150.57, 55.23 (H-23$_{1/2}$/C-2, 24), 4.24/150.57, 55.23 (H-23$_{1/2}$/C-2, 24), 3.38/152.12, 141.21, 124.94, 23.90, 23.84 (H-14/C-16, 13, 12, 15$_{1/2}$), 2.28/97.57 (H-24/C-23), 1.19/141.21, 25.69 (H-15$_{1/2}$/C-13, 14), 1.16/141.21, 25.69 (H-15$_{1/2}$/C-13, 14).

MS (ESI-pos, MeOH): *m/z* = 996.0306 ([M+2Na]$^{2+}$, calcd. 996.0297 [$C_{120}H_{152}O_{22}Na_2$]$^{2+}$), *m/z* = 991.0750 ([M+2NH$_4$]$^{2+}$, calcd. 991.0743 [$C_{120}H_{160}O_{22}N_2$]$^{2+}$).

IR (ATR-FT): $\tilde{\nu}$ (cm^{-1}) = 703, 712, 733, 738, 749, 760, 764, 888, 1107, 1263, 1270, 1275, 1457, 2871, 2997, 3005, 3038, 3054.

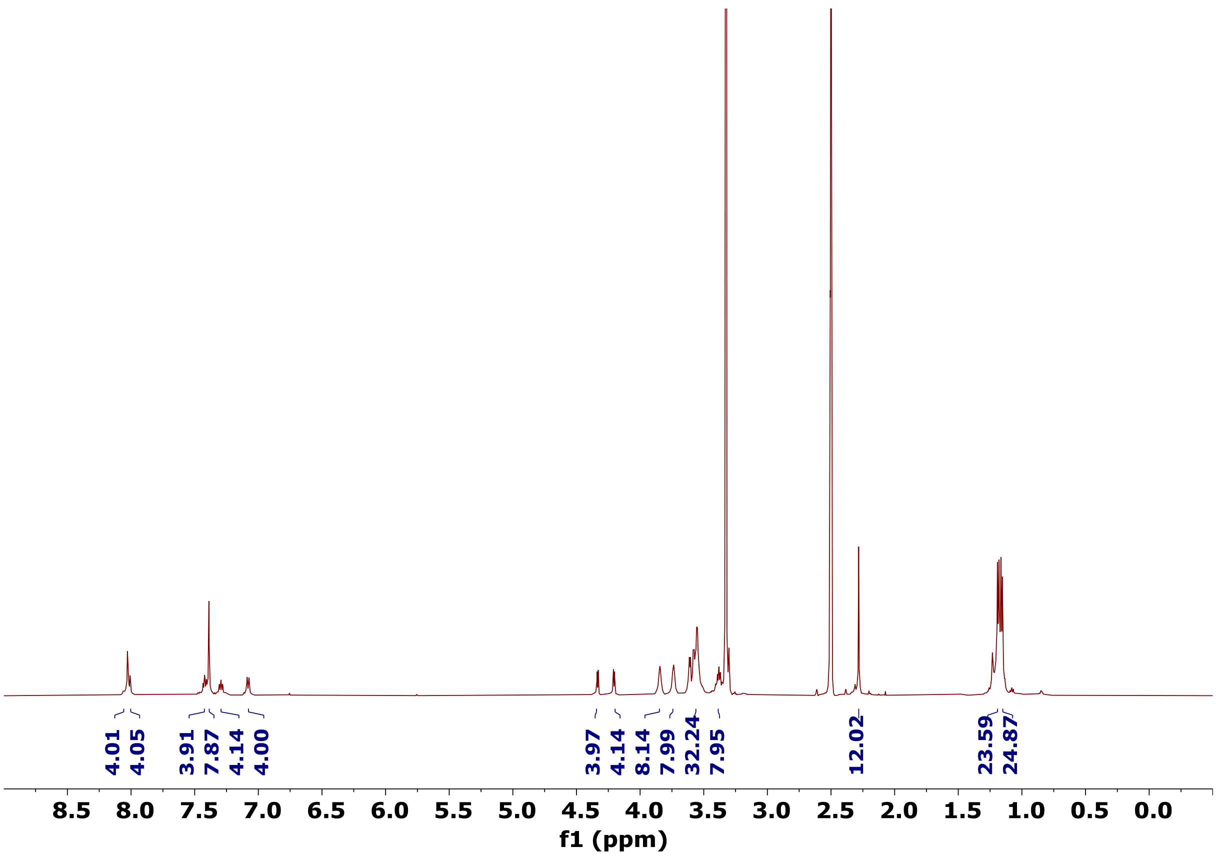

Figure S55: ^{1}H NMR spectrum of (*S,S*)-**iPr-M2$_6$** (DMSO-d_6, 298 K, 400 MHz).

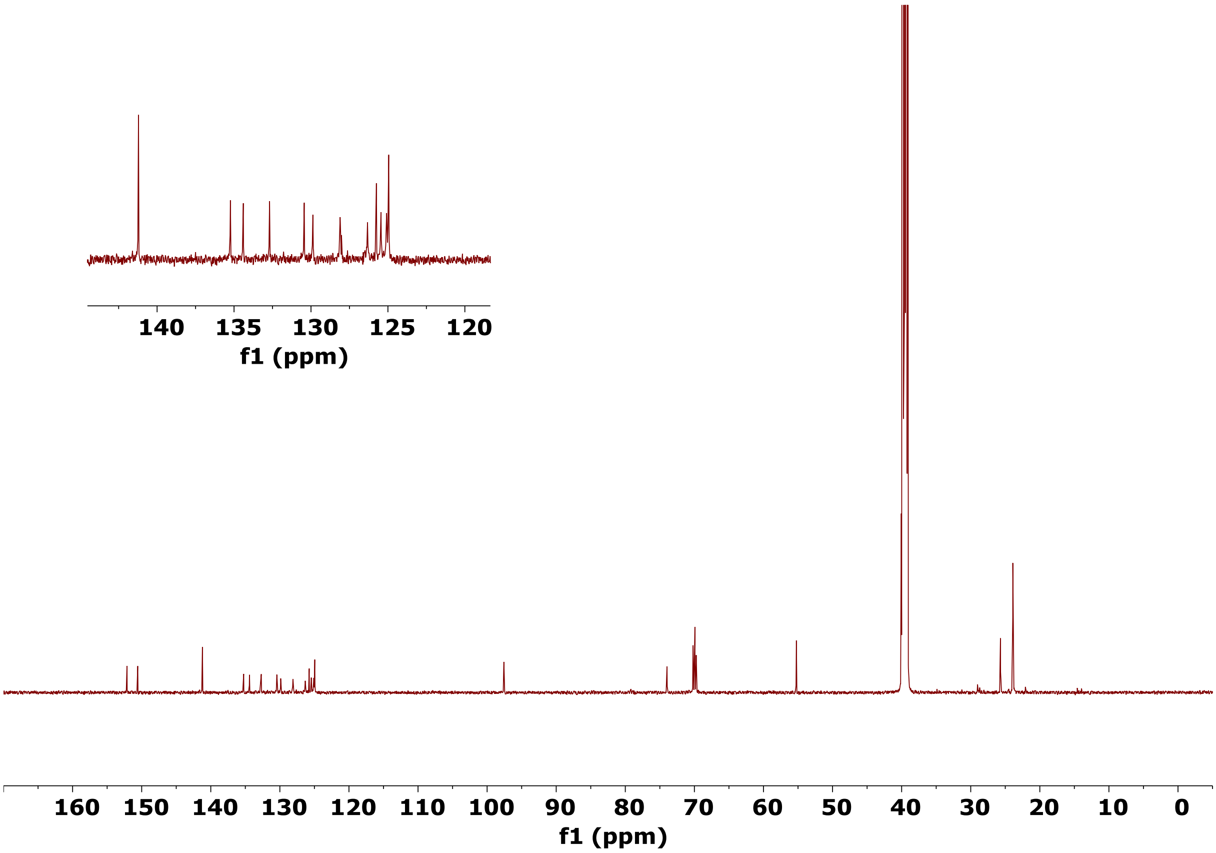

Figure S56: ^{13}C NMR spectrum of (*S,S*)-**iPr-M2$_6$** (DMSO-d_6, 298 K, 400 MHz).

Compound **HiPr-M2$_6$**

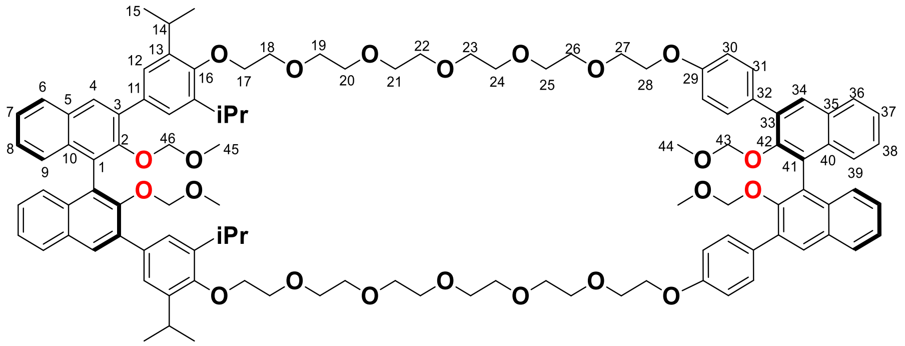

As described above (General procedure **C**), compound **HiPr-M2$_6$** was synthesized using compound **H-2** (10.0 mg, 0.0179 mmol, 1.0 equiv), cesium carbonate (18.0 mg, 0.0552 mmol, 3.2 equiv) and **iPr-3$_6$** (27.9 mg, 0.0178 mmol, 1.0 equiv) dissolved in degassed acetonitrile (1 mL). After purification by column chromatography (methanol/dichloromethane 1:50) the product was obtained as a colorless wax (17.9 mg, 0.0101 mmol, 55.6 %yield).

$C_{108}H_{128}O_{22}$: 1778.19 g/mol.

^{1}H-NMR (400 MHz, [D$_6$]-dimethyl sulfoxide, 298 K) δ [in ppm] = 8.05 (s, 2H, H-4), 8.03 (d, *J* = 7.8 Hz, 2H, H-6), 8.01 (s, 2H, H-34), 7.99 (d, *J* = 8.4 Hz, 2H, H-36), 7.61 (d, *J* = 8.8 Hz, 4H, H-30), 7.46-7.41 (m, 4H, H-7, 37), 7.40 (s, 4H, H-12), 7.33-7.26 (m, 4H, H-8, 38), 7.10-7.06 (m, 4H, H-9, 39), 7.04 (d, *J* = 8.8 Hz, 4H, H-31), 4.36 (d, *J* = 5.4 Hz, 2H, H-46$_{1/2}$), 4.30 (d, *J* = 5.4 Hz, 2H, H-46$_{1/2}$), 4.22 (d, *J* = 5.2 Hz, 2H, H-43$_{1/2}$), 4.20 (d, *J* = 5.3 Hz, 2H, H-43$_{1/2}$), 4.12 (t, *J* = 3.9 Hz, 4H, H-17), 3.85 (t, *J* = 3.6 Hz, 4H, H-28), 3.76-3.73 (m, 8H, H-18, 27), 3.62-3.51 (m, 32H, H-19, 20, 21, 22, 23, 24, 25, 26), 3.39 (hept., *J* = 6.8 Hz, 4H, H-14), 2.31 (s, 6H, H-45), 2.23 (s, 6H, H-44), 1.19 (d, *J* = 6.8 Hz, 12H, H-15$_{1/2}$), 1.16 (d, *J* = 6.8 Hz, 12H, H-15$_{1/2}$).

^{13}C-NMR (101 MHz, [D$_6$]-dimethyl sulfoxide, 298 K) δ [in ppm] = 157.93 (C-16), 152.13 (C-29), 150.59 (C-2), 150.58 (C-42), 141.23 (C-13), 135.27 (C-3), 134.49 (C-11), 134.40 (C-32),132.69 (C-10), 132.65 (C-40), 130.59 (C-5), 130.51 (C-33), 130.45 (C-35), 130.33 (C-30), 129.91 (C-4), 129.87 (C-34), 128.15 (C-6), 127.97 (C-36), 126.34 (C-8 or 38), 126.24 (C-8 or 38), 125.85 (C-9 or 39), 125.76 (C-9 or 39), 125.61 (C-1, 41), 125.46 (C-12), 125.10 (C-7 or 37), 124.98 (C-7 or 37), 114.40 (C-31), 97.61 (C-46), 97.47 (C-43), 73.96 (C-28), 70.16, 69.99, 69.98, 69.95, 69.87, 69.86, 69.84, 69.83 (C-19, 20, 21, 22, 23, 24, 25, 26), 69.73 (C-18 or C-27), 68.95 (C-18 or 27), 67.18 (C-17), 55.29 (C-45), 55.15 (C-44), 25.71 (C-14), 23.94 (C-15$_{1/2}$), 23.88 (C-15$_{1/2}$).

^{1}H,^{1}H-COSY (400 MHz / 400 MHz, [D$_6$]-dimethyl sulfoxide, 298 K) δ [in ppm] = 8.03/7.46-7.41 (H-6/H-7), 7.99/7.46-7.41 (H-36/H-37), 7.61/7.04 (H-30/H-31), 7.46-7.41/8.03, 7.99, 7.33-7.26 (H-7, 37/H-6, 36, 8, 38), 7.10-7.06/7.33-7.26 (H-9, 39/H-8, 38), 7.04/7.61 (H-31/H-30), 4.36/4.30 (H-46$_{1/2}$/ H-46$_{1/2}$), 4.30/4.36 (H-46$_{1/2}$/ H-46$_{1/2}$), 4.22/4.20 (H-43$_{1/2}$/H-43$_{1/2}$), 4.20/4.22 (H-43$_{1/2}$/H-43$_{1/2}$), 4.12/3.76-3.73 (H-17/H-18), 3.85/3.76-3.73 (H-28/H-27), 3.76-3.73/4.12, 3.85 (H-18, 27/H-17, 28), 3.39/1,21, 1.19 (H-14/H-15).

$^{1}H,^{13}C$-GHSQC (400 MHz / 101 MHz, [D$_6$]-dimethyl sulfoxide, 298 K) δ (^{1}H) / δ (^{13}C) [in ppm] = 8.05/129.91 (H-4/C-4), 8.03/128.15 (H-6/C-6), 8.01/129.87 (H-34/C-34), 7.99/127.97 (H-36/C-36), 7.61/130.33 (H-30/C-30), 7.46-7.41/125.10, 124.98 (H-7, 37/C-7, 37), 7.40/125.46 (H-12/C-12), 7.33-7.26/126.34, 126.24 (H-8, 38/C-8, 38), 7.10-7.06/125.85, 125.76 (H-9, 39/C-9, 39), 7.04/114.40 (H-31/C-31), 4.36/97.61 (H-$46_{1/2}$/C-46), 4.30/97.61 (H-$46_{1/2}$/C-46), 4.22/97.47 (H-$43_{1/2}$/C-43), 4.20/97.47 (H-$43_{1/2}$/C-43), 4.12/67.18 (H-17/C-17), 3.85/73.96 (H-28/C-28), 3.76-3.73/69.73, 68.95 (H-18, 27/C-18, 27), 3.62-3.51/70.16, 69.99, 69.98, 69.95, 69.87, 69.86, 69.84,69.83 (H-19, 20, 21, 22, 23, 24, 25, 26/ C-19, 20, 21, 22, 23, 24, 25, 26), 3.39/25.71 (H-14/C-14), 2.31/55.29 (H-45/C-45), 2.23/55.15 (H-44/C-44), 1.19/23.94 (H-$15_{1/2}$/C-$15_{1/2}$), 1.16/23.88 (H-$15_{1/2}$/C-$15_{1/2}$).

$^{1}H,^{13}C$-GHMBC (400 MHz / 101 MHz, [D$_6$]-dimethyl sulfoxide, 298 K) δ (^{1}H) / δ (^{13}C) [in ppm] = 8.05/150.59, 134.49, 132.69, 128.15 (H-4/C-2, 11, 10, 6), 8.03/132.69, 129.91, 126.34 (H-6/C-10, 4, 8), 8.01/150.58, 132.65, 134.40, 127.97 (H-34/C-42, 40, 32, 36), 7.99/132.65, 129.87, 126.34 (C-40, 34, 38), 7.61/134.40 (H-30/C-32), 7.46-7.41/130.59, 130.45, 125.85, 125.76 (H-7, 37/C-5, 35, 9, 39), 7.40/157.93, 135.27 (H-12/C-16, C-3), 7.33-7.26/132.69, 132.65, 128.15, 127.97 (H-8, 38/C-10, 40, 6, 36), 7.10-7.06/130.59, 130.45, 125.61, 125.10, 124.98 (H-9, 39/C-5, 35, 7, 37, 1, 41), 7.04/152.13, 130.51 (H-31/C-29, 33), 4.36/55.29 (H-$46_{1/2}$/C-45), 4.30/55.29 (H-$46_{1/2}$/C-45), 4.22/55.15 (H-$43_{1/2}$/C-45), 4.20/55.15 (H-$43_{1/2}$/C-45), 3.39/157.93, 125.46, 23.94, 23.88 (H-14/C-16, 12, $15_{1/2}$), 2.31/97.61 (H-45/C-46), 2.23/97.47 (H-44/C-43), 1.19/141.23, 25.71 (H-$15_{1/2}$/C-13, 14), 1.16/141.23, 25.71 (H-$15_{1/2}$/C-13, 14).

MS (ESI-pos, MeOH**)**: *m/z* = 1800.8847 ([M+Na]$^{+}$, calcd. 1800.8823 [$C_{108}H_{128}O_{22}Na$]$^{+}$).

IR (ATR-FT): $\tilde{\nu}$ (cm^{-1}) = 703, 749, 764, 897, 1262, 1275, 1457, 2869, 2989, 3005, 3053.

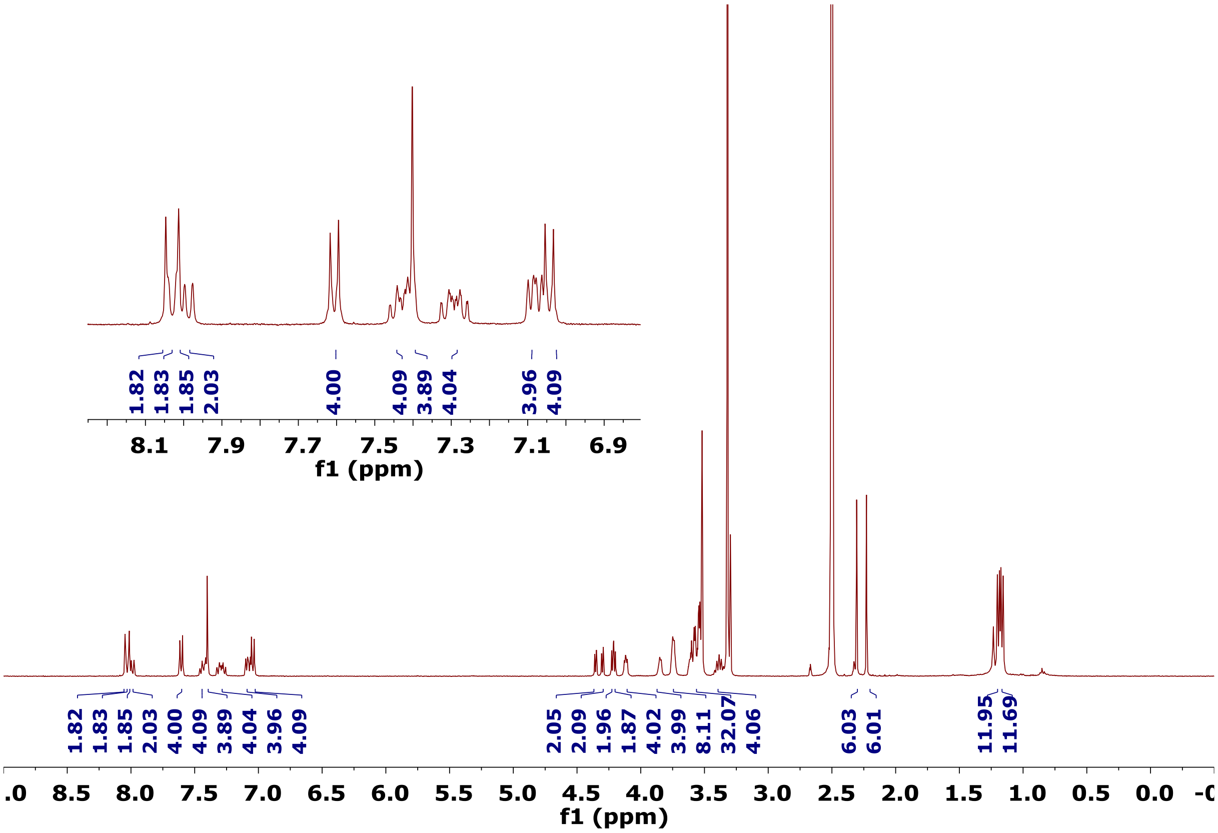

Figure S57: ^{1}H NMR spectrum of (*S,S*)- **HiPr-M2$_6$** (DMSO-*d$_6$*, 298 K, 400 MHz).

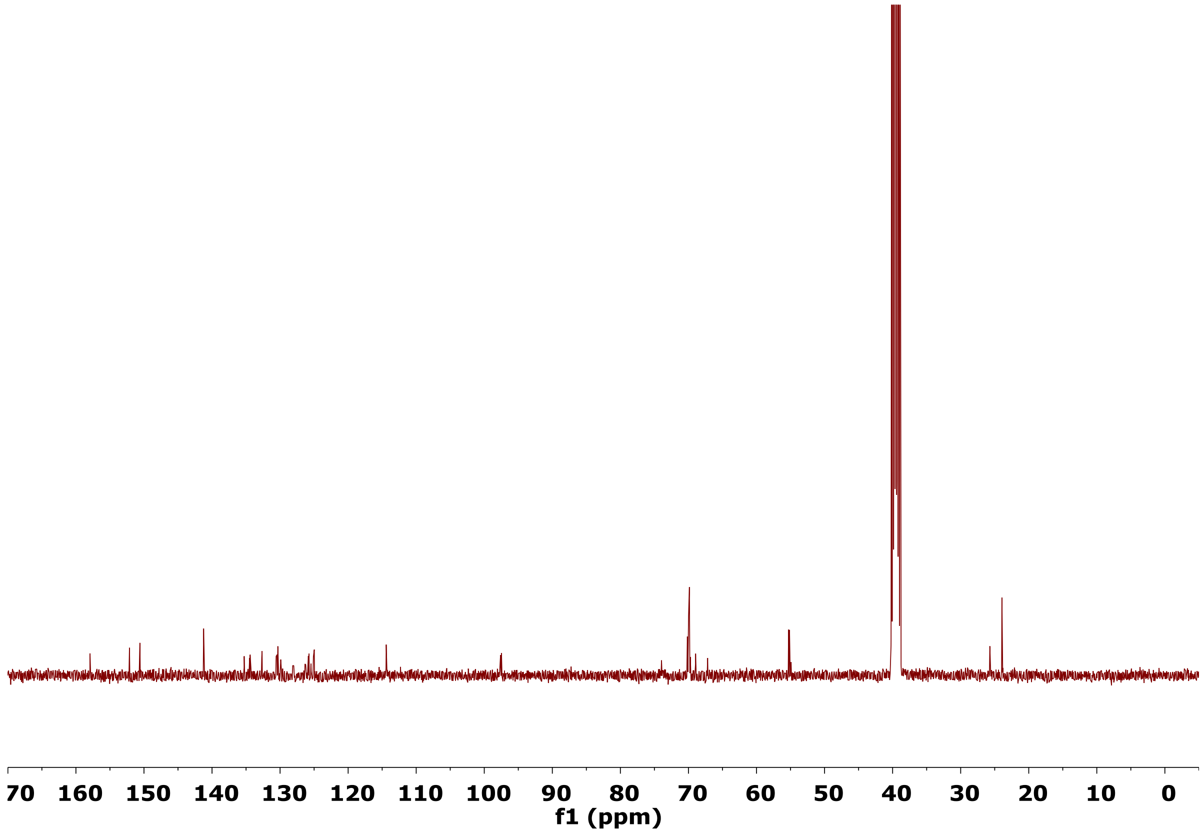

Figure S58: ^{13}C NMR spectrum of (*S,S*)- **HiPr-M2$_6$** (DMSO-*d$_6$*, 298 K, 400 MHz).

Compound **Me-M2$_2$**

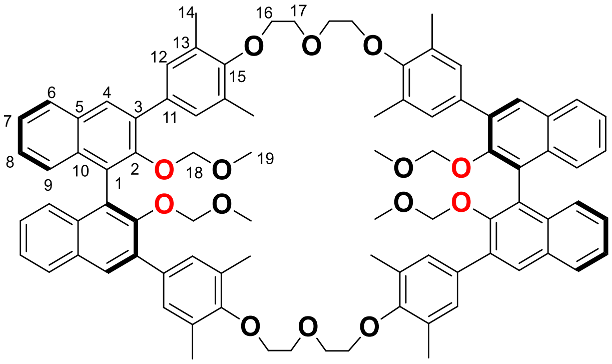

As described before (General procedure **D**), compound **Me-M2$_2$** was synthesized using compound **Me-2** (20.0 mg, 0.0325 mmol, 1.0 equiv), cesium carbonate (31.0 mg, 0.0951 mmol, 3.2 equiv) and **Me-6$_2$** (26.9 mg, 0.0325 mmol, 1.0 equiv) dissolved in degassed acetonitrile (3 mL). After purification by column chromatography (cyclohexane/ethyl acetate 6:1) the product was obtained as colorless wax (25.9 mg, 0.0189 mmol, 59.4% yield).

$C_{88}H_{88}O_{14}$: 1369.66 g/mol.

^{1}H-NMR (400 MHz, [D$_1$]-chloroform, 298 K) δ [in ppm] = 7.92 (s, 4H, H-4), 7.86 (d, *J* = 8.4 Hz, 4H, H-6), 7.48 (s, 8H, H-12), 7.40-7.36 (m, 4H, H-7), 7.23-7.21 (m, 8H, H-8, 9), 4.37 (d, *J* = 6.2 Hz, 4H, H-18$_{1/2}$), 4.31 (d, *J* = 6.2 Hz, 4H, H-18$_{1/2}$), 4.15-4.06 (m, 4H, H-16), 4.05-4.01 (m, 8H, H-17), 3.93-3.90 (m, 4H, H-16$^{'}$), 2.46 (s, 24H, H-14), 2.28 (s, 12H, H-19).

^{13}C-NMR (101 MHz, [D$_1$]-chloroform, 298 K) δ [in ppm] = 154.73 (C-15), 151.72 (C-2), 135.22 (C-3), 134.76 (C-11), 133.52 (C-10), 131.05 (C-13), 130.83 (C-5), 130.24 (C-4), 130.08 (C-12), 127.75 (C-6), 126.48 (C-1), 126.43 (C-8, 9), 126.02 (C-8, 9), 124.96 (C-7), 98.56 (C-18), 71.37, 70.97 (C-16, 17), 55.79 (C-19), 16.21 (C-14).

^{1}H,^{1}H-COSY (400 MHz / 400 MHz, [D$_1$]-chloroform, 298 K) δ [in ppm] = 7.86/7.40-7.36 (H-6/H-7), 7.48/2.46 (H-12/H-14), 7.40-7.36/7.86, 7.23-7.21 (H-7/H-6, 8, 9), 7.23-7.21/7.40-7.36 (H-8, 9/H-7), 4.37/4.31 (H-18$_{1/2}$/H-18$_{1/2}$), 4.31/4.37 (H-18$_{1/2}$/ H-18$_{1/2}$), 2.46/7.48 (H-14/H-12).

^{1}H,^{13}C-GHSQC (400 MHz / 101 MHz, [D$_1$]-chloroform, 298 K) δ (^{1}H) / δ (^{13}C) [in ppm] = 7.92/130.24 (H-4/C-4), 7.86/127.75 (H-6/C-6), 7.48/130.08 (H-12/C-12), 7.40-7.36/124.96 (H-7/C-7), 7.23-7.21/126.43, 126.02 (H-8, 9/C-8, 9), 4.37/98.56 (H-18$_{1/2}$/C-18), 4.31/98.56 (H-18$_{1/2}$/C-18), 4.15-3.90/71.37, 70.97 (H-16, 17/C-16, 17), 2.46/16.21 (H-14/C-14), 2.28/55.79 (H-19/C-19).

^{1}H,^{13}C-GHMBC (400 MHz / 101 MHz, [D$_1$]-chloroform, 298 K) δ (^{1}H) / δ (^{13}C) [in ppm] = 7.92/151.72, 134.76, 133.52, 127.75 (H-4/C-2, 11, 10, 6), 7.86/133.52, 130.24, 126.43, 126.02 (H-6/C-10, 4, 8, 9), 7.48/154.73, 135.22, 131.05, 16.21 (H-12/C-16, 3, 13, 14), 7.40-7.36/130.83, 126.43, 126.02(H-7/C-5, 8, 9), 7.23-7.21/130.83, 127.75, 126.48 (H-8, 9/C-5, 6, 1), 4.37/151.72, 55.79 (H-18$_{1/2}$/C-2, 19), 4.31/151.72, 55.79 (H-18$_{1/2}$/C-2, 19), 2.46/154.73, 131.05, 130.08 (H-14/C-15, 13, 12),2.28/98.56 (H-19/C-18).

MS (ESI-pos, MeOH): *m/z* = 1391.6074 ([M+Na]$^+$, calcd. 1391.6066 [$C_{88}H_{88}O_{14}Na$]$^+$).

IR (ATR-FT): $\tilde{\nu}$ (cm^{-1}) = 703, 749, 764, 896, 1199, 1263, 1274, 1559, 2989, 3006, 3054.

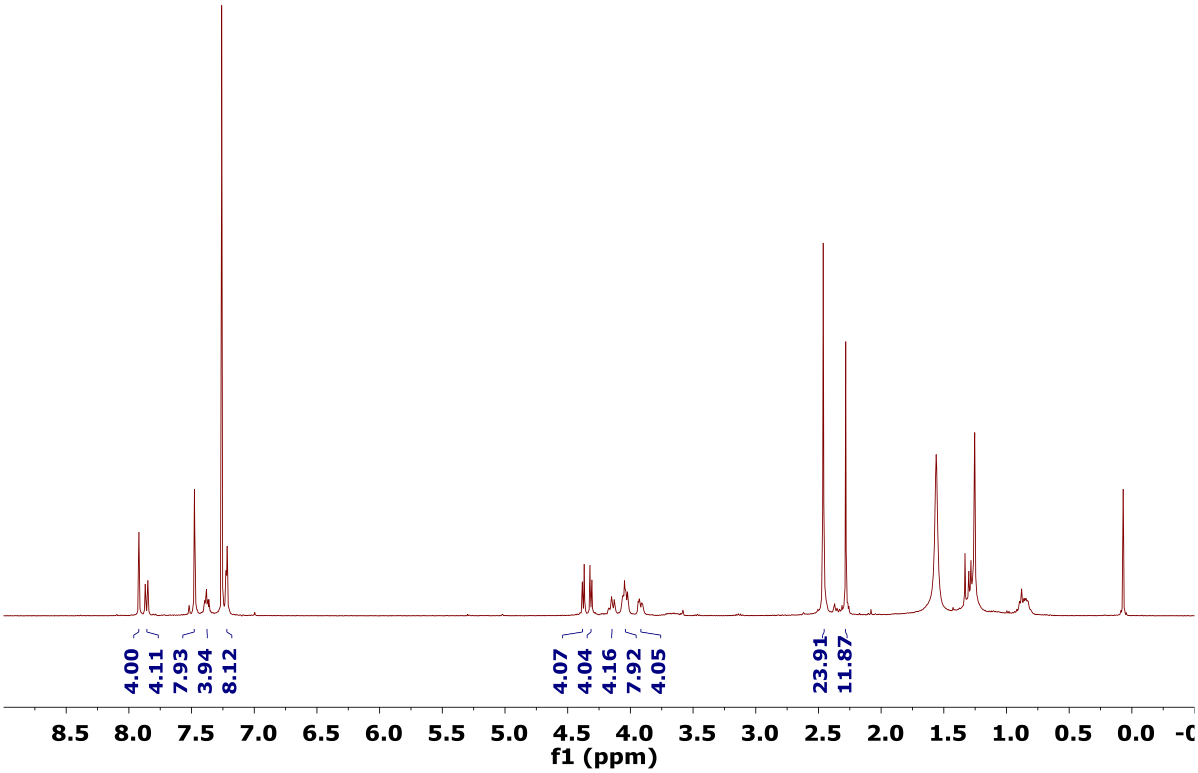

Figure S59: ^{1}H NMR spectrum of (*S,S*)-**Me-M2$_2$** ($CDCl_3$, 298 K, 400 MHz).

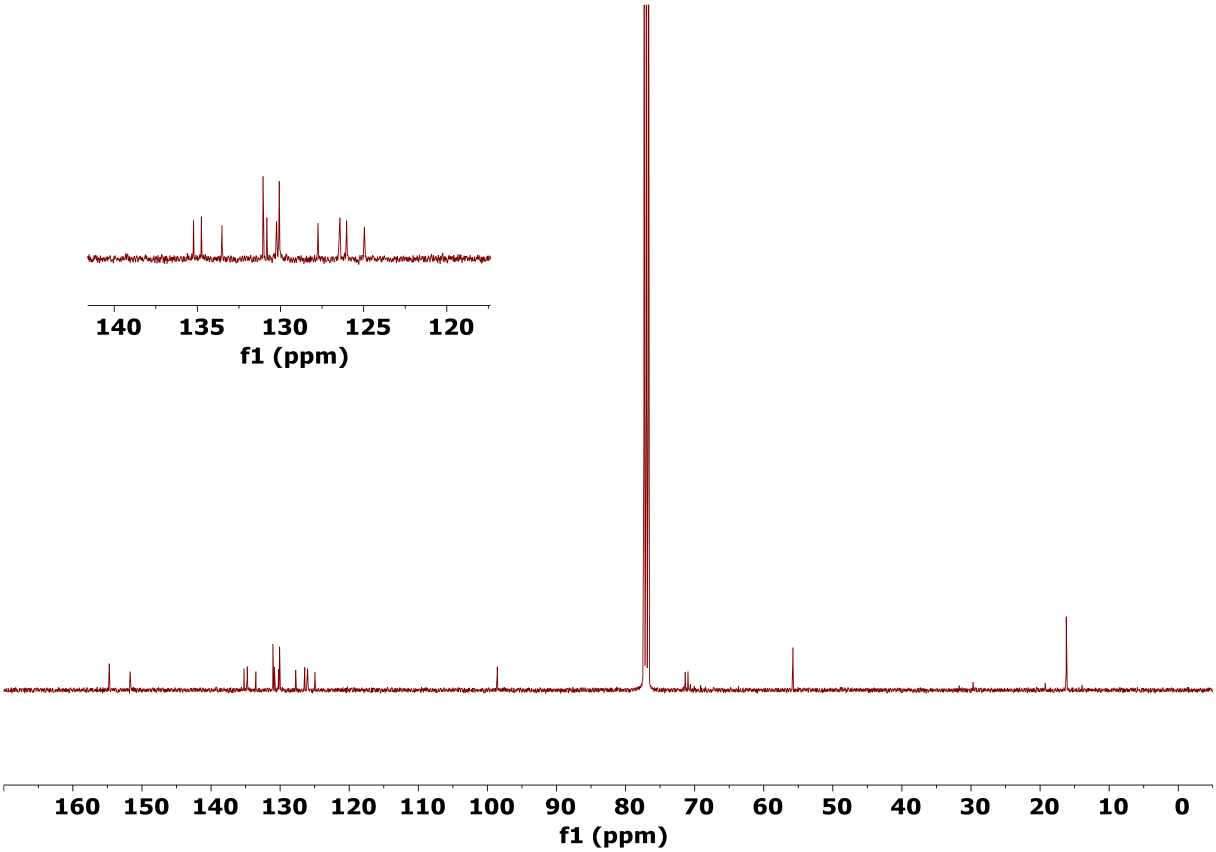

Figure S60: ^{13}C NMR spectrum of (*S,S*)-**Me-M2$_2$** ($CDCl_3$, 298 K, 400 MHz).

Compound **H-M2$_2$**

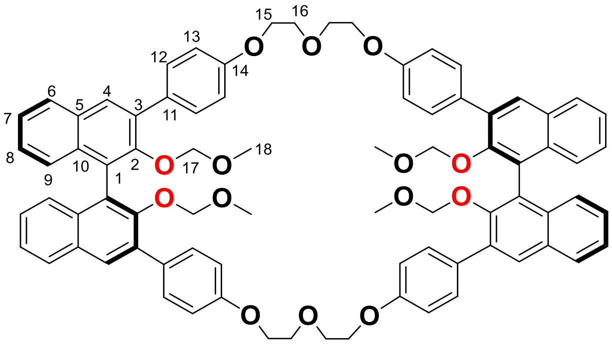

As described before (General procedure **D**), compound **H-M2$_2$** was synthesized using compound **H-2** (10.1 mg, 0.0181 mmol, 1.0 equiv), cesium carbonate (18.3 mg, 0.0561 mmol, 3.2 equiv) and **H-6$_2$** (12.9 mg, 0.0167 mmol, 1.0 equiv) dissolved in degassed acetonitrile (2 mL). After purification by column chromatography (cyclohexane/ethyl acetate 3:1) the product was obtained as colorless wax (17.7 mg, 0.0141 mmol, 77.8% yield).

$C_{80}H_{72}O_{14}$: 1257.44 g/mol.

^{1}H-NMR (400 MHz, [D$_1$]-chloroform, 298 K) δ [in ppm] = 7.89 (s, 4H, H-4), 7.84 (d, *J* = 8.2 Hz, 4H, H-6), 7.69 (d, *J* = 8.6 Hz, 8H, H-12), 7.40-7.36 (m, 4H, H-7), 7.25-7.23 (m, 8H, H-8, 9), 7.03 (d, *J* = 8.6 Hz, 8H, H-13), 4.37 (d, *J* = 5.9 Hz, 4H, H-17$_{1/2}$), 4.32 (d, *J* = 5.9 Hz, 4H, H-17$_{1/2}$), 4.25-4.23 (m, 8H, H-15), 3.99-3.97 (m, 8H, H-16), 2.29 (s, 12H, H-18).

^{13}C-NMR (101 MHz, [D$_1$]-chloroform, 298 K) δ [in ppm] = 158.24 (C-14), 151.49 (C-2), 134.97 (C-3), 133.40 (C-10), 131.63 (C-11), 130.86 (C-5), 130.68 (C-12), 130.02 (C-4), 127.72 (C-6), 126.53 (C-9), 126.43 (C-1), 126.02 (C-8), 125.01 (C-7), 114.65 (H-13), 98.37 (C-17), 69.87 (C-16), 67.62 (C-15), 55.81 (C-18).

^{1}H,^{1}H-COSY (400 MHz / 400 MHz, [D$_1$]-chloroform, 298 K) δ [in ppm] = 7.84/7.40-7.36 (H-6/H-7), 7.69/7.03 (H-12/H-13), 7.40-7.36/7.84, 7.25-7.23 (H-7/H-6, 8, 9), 7.25-7.23/7.42-7.36 (H-8, 9/H-7), 7.03/7.69 (H-13/H-12), 4.37/4.32 (H-17$_{1/2}$/H-17$_{1/2}$), 4.32/4.37 (H-17$_{1/2}$/H-17$_{1/2}$), 4.25-4.23/3.99-3.97 (H-15/H-16), 3.99-3.97/4.25-4.23 (H-16/H-15).

^{1}H,^{13}C-GHSQC (400 MHz / 101 MHz, [D1]-chloroform, 298 K) δ (^{1}H) / δ (^{13}C) [in ppm] = 7.89/130.02 (H-4/C-4), 7.84/127.72 (H-6/C-6), 7.69/130.68 (H-12/C-12), 7.40-7.37/125.01 (H-7/C-7), 7.25-7.23/126.53, 126.02 (H-8, 9/C-8, 9), 7.03/114.65 (H-13/C-13), 4.37/98.37 (H-17$_{1/2}$/C-17), 4.32/98.37 (H-17$_{1/2}$/C-17), 4.25-4.23/67.62 (H-15/C-15), 3.99-3.97/69.87 (H-16/C-16), 2.29/55.81 (H-18/C-18).

^{1}H,^{13}C-GHMBC (400 MHz / 101 MHz, [D1]-chloroform, 298 K) δ (^{1}H) / δ (^{13}C) [in ppm] = 7.89/151.49, 133.40, 131.63, 127.72 (H-4/C-2, 10, 11, 6), 7.84/133.40, 130.02, 126.02 (H-6/C-10, 4, 8), 7.69/158.24, 134.97 (H-12/C-14, 3), 7.40-7.37/130.86, 126.53 (H-7/C-5, 9), 7.25-7.23/133.40, 127.72, 126.42, 125.01 (H-8, 9/C-10, 6, 1, 7), 7.03/131.63 (H-13/C-11),.4.37/151.49, 55.81 (H-17$_{1/2}$/C-2, 18), 4.32/151.49, 55.81 (H-17$_{1/2}$/C-2, 18), 2.29/98.37 (H-18/C-17).

MS (ESI-pos, MeOH): *m/z* = 1279.4806 ([M+Na]$^+$, calcd. 1279.4814 [$C_{80}H_{72}O_{14}Na$]$^+$).

IR (ATR-FT): $\tilde{\nu}$ (cm^{-1}) = 702, 734, 749, 764, 897, 1079, 1097, 1177, 1263, 1275, 1361, 1512, 1606, 2827, 2998, 3004, 3054.

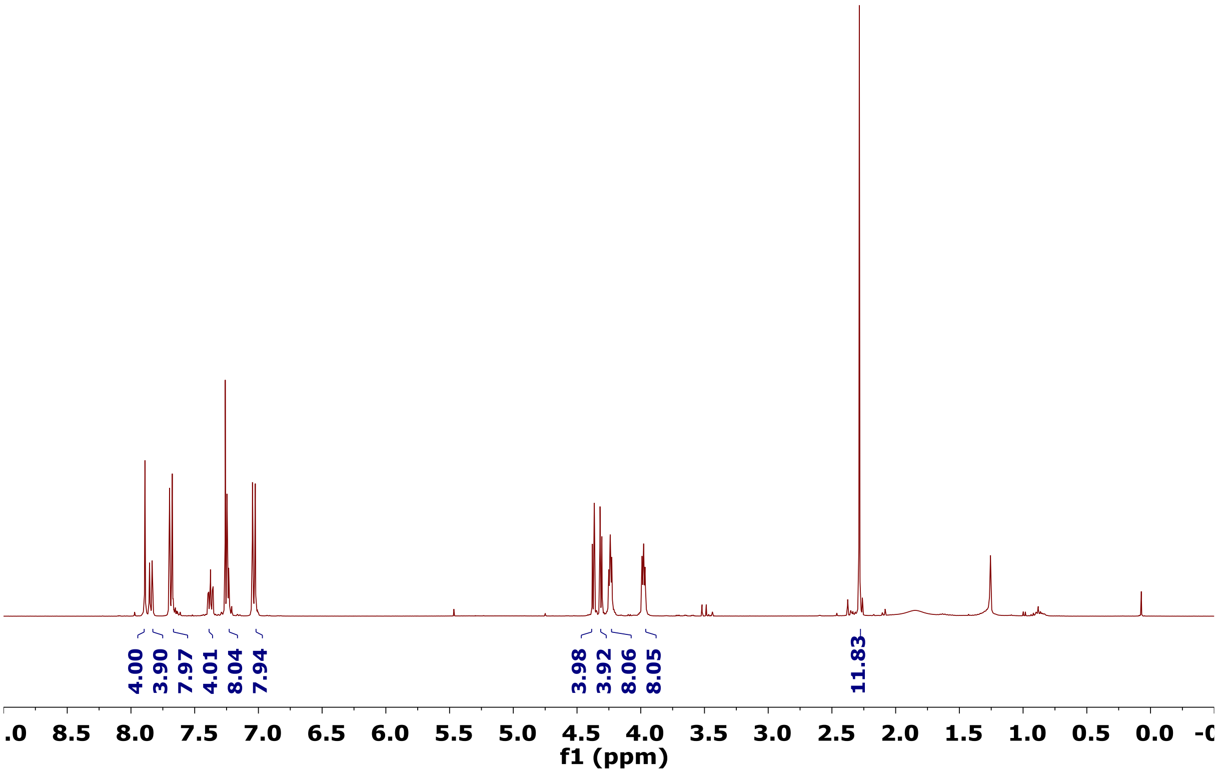

Figure S61: ^{1}H NMR spectrum of (*S*,*S*)-**H-M2$_2$** ($CDCl_3$, 298 K, 400 MHz).

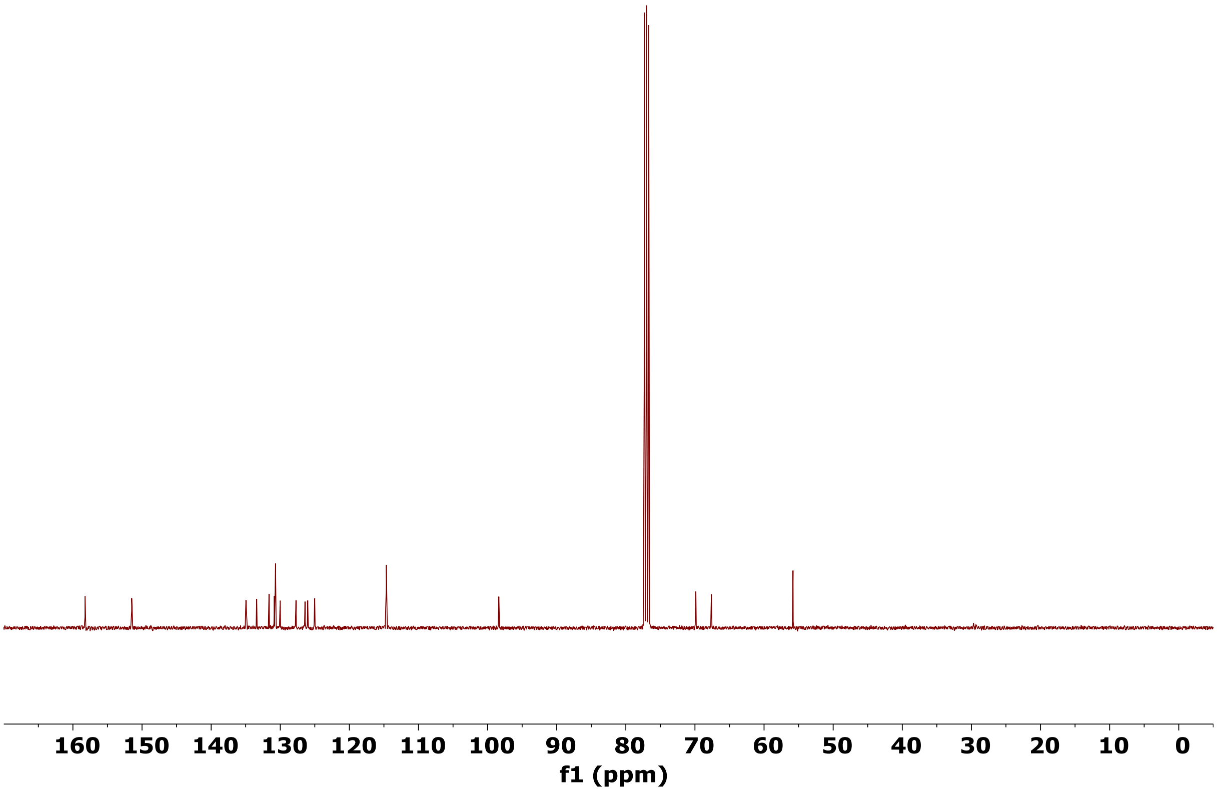

Figure S62: ^{1}H NMR spectrum of (*S*,*S*)-**H-M2$_2$** ($CDCl_3$, 298 K, 400 MHz).

Compound **iPr-M2₂**

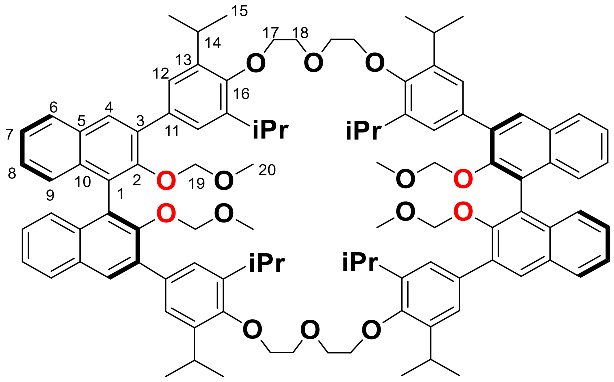

As described above (General procedure **D**), compound **iPr-M2$_2$** was synthesized using compound **iPr-2** (12.2 mg, 0.0168 mmol, 1.0 equiv), cesium carbonate (20.9 mg, 0.0641 mmol, 3.2 equiv) and **iPr-6$_2$** (15.1 mg, 0.0161 mmol, 1.0 equiv) dissolved in degassed acetonitrile (2 mL). After purification by column chromatography (cyclohexane/ethyl acetate 3:1) the product was obtained as a white solid (20.6 mg, 0.0129 mmol, 76.5% yield).

$C_{104}H_{120}O_{14}$: 1594.09 g/mol.

^{1}H-NMR (400 MHz, [D$_1$]-chloroform, 298 K) δ [in ppm] = 7.97 (s, 4H, H-4), 7.90 (d, *J* = 8.8 Hz, 4H, H-6), 7.59 (s, 8H, H-12), 7.43-7.39 (m, 8H, H-9, 7), 7.29-7.25 (m, 4H, H-8), 4.28 (d, *J* = 6.3 Hz, 4H, H-19$_{1/2}$), 4.26 (d, *J* = 6.3 Hz, 4H, H-19$_{1/2}$), 4.10-4.06 (m, 8H, H-17), 4.03-4.01 (m, 8H, H-18), 3.57 (hept., *J* = 6.8 Hz 8H, H-14), 2.26 (s, 12H, H-20), 1.42 (d, *J* = 6.9 Hz, 24H, H-15$_{1/2}$), 1.32 (d, *J* = 6.9 Hz, 24H, H-15$_{1/2}$).

^{13}C-NMR (101 MHz, [D$_1$]-chloroform, 298 K) δ [in ppm] = 152.14 (C-16), 151.99 (C-2), 141.72 (C-13), 135.65 (C-3), 135.21 (C-11), 133.00 (C-10), 130.94 (C-5), 129.54 (C-4), 127.97 (C-6), 126.40 (C-9), 126.29 (C-1), 126.12 (C-8), 125.51 (C-12), 124.90 (C-7), 98.53 (C-19), 74.18 (C-17), 71.26 (C-18), 55.58 (C-20), 26.27 (C-14), 24.31 (C-15$_{1/2}$), 24.23 (C-15$_{1/2}$).

^{1}H,^{1}H-COSY (400 MHz / 400 MHz, [D$_1$]-chloroform, 298 K) δ [in ppm] = 7.90/7.43-7.39 (H-6/H-7), 7.43-7.39/7.90, 7.29-7.25 (H-7, 9/H-6, 8), 3.57/1.42, 1.32 (H-14/H-15$_{1/2}$), 1.42/3.57 (H-15$_{1/2}$/H-14), 1.32/3.57 (H-15$_{1/2}$/H-14).

^{1}H,^{13}C-GHSQC (400 MHz / 101 MHz, [D1]-chloroform, 298 K) δ (^{1}H) / δ (^{13}C) [in ppm] = 7.97/129.54 (H-4/C-4), 7.90/127.97 (H-6/C-6), 7.59/125.51 (H-12/C-12), 7.43-7.39/126.40, 124.90 (H-7, 9/C-7, 9), 7.29-7.25/126.12 (H-8/C-8), 4.28/98.53 (H-19$_{1/2}$/C-19), 4.26/98.53 (H-19$_{1/2}$/C-19), 4.10-4.06/74.18 (H-17/C-17), 4.03-4.01/71.26 (H-18/C-18), 3.57/26.27 (H-14/C-14), 2.26/55.58 (H-20/C-20), 1.42/24.31 (H-15$_{1/2}$/C-15), 1.32/24.23 (H-15$_{1/2}$/C-15).

^{1}H,^{13}C-GHMBC (400 MHz / 101 MHz, [D$_1$]-chloroform, 298 K) δ (^{1}H) / δ (^{13}C) [in ppm] = 7.97/151.99, 135.21, 133.00, 127.97 (H-4/C-2, 11, 10, 6), 7.90/133.00, 129.54, 126.12 (H-6/C-10, 4, 8), 7.59/152.14, 141.72, 135.65, 26.27 (H-12/C-16, 13, 3, 14), 7.43-7.39/130.94, 126.29 (H-7, 9/C-5, 1), 7.29-7.25/133.00, 127.97 (H-8/C-10, 6), 4.28/151.99, 55.58 (H-19$_{1/2}$/C-2, 20), 4.26/151.99, 55.58 (H-19$_{1/2}$/C-2, 20), 3.57/152.14, 141.72, 125.51, 24.31, 24.23 (H-14/C-16, 13, 12, 15), 1.42/141.72 (H-15$_{1/2}$/C-13), 1.32/141.72 (H-15$_{1/2}$/C-13).

MS (ESI-pos, MeOH): *m/z* = 1616.8601 ([M+Na]$^+$, calcd. 1616.8604 [$C_{104}H_{120}O_{14}Na$]$^+$).

IR (ATR-FT): $\tilde{\nu}$ (cm^{-1}) = 703, 748, 764, 737, 897, 1080, 1191, 1263, 1275, 1457, 2928, 3000, 3005, 3052.

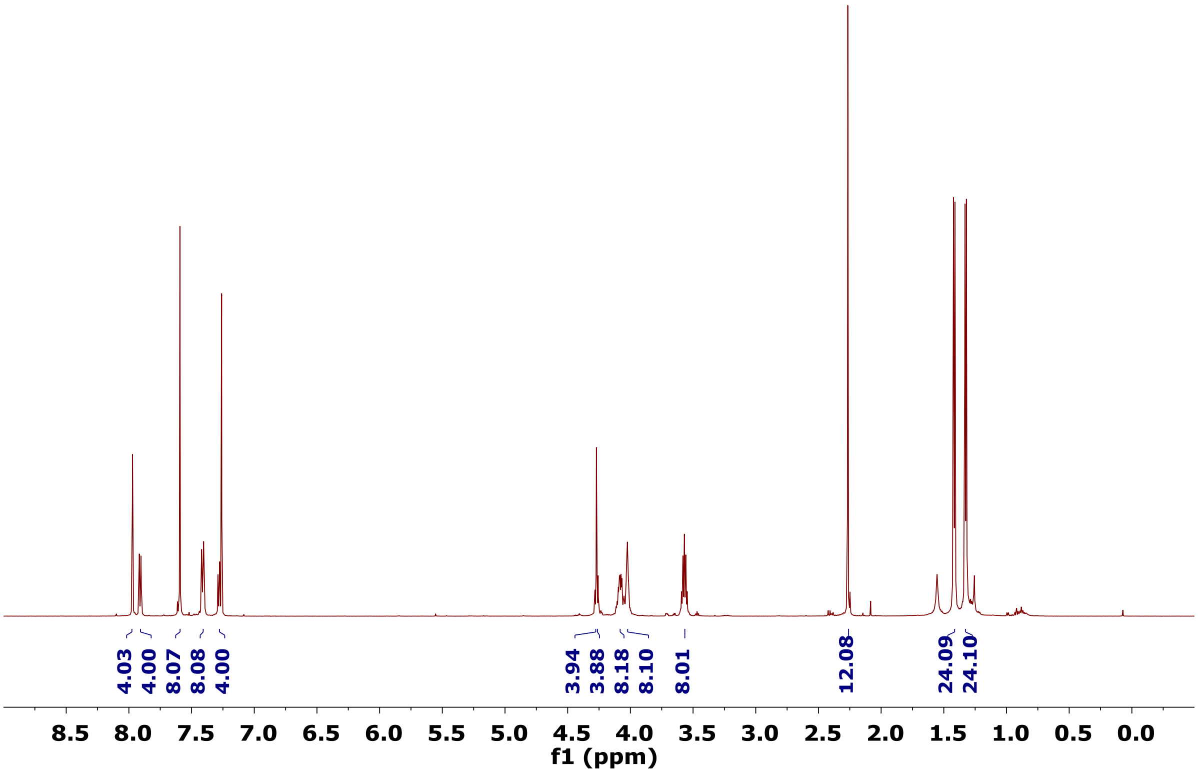

Figure S63: ^{1}H NMR spectrum of (*S,S*)-**iPr-M2$_2$** ($CDCl_3$, 298 K, 400 MHz).

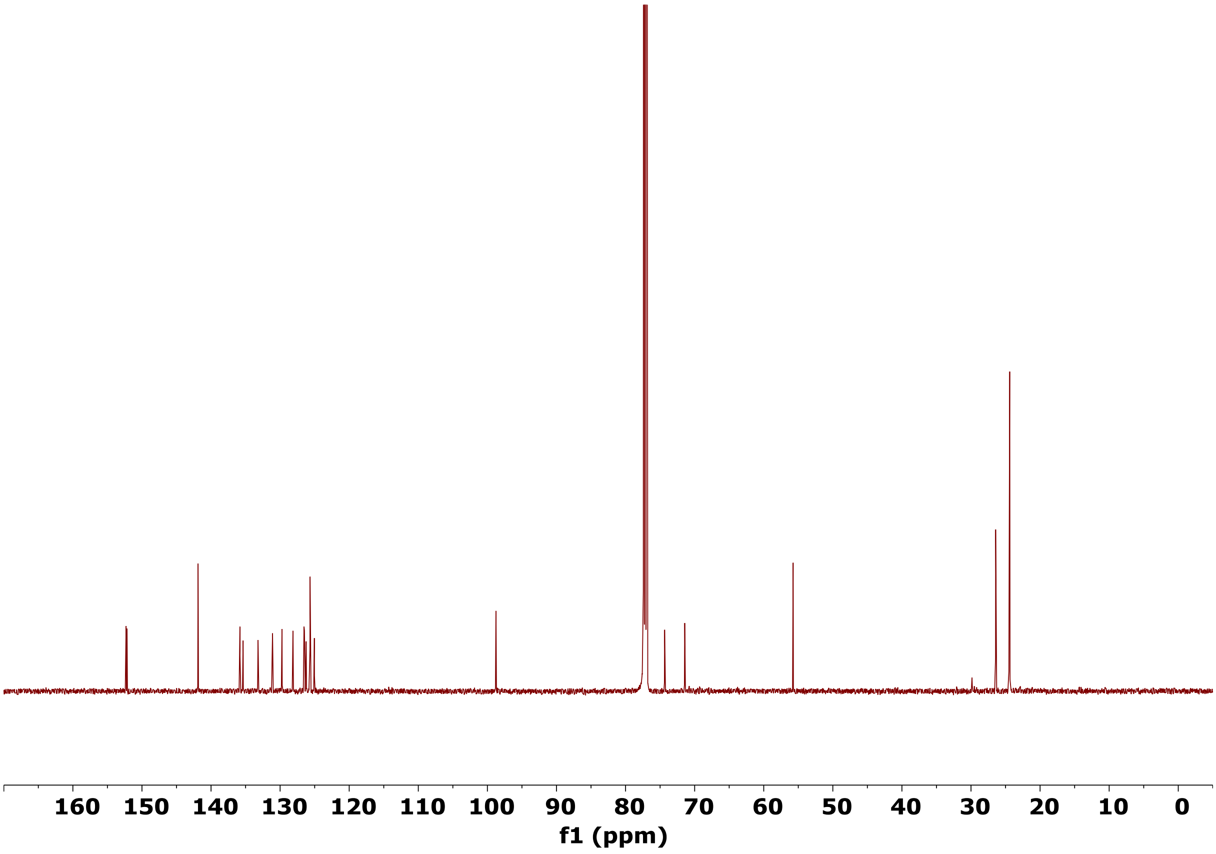

Figure S64: ^{13}C NMR spectrum of (*S,S*)-**iPr-M2$_2$** ($CDCl_3$, 298 K, 400 MHz).

Compound (*S*,*S*)-**HiPr-M2$_2$**

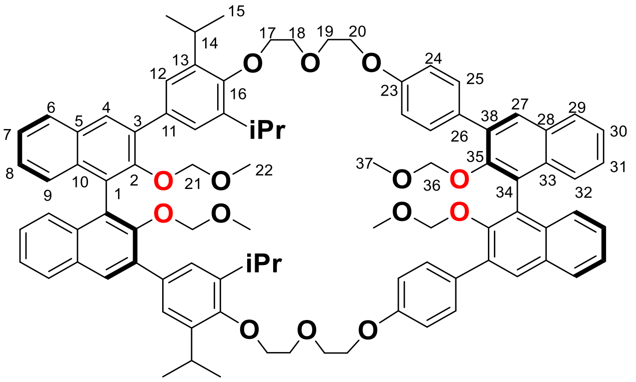

As described before (General procedure **D**), compound (*S*,*S*)-**HiPr-M2$_2$** was synthesized using compound **iPr-2** (16.1 mg, 0.0221 mmol, 1.0 equiv), cesium carbonate (21.1 mg, 0.0647 mmol, 3.2 equiv) and **H-6$_2$** (15.1 mg, 0.0196 mmol, 1.0 equiv) dissolved in degassed acetonitrile (2.5 mL). After purification by column chromatography (cyclohexane/ethyl acetate 6:1) the product was obtained as a colorless wax (22.3 mg, 0.0156 mmol, 72.7% yield).

$C_{92}H_{96}O_{14}$: 1425.77 g/mol.

^{1}H-NMR (400 MHz, [D$_1$]-chloroform, 298 K) δ [in ppm] = 7.93 (s, 2H, H-4), 7.92 (s, 2H, H-27), 7.87 (t, *J* = 7.3 Hz, 4H, H-6+29), 7.72 (d, *J* = 8.7 Hz, 4H, H-24), 7.51 (s, 4H, H-12), 7.41-7.37 (m, 4H, H-7+30), 7.30-7.22 (m, 8H, H-8+31, 9+32), 7.07 (d, *J* = 8.7 Hz, 4H, H-25), 4.38 (d, *J* = 2.3 Hz, 2H, H-$21_{1/2}$), 4.37 (d, *J* = 2.2 Hz, 2H, H-$36_{1/2}$), 4.32 (d, *J* = 2.2 Hz, 2H, H-$36_{1/2}$), 4.31 (d, *J* = 2.3 Hz, 2H, H-$21_{1/2}$), 4.29-4.26 (m, 4H, H-17), 4.05-4.01 (m, 8H, H-18, 19), 3.99-3.97 (m, 4H, H-20), 3.54 (hept., *J* = 6.8 Hz, 4H, H-14), 2.33 (s, 6H, H-22), 2.32 (s, 6H, H-37), 1.34 (d, *J* = 6.9 Hz, 12H, H-$15_{1/2}$), 1.27 (d, *J* = 6.9 Hz, 12H, H-$21_{1/2}$).

^{13}C-NMR (101 MHz, [D$_1$]-chloroform, 298 K) δ [in ppm] = 158.37 (C-23), 152.39 (C-16), 151.69 (C-2+35), 141.77 (C-13), 135.72 (C-3), 135.22 (C-11), 135.04 (C-26), 133.46 (C-10), 133.38 (C-33), 131.67 (C-38), 130.87 (C-1+34)*, 130.81 (C-5+28)*, 130.75 (C-24), 130.07 (C-4), 129.91 (C-27), 127.78 (C-6+29)*, 126.47 (C-9), 126.43 (C-32), 126.09 (C-31), 125.95 (C-8), 125.52 (C-12), 125.03 (C-7), 124.90 (C-30), 114.64 (C-25), 98.58 (C-21), 98.26 (C-36), 74.42, 69.96 (C-18, 19), 70.75 (C-20), 67.87 (C-17), 55.87 (C-22), 55.67 (C-37), 26.32 (C-14), 24.29 (C-$15_{1/2}$), 24.16 (C-$15_{1/2}$).

* Partially overlapping signals.

^{1}H,^{1}H-COSY (400 MHz / 400 MHz, [D$_1$]-chloroform, 298 K) δ [in ppm] = 7.87/7.41-7.37 (H-6+29/H-7+30), 7.72/7.07 (H-24/H-25), 7.41-7.37/7.87, 7.30-7.22 (H-7+30/H-6+29, 8+31), 7.30-7.22/7.37 (H-8+31/H-7+30), 7.07/7.72 (H-25/H-24), 4.38/4.31 (H-$21_{1/2}$/H-$21_{1/2}$), 4.37/4.32 (H-$36_{1/2}$/H-$36_{1/2}$), 4.32/4.37 (H-$36_{1/2}$/H-$36_{1/2}$), 4.31/4.38 (H-$21_{1/2}$/H-$21_{1/2}$), 3.54/1.34, 1.27 (H-14/H-15), 1.34/3.54 (H-$15_{1/2}$/H-14), 1.27/3.54 (H-$15_{1/2}$/H-14).

^{1}H,^{13}C-GHSQC (400 MHz / 101 MHz, [D$_1$]-chloroform, 298 K) δ (^{1}H) / δ (^{13}C) [in ppm] = 7.93/130.07 (H-4/C-4), 7.92/129.91 (H-27/C-27), 7.87/127.78 (H-6+29/C-6+29), 7.72/130.75 (H-24/C-24), 7.51/125.52 (H-12/C-12), 7.41-7.37/125.03, 124.90 (H-7+30/C-7, 30), 7.30-7.22/126.47, 126.43, 126.09, 125.95 (H-9+32, 8+31/C-9, 32, 31, 8), 7.07/114.64 (H-25/C-25), 4.38/98.58 (H-$21_{1/2}$/C-21), 4.37/98.26 (H-$36_{1/2}$/C-36), 4.32/98.26 (H-$36_{1/2}$/C-36), 4.31/98.58 (H-$21_{1/2}$/C-21), 4.29-4.26/67.87 (H-17/C-17), 4.05-4.01/74.42, 69.96 (H-18, 19/C-18, 19), 3.99-3.97/70.75 (H-20/C-20), 3.54/26.32 (H-14/C-14), 2.33/55.87 (H-22/C-22), 2.32/55.67 (H-37/C-37), 1.34/24.29 (H-$15_{1/2}$/C-$15_{1/2}$), 1.27/24.16 (H-$15_{1/2}$/C-$15_{1/2}$).

^{1}H,^{13}C-GHMBC (400 MHz / 101 MHz, [D_1]-chloroform, 298 K) δ (^{1}H) / δ (^{13}C) [in ppm] = 7.93/151.69, 135.22, 133.46, 127.78 (H-4/C-2, 11, 10, 6), 7.92/151.69, 135.04, 133.38, 127.78 (H-27/C-35, 26, 33, 29), 7.87/133.46, 133.38, 130.07, 129.91, 126.47, 126.43, 126.09, 125.95 (H-6+29/C-10, 33, 4, 27, 31, 8), 7.72/158.37, 135.04 (H-24/C-23, 26), 7.51/152.39, 135.72, 26.32 (H-12/C-16, 3, 14), 7.41-7.37/130.81, 126.47, 126.43(H-7+30/C-5+28, 9, 32), 7.30-7.22/133.46, 133.38, 130.87, 130.81, 127.78, 125.03, 124.90 (H-8+31, 9+32/C-10, 33, 1+34, 5+28, 6+29, 7, 30), 7.07/158.37, 131.67 (H-25/C-23, 38), 4.38-4.31/151.69, 55.87, 55.67 (H-$21_{1/2}$, $36_{1/2}$/C-2+35, 22, 37), 3.54/152.39, 125.52 (H-14/C-16, 12), 2.33/98.58 (H-22/C-21), 2.32/98.26 (H-37/C-36), 1.34, 1.27/141.77 (H-$21_{1/2}$/C-13).

MS (ESI-pos, MeOH**)**: *m/z* = 1448.6662 ($[M+Na]^+$, calcd. 1448.6726 $[C_{92}H_{96}O_{14}Na]^+$).

IR (ATR-FT): $\tilde{\nu}$ (cm^{-1}) = 701, 750, 764, 996, 1035, 1097, 1261, 1275, 1457, 1472, 2872, 2988, 3005, 3055.

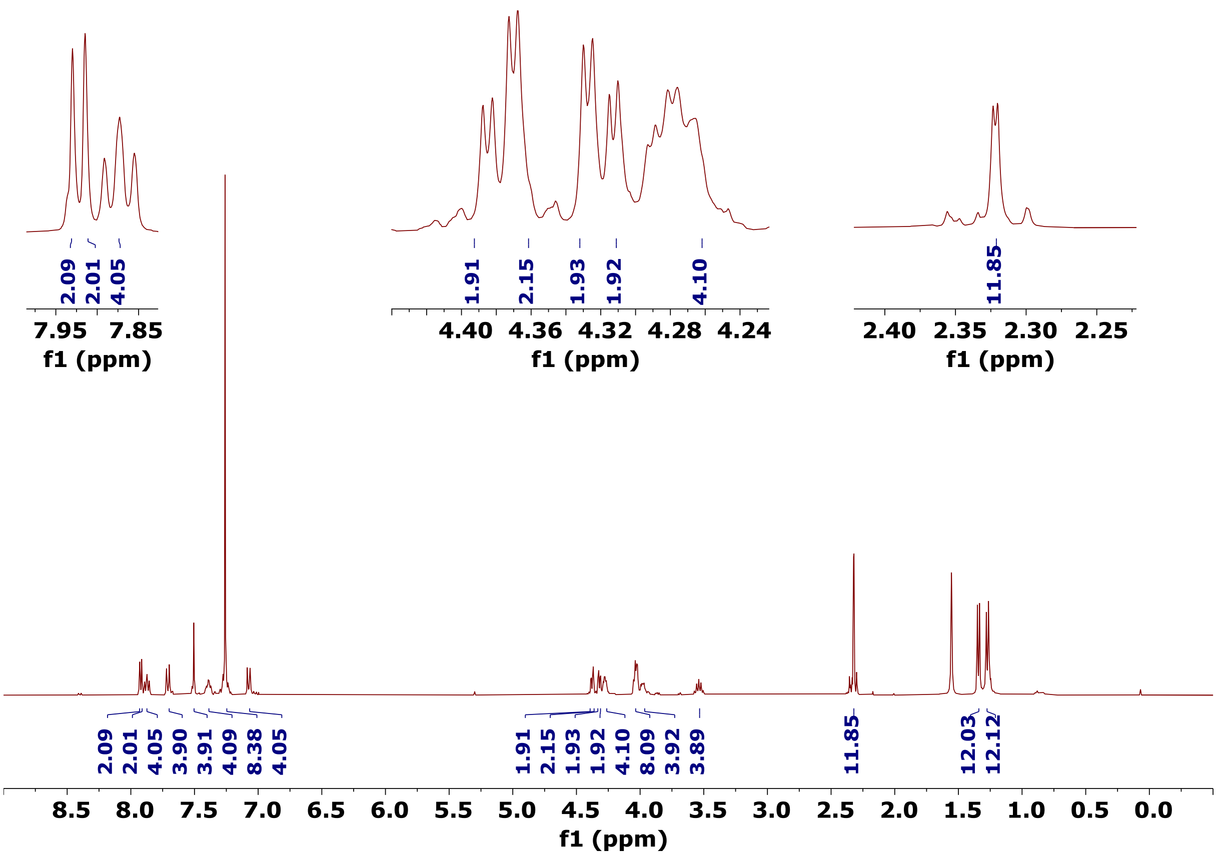

Figure S65: ^{1}H NMR spectrum of (*S,S*)-**HiPr-M2$_2$** ($CDCl_3$, 298 K, 400 MHz).

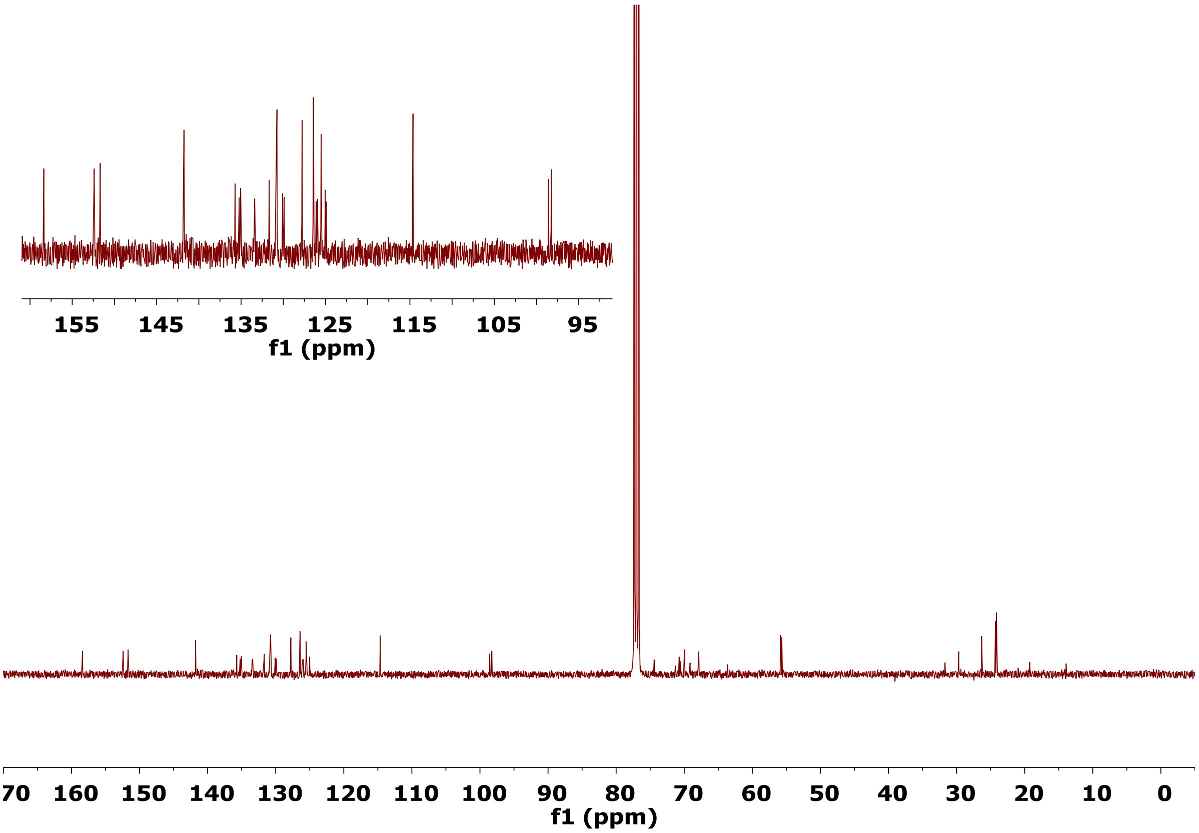

Figure S66: ^{13}C NMR spectrum of (*S,S*)-**HiPr-M2$_2$** ($CDCl_3$, 298 K, 400 MHz).

Compound (*R*,*S*)-HiPr-M2$_2$

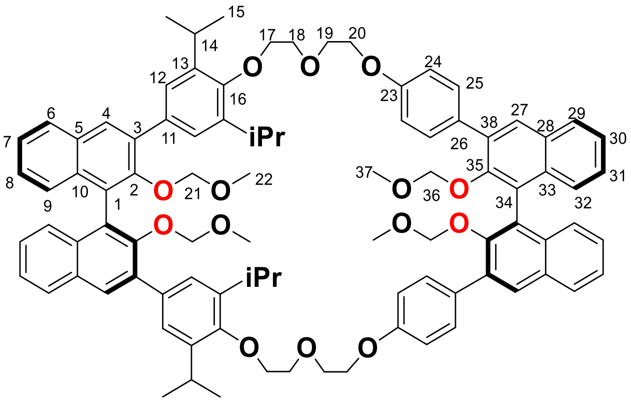

As described above (General procedure **D**), compound (*R*,*S*)-**HiPr-M2$_2$** was synthesized using compound **iPr-2** (28.1 mg, 0.0386 mmol, 1.0 equiv), cesium carbonate (39.0 mg, 0.120 mmol, 3.2 equiv) and (*R*)-**H-6$_2$** (28.8 mg, 0.0373 mmol, 1.0 equiv) dissolved in degassed acetonitrile (4 ml). After purification by column chromatography (cyclohexane/ethyl acetate 6.1) the product was obtained as a colorless wax (26.9 mg, 0.0189 mmol, 48.9% yield).

$C_{92}H_{96}O_{14}$: 1425.77 g/mol.

^{1}H-NMR (400 MHz, [D$_1$]-chloroform, 298 K) δ [in ppm] = 7.93 (s, 2H, H-4), 7.92 (s, 2H, H-27), 7.89 (d, *J* = 5.2 Hz, 4H, H-6), 7.87 (d, *J* = 5.2 Hz, 4H, H-29), 7.72 (d, *J* = 8.7 Hz, 4H, H-24), 7.51 (s, 4H, H-12), 7.41-7.37 (m, 4H, H-7+30), 7.30-7.22 (m, 8H, H-9+32, 8+31), 7.08 (d, *J* = 8.7 Hz, 4H, H-25), 4.38 (d, *J* = 6.0 Hz, 2H, H-21$_{1/2)}$, 4.37 (d, *J* = 5.9 Hz, 2H, H-36$_{1/2}$), 4.32 (d, *J* = 5.9 Hz, 2H, H-36$_{1/2}$), 4.31 (d, *J* = 6.0 Hz, 2H, H-21$_{1/2}$), 4.30-4.26 (m, 4H, H-17), 4.05-4.01 (m, 8H, H-18, 19), 3.99-3.97 (m, 4H, H-20), 3.55 (hept., *J* = 6.8 Hz, 4H, H-14), 2.33 (s, 6H, H-22), 2.32 (s, 6H, H-37), 1.34 (d, *J* = 6.9 Hz, 12H, H-15$_{1/2}$), 1.27 (d, *J* = 6.9 Hz, 12H, H-15$_{1/2}$).

^{13}C-NMR (101 MHz, [D$_1$]-chloroform, 298 K) δ [in ppm] = 158.40 (C-23), 152.39 (C-16), 151.67 (C-2+35), 141.74 (C-13), 135.78 (C-3), 135.25 (C-11), 135.01 (C-26), 133.46 (C-10), 133.38 (C-33), 131.65 (C-38), 130.89 (C-1+34)*, 130.80 (C-5+28)*, 130.74 (C-24), 130.05 (C-4), 129.92 (C-27), 127.78 (C-6+29)*, 126.45 (C-9 or 32)*, 126.43 (C-9 or 32)*, 126.08 (C-8), 125.95 (C-31), 125.52 (C-12), 125.04 (C-7), 124.89 (C- 30), 114.69 (C-25), 98.58 (C-21), 98.28 (C-36), 74.45 (C-19), 70.77 (C-20), 70.01 (C-18), 67.90 (C-17), 55.88 (C-22), 55.67 (C-37), 26.33 (C-14), 24.26 (C-15$_{1/2}$), 24.20 (C-15$_{1/2}$).

* Partially overlapping signals.

^{1}H,^{1}H-COSY (400 MHz / 400 MHz, [D$_1$]-chloroform, 298 K) δ [in ppm] = 7.89/7.41-7.37 (H-6/H-7+30), 7.87/7.41-7.37 (H-29/H-7+30), 7.72/7.08 (H-24/H-25), 7.41-7.37/7.89, 7.87, 7.30-7.22 (H-7+30/H-6, 29, 8+31), 7.30-7.22/7.41-7.37 (H-8+31/H-7+30), 7.08/7.72 (H-25/H-24), 4.38/4.31 (H-21$_{1/2}$/H-21$_{1/2}$), 4.37/4.32 (H-36$_{1/2}$/H-36$_{1/2}$), 4.32/4.37 (H-36$_{1/2}$/H-36$_{1/2}$), 4.31/4.38 (H-21$_{1/2}$/H-21$_{1/2}$), 3.55/1.34, 1.27 (H-14/H-15), 1.34/3.55 (H-15$_{1/2}$/H-14), 1.27/3.55 (H-15$_{1/2}$/H-14).

^{1}H,^{13}C-GHSQC (400 MHz / 101 MHz, [D$_1$]-chloroform, 298 K) δ (^{1}H) / δ (^{13}C) [in ppm] = 7.93, /130.05 (H-4/C-4), 7.92/129.92 (H-27/C-27), 7.89/127.78 (H-6/C-6), 7.87/127.78 (H-29/C-29), 7.72/130.74 (H-24/C-24), 7.51/125.52 (H-12/C-12), 7.41-7.37/125.04, 124.89 (H-7+30/C-7, 30), 7.30-7.22/126.45, 126.43, 126.08, 125.95 (H-9+32, 8+31/C-9 or 32, 8, 31), 7.08/114.69 (H-25/C-25), 4.38/98.58 (H-$21_{1/2}$/C-21), 4.37/98.28 (H-$36_{1/2}$/C-36), 4.32/98.28 (H-$36_{1/2}$/C-36), 4.31/98.58 (H-$21_{1/2}$/C-21), 4.30-4.26/67.90 (H-17/C-17), 4.05-4.01/74.45, 70.01 (H-18, 19/C-19, 18), 3.99-3.97/70.77 (H-20/C-20), 3.55/26.33 (H-14/C-14), 2.33/55.88 (H-22/C-22), 2.32/55.67 (H-37/C-37), 1.34/24.26 (H-$15_{1/2}$/C-$15_{1/2}$), 1.27/24.20 (H-$15_{1/2}$/C-$15_{1/2}$).

^{1}H,^{13}C-GHMBC (400 MHz / 101 MHz, [D$_1$]-chloroform, 298 K) δ (^{1}H) / δ (^{13}C) [in ppm] = 7.93/151.67, 135.25, 133.46, 127.78 (H-4/C-2+35, 11, 10, 6+29), 7.92/151.67, 133.38, 127.78 (H-27/C-2+35, 33, 26, 6+29), 7.89/133.46, 130.05, 126.08 (H-6/C-10, 4, 8), 7.87/133.38, 129.92 125.95 (H-29/C-33, 27, 31), 7.72/135.01 (H-24/C-26), 7.51/152.39, 135.78, 26.33 (H-12/C-16, 3, 14), 7.41-7.37/130.80, 126.45, 126.43 (H-7+30/C-5+28, 9 or 32), 7.30-7.22/133.46, 133.38, 130.89, 130.80, 127.78 (H-9+32, 8+31/C-10, 33, 1+34, 5+28, 6+29), 7.08/158.40, 131.65 (H-25/C-23, 38), 4.38/151.67, 55.88 (H-$21_{1/2}$/C-2+35, 22), 4.37/151.67, 55.67 (H-$36_{1/2}$/C-2+35, 37), 4.32/151.67, 55.67 (H-$36_{1/2}$/C-2+35, 37), 4.31/151.67, 55.88 (H-$21_{1/2}$/C-2+35, 22), 3.55/152.39, 125.52 (H-14/C-16, 12), 2.33/98.58 (H-22/C-21), 2.32/98.28 (H-37/C-36), 1.34, 1.27/141.74 (H-$15_{1/2}$/C-13).

MS (ESI-pos, MeOH**)**: *m/z* = 1448.6666 ([M+Na]$^+$, calcd. 1448.6726 [$C_{92}H_{96}O_{14}Na$]$^+$).

IR (ATR-FT): $\tilde{\nu}$ (cm^{-1}) = 702, 731, 749, 764, 1100, 1264, 1275, 1457, 2872, 2987, 3004, 3053.

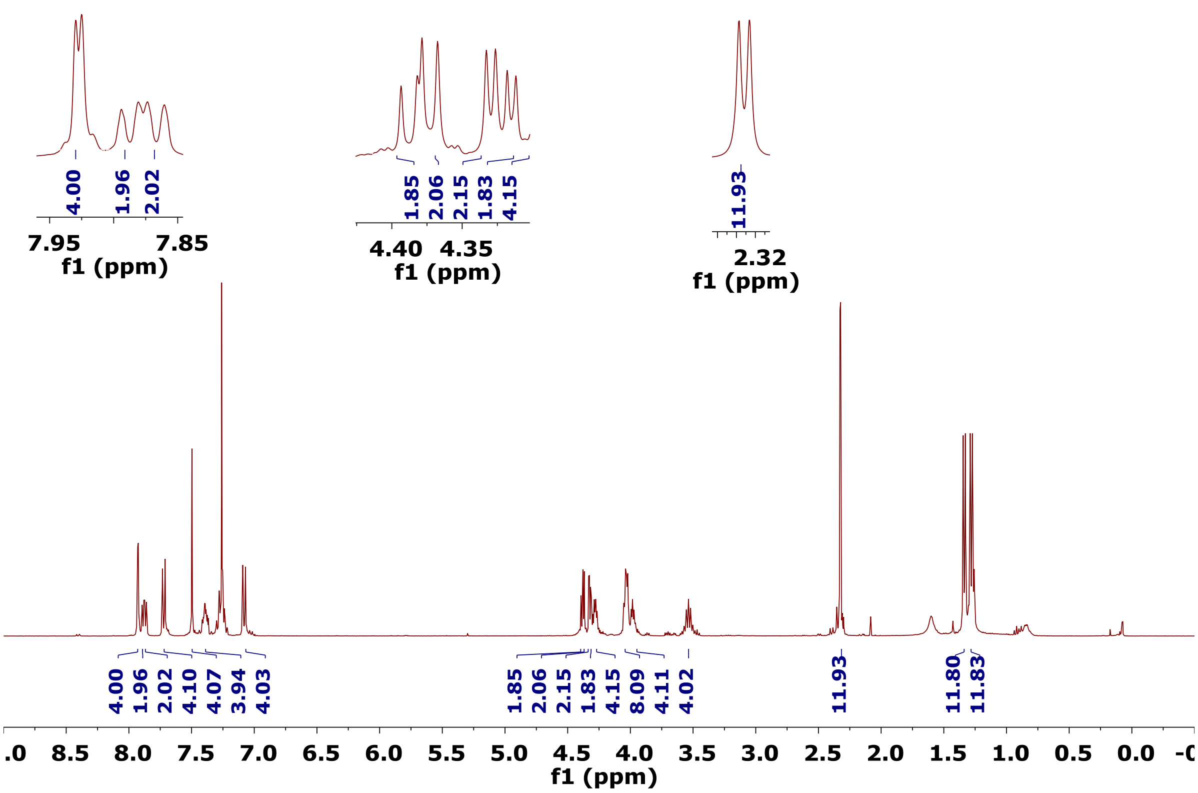

Figure S67: ^{1}H NMR spectrum of (*R,S*)-**HiPr-M2$_2$** ($CDCl_3$, 298 K, 400 MHz).

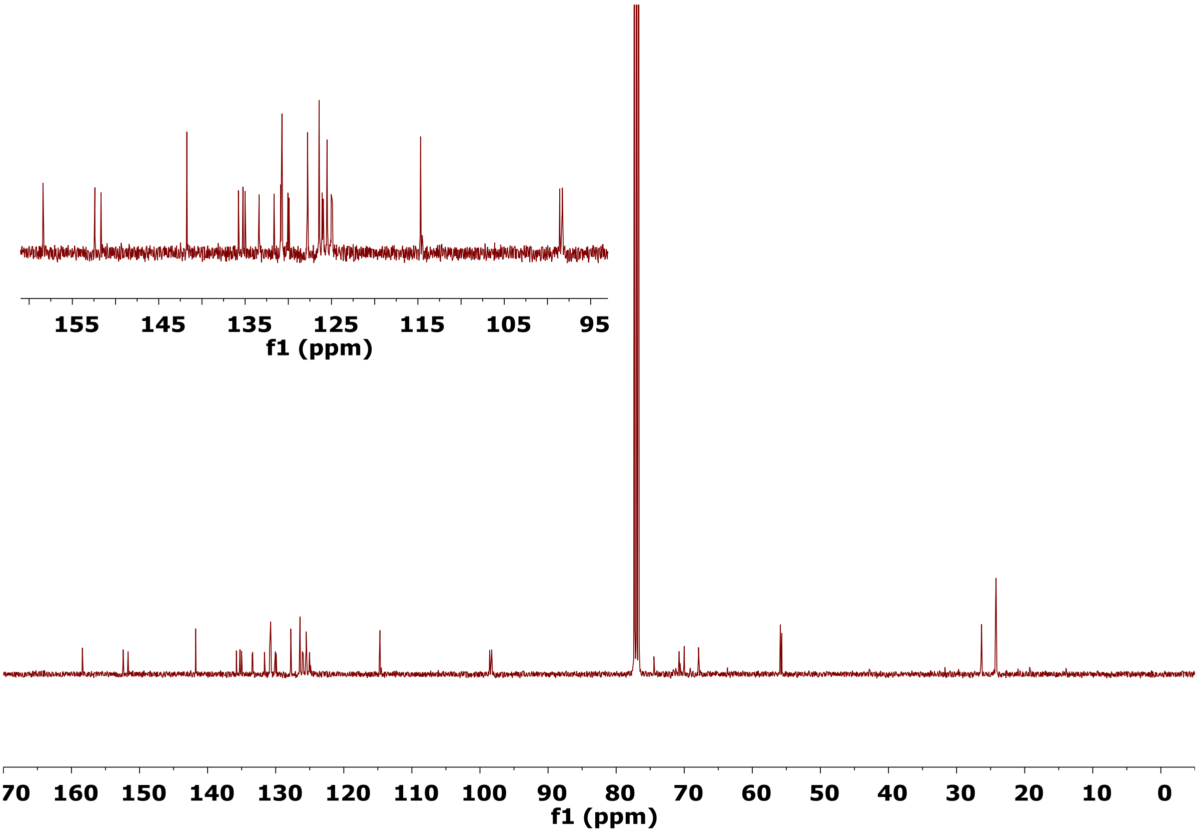

Figure S68: ^{13}C NMR spectrum of (*R,S*)-**HiPr-M2$_2$** ($CDCl_3$, 298 K, 400 MHz).

3. Crystal structure of Me-M1$_6$

The crystals were mounted on nylon loops in inert oil. Data were collected on a Bruker AXS D8 Venture diffractometer with Photon II detector (mono-chromated Cu$_{K\alpha}$ radiation, λ = 1.54178 Å, mircofocus source) at 100(2) K. The structures were solved by Direct Methods (SHELXS-2013)[4] and refined anisotropically by full-matrix least-squares on F^2 (SHELXL-2017)[5,6]. Absorption corrections were performed semi-empirically from equivalent reflections on basis of multi-scans (Bruker AXS APEX3). Hydrogen atoms were refined using a riding model or rigid methyl groups.

CCDC-2427523 contains the supplementary crystallographic data for this paper. These data can be obtained free of charge from The Cambridge Crystallographic Data Centre via www.ccdc.cam.ac.uk/data_request/cif. The absolute structure could *not* be determined[7] reliably from the measurement but was chosen to match the known chirality of a stereocenter. Refinement as inversion twin led to a minor component of approximate 7% with a high standard uncertainty rendering this value meaningless. Thus, the twinning model was discarded. The polyether chain is partially disordered. The corresponding bond lengths and angles were restrained to be equal. RIGU and SIMU restraints were applied to the displacement parameters of the disordered atoms.

Table S1: Crystal structure data

**Identification code**	**mt_842_1m**
Empirical formula	$C_{52}H_{60}O_{11}$
Formula weight	861.00
Density (calculated)	1.286 g·cm^{-3}
F(000)	1840
Temperature	100(2) K
Crystal size	0.644 × 0.204 × 0.196 mm
Crystal color	colorless
Crystal description	prism
Wavelength	1.54178 Å
Crystal system	tetragonal
Space group	$P4_12_12$
Unit cell dimensions	
a [Å]	9.7530(5)
b [Å]	9.7530(5)
c [Å]	46.740(2)
α [°]	90
β [°]	90
γ [°]	90
Volume	4445.9(5) Å^3
Z	4
Cell measurement reflections used	9822
Cell measurement ϑ min/max	3.78°/79.56°

Diffractometer control software	Bruker APEX3(v2017.3-0)
Diffractometer measurement device	Bruker D8 Venture (Photon II detector)
Diffractometer measurement method	Data collection strategy APEX 3/Queen
ϑ range for data collection	3.783°- 79.596°
Completeness to ϑ = 67.679°	100.0%
Completeness to ϑ_{max} = 79.596°	99.9%
Index ranges	$-12 \leq h \leq 12$
	$-12 \leq k \leq 12$
	$-53 \leq l \leq 59$
Computing data reduction	Bruker APEX3(v2017.3-0)
Absorption coefficient	0.725 mm^{-1}
Absorption correction	Semi-empirical from equivalents
Computation absorption correction	SADABS
Max./min. Transmission	0.75/0.63
R_{merg} before/after correction	0.1495/0.0887
Computing structure solution	Bruker APEX3(v2017.3-0)
Computing structure refinement	SHELXL-2017/1 (Sheldrick, 2017)
Refinement method	Full-matrix least-squares on F^2
Reflections collected	217218
Independent reflections	4853
R_{int}	0.0632
Reflections with $I > 2\sigma(I)$	4801
Restraints	8
Parameter	298
GooF	1.110
Weighting details	$w = 1/[\sigma^2(F_{obs}^2) + (0.0436P)^2 + 1.3502P]$
	where $P = (F_{obs}^2 + 2F_{calc}^2)/3$
R_1 $[I > 2\sigma(I)]$	0.0331
wR_2 $[I > 2\sigma(I)]$	0.0905
R_1 [all data]	0.0334
wR_2 [all data]	0.0908
Absolute structure parameter	0.11(2)
Largest diff. peak and hole	0.214/-0.229

Table S2: Atomic coordinates (× 10^4) and equivalent isotropic displacement parameters (Å^2 × 10^3) for mt_842_1m. U_{eq} is defined as one third of the trace of the orthogonalized U_{ij} tensor.

	x	*y*	*z*	U_{eq}
O(1)	9514(1)	8807(1)	331(1)	21(1)
O(2)	10106(2)	6678(1)	529(1)	27(1)
O(3)	10012(1)	12870(1)	1381(1)	27(1)
O(4)	13014(2)	12464(2)	1299(1)	29(1)
O(5)	14072(2)	12568(2)	714(1)	33(1)
O(6)	13876(2)	13876(2)	0	62(1)
C(1)	7410(2)	8192(2)	110(1)	20(1)
C(2)	8095(2)	8842(2)	330(1)	19(1)
C(3)	7376(2)	9612(2)	543(1)	21(1)
C(4)	5980(2)	9694(2)	526(1)	22(1)
H(4)	5495	10175	671	27
C(5)	5236(2)	9086(2)	300(1)	22(1)
C(6)	3804(2)	9302(2)	270(1)	25(1)
H(6)	3323	9813	411	30
C(7)	3108(2)	8783(2)	40(1)	28(1)
H(7)	2154	8955	19	33
C(8)	3813(2)	7991(2)	-166(1)	29(1)
H(8)	3325	7622	-324	35
C(9)	5190(2)	7748(2)	-141(1)	24(1)
H(9)	5641	7194	-279	29
C(10)	5954(2)	8313(2)	90(1)	21(1)
C(11)	10127(2)	8094(2)	566(1)	24(1)
H(11A)	11089	8401	587	28
H(11B)	9631	8330	744	28
C(12)	11035(2)	6210(2)	314(1)	29(1)
H(12A)	10846	6688	134	44
H(12B)	10917	5221	287	44
H(12C)	11978	6400	374	44
C(13)	8110(2)	10400(2)	770(1)	21(1)
C(14)	7729(2)	10285(2)	1057(1)	22(1)
H(14)	7037	9649	1110	26
C(15)	8351(2)	11091(2)	1269(1)	22(1)
C(16)	9372(2)	12012(2)	1184(1)	22(1)
C(17)	9729(2)	12192(2)	897(1)	23(1)
C(18)	9109(2)	11352(2)	694(1)	22(1)
H(18)	9374	11429	499	27
C(19)	7880(2)	10990(2)	1575(1)	27(1)
H(19A)	6935	10649	1581	41
H(19B)	7920	11898	1664	41
H(19C)	8478	10357	1680	41
C(20)	10710(2)	13296(2)	806(1)	29(1)
H(20A)	10905	13203	601	43
H(20B)	11565	13207	915	43
H(20C)	10301	14196	843	43
C(21)	10962(2)	12218(2)	1574(1)	31(1)
H(21A)	10451	11545	1691	37
H(21B)	11332	12924	1705	37
C(22)	12146(2)	11494(2)	1433(1)	29(1)
H(22A)	12671	10976	1579	35
H(22B)	11798	10835	1290	35
C(23)	14247(2)	11860(2)	1195(1)	31(1)
H(23A)	14051	10944	1113	37
H(23B)	14909	11747	1354	37
C(24)	14846(2)	12778(2)	968(1)	32(1)
H(24A)	14788	13749	1029	39
H(24B)	15821	12546	935	39
C(25)	14605(11)	13197(16)	467(2)	34(2)
H(25A)	15482	12755	413	41
H(25B)	14786	14178	505	41
C(25')	14272(11)	13573(10)	503(2)	32(2)
H(25C)	15264	13734	473	38
H(25D)	13842	14447	563	38
C(26)	13627(3)	13067(2)	234(1)	38(1)
H(26A)	13612	12098	171	45
H(26B)	12703	13289	308	45
H(26C)	13971	12131	194	45
H(26D)	12623	13004	263	45

Table S3: Anisotropic displacement parameters (Å^2 × 10^3) for mt_842_1m. The anisotropic displacement factor exponent takes the form: $-\pi^2[h^2a^{*2}U_{11} + ... + 2hka^*b^*U_{12}]$

	U_{11}	U_{22}	U_{33}	U_{23}	U_{13}	U_{12}
O(1)	21(1)	25(1)	17(1)	0(1)	0(1)	1(1)
O(2)	30(1)	26(1)	26(1)	3(1)	1(1)	2(1)
O(3)	26(1)	29(1)	26(1)	-8(1)	-4(1)	1(1)
O(4)	28(1)	31(1)	28(1)	-2(1)	3(1)	6(1)
O(5)	35(1)	40(1)	24(1)	1(1)	1(1)	2(1)
O(6)	76(1)	76(1)	34(1)	22(1)	-22(1)	-38(2)
C(1)	24(1)	21(1)	14(1)	2(1)	2(1)	0(1)
C(2)	20(1)	21(1)	16(1)	2(1)	1(1)	0(1)
C(3)	25(1)	22(1)	15(1)	1(1)	1(1)	0(1)
C(4)	26(1)	24(1)	17(1)	-2(1)	4(1)	2(1)
C(5)	23(1)	24(1)	18(1)	2(1)	2(1)	0(1)
C(6)	25(1)	27(1)	22(1)	2(1)	3(1)	2(1)
C(7)	22(1)	34(1)	26(1)	3(1)	-2(1)	1(1)
C(8)	28(1)	35(1)	24(1)	-2(1)	-6(1)	-2(1)
C(9)	25(1)	28(1)	19(1)	-2(1)	-1(1)	-1(1)
C(10)	22(1)	23(1)	16(1)	2(1)	0(1)	0(1)
C(11)	24(1)	27(1)	20(1)	1(1)	-3(1)	4(1)
C(12)	31(1)	28(1)	29(1)	-2(1)	0(1)	5(1)
C(13)	23(1)	22(1)	19(1)	-3(1)	-1(1)	3(1)
C(14)	22(1)	24(1)	20(1)	-2(1)	2(1)	2(1)
C(15)	22(1)	26(1)	19(1)	-2(1)	0(1)	4(1)
C(16)	23(1)	23(1)	20(1)	-5(1)	-2(1)	5(1)
C(17)	22(1)	23(1)	23(1)	0(1)	-1(1)	2(1)
C(18)	25(1)	26(1)	17(1)	0(1)	1(1)	2(1)
C(19)	27(1)	36(1)	19(1)	-5(1)	2(1)	3(1)
C(20)	29(1)	29(1)	28(1)	1(1)	-1(1)	-5(1)
C(21)	27(1)	44(1)	22(1)	-5(1)	-4(1)	0(1)
C(22)	28(1)	34(1)	24(1)	1(1)	-4(1)	2(1)
C(23)	31(1)	36(1)	26(1)	-1(1)	2(1)	10(1)
C(24)	28(1)	42(1)	27(1)	0(1)	0(1)	5(1)
C(25)	25(4)	49(6)	29(3)	6(3)	3(2)	4(3)
C(25')	28(4)	39(4)	29(2)	3(2)	2(2)	1(2)
C(26)	46(1)	40(1)	27(1)	1(1)	1(1)	-2(1)

Table S4: Bond lengths [Å] for mt_842_1m.

O(1)-C(2)	1.385(2)
O(1)-C(11)	1.429(2)
O(2)-C(11)	1.391(2)
O(2)-C(12)	1.428(2)
O(3)-C(16)	1.391(2)
O(3)-C(21)	1.441(2)
O(4)-C(22)	1.416(2)
O(4)-C(23)	1.424(2)
O(5)-C(25')	1.403(7)
O(5)-C(25)	1.408(9)
O(5)-C(24)	1.424(2)
O(6)-C(26)#1	1.368(3)
O(6)-C(26)	1.368(3)
C(1)-C(2)	1.380(2)
C(1)-C(10)	1.427(2)
C(1)-C(1)#1	1.491(3)
C(2)-C(3)	1.430(2)
C(3)-C(4)	1.366(3)
C(3)-C(13)	1.494(2)
C(4)-C(5)	1.412(2)
C(5)-C(6)	1.420(3)
C(5)-C(10)	1.423(2)
C(6)-C(7)	1.367(3)
C(7)-C(8)	1.414(3)
C(8)-C(9)	1.369(3)
C(9)-C(10)	1.421(2)
C(13)-C(18)	1.391(3)
C(13)-C(14)	1.398(2)
C(14)-C(15)	1.400(2)
C(15)-C(16)	1.398(3)
C(15)-C(19)	1.508(2)
C(16)-C(17)	1.400(2)
C(17)-C(18)	1.390(2)
C(17)-C(20)	1.501(3)
C(21)-C(22)	1.504(3)
C(23)-C(24)	1.505(3)
C(25)-C(26)	1.454(9)
C(25')-C(26)	1.493(7)

#1 *y,x,-z*

Table S5: Bond angles [°] for mt_842_1m.

C(2)-O(1)-C(11)	115.69(13)
C(11)-O(2)-C(12)	113.25(15)
C(16)-O(3)-C(21)	115.89(15)
C(22)-O(4)-C(23)	112.32(16)
C(25')-O(5)-C(24)	114.3(3)
C(25)-O(5)-C(24)	115.3(4)
C(26)#1-O(6)-C(26)	115.6(3)
C(2)-C(1)-C(10)	119.54(15)
C(2)-C(1)-C(1)#1	120.09(15)
C(10)-C(1)-C(1)#1	120.34(14)
C(1)-C(2)-O(1)	118.41(15)
C(1)-C(2)-C(3)	121.46(16)
O(1)-C(2)-C(3)	120.01(15)
C(4)-C(3)-C(2)	118.65(16)
C(4)-C(3)-C(13)	119.26(16)
C(2)-C(3)-C(13)	121.97(16)
C(3)-C(4)-C(5)	122.04(16)
C(4)-C(5)-C(6)	121.16(17)
C(4)-C(5)-C(10)	119.10(16)
C(6)-C(5)-C(10)	119.63(17)
C(7)-C(6)-C(5)	120.77(18)
C(6)-C(7)-C(8)	119.74(18)
C(9)-C(8)-C(7)	120.83(18)
C(8)-C(9)-C(10)	120.89(17)
C(9)-C(10)-C(5)	118.08(16)
C(9)-C(10)-C(1)	122.70(16)
C(5)-C(10)-C(1)	119.15(16)
O(2)-C(11)-O(1)	112.49(14)
C(18)-C(13)-C(14)	118.95(16)
C(18)-C(13)-C(3)	119.87(15)
C(14)-C(13)-C(3)	120.95(17)
C(13)-C(14)-C(15)	121.19(17)
C(16)-C(15)-C(14)	118.01(16)
C(16)-C(15)-C(19)	121.84(16)
C(14)-C(15)-C(19)	120.11(17)
O(3)-C(16)-C(15)	121.33(16)
O(3)-C(16)-C(17)	116.56(17)
C(15)-C(16)-C(17)	121.94(16)
C(18)-C(17)-C(16)	118.12(17)
C(18)-C(17)-C(20)	120.56(16)
C(16)-C(17)-C(20)	121.28(17)
C(17)-C(18)-C(13)	121.65(16)
O(3)-C(21)-C(22)	115.31(15)
O(4)-C(22)-C(21)	109.83(17)
O(4)-C(23)-C(24)	108.74(17)
O(5)-C(24)-C(23)	107.25(18)
O(5)-C(25)-C(26)	109.5(6)
O(5)-C(25')-C(26)	107.6(5)
O(6)-C(26)-C(25)	115.5(4)
O(6)-C(26)-C(25')	114.1(3)

#1 *y,x,-z*

4. References

[1] P. I. Abronina, A. I. Zinin, A. V. Orlova, S. L. Sedinkin, L. O. Kononov. *Tetrahedron Lett.* **2013**, 54, 4533-4535.

[2] M. Thiele, T. Rose, M. Lõkov, S. Stadtfeld, S. Tshepelevitsh, E. Parman, K. Opara, C. Wölper, I. Leito, S. Grimme, and J. Niemeyer, *Chem. Eur. J.* **2023**, 29, e202202953.

[3] H. Jia, Q. Li, A. Bayaguud, S. She, Y. Huang, K. Chen, Y. Wei, *Sci. Rep.* **2017***, 7,* 12523-12532.

[4] G. M. Sheldrick, *Acta Crystallogr.* **1990**, A46, 467.

[5] G. M. Sheldrick, SHELXL-2017, Program for the Refinement of Crystal Structures University of Göttingen, Göttingen (Germany) **2017**. (see also: G.M. Sheldrick, "Crystal structure refinement with SHELXL", *Acta Cryst.* **2015** *C71*, 3-8).

[6] shelXle, *A Qt GUI for SHELXL*, C. B. Hübschle, G. M. Sheldrick, B. Dittrich, *J. Appl. Cryst.* **2011**, *44*, 1281-1284.

[7] S. Parsons, H. D. Flack, *Acta Cryst*. **2004**, A60, s61 and S. Parsons, H. D. Flack, T. Wagner, *Acta Cryst*. **2013**, B69, 249-259.
